# Supplementary material for: Dissecting regulatory pathways for transcription recovery following DNA damage reveals a non-canonical function of the histone chaperone HIRA
Source: Nat Commun. 2021 Jun 22;12:3835. doi: 10.1038/s41467-021-24153-1 (PMC8219801; doi:10.1038/s41467-021-24153-1)
Supplement: Supplementary file 1 — Supplementary Information [file 41467_2021_24153_MOESM1_ESM.pdf]

Supplementary Figure 1

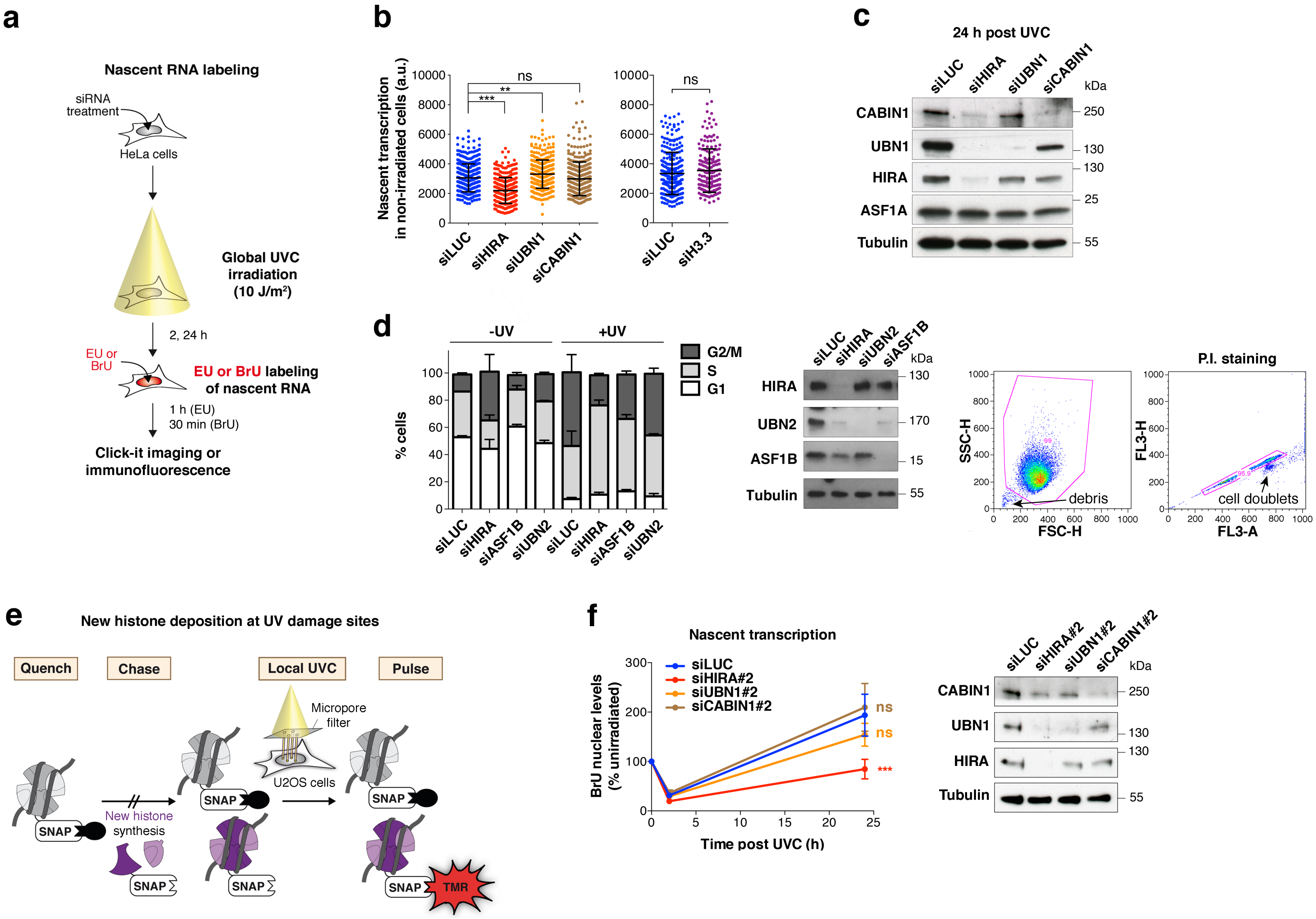

Supplementary Fig. 1: Functional consequences of HIRA complex subunit knockdown

a, Scheme of the procedure for nascent RNA labelling with EU or BrU in HeLa cells treated with siRNAs. Cells were irradiated at different time points after siRNA transfection to be labelled all at the same time with EU or BrU and harvested right after. **b**, Raw transcription data from Fig. 1a and 1d (undamaged conditions). The scatter plots show data from a representative experiment (mean +/- s.d. from at least 183 cells; a.u., arbitrary units). Similar results were obtained in three independent experiments. **c**, siRNA efficiencies for the experiment shown in Fig. 1a controlled by western blot at the end of the experiment (siLUC, control; Tubulin, loading control). **d**, Cell cycle distribution of HeLa cells treated with the indicated siRNAs and analysed by flow cytometry before (-UV) and 24 h after global UVC irradiation at 10 J/m^2 (+UV). Mean +/- s.d. from three independent experiments. siRNA efficiencies are controlled by western blot (Tubulin, loading control). The gating strategy to gate out debris and cell doublets is presented on the right (FSC-H, forward scatter-height; SSC-H, side scatter-height; FL3-A/-H, red fluorescent channel-area/-height; P.I., Propidium Iodide). **e**, Scheme of the assay for monitoring new H3.3 deposition at sites of local UVC irradiation in U2OS H3.3-SNAP cells. Pre-existing SNAP-tagged histones are quenched with a non-fluorescent substrate (block) and histones neo-synthesised during the chase period are labelled with the red fluorescent substrate tetramethylrhodamine (TMR)-star during the pulse step. Local UVC irradiation through micropore filters is performed immediately before the pulse step. **f**, Nascent transcript levels analysed by bromo-uridine (BrU) incorporation in UVC-irradiated HeLa cells treated with the indicated siRNAs. The graphs represent nascent transcript levels relative to unirradiated cells (mean +/- s.e.m. from six independent experiments scoring at least 94 cells per condition). siRNA efficiencies are controlled by western blot (siLUC, control; Tubulin, loading control). Statistical significance is calculated by one-way ANOVA with Bonferroni post-test (b, left), two-sided Student's t-test (b, right) and two-way ANOVA with Bonferroni post-test (f). **: $p<0.01$; ***: $p< 0.001$; ns: $p>0.05$. Source data are provided as a Source Data file.

Supplementary Figure 2

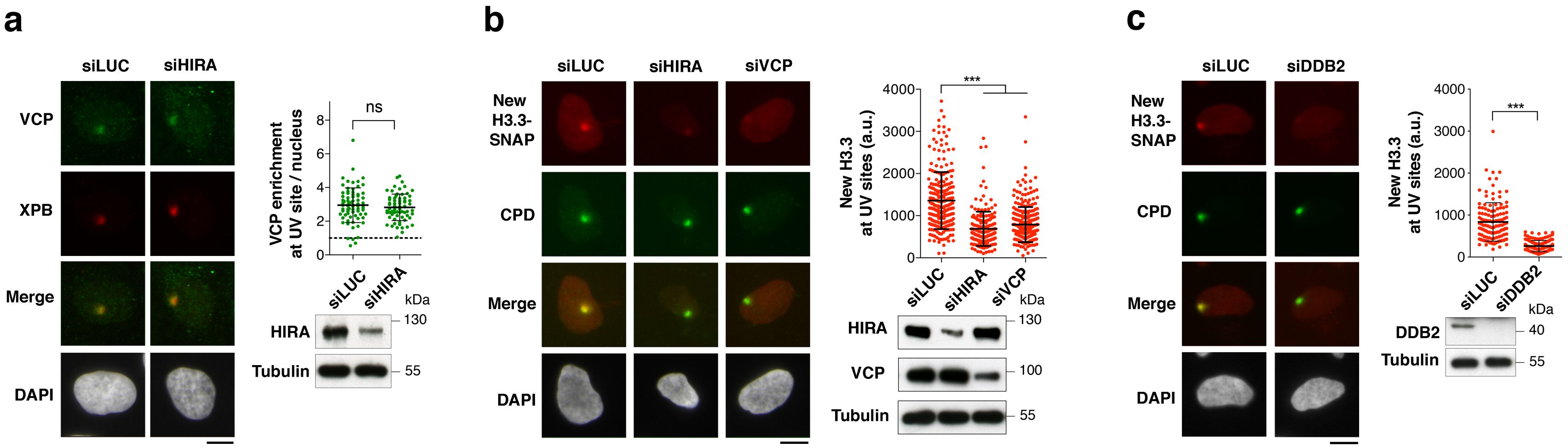

Supplementary Fig. 2: HIRA and VCP interplay at UVC damage sites

a, VCP recruitment to UVC damage sites (marked by the repair factor XPB) analysed by immunostaining 5 min after local UVC irradiation in U2OS cells treated with the indicated siRNAs (siLUC, control). **b**, **c** New H3.3 accumulation at UVC damage sites (marked by CPD immunostaining) analysed 2 h (b) or 1 h (c) post local UVC irradiation in U2OS H3.3-SNAP cells treated with the indicated siRNAs (siLUC, control). siRNA efficiencies are controlled by western blot (Tubulin, loading control). New H3.3 histones were labelled with SNAP-cell TMR star (b) or Oregon green (c) and are always shown in red for consistency. The scatter plots show data from a representative experiment (mean +/- s.d. from at least 67 cells; a.u., arbitrary units). Similar results were obtained in three (a, b) and two (c) independent experiments. Statistical significance is calculated by two-sided Student's t-test with Welch's correction when necessary (a, c) and one-way ANOVA with Bonferroni post-test (b). ***: $p < 0.001$; ns: $p > 0.05$. Scale bar, 10 μm. Source data are provided as a Source Data file.

Supplementary Figure 3

a

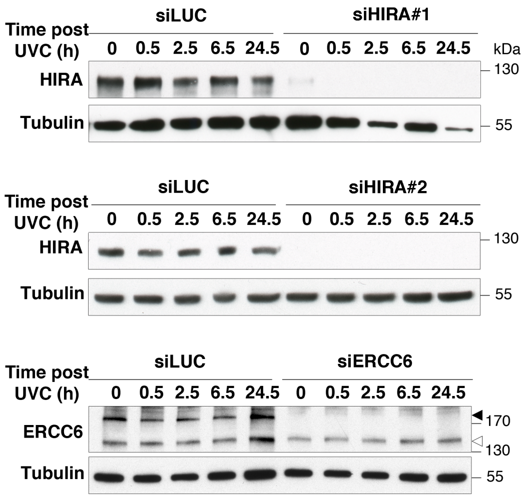

b

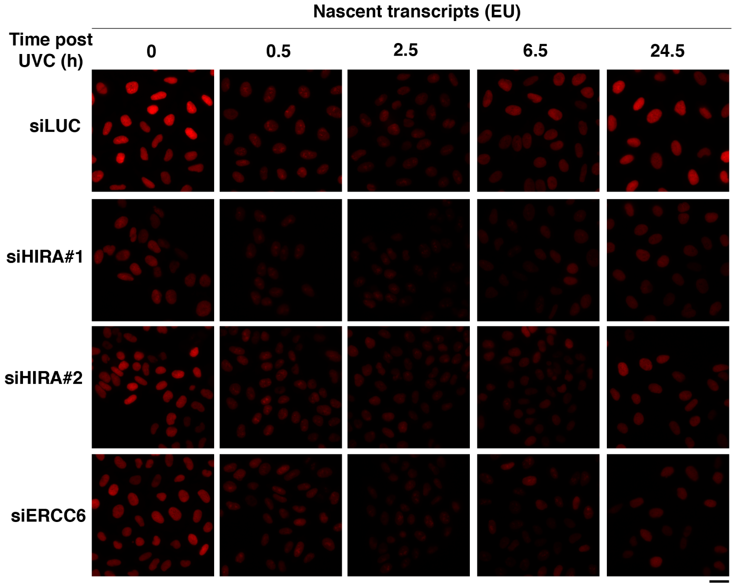

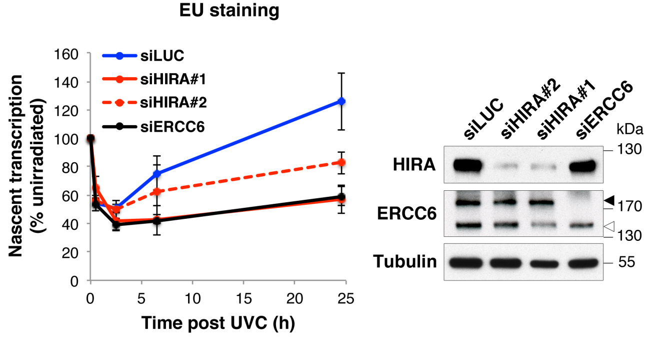

BIOLOGICAL SCALING NORMALIZATION: CORRECTION FACTORS

	Time post UVC (h)				
**siRNA**	**0**	**0.5**	**2.5**	**6.5**	**24.5**
**siLUC**	1	0.540	0.505	0.754	1.259
**siHIRA#1**	1	0.652	0.418	0.432	0.569
**siHIRA#2**	1	0.566	0.496	0.623	0.828
**siERCC6**	1	0.533	0.387	0.421	0.593

c

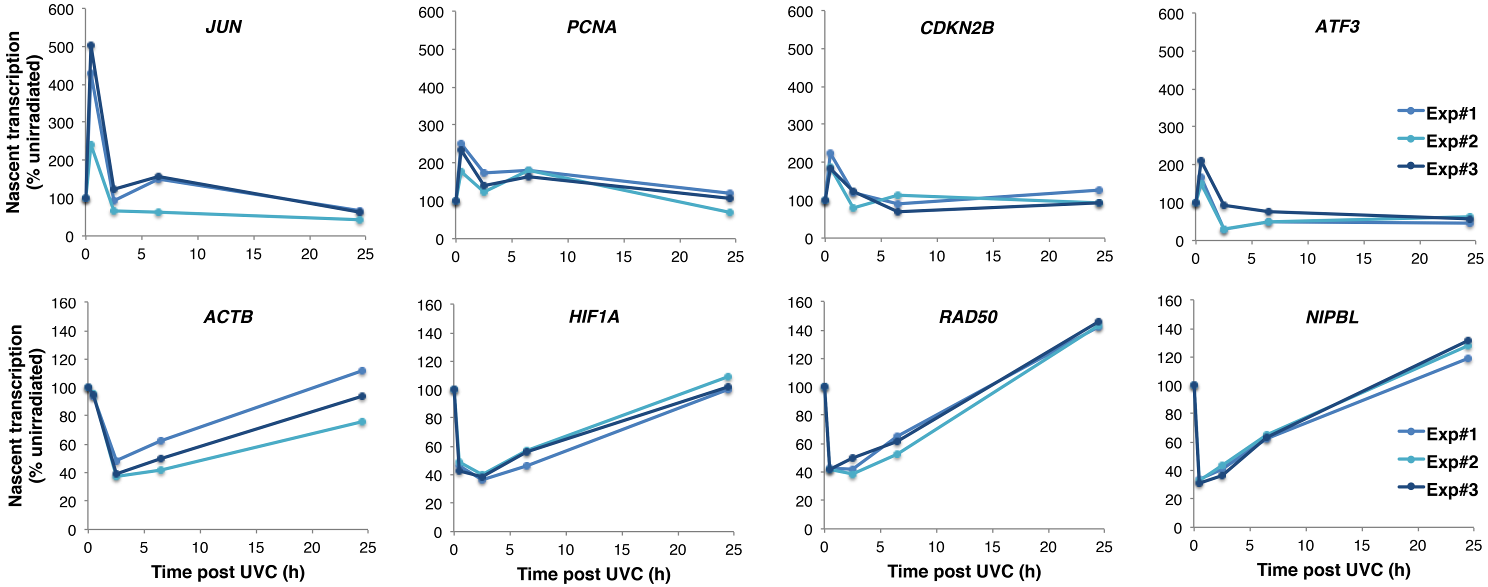

UV-INDUCED GENES

n	(% expressed genes)	median length
702	4%	6.2 kb
425	3%	7.8 kb
759	5%	5.7 kb

UV-REPRESSED GENES

n	(% expressed genes)	median length
6285	38%	48.7 kb
7395	46%	35.7 kb
6898	41%	40.4 kb

Supplementary Fig. 3: Biological scaling normalisation for Bru-seq and examples of UV-regulated genes

a, Western blot controls of HIRA and ERCC6 knock-downs at the indicated time points post UVC irradiation in HeLa cells (siLUC, control; black arrowhead, full-length ERCC6; white arrowhead, ERCC6 splice variant; Tubulin, loading control). Each western blot corresponds to a Bru-seq experiment (n=1). **b**, Ethynyl-uridine (EU) labelling of nascent transcripts in HeLa cells treated with the indicated siRNAs (siLUC, control). Scale bar, 10 μm. The western blot shows the efficiency of protein knock-down by siRNA at the end of the experiment as in (a). The graph represents nascent transcript levels post UVC irradiation normalised to before damage (mean values +/- s.d from three independent experiments). These values are used as correction factors for the normalisation of Bru-seq data (table). **c**, Examples of UV-induced (top) and UV-repressed genes (bottom) analysed by Bru-seq with biological scaling normalisation (three independent experiments). Nascent transcript levels post UVC irradiation normalised to before damage are presented on the graphs. Statistics for each category of genes are shown in the tables (n, number of genes). Source data are provided as a Source Data file.

Supplementary Figure 4

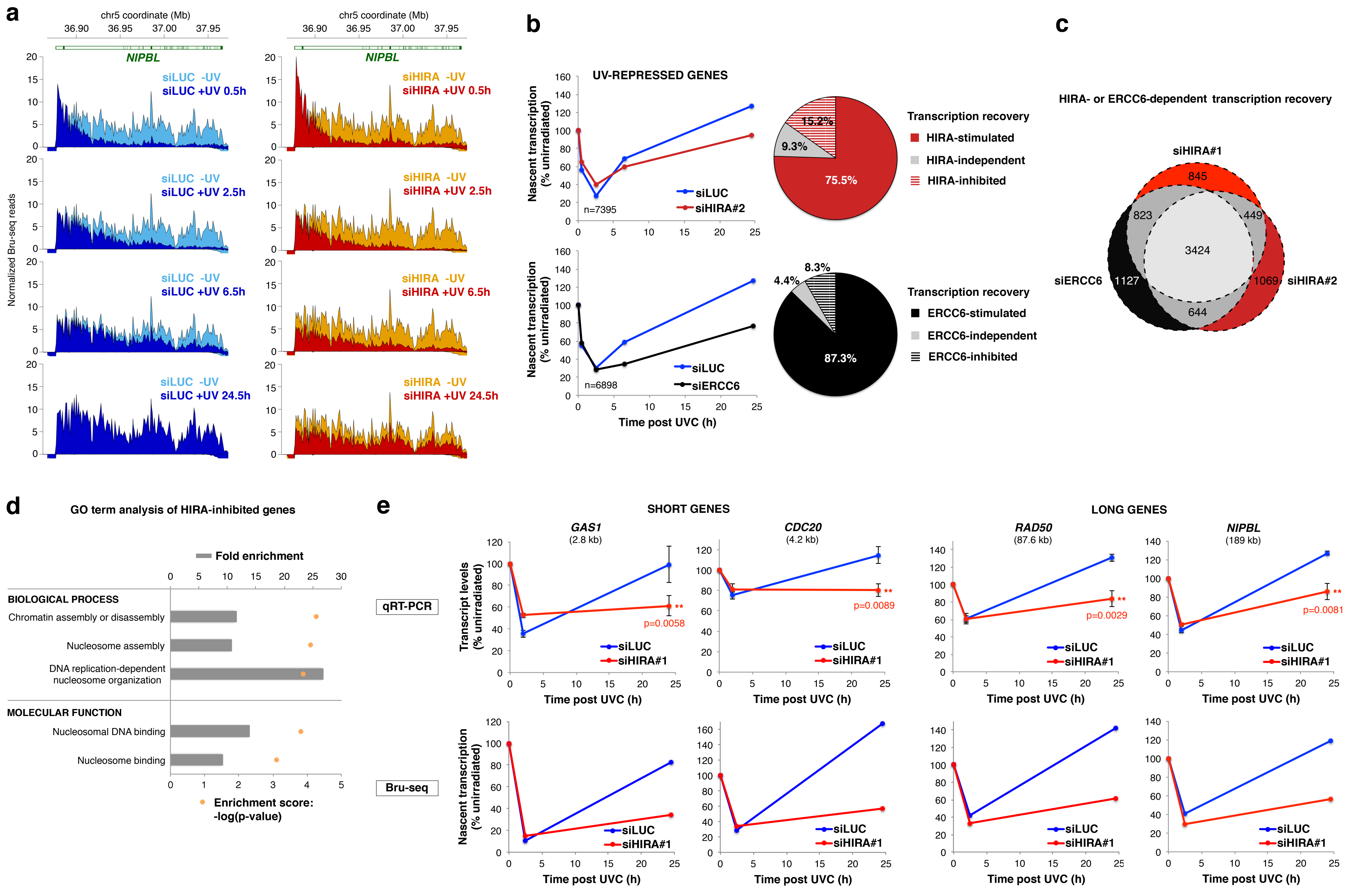

Supplementary Fig. 4: Bru-seq analysis and validation by qRT-PCR

a, Distribution of Bru-seq reads (with biological scaling normalisation) on the *NIPBL* gene over time after UV irradiation (+UV, dark colours) compared to non-irradiated samples (-UV, light colours) in HeLa cells treated with the indicated siRNAs (siLUC, control). **b**, Bru-seq analysis of UV-repressed genes in HeLa cells treated with the indicated siRNAs (siLUC, control). Nascent transcript levels post UVC irradiation normalised to before damage averaged over n genes are presented on the graphs. The pie charts show UV-repressed genes as a function of their dependency on HIRA or ERCC6 for transcription recovery. **c**, Venn diagrams showing the number of genes requiring HIRA or ERCC6 for transcription recovery 24h30 post UVC irradiation. **d**, Gene ontology (GO) analysis of escapees that recover transcription better in the absence of HIRA (HIRA-inhibited genes identified in both experiments with siHIRA#1 and siHIRA#2) using the GOrilla statistical model. **e**, Validation of Bru-seq results by qRT-PCR on total RNA, focusing on short and long genes in HeLa cells treated with the indicated siRNAs (siLUC, control). Bru-seq and qRT-PCR results (mean +/- s.e.m. from three independent experiments) are presented one above the other for comparison. Statistical significance is calculated by two-way ANOVA with Bonferroni post-test. Source data are provided as a Source Data file.

Supplementary Figure 5

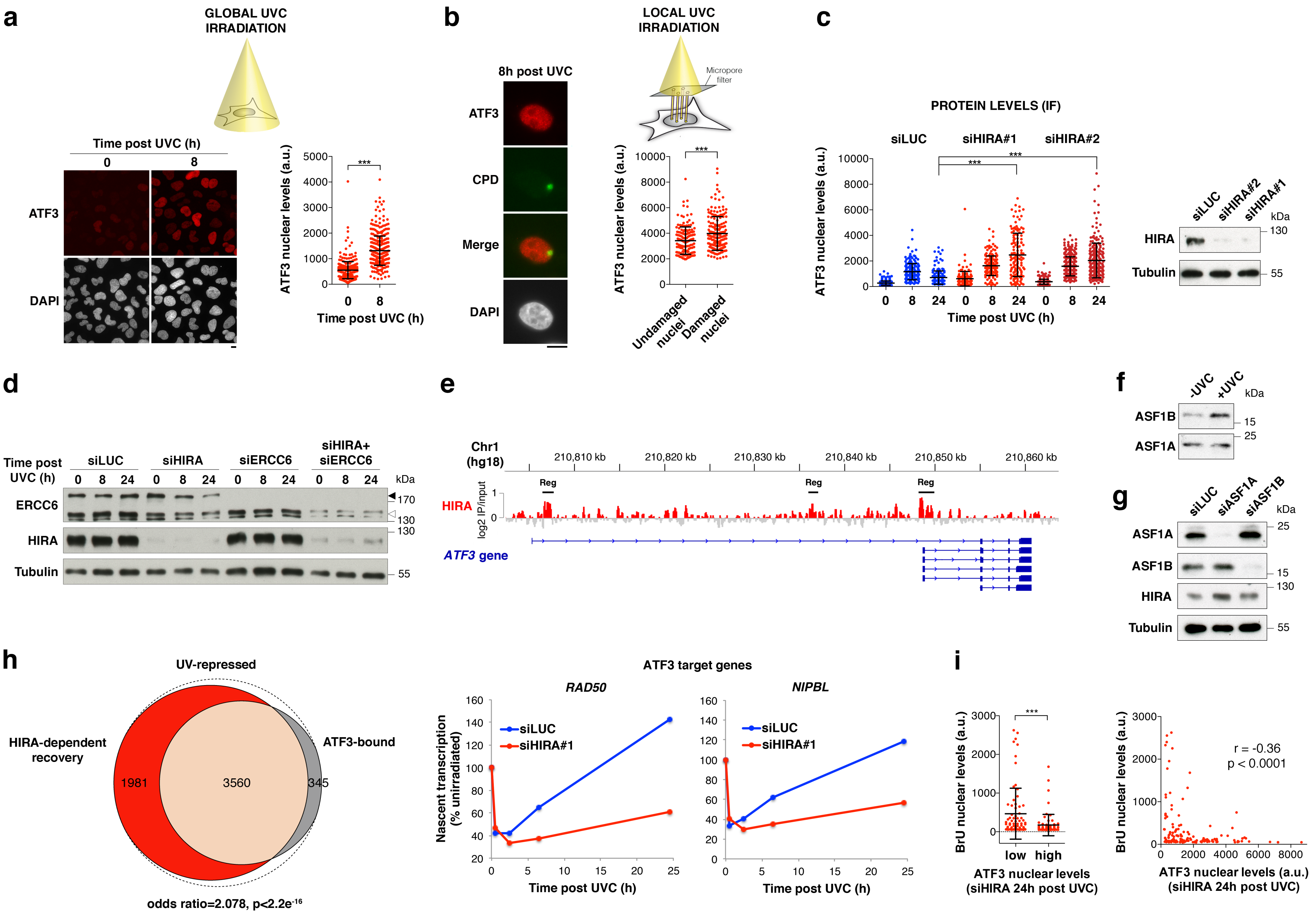

Supplementary Fig. 5: HIRA impact on ATF3 levels following UVC irradiation

a-b, ATF3 levels analysed by immunofluorescence in U2OS cell nuclei 8 h after global (a) or local (b) UVC irradiation. CPD, UVC damage sites. **c**, ATF3 protein levels analysed by immunofluorescence (IF) at the indicated time points post UVC in HeLa cells treated with the indicated siRNAs (siLUC, control). HIRA knock-down is controlled by western blot (Tubulin, loading control). The scatter plots (a, b, c) show ATF3 nuclear levels (mean +/- s.d. from at least 151 cells). Similar results were obtained in two independent experiments. a.u., arbitrary units. **d**, Knock-down efficiencies of data presented in Fig. 6b controlled by western blot (black and white arrowheads, ERCC6 full-length and splice variant; Tubulin, loading control). Similar results were obtained in two independent experiments. **e**, HIRA ChIP-seq profile on the *ATF3* gene in HeLa cells. Reg, regulatory regions. **f**, ASF1A and ASF1B levels analysed by western blot on total extracts from HeLa cells before (-UVC) and 15h after irradiation (+UVC). Equal loading was verified by total protein stain (n=2). **g**, Knock-down efficiencies of data presented in Fig. 6g controlled by western blot (Tubulin, loading control; n=3). **h**, Venn diagram showing the overlap between UV-repressed genes that require HIRA for transcription recovery and those bound by ATF3 (based on Bru-seq experiment with siHIRA#1 and on ATF3 ChIP-seq data 8 h post UVC from Epanchintsev et al., 2017). The graphs show examples of ATF3-target genes that require HIRA for transcription recovery post UVC (Bru-seq data with biological scaling normalisation). **i**, Anti-correlation between ATF3 and BrU nuclear levels in HIRA-knocked down cells 24h post UVC. Left graph, ATF3 levels were partitioned in two groups based on the median value (mean +/- s.d. from 73 cells). Right graph, Spearman correlation. r, correlation coefficient (n=146). Similar results were obtained in three independent experiments. Statistical significance is calculated by two-sided Student's t-test with Welch's correction when necessary (a, b, i left), one-way ANOVA with Bonferroni post-test (c), Fisher's exact test (h), and Spearman correlation test (i right). ***: $p < 0.001$. Scale bars, 10 μm. Source data are provided as a Source Data file.

Supplementary Figure 6

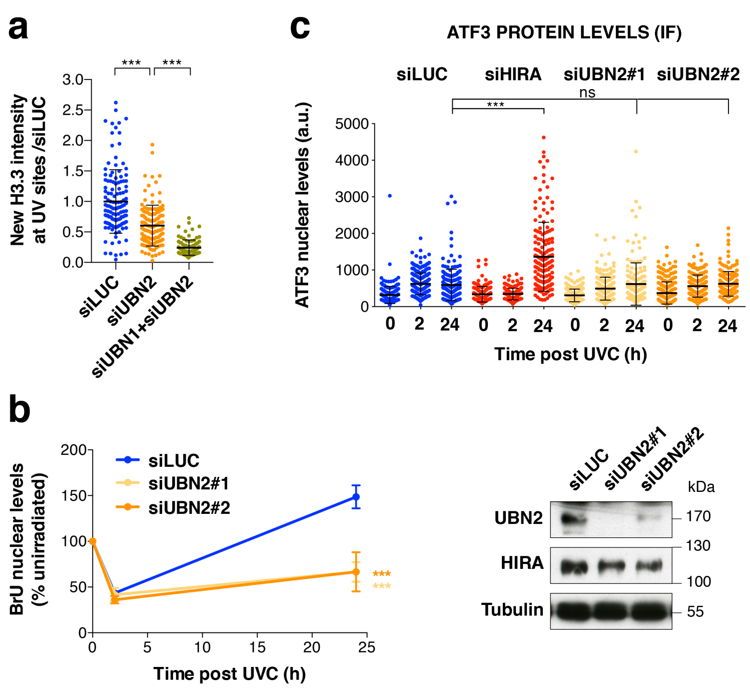

Supplementary Fig. 6: Role of UBN2 in transcription restart after UV damage

a, New H3.3 accumulation at UVC damage sites analysed 2h post local UVC irradiation in U2OS H3.3-SNAP cells treated with the indicated siRNAs (siLUC, control; mean +/- s.d. from at least 95 cells). **b**, Nascent transcript levels analysed by bromo-uridine (BrU) incorporation in UVC-irradiated HeLa cells treated with the indicated siRNAs (siLUC, control). The efficiency of UBN2 knockdown is controlled by western blot (Tubulin, loading control). The graph shows mean values +/- s.d. from three independent experiments scoring at least 118 cells per experiment. **c**, ATF3 nuclear levels analysed by immunofluorescence (IF) at the indicated time points post UVC irradiation in HeLa cells treated with the indicated siRNAs (siLUC, control) in parallel to the experiment shown in panel b. The scatter plot shows mean values +/- s.d. from at least 146 cells. Similar results were obtained in three independent experiments. a.u., arbitrary units. Statistical significance is calculated by one-way (a, c) and two-way ANOVA (b) with Bonferroni post-test. ***: $p< 0.001$; ns: $p>0.05$. Source data are provided as a Source Data file.

SUPPLEMENTARY TABLES

Supplementary Table 1: siRNA sequences

**siRNA**	**Target sequence**
siASF1A	$^{5'}$ AAGTGAAGAATACGATCAAGT $^{3'}$
siASF1B	$^{5'}$ AACAACGAGTACCTCAACCCT $^{3'}$
siATF3	$^{5'}$ GAGGCGACGAGAAAGAAAT $^{3'}$
siCABIN1	$^{5'}$ GGATTGATTTGTCGGACTA $^{3'}$
siCABIN1#2	$^{5'}$ GGAGAAGGATTGCCGGTAC $^{3'}$
siERCC6	$^{5'}$ GAAGAGTTGTCAGTGATTA $^{3'}$
siDDB2	$^{5'}$ TCACTGGGCTGAAGTTTAA $^{3'}$
siLUC (Luciferase)	$^{5'}$ CGTACGCGGAATACTTCGA $^{3'}$
siHIRA#1= siHIRA	$^{5'}$ GAAGGACTCTCGTCTCATG $^{3'}$
siHIRA#2	$^{5'}$ GGAGATGACAAACTGATTA $^{3'}$
siH3.3	1:1 combination of<br>siH3.3A: $^{5'}$ CTACAAAAGCCGCTCGCAA $^{3'}$ and<br>siH3.3B: $^{5'}$ GCTAAGAGAGTCACCATCAT $^{3'}$
siUBN1	$^{5'}$ ATACAGGACTTGATCGATA $^{3'}$
siUBN1#2	$^{5'}$ GCATAAGCCTGTTGCGGTC $^{3'}$
siUBN2#1=siUBN2	$^{5'}$ TCCTGTTCCTCCTCACTTA $^{3'}$
siUBN2#2	$^{5'}$ TCAGGCCTTCCACCTACAA $^{3'}$
siVCP#1=siVCP	$^{5'}$ GAATAGAGTTGTTCGGAAT $^{3'}$
siVCP#2	$^{5'}$ GGAGGTAGATATTGGAATT $^{3'}$

Supplementary Table 2: Antibodies
IF: immunofluorescence; WB: western blot

	Antibody	Species	Dilution	Application	Supplier (reference)
Primary	ASF1A	Rabbit	1:1000	WB	Cell Signaling Technology (2990)
	ASF1B	Rabbit	1:250	WB	Cell Signaling Technology (2902)
	ATF3	Rabbit	1:100/ 1:100	WB/IF	Abcam (ab207434)
		Mouse	1:100	WB	MERCK Millipore (DR1086)
	BrdU	Mouse	1:250	IF	BD Biosciences (555627)
	CABIN1	Rabbit	1:1000	WB	Abcam (ab3349)
	CPD	Mouse	1:1000	IF	Kamiya Biomedical Company (MC-062)
	DDB2	Mouse	1:200	WB	Abcam (ab51017)
	ERCC6	Rabbit	1:500	WB	Santa Cruz Biotechnology (sc-25370)
	ERCC6	Rabbit	1:1000	WB	Euromedex (GTX104589)
	HIRA	Mouse	1:200/ 1:100	WB/IF	Active Motif (39557)
	RNAPII	Rabbit	1:500	WB	Santa Cruz Biotechnology (sc-899)
	RNAPII Ser2-P	Rabbit	1:10000	WB	Abcam (ab5095)
	Tubulin	Mouse	1:10000	WB	Sigma-Aldrich (T9026-.2ML)
	UBN1	Rabbit	1:2000	WB	Abcam (ab101282)
	UBN2	Rabbit	1:1000	WB	Antibodies-online (ABIN5999135)
	Ubiquitin	Mouse	1:200	WB	Eurogentec (MMS-257P-0200)
	VCP	Mouse	1:2000/ 1:1000	WB/IF	Abcam (ab11433)
	XPB	Rabbit	1:400	IF	Santa Cruz Biotechnology (sc-293)
Secondary	Anti-Mouse HRP	Goat	1:10000	WB	Jackson Immunoresearch (115-035-068)
	Anti-Rabbit HRP	Donkey	1:10000	WB	Jackson Immunoresearch (711-035-152)
	Anti-Mouse AlexaFluor 488	Goat	1:10000	IF	Invitrogen (A11029)
	Anti-Rabbit AlexaFluor 594	Goat	1:10000	IF	Invitrogen (A11037)
	Anti-Rabbit IRDye 680RD Conjugated	Goat	1:15000	WB	LI-COR Biosciences (926-68071)
	Anti-Rabbit IRDye 800CW Conjugated	Goat	1:15000	WB	LI-COR Biosciences (926-32211)

	Anti-Mouse IRDye 680RD Conjugated	Goat	1:15000	WB	LI-COR Biosciences (926-68070)
	Anti-Mouse IRDye 800CW Conjugated	Goat	1:15000	WB	LI-COR Biosciences (926-32210)

Supplementary Table 3: qRT-PCR primers
F: forward; R: reverse.

**Designation**	**Sequence**
ATF3_F	5' GGAGTGCCTGCAGAAAGAGT 3'
ATF3_R	5' CCATTCTGAGCCCGGACAAT 3'
CDC20_F	5' AGTACTCTACAGCCAAAAGGC 3'
CDC20_R	5' AGGGAAGGAATGTAACGGCA 3'
GAPDH_F	5' CAAGGCTGTGGGCAAGGT 3'
GAPDH_R	5' GGAAGGCCATGCCAGTGA 3'
GAS1_F	5' GAAACTCCCAACTCGTCTGC 3'
GAS1_R	5' ACCTTCCCTTTCGAGTCCAG 3'
NIPBL_F	5' AGAGCAGAGTGAGAAAGCGG 3'
NIPBL_R	5' TCCTCTTGCTGGTCCATGTC 3'
RAD50_F	5' CATGTTGCCCCGTTTGTCAG 3'
RAD50_R	5' TTTCATCACGCCGCTTTTCC 3'
