# Supplementary material for: Dissecting regulatory pathways for transcription recovery following DNA damage reveals a non-canonical function of the histone chaperone HIRA
Source: Nat Commun. 2021 Jun 22;12:3835. doi: 10.1038/s41467-021-24153-1 (PMC8219801; doi:10.1038/s41467-021-24153-1)
Supplement: Supplementary file 3 — Description of Additional Supplementary Files [file 41467_2021_24153_MOESM3_ESM.pdf]

Description of Additional Supplementary Files

File Name: Supplementary Data 1
Description: **Bru-seq in UV-irradiated HeLa cells upon HIRA knockdown (siHIRA#1).**
RPKM values before and after biological scaling normalization for all genes with RPKM>0 at the indicated time points after UVC irradiation in HeLa cells treated with the indicated siRNAs (siLUC, control).

File Name: Supplementary Data 2
Description: **Bru-seq in UV-irradiated HeLa cells upon HIRA knockdown (siHIRA#2).**
RPKM values before and after biological scaling normalization for all genes with RPKM>0 at the indicated time points after UVC irradiation in HeLa cells treated with the indicated siRNAs (siLUC, control).

File Name: Supplementary Data 3
Description: **Bru-seq in UV-irradiated HeLa cells upon ERCC6 knockdown.**
RPKM values before and after biological scaling normalization for all genes with RPKM>0 at the indicated time points after UVC irradiation in HeLa cells treated with the indicated siRNAs (siLUC, control).
