# Supplementary material for: Dissecting regulatory pathways for transcription recovery following DNA damage reveals a non-canonical function of the histone chaperone HIRA
Source: Nat Commun. 2021 Jun 22;12:3835. doi: 10.1038/s41467-021-24153-1 (PMC8219801; doi:10.1038/s41467-021-24153-1)
Supplement: Supplementary file 7 — Reporting Summary [file 41467_2021_24153_MOESM7_ESM.pdf]

nature research

Corresponding author(s): Sophie E. Polo

Last updated by author(s): May 6, 2021

Reporting Summary

Nature Research wishes to improve the reproducibility of the work that we publish. This form provides structure for consistency and transparency in reporting. For further information on Nature Research policies, see our Editorial Policies and the Editorial Policy Checklist.

Statistics

For all statistical analyses, confirm that the following items are present in the figure legend, table legend, main text, or Methods section.

n/a	Confirmed	
☐	☒	The exact sample size (*n*) for each experimental group/condition, given as a discrete number and unit of measurement
☐	☒	A statement on whether measurements were taken from distinct samples or whether the same sample was measured repeatedly
☐	☒	The statistical test(s) used AND whether they are one- or two-sided<br>*Only common tests should be described solely by name; describe more complex techniques in the Methods section.*
☒	☐	A description of all covariates tested
☐	☒	A description of any assumptions or corrections, such as tests of normality and adjustment for multiple comparisons
☐	☒	A full description of the statistical parameters including central tendency (e.g. means) or other basic estimates (e.g. regression coefficient) AND variation (e.g. standard deviation) or associated estimates of uncertainty (e.g. confidence intervals)
☐	☒	For null hypothesis testing, the test statistic (e.g. *F*, *t*, *r*) with confidence intervals, effect sizes, degrees of freedom and *P* value noted<br>*Give P values as exact values whenever suitable.*
☒	☐	For Bayesian analysis, information on the choice of priors and Markov chain Monte Carlo settings
☒	☐	For hierarchical and complex designs, identification of the appropriate level for tests and full reporting of outcomes
☒	☐	Estimates of effect sizes (e.g. Cohen's *d*, Pearson's *r*), indicating how they were calculated

Our web collection on statistics for biologists contains articles on many of the points above.

Software and code

Policy information about availability of computer code

Data collection	Metamorph v.7.10.2.240 was used for image acquisition. CellQuest Pro v. 6.0 was used for collecting flow cytometry data.
Data analysis	Image Studio Lite v.5.2.5 was used for western blot quantifications. Image J v.2.0.0-rc-43/1.50i and ICY v.1.8.5.1 were used for image analyses. Flow cytometry data were analyzed with FlowJo v.9.9.5. Quantitative PCR analyses were performed with Applied Biosystems 7500 Fast v.2.3. R v.3.5.1 and Graphpad Prism (versions 6 to 9) were used for statistical analyses. Gene ontology analyses were performed with GOrilla95 (database version Jan 19, 2019). Analysis of nascent RNA sequencing data (Bru-seq) was done using Illumina Casava v.1.8.2 for base calling, Bowtie v.0.12.8 and TopHat v.1.4.1 for read mapping, Bedtools v.2.16.2 for genome annotation as described in Paulsen MT et al., Methods 67:45-54, 2014. Integrative Genomics Viewer (IGV 2.3) was used for the representation of ChIP-seq data.

For manuscripts utilizing custom algorithms or software that are central to the research but not yet described in published literature, software must be made available to editors and reviewers. We strongly encourage code deposition in a community repository (e.g. GitHub). See the Nature Research guidelines for submitting code & software for further information.

Data

Policy information about availability of data

All manuscripts must include a data availability statement. This statement should provide the following information, where applicable:

- Accession codes, unique identifiers, or web links for publicly available datasets
- A list of figures that have associated raw data
- A description of any restrictions on data availability

All data generated during this study are included in this article and its supplementary information files. Source data for Figures 1-7 and Supplementary figures 1-6 are provided with the paper. The accession code for RNA sequencing data is GSE151833 (https://www.ncbi.nlm.nih.gov/geo/query/acc.cgi?acc=GSE151833) as indicated in the method section and data availability section of the manuscript.

Field-specific reporting

Please select the one below that is the best fit for your research. If you are not sure, read the appropriate sections before making your selection.

☒ Life sciences ☐ Behavioural & social sciences ☐ Ecological, evolutionary & environmental sciences

For a reference copy of the document with all sections, see nature.com/documents/nr-reporting-summary-flat.pdf

Life sciences study design

All studies must disclose on these points even when the disclosure is negative.

Sample size	When analysing fluorescence microscopy images, we aimed for a sample size of 100-150 cells per condition per experiment because it provided us with sufficient power to discriminate between experimental conditions and ensured the robustness of our analysis. For FACS analyses, 20 000 cells were analyzed per sample, consistent with previously published protocols. For Bru-seq experiments, sample size was the number of genes in each category of interest (UV-repressed vs. induced, short vs. long, high vs. low expression, protein coding vs. non-coding) based on criteria detailed in the method section. For all other experiments, sample size was taken as the number of biological replicates.
Data exclusions	S-phase cells were excluded from TCR assay analyses due to pan-nuclear EdU staining in S-phase, which precludes the quantification of local EdU staining at UV sites. Genes with null RPKM values in undamaged conditions were excluded from Bru-seq analyses to be able to compute ratios with transcript levels measured in undamaged cells. All the data exclusion criteria were pre-established.
Replication	Most experiments have been replicated at least 3 times in this study. All replicates were successful. The number of replicates is indicated in all figure legends.
Randomization	Randomization was not necessary because cell types and conditions were confirmed by western-blot, immunofluorescence, or RT-qPCR independently of the experiment outcomes.
Blinding	Experimenters were not blinded to the sample types and experimental conditions. This was not necessary because our study did not include human patient cohorts. Furthermore, the cells to be imaged were randomly selected and analyses and quantifications of fluorescence microscopy images were automated using ImageJ macros, therefore minimizing the subjectivity of the experimenters. Other experiments also did not need blinding since the data was obtained and analyzed in an unbiased manner .

Reporting for specific materials, systems and methods

We require information from authors about some types of materials, experimental systems and methods used in many studies. Here, indicate whether each material, system or method listed is relevant to your study. If you are not sure if a list item applies to your research, read the appropriate section before selecting a response.

Materials & experimental systems

n/a	Involved in the study	
☐	☒	Antibodies
☐	☒	Eukaryotic cell lines
☒	☐	Palaeontology and archaeology
☒	☐	Animals and other organisms
☒	☐	Human research participants
☒	☐	Clinical data
☒	☐	Dual use research of concern

Methods

n/a	Involved in the study	
☒	☐	ChIP-seq
☐	☒	Flow cytometry
☒	☐	MRI-based neuroimaging

Antibodies

Antibodies used

ASF1A, Cell Signaling Technology (2990)
ASF1B, Cell Signaling Technology (2902)
ATF3, Abcam (ab207434)
ATF3, MERCK Millipore (DR1086)
BrdU, BD Biosciences (555627)
CABIN1, Abcam (ab3349)
CPD, Kamiya Biomedical Company (MC-062)
DDB2, Abcam (ab51017)
ERCC6, Santa Cruz Biotechnology (sc-25370)
ERCC6, Euromedex (GTX104589)
HIRA, Active Motif (39557)
RNAPII, Santa Cruz Biotechnology (sc-899)
RNAPII Ser2-P, Abcam (ab5095)

	Tubulin, Sigma-Aldrich (T9026-.2ML)<br>UBN1, Abcam (ab101282)<br>UBN2, Antibodies-online (ABIN5999135)<br>Ubiquitin, Eurogentec (MMS-257P-0200)<br>VCP, Abcam (ab11433)<br>XPB, Santa Cruz Biotechnology (sc-293)<br>Anti-Mouse HRP, Jackson Immunoresearch (115-035-068)<br>Anti-Rabbit HRP, Jackson Immunoresearch (711-035-152)<br>Anti-Mouse AlexaFluor 488, Invitrogen (A11029)<br>Anti-Rabbit AlexaFluor 594, Invitrogen (A11037)<br>Anti-Rabbit IRDye 680RD Conjugated, LI-COR Biosciences (926-68071)<br>Anti-Rabbit IRDye 800CW Conjugated, LI-COR Biosciences (926-32211)<br>Anti-Mouse IRDye 680RD Conjugated, LI-COR Biosciences (926-68070)<br>Anti-Mouse IRDye 800CW, LI-COR Biosciences (926-32210)<br>Anti-Mouse IgG coupled to Dynabeads, Invitrogen (11033)
Validation	All antibodies were purchased from commercial vendors, who provide validation information on their website. In addition, antibody specificity was validated in our lab by western blot and immunofluorescence in control vs siRNA knockdown conditions.<br><br>Data provided in the manuscript validating key antibodies used in this study:<br>- HIRA, UBN1 and CABIN1 antibodies are validated by their corresponding siRNAs (WB Fig. 1a-b, 6d)<br>- UBN2 antibody is validated by siUBN2 (WB Fig. 7a-b, Suppl. Fig. 6b)<br>- H3.3 antibody is validated by siH3.3 (WB Fig. 1d)<br>- ERCC6 antibody is validated by siERCC6 (WB Fig. 2c, 3d)<br>- VCP antibody is validated by siVCP (WB Fig. 3a, 3c)<br>- ATF3 antibody is validated by siATF3 (WB Fig. 6h, 6j)<br>- DDB2 antibody is validated by siDDB2 (WB Suppl. Fig. 2c)<br>- ASF1A and ASF1B antibodies are validated by their corresponding siRNAs (WB Suppl. Fig. 5g)<br><br>Validation/specificity statements on manufacturer's websites for key antibodies used in this study:<br>- CPD antibody:<br>Reacts specifically with thymine dimers produced by UV irradiation in double-or single-stranded DNA. Does not react with (6-4) photo products (https://www.kamiyabiomedical.com/pdf/MC-062.pdf)<br>-BrdU antibody:<br>The 3D4 monoclonal antibody reacts with BrdU, but not other nucleotides, in single-stranded DNA (https://www.bdbiosciences.com/eu/applications/research/clinical-research/oncology-research/proliferation-cell-cycle-and-cell-death/intracellular/cell-signalling-and-transcription-factors/human/purified-mouse-anti--brdu-3d4/p/555627)

Eukaryotic cell lines

Policy information about cell lines

Cell line source(s)	U2OS (ATCC HTB-96, human osteosarcoma, female)<br>HeLa (American Type Culture Collection ATCC CCL-2, human cervical carcinoma, female)<br>XP4PA-SV (Coriell Institute for Medical Research, GM15983, human XPC-deficient skin fibroblasts, male)
Authentication	None of the cells used were authenticated in this study.
Mycoplasma contamination	U2OS, HeLa and XP4PA-SV cell lines tested negative for mycoplasma contamination.
Commonly misidentified lines<br>(See ICLAC register)	This study did not involve misidentified cell lines.

Flow Cytometry

Plots

Confirm that:

☒ The axis labels state the marker and fluorochrome used (e.g. CD4-FITC).

☒ The axis scales are clearly visible. Include numbers along axes only for bottom left plot of group (a 'group' is an analysis of identical markers).

☒ All plots are contour plots with outliers or pseudocolor plots.

☒ A numerical value for number of cells or percentage (with statistics) is provided.

Methodology

Sample preparation	Cells were fixed in ice-cold 70% ethanol before DNA staining with 50 µg/ml propidium iodide (Sigma-Aldrich) in PBS containing 0.05% Tween and 0.5 mg/ml RNase A (USB/Affymetrix).
Instrument	BD FACScalibur flow cytometer (BD Biosciences)

Software	FlowJo software (TreeStar)
Cell population abundance	We did not perform cell sorting.
Gating strategy	Cell debris were gated out using FSC/SSC and cell doublets with FL3-A/FL3-H.

☒ Tick this box to confirm that a figure exemplifying the gating strategy is provided in the Supplementary Information.
