# Supplementary material for: Dissecting regulatory pathways for transcription recovery following DNA damage reveals a non-canonical function of the histone chaperone HIRA
Source: Nat Commun. 2021 Jun 22;12:3835. doi: 10.1038/s41467-021-24153-1 (PMC8219801; doi:10.1038/s41467-021-24153-1)
Supplement: Supplementary file 8 — Source Data [file 41467_2021_24153_MOESM8_ESM.zip › RawData/Main Figures/Fig1/b/Key.docx]

**Key**

w1DAPI : DAPI

w2GFP : CPD

w3CY3 : new H3.3-SNAP
