# Supplementary figures and images for: Dissecting regulatory pathways for transcription recovery following DNA damage reveals a non-canonical function of the histone chaperone HIRA (part 2 of 2)
Source: Nat Commun. 2021 Jun 22;12:3835. doi: 10.1038/s41467-021-24153-1 (PMC8219801; doi:10.1038/s41467-021-24153-1)

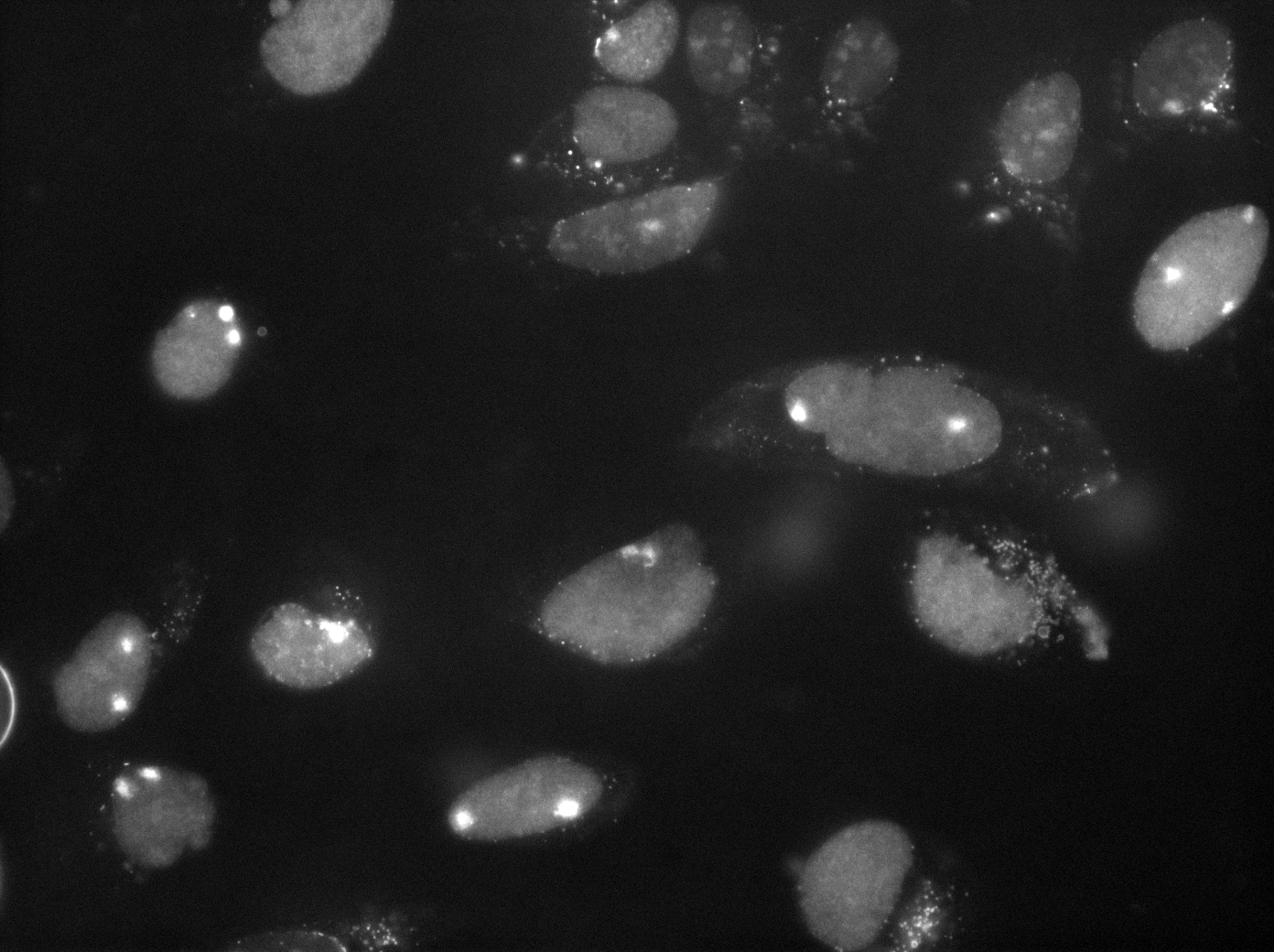

Supplement: Supplementary file 8 — Source Data [file 41467_2021_24153_MOESM8_ESM.zip › RawData/Main Figures/Fig6/j/U3S_150J_siHIRAplusATF3_09_w3CY3.TIF]

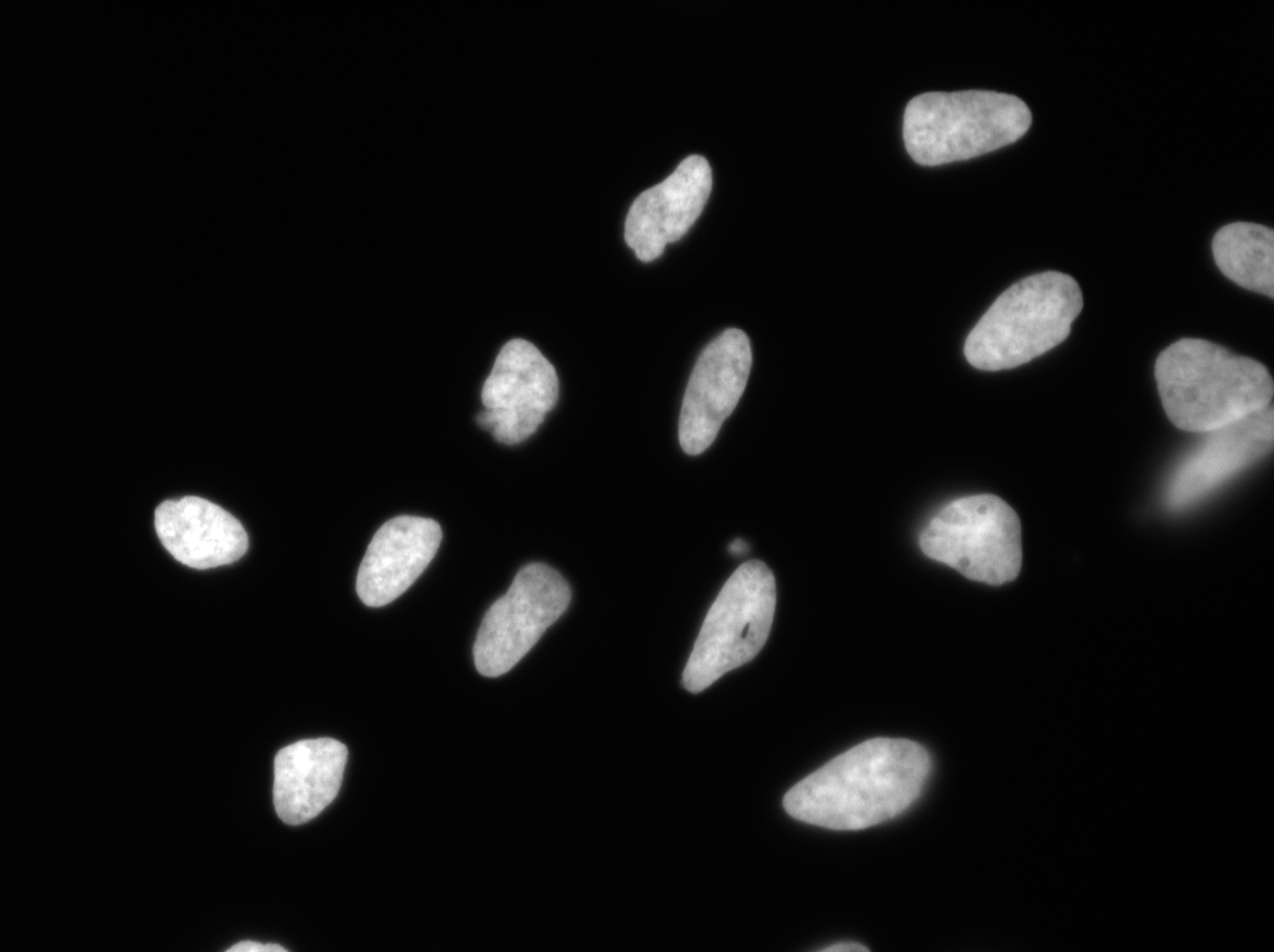

Supplement: Supplementary file 8 — Source Data [file 41467_2021_24153_MOESM8_ESM.zip › RawData/Main Figures/Fig6/j/U3S_150J_siLUC_04_w1DAPI.TIF]

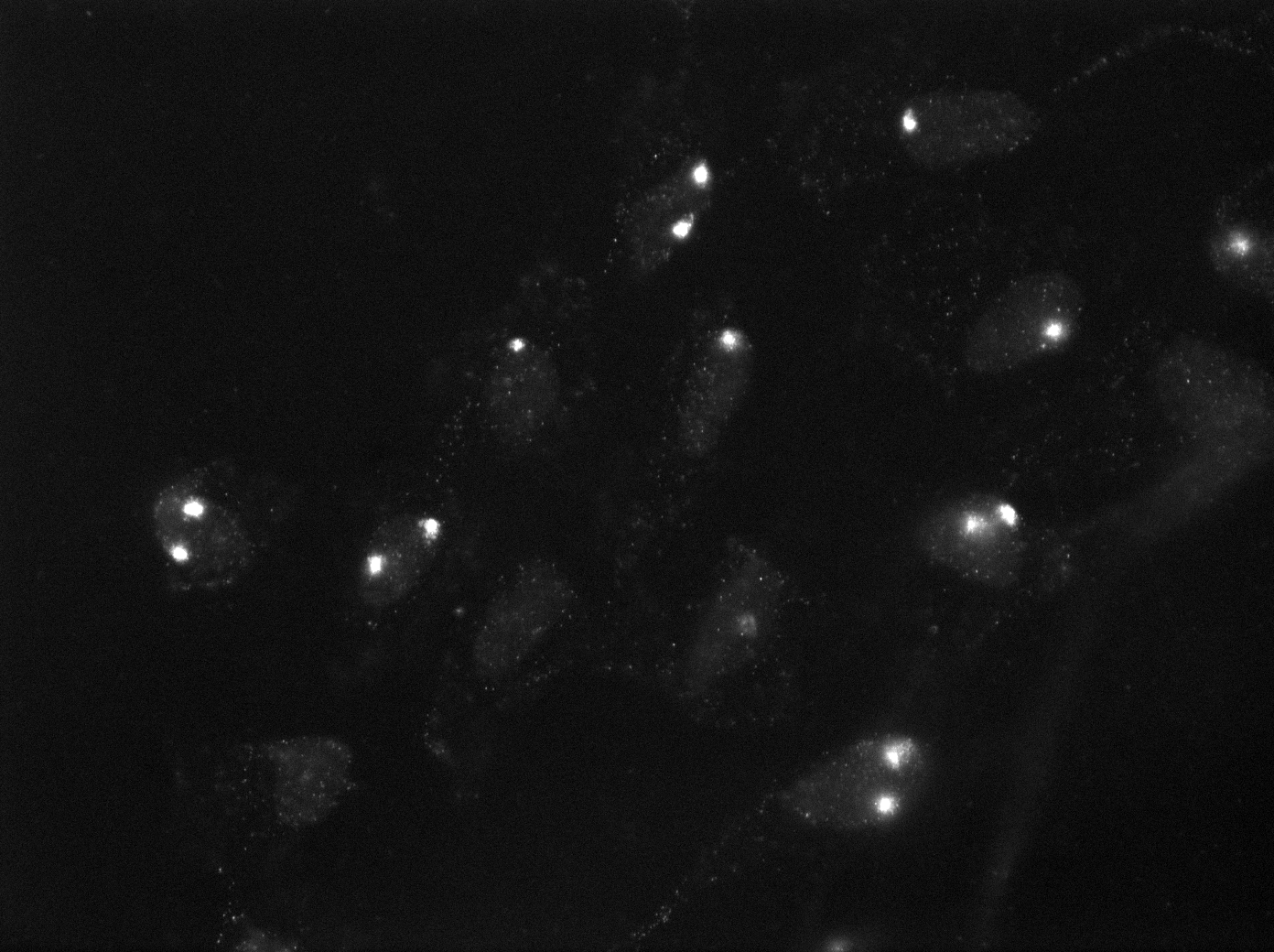

Supplement: Supplementary file 8 — Source Data [file 41467_2021_24153_MOESM8_ESM.zip › RawData/Main Figures/Fig6/j/U3S_150J_siLUC_04_w2GFP.TIF]

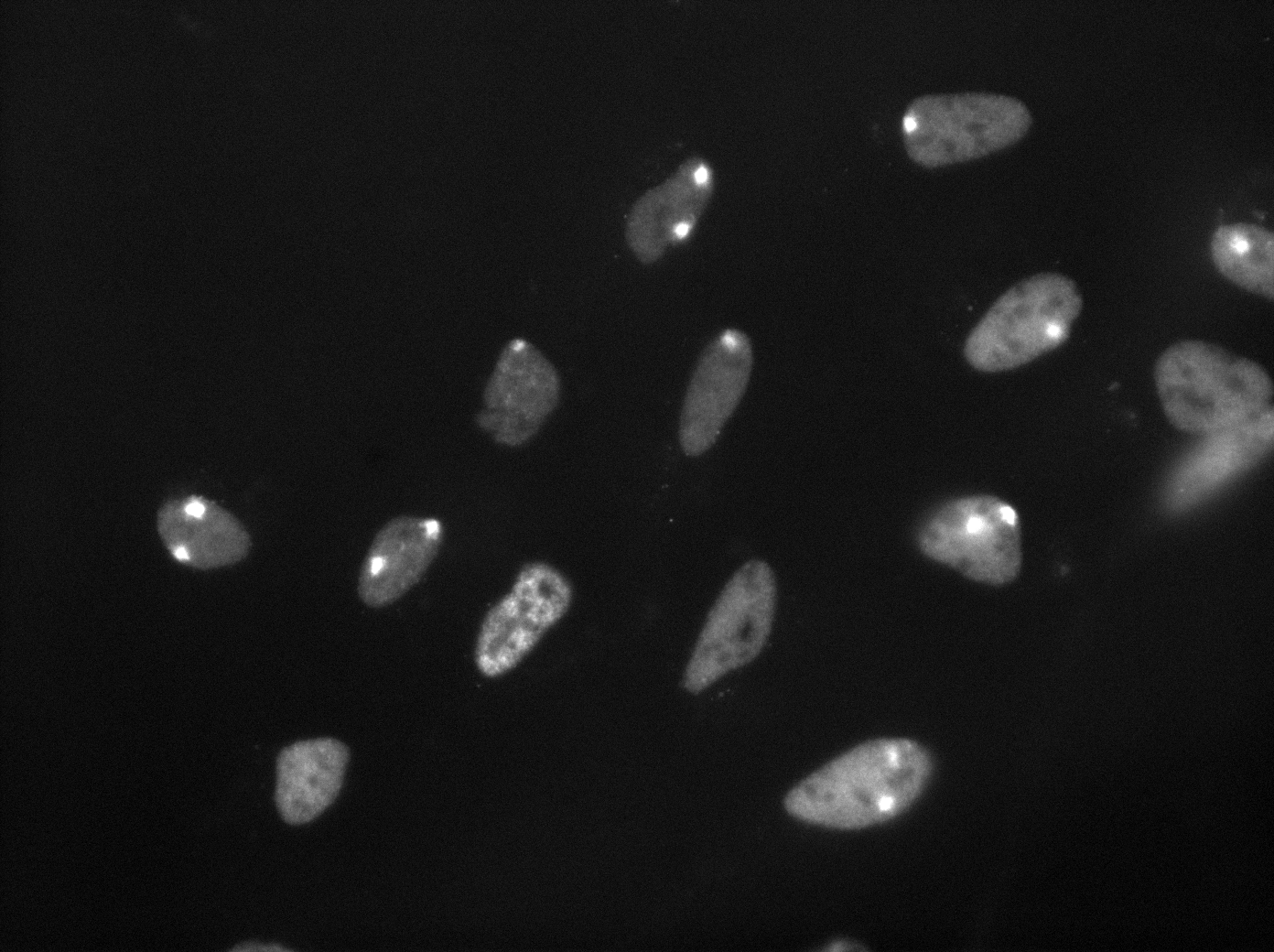

Supplement: Supplementary file 8 — Source Data [file 41467_2021_24153_MOESM8_ESM.zip › RawData/Main Figures/Fig6/j/U3S_150J_siLUC_04_w3CY3.TIF]

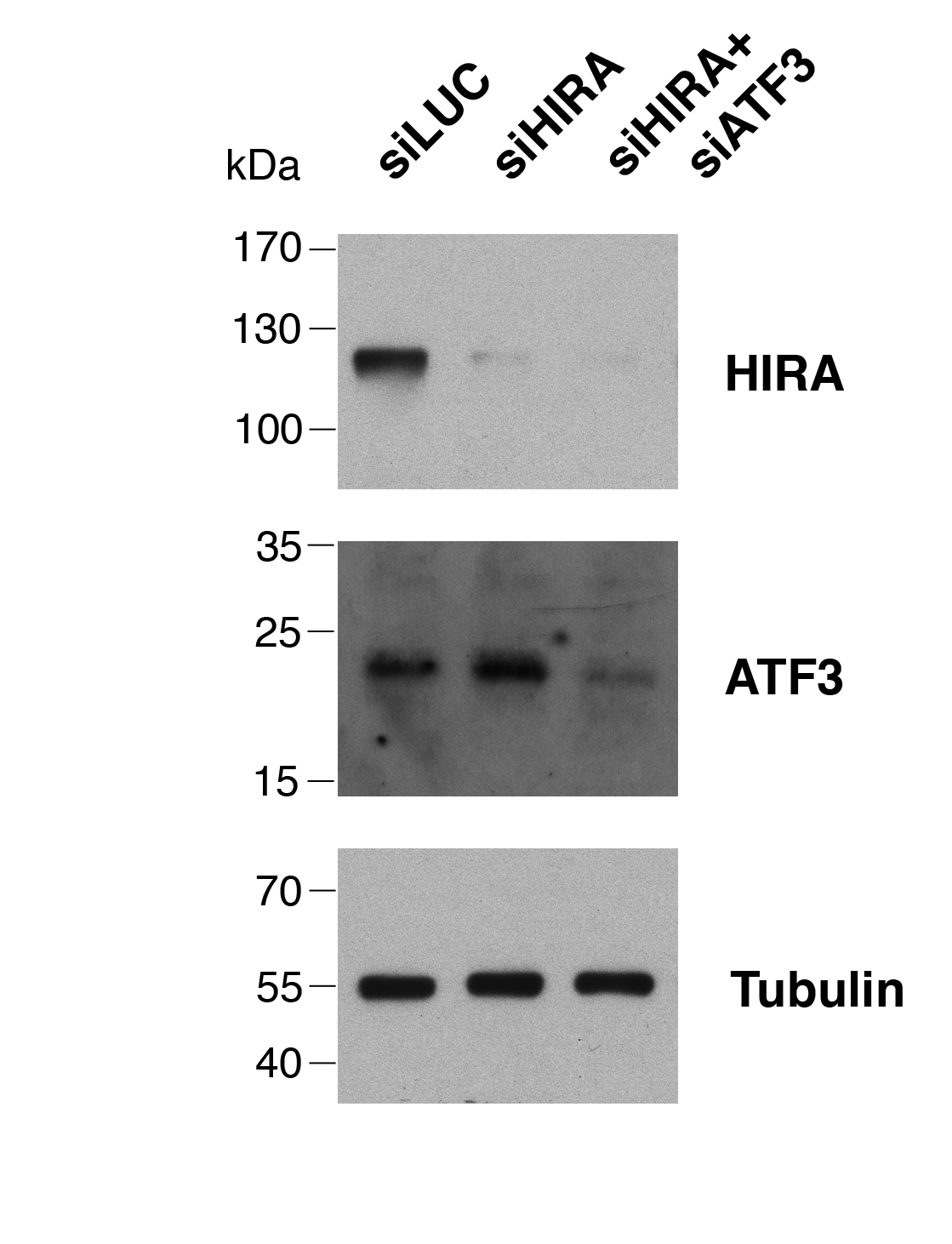

Supplement: Supplementary file 8 — Source Data [file 41467_2021_24153_MOESM8_ESM.zip › RawData/Main Figures/Fig6/j/WB.tif]

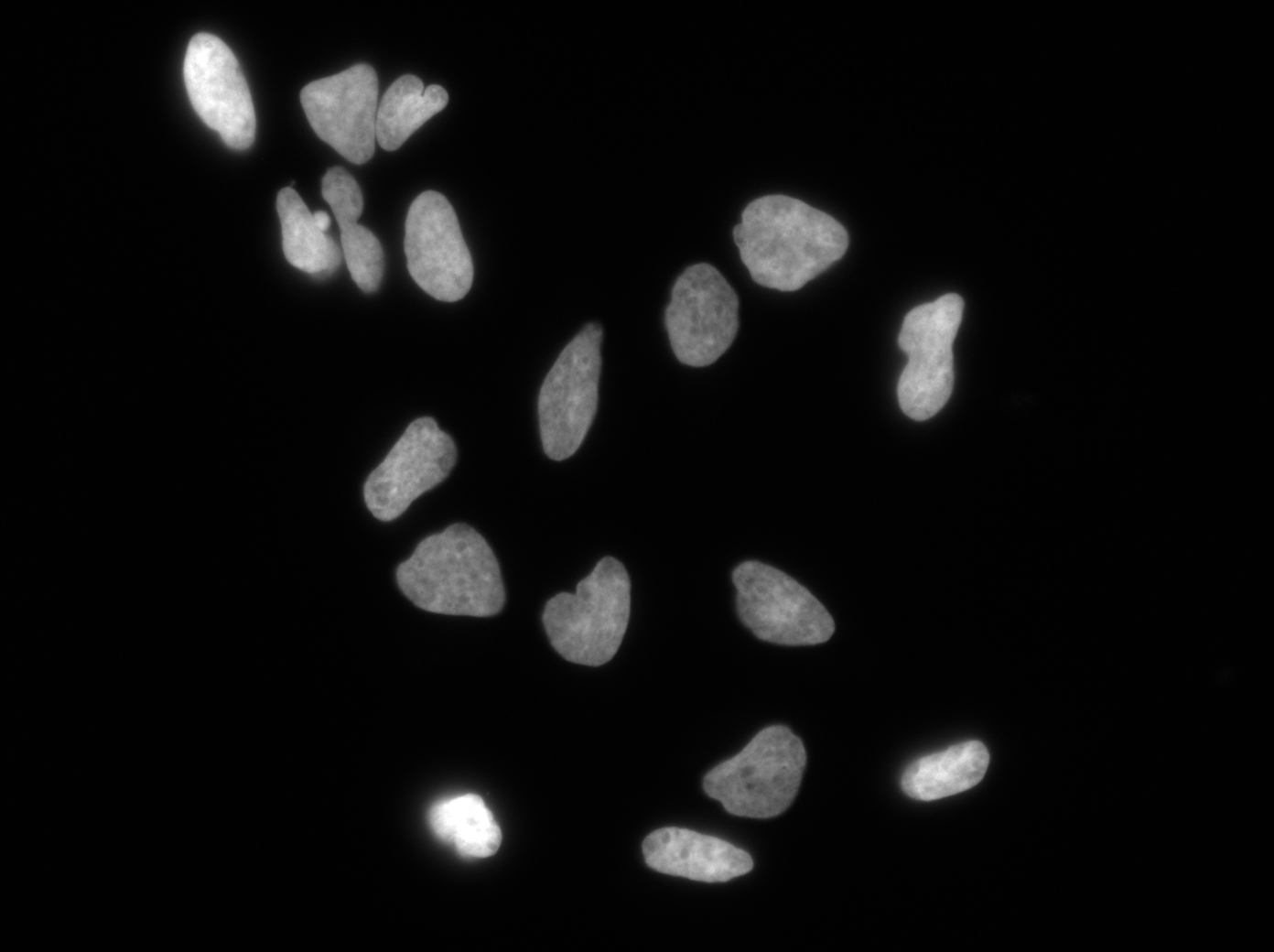

Supplement: Supplementary file 8 — Source Data [file 41467_2021_24153_MOESM8_ESM.zip › RawData/Main Figures/Fig7/a/U3S_QCUVP_siLUC_09_w1DAPI.TIF]

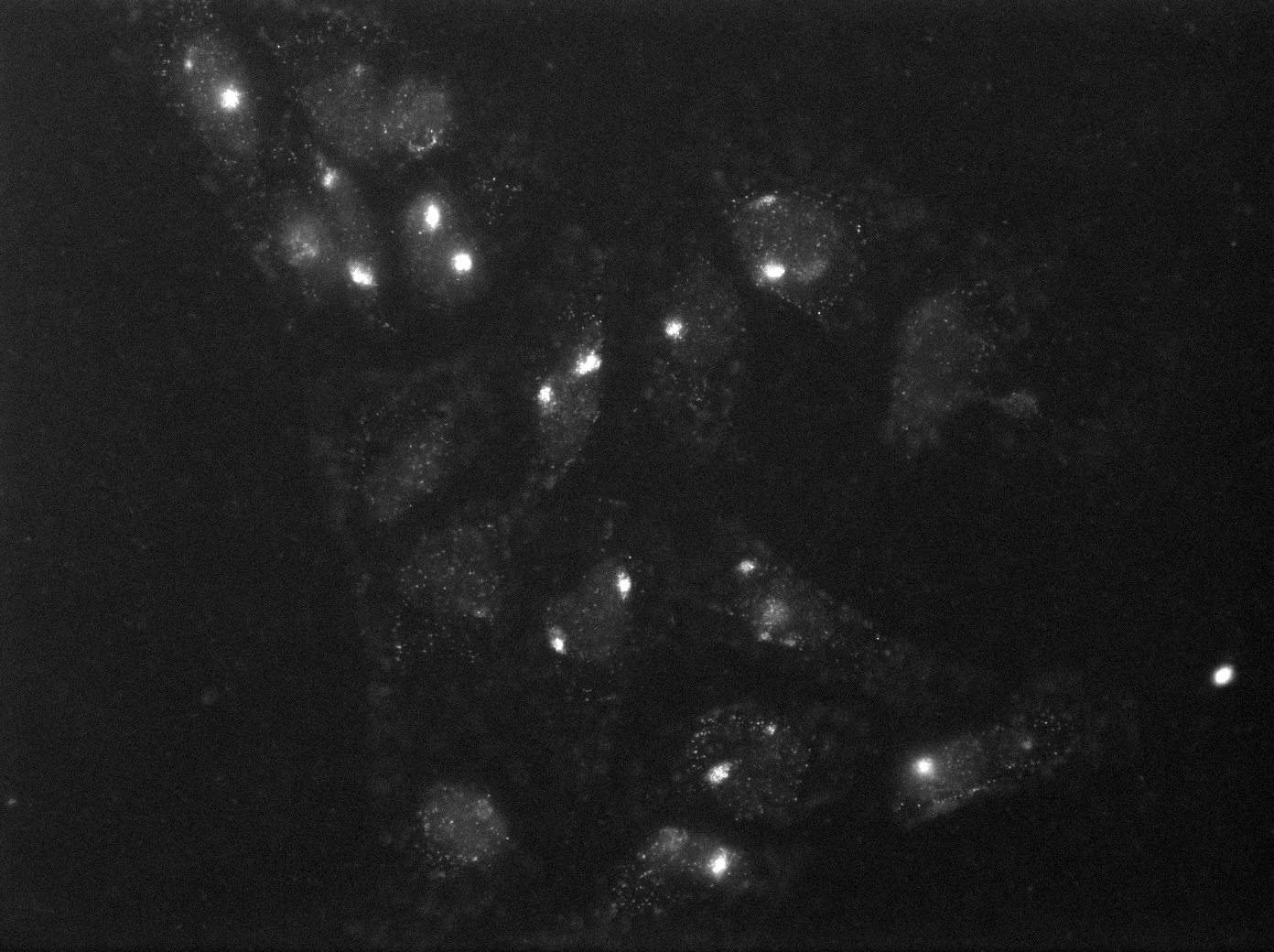

Supplement: Supplementary file 8 — Source Data [file 41467_2021_24153_MOESM8_ESM.zip › RawData/Main Figures/Fig7/a/U3S_QCUVP_siLUC_09_w2GFP.TIF]

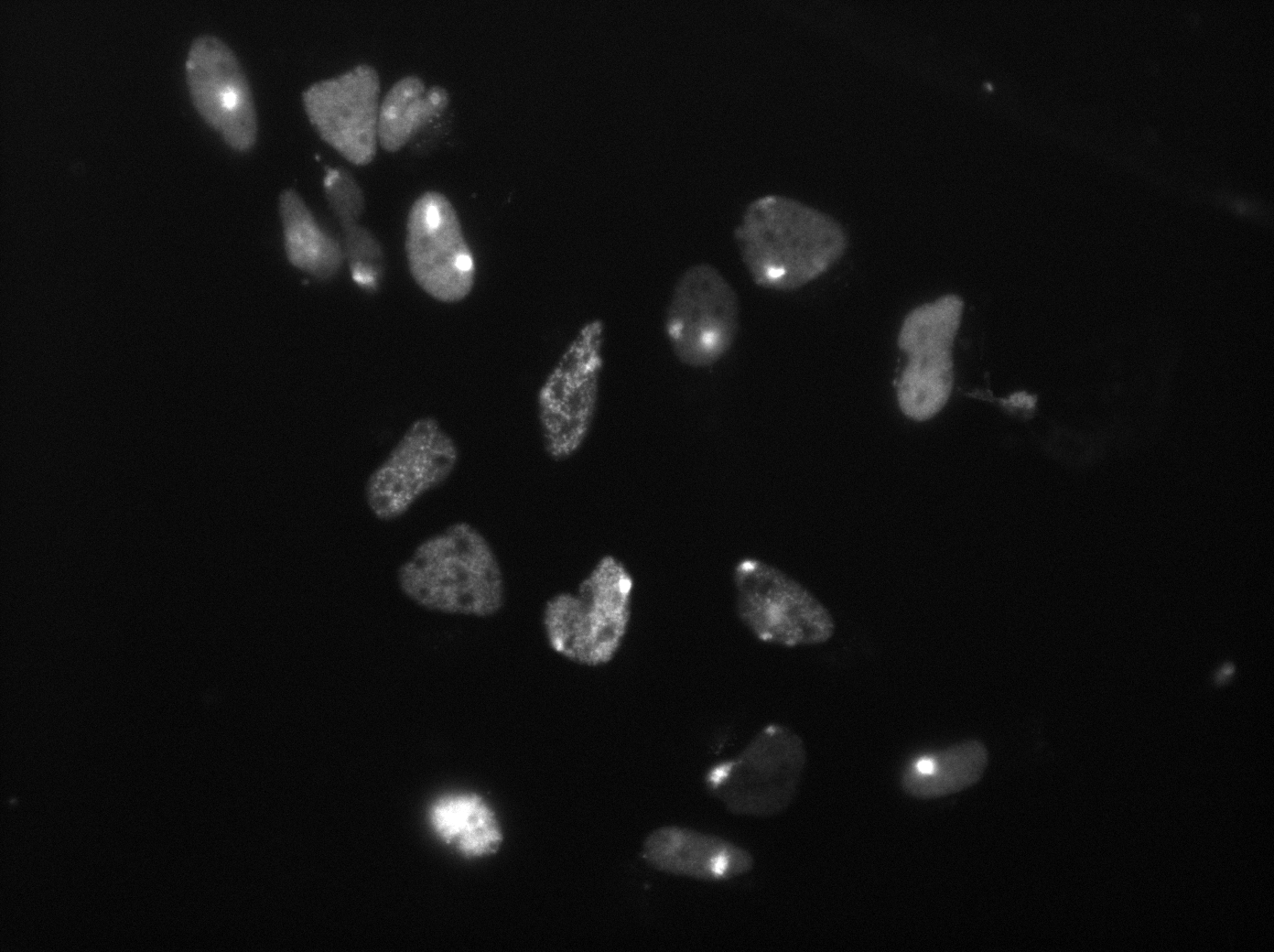

Supplement: Supplementary file 8 — Source Data [file 41467_2021_24153_MOESM8_ESM.zip › RawData/Main Figures/Fig7/a/U3S_QCUVP_siLUC_09_w3CY3.TIF]

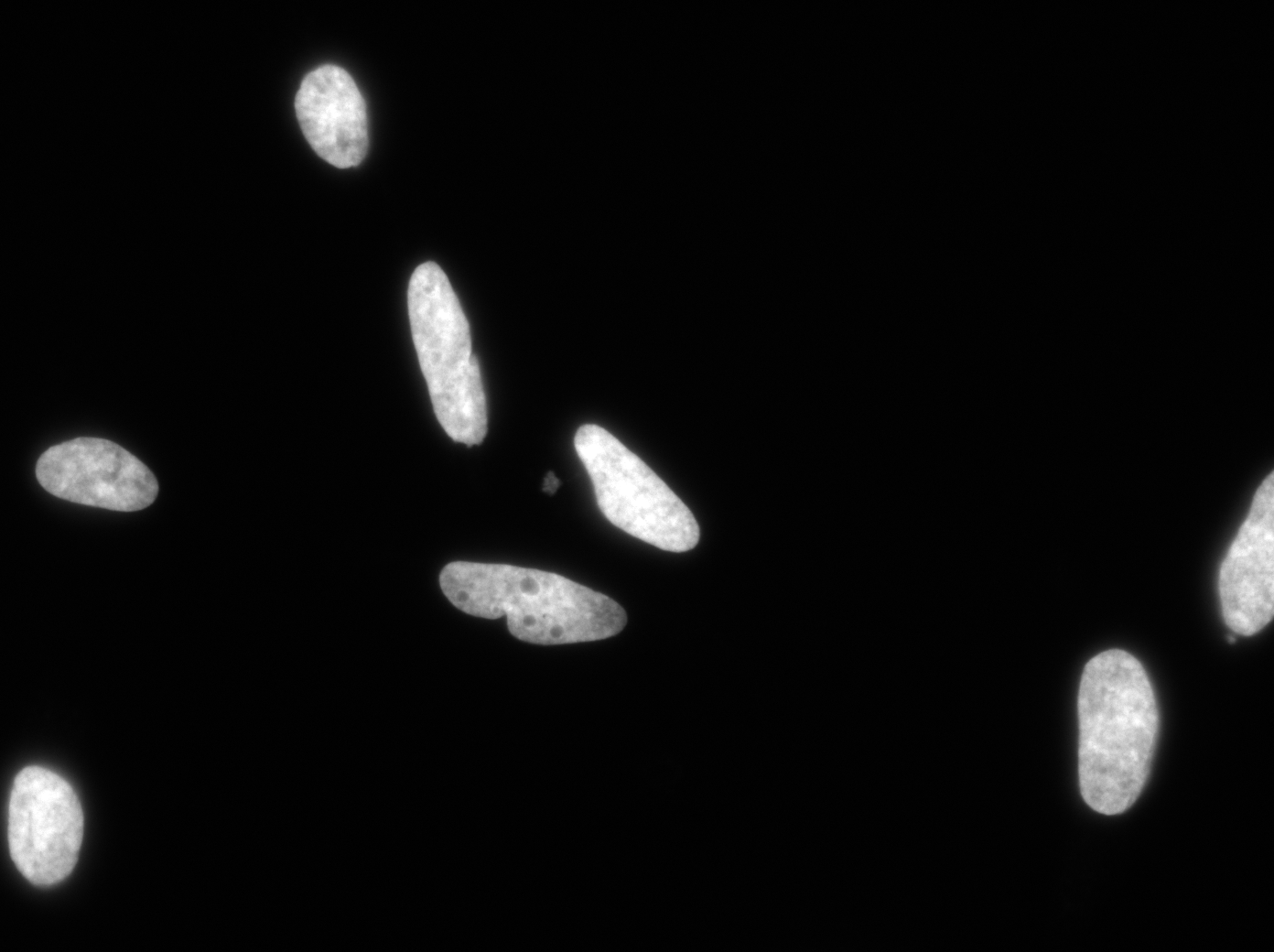

Supplement: Supplementary file 8 — Source Data [file 41467_2021_24153_MOESM8_ESM.zip › RawData/Main Figures/Fig7/a/U3S_QCUVP_siUBN2_1_01_w1DAPI.TIF]

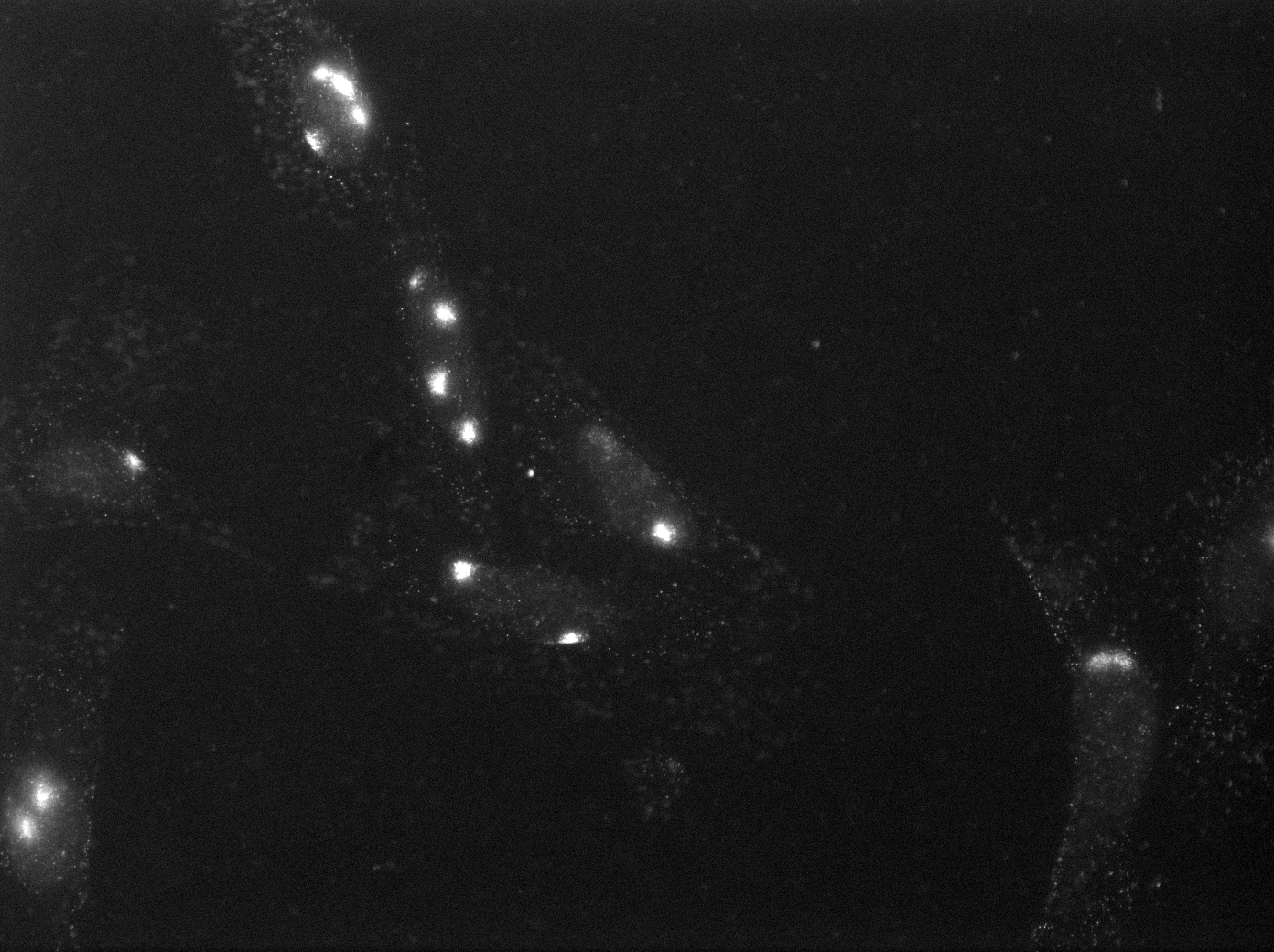

Supplement: Supplementary file 8 — Source Data [file 41467_2021_24153_MOESM8_ESM.zip › RawData/Main Figures/Fig7/a/U3S_QCUVP_siUBN2_1_01_w2GFP.TIF]

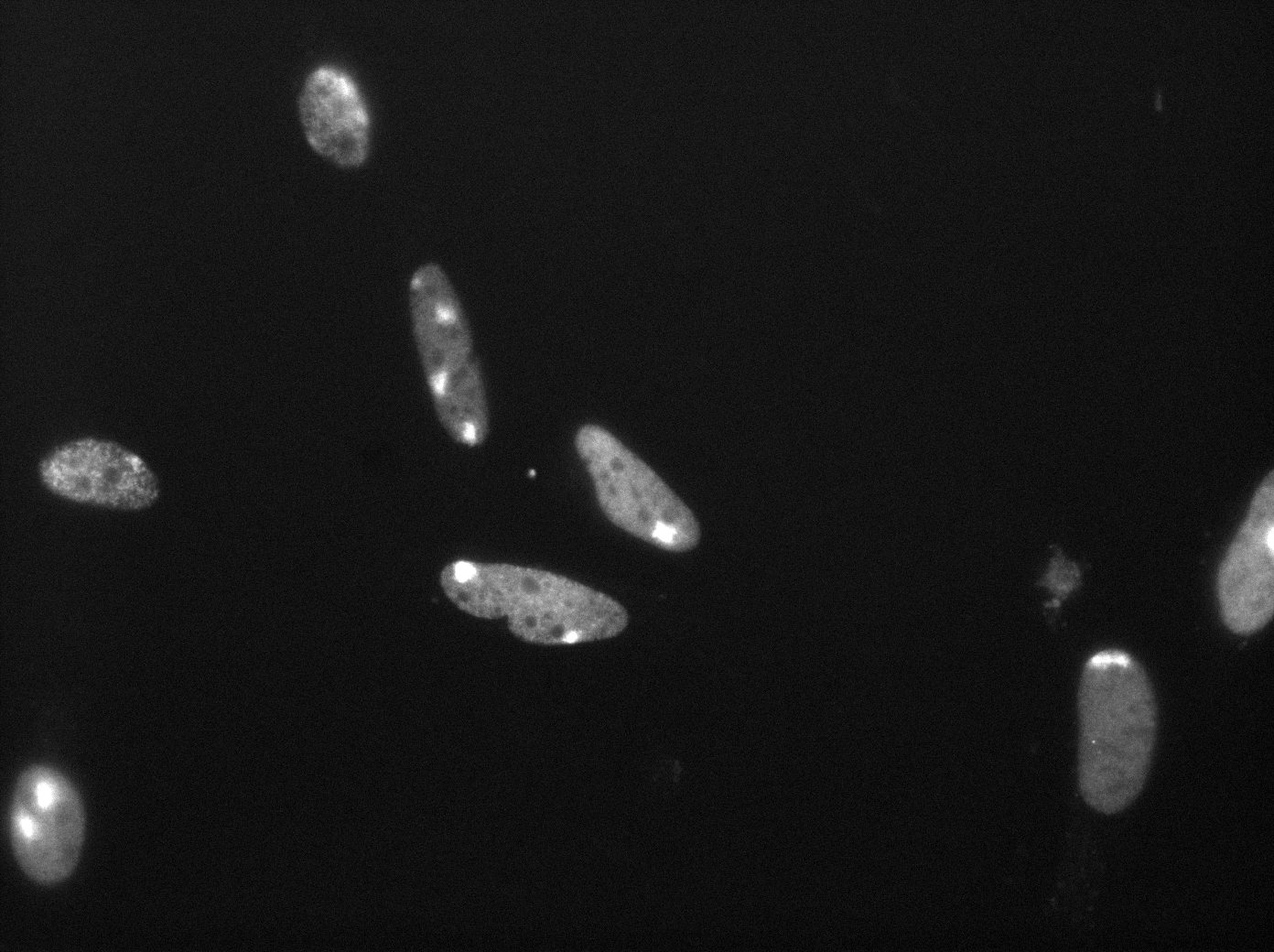

Supplement: Supplementary file 8 — Source Data [file 41467_2021_24153_MOESM8_ESM.zip › RawData/Main Figures/Fig7/a/U3S_QCUVP_siUBN2_1_01_w3CY3.TIF]

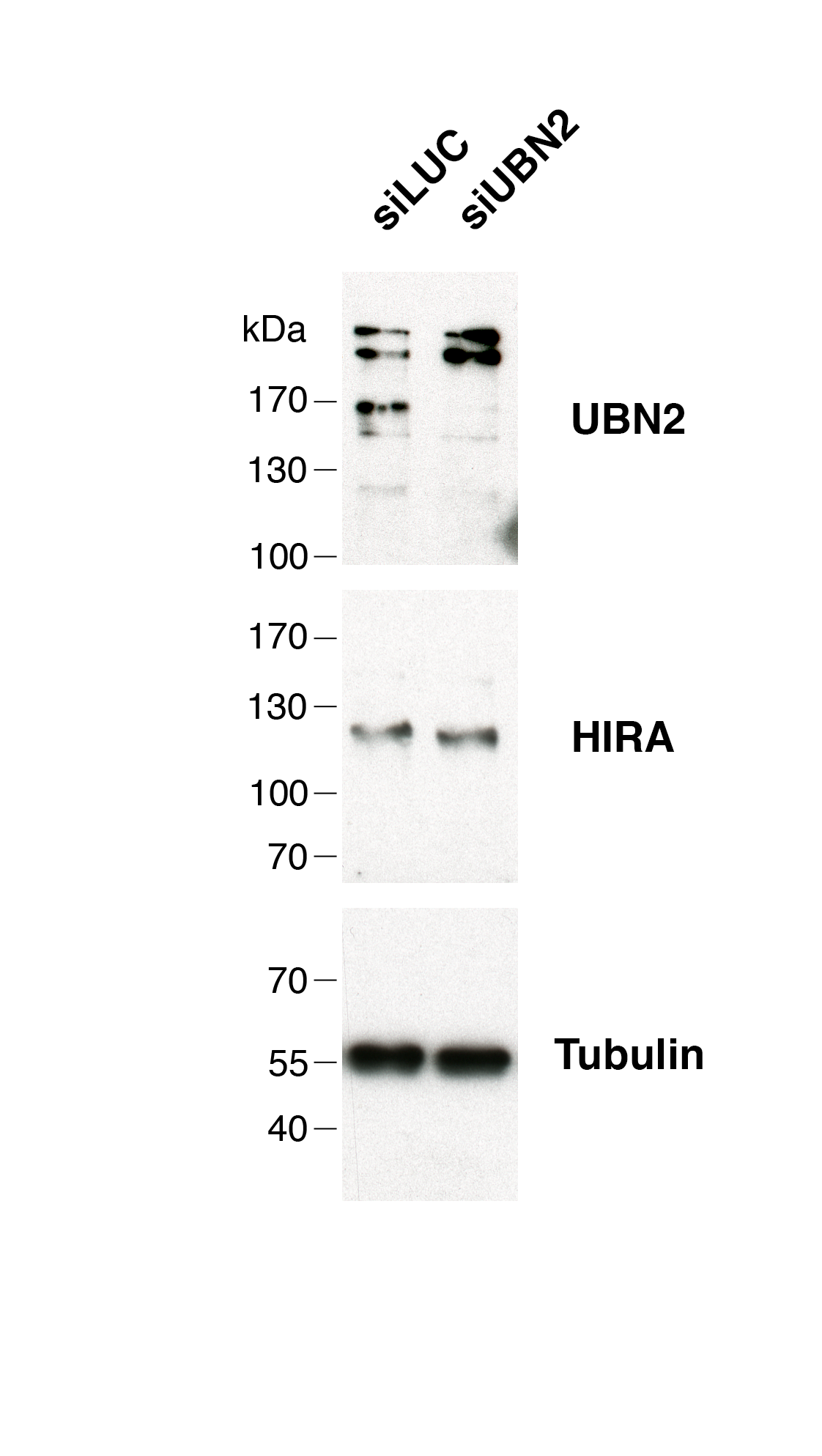

Supplement: Supplementary file 8 — Source Data [file 41467_2021_24153_MOESM8_ESM.zip › RawData/Main Figures/Fig7/a/WB.tif]

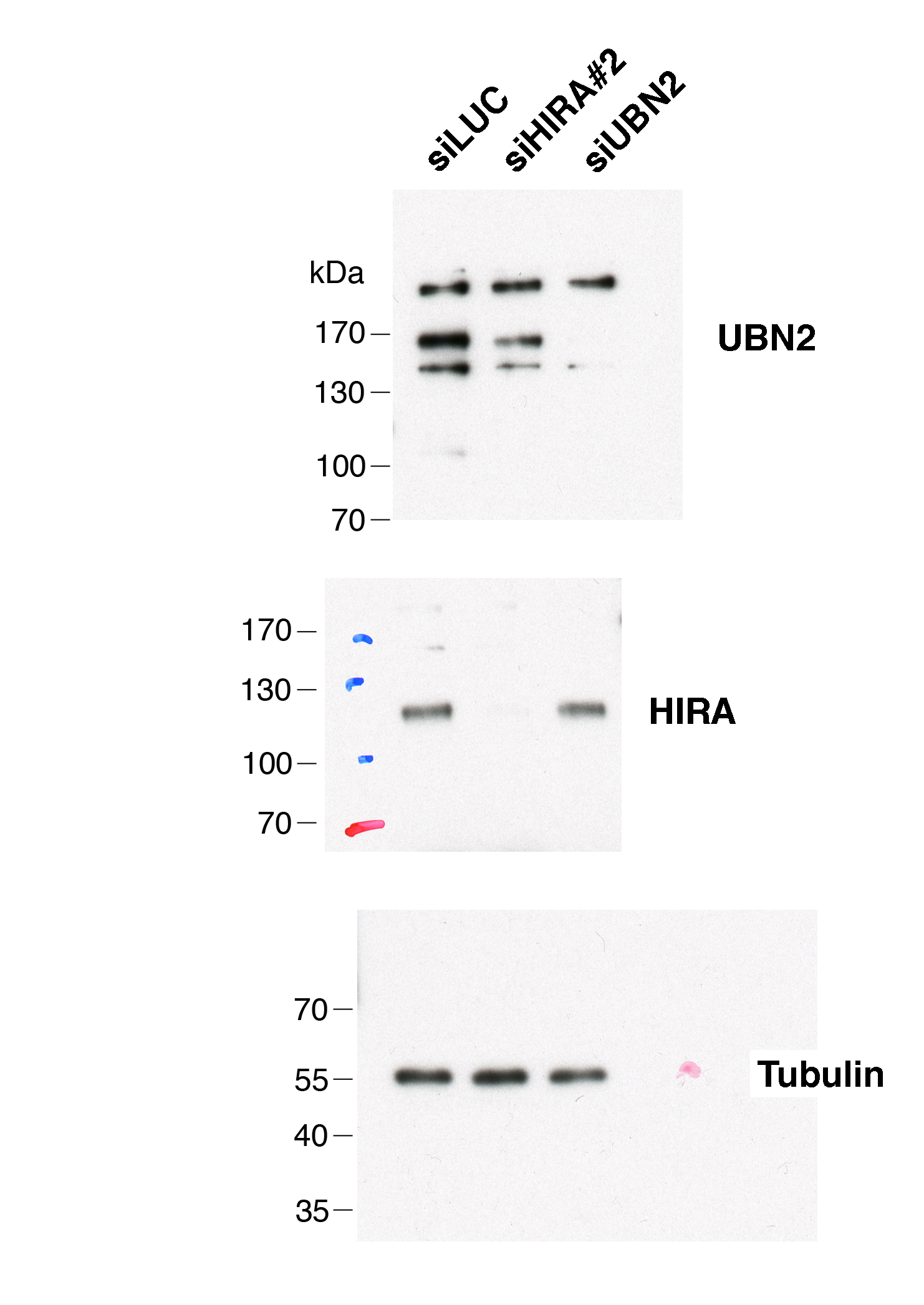

Supplement: Supplementary file 8 — Source Data [file 41467_2021_24153_MOESM8_ESM.zip › RawData/Main Figures/Fig7/b/WB.tif]

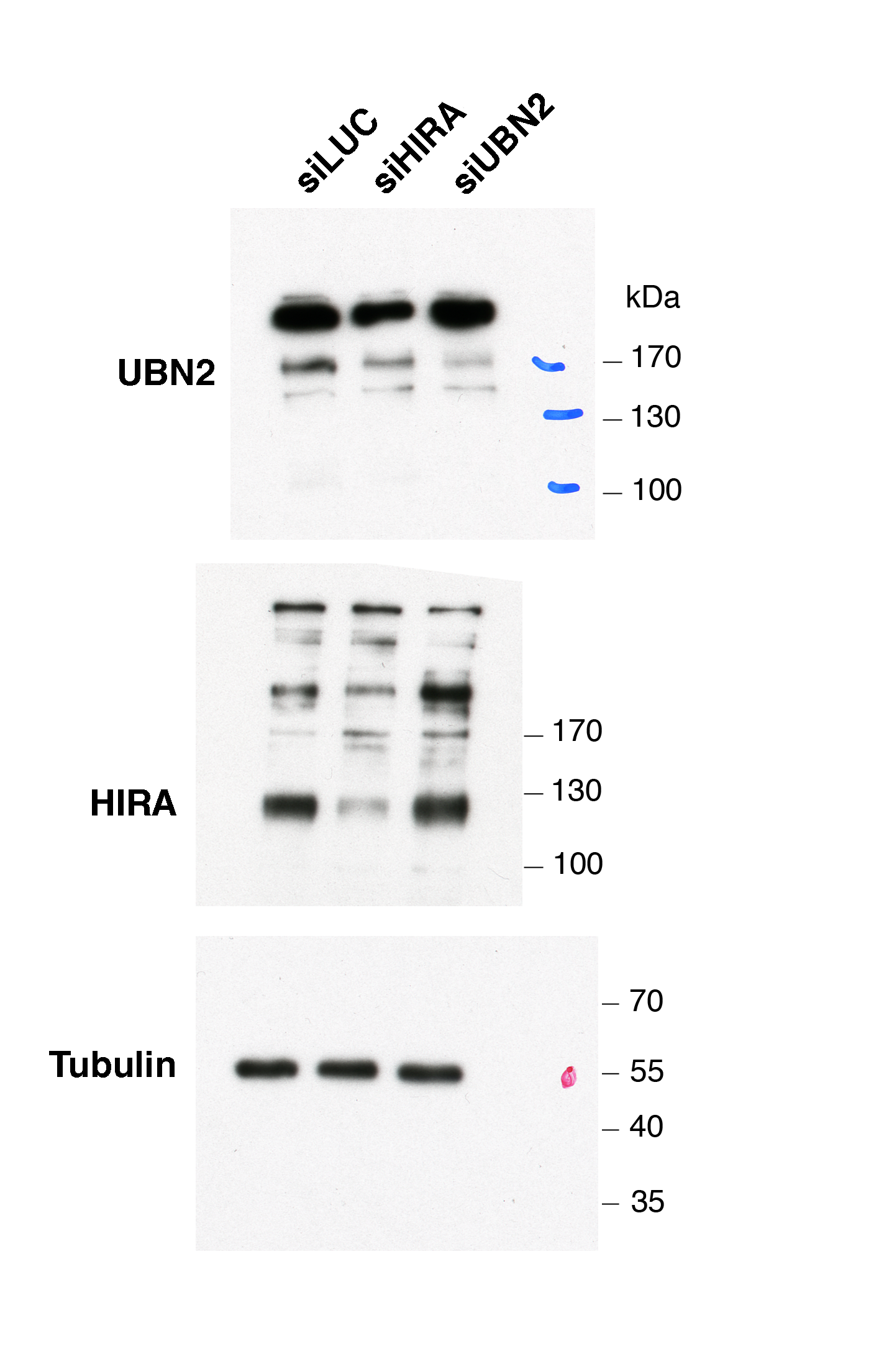

Supplement: Supplementary file 8 — Source Data [file 41467_2021_24153_MOESM8_ESM.zip › RawData/Main Figures/Fig7/c/WB.tif]

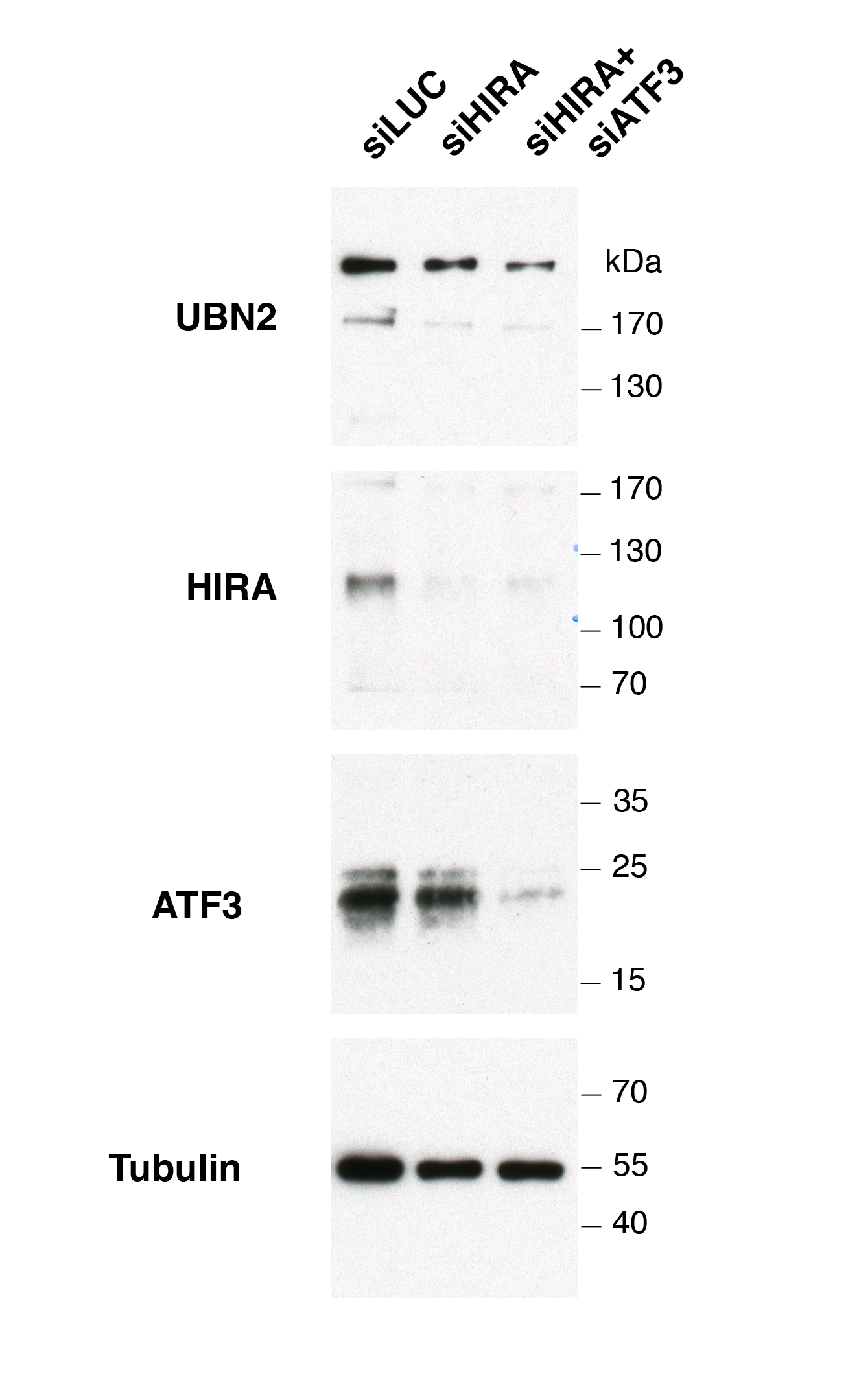

Supplement: Supplementary file 8 — Source Data [file 41467_2021_24153_MOESM8_ESM.zip › RawData/Main Figures/Fig7/d/WB.tif]

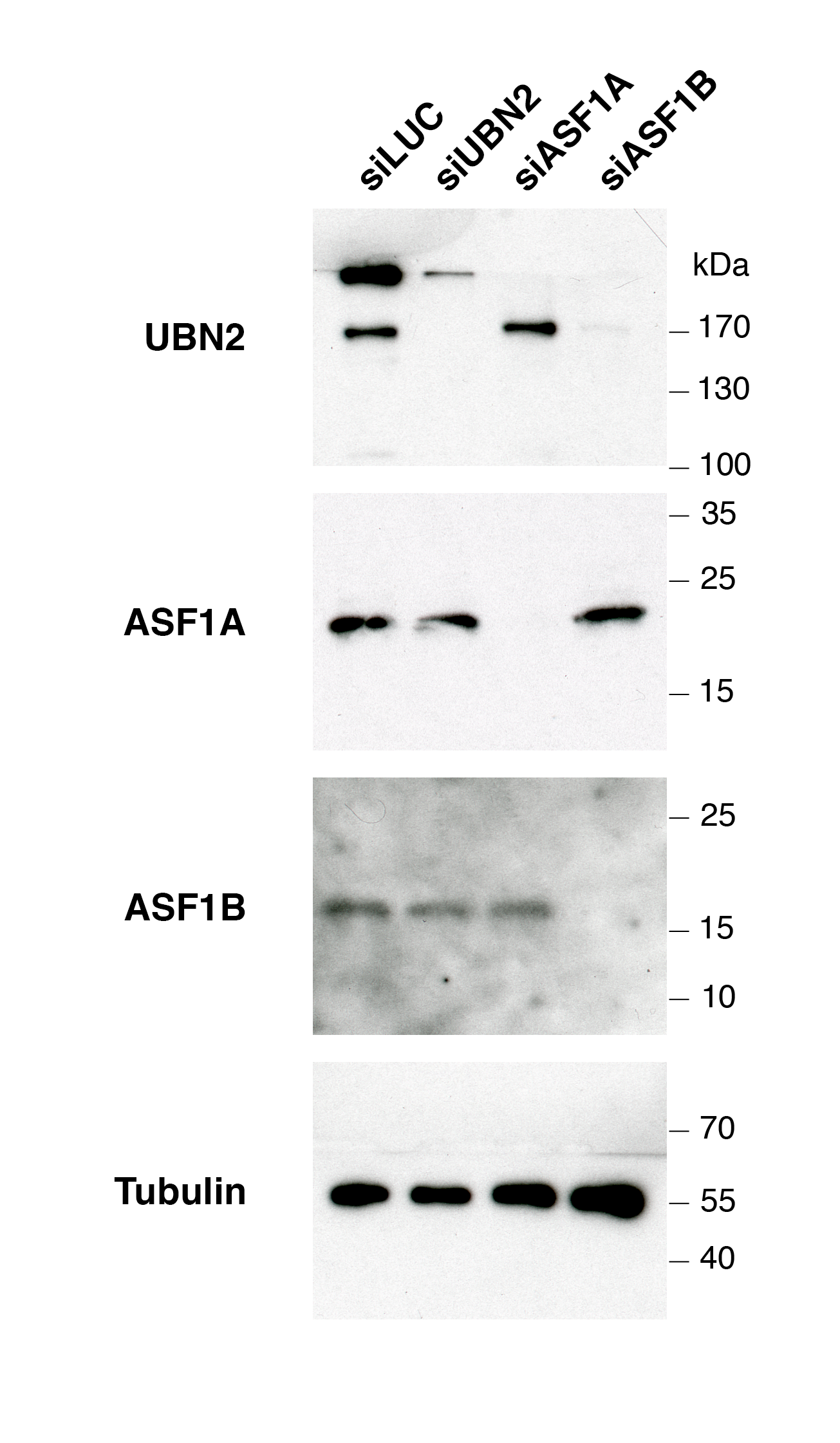

Supplement: Supplementary file 8 — Source Data [file 41467_2021_24153_MOESM8_ESM.zip › RawData/Main Figures/Fig7/e/WB.tif]

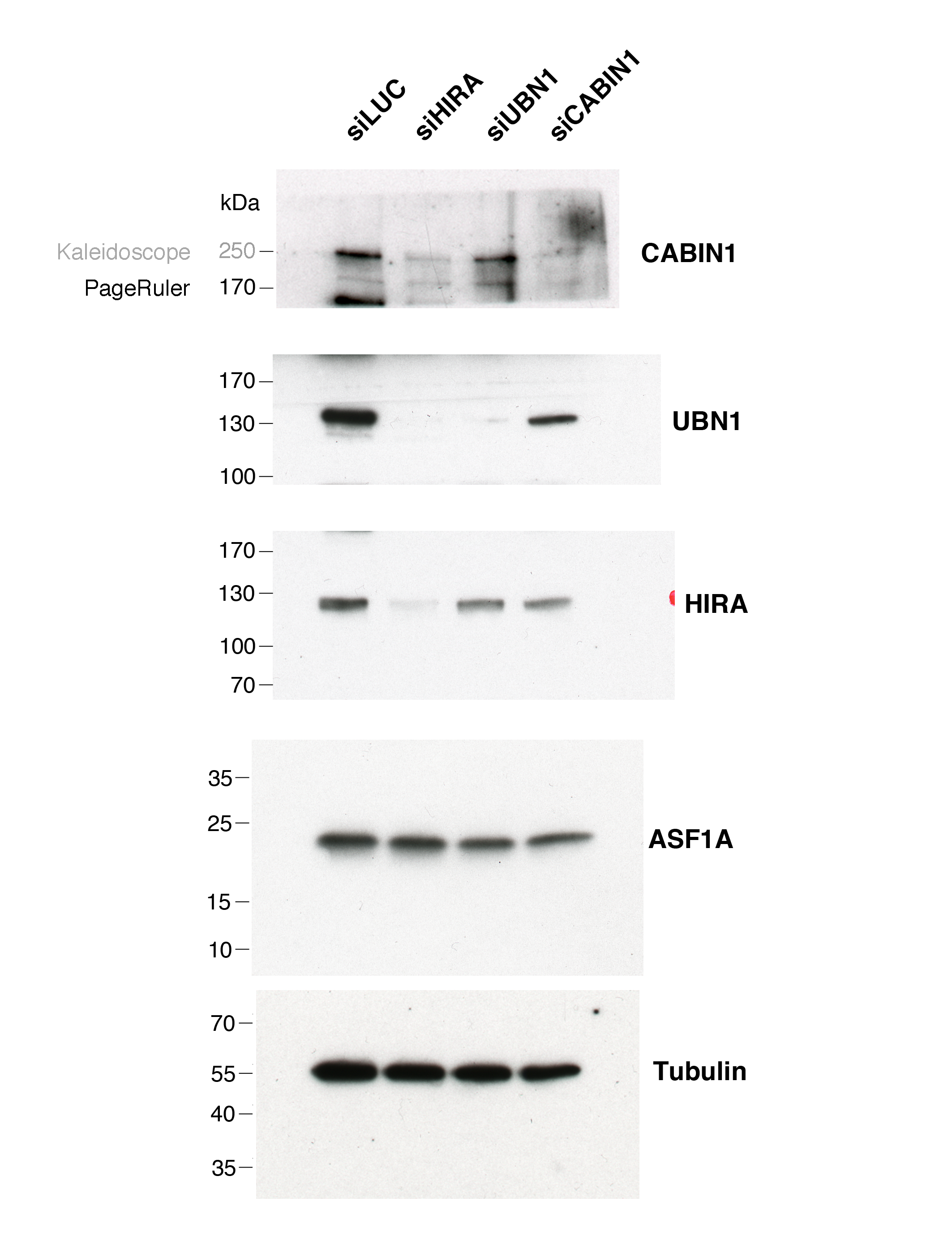

Supplement: Supplementary file 8 — Source Data [file 41467_2021_24153_MOESM8_ESM.zip › RawData/Supplementary Figures/FigS1/c/WB_24hpostUV.tif]

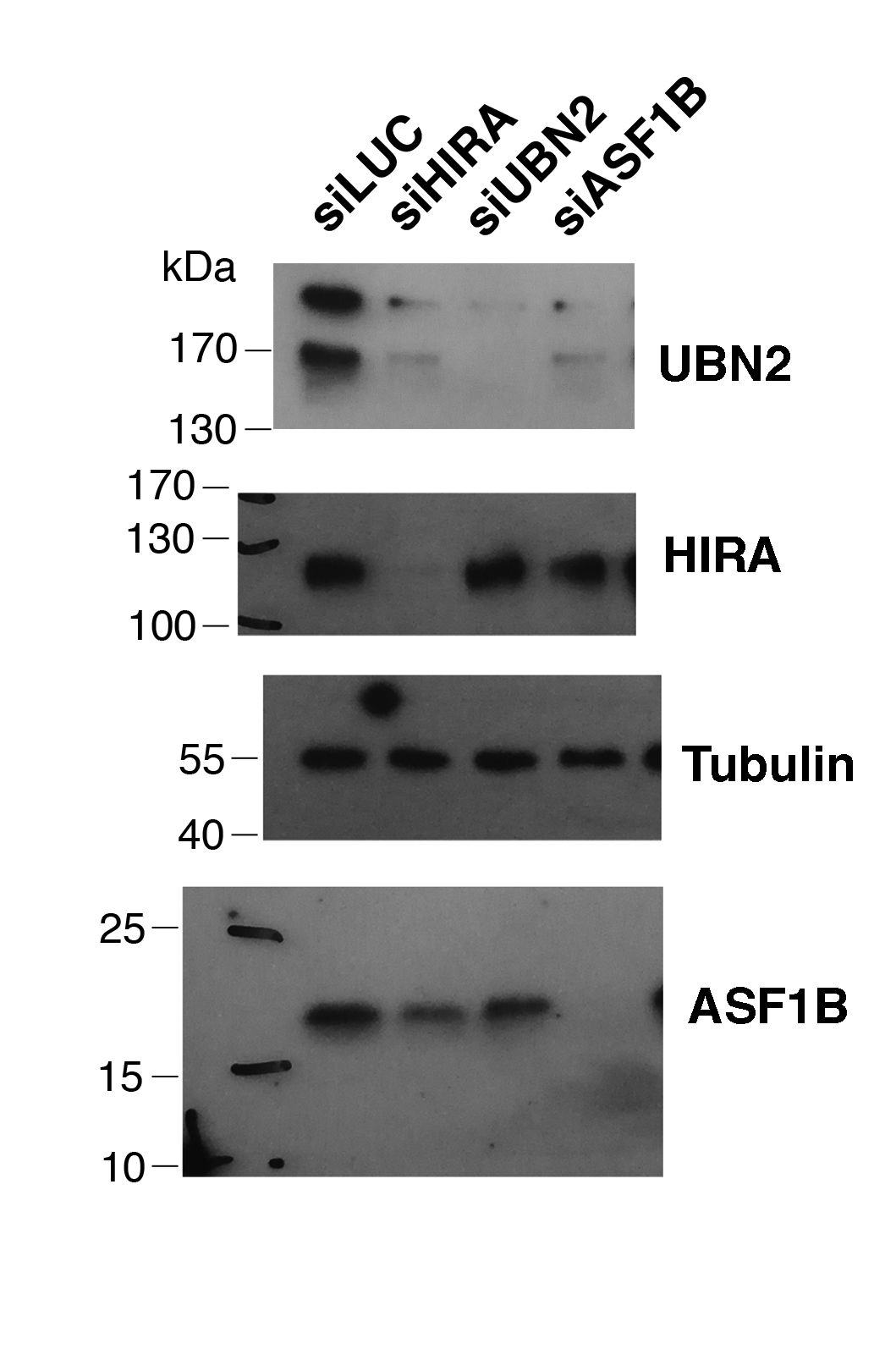

Supplement: Supplementary file 8 — Source Data [file 41467_2021_24153_MOESM8_ESM.zip › RawData/Supplementary Figures/FigS1/d/WB.tif]

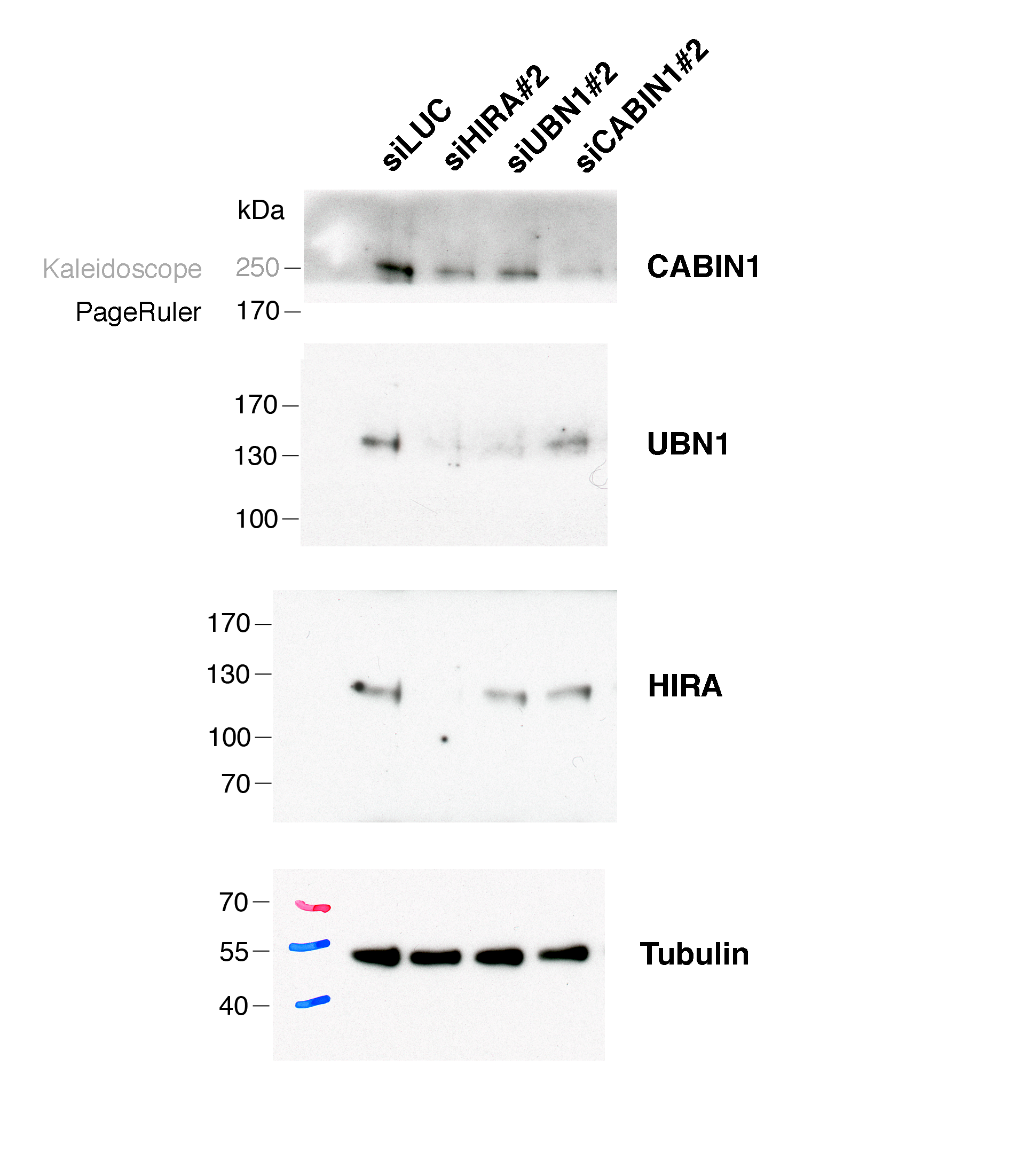

Supplement: Supplementary file 8 — Source Data [file 41467_2021_24153_MOESM8_ESM.zip › RawData/Supplementary Figures/FigS1/f/WB.tif]

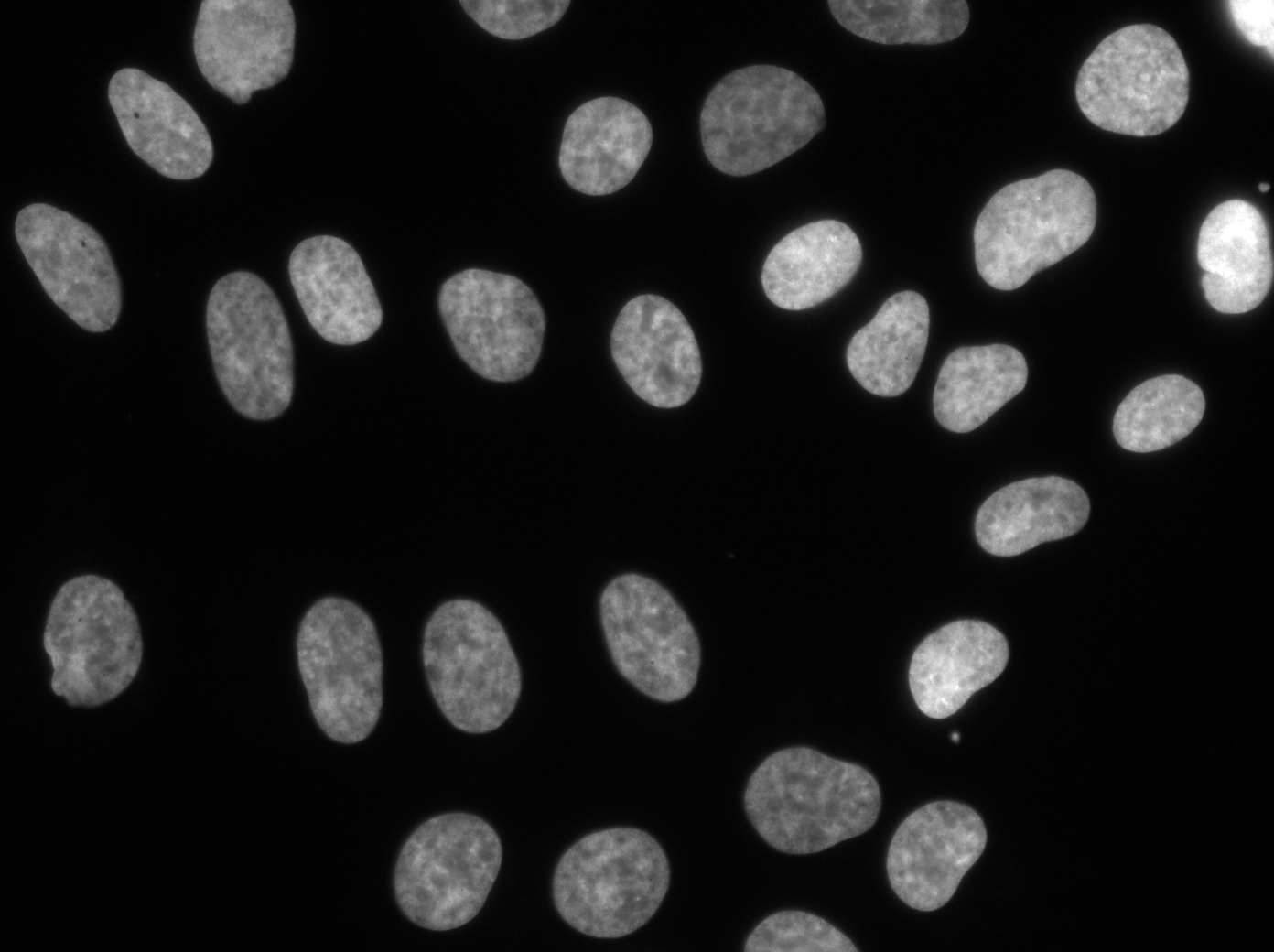

Supplement: Supplementary file 8 — Source Data [file 41467_2021_24153_MOESM8_ESM.zip › RawData/Supplementary Figures/FigS2/a/U2OS_500J5mnTx_siHIRA_05_w1DAPI.TIF]

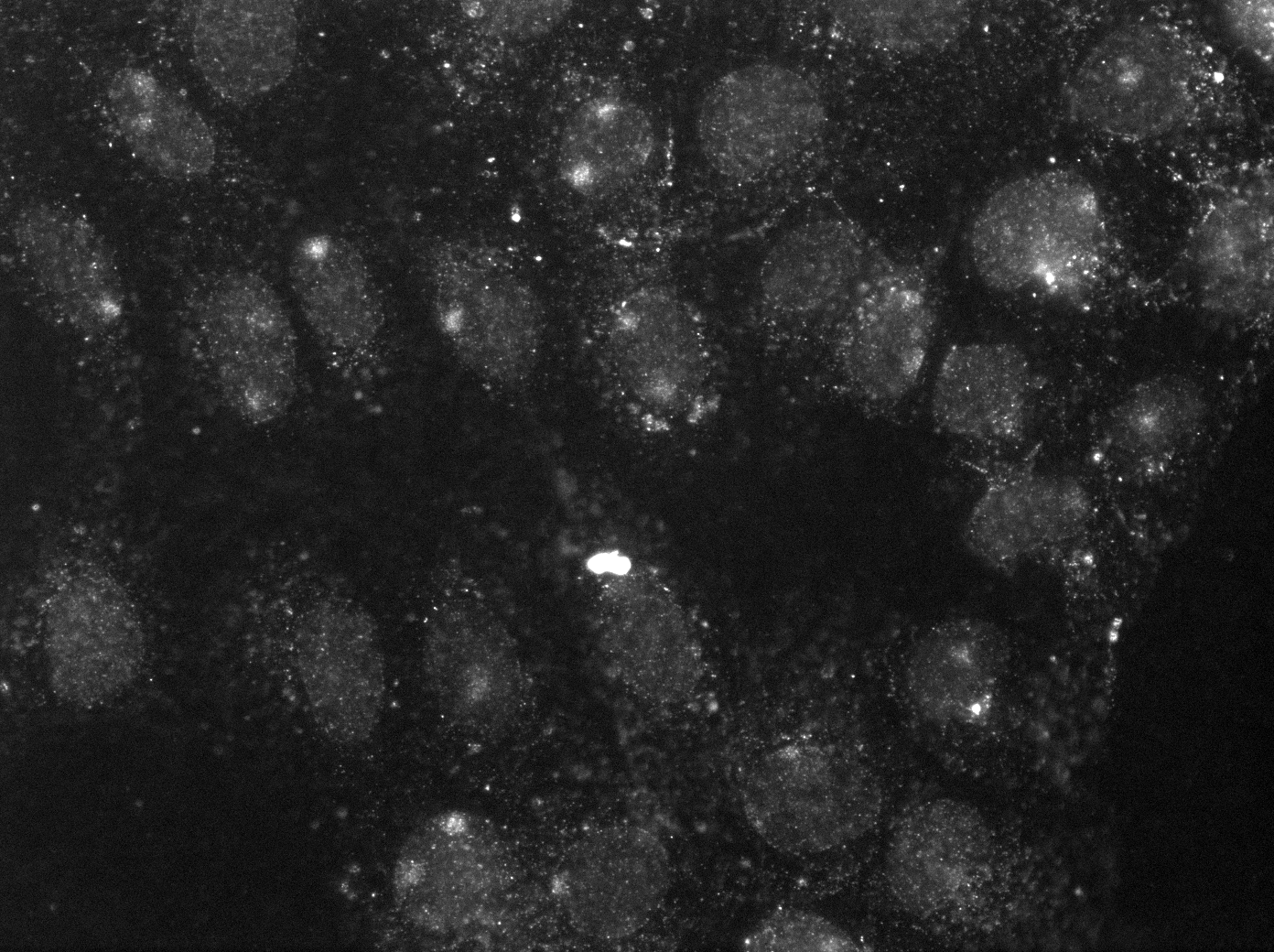

Supplement: Supplementary file 8 — Source Data [file 41467_2021_24153_MOESM8_ESM.zip › RawData/Supplementary Figures/FigS2/a/U2OS_500J5mnTx_siHIRA_05_w2GFP.TIF]

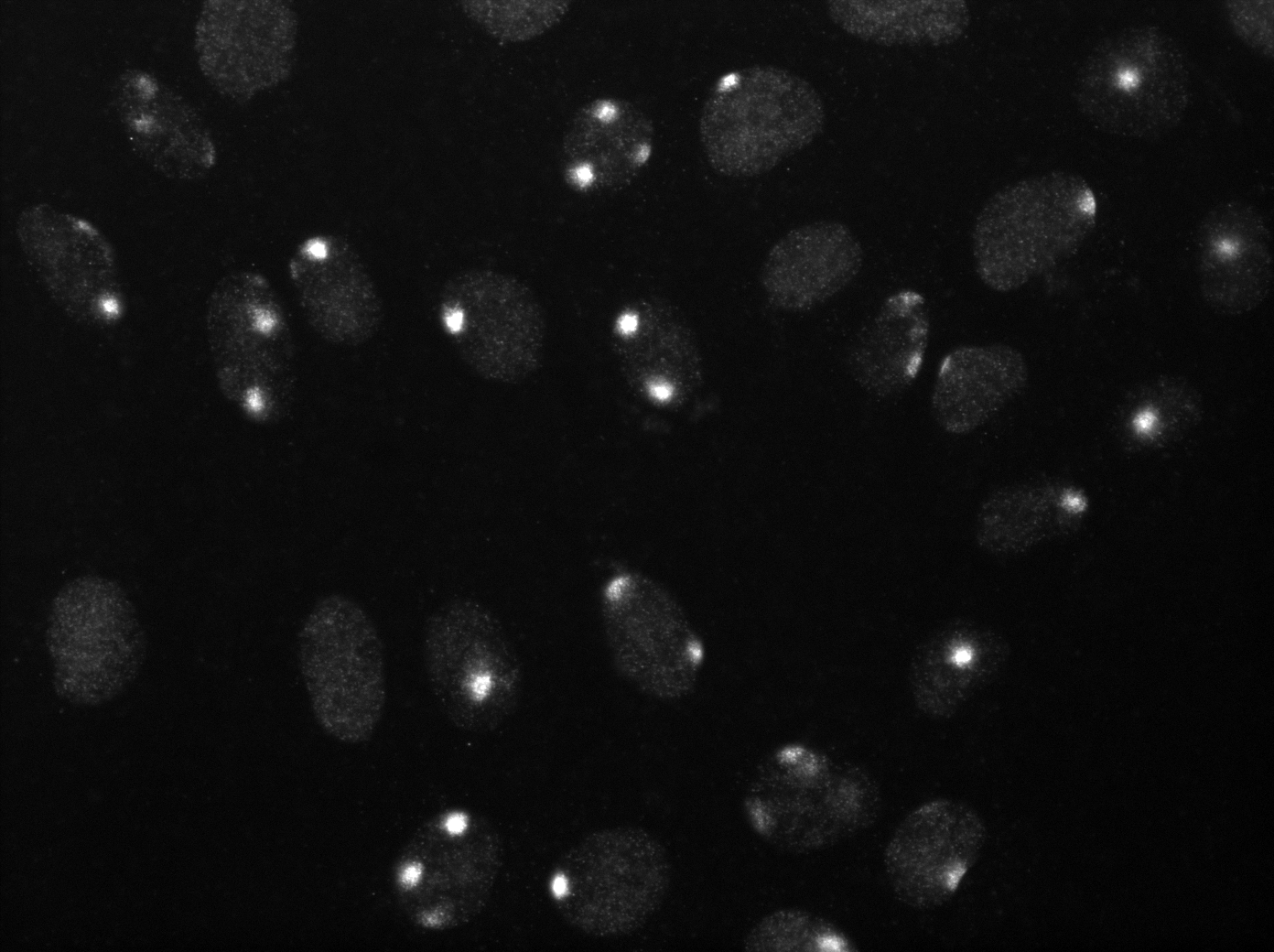

Supplement: Supplementary file 8 — Source Data [file 41467_2021_24153_MOESM8_ESM.zip › RawData/Supplementary Figures/FigS2/a/U2OS_500J5mnTx_siHIRA_05_w3TX.TIF]

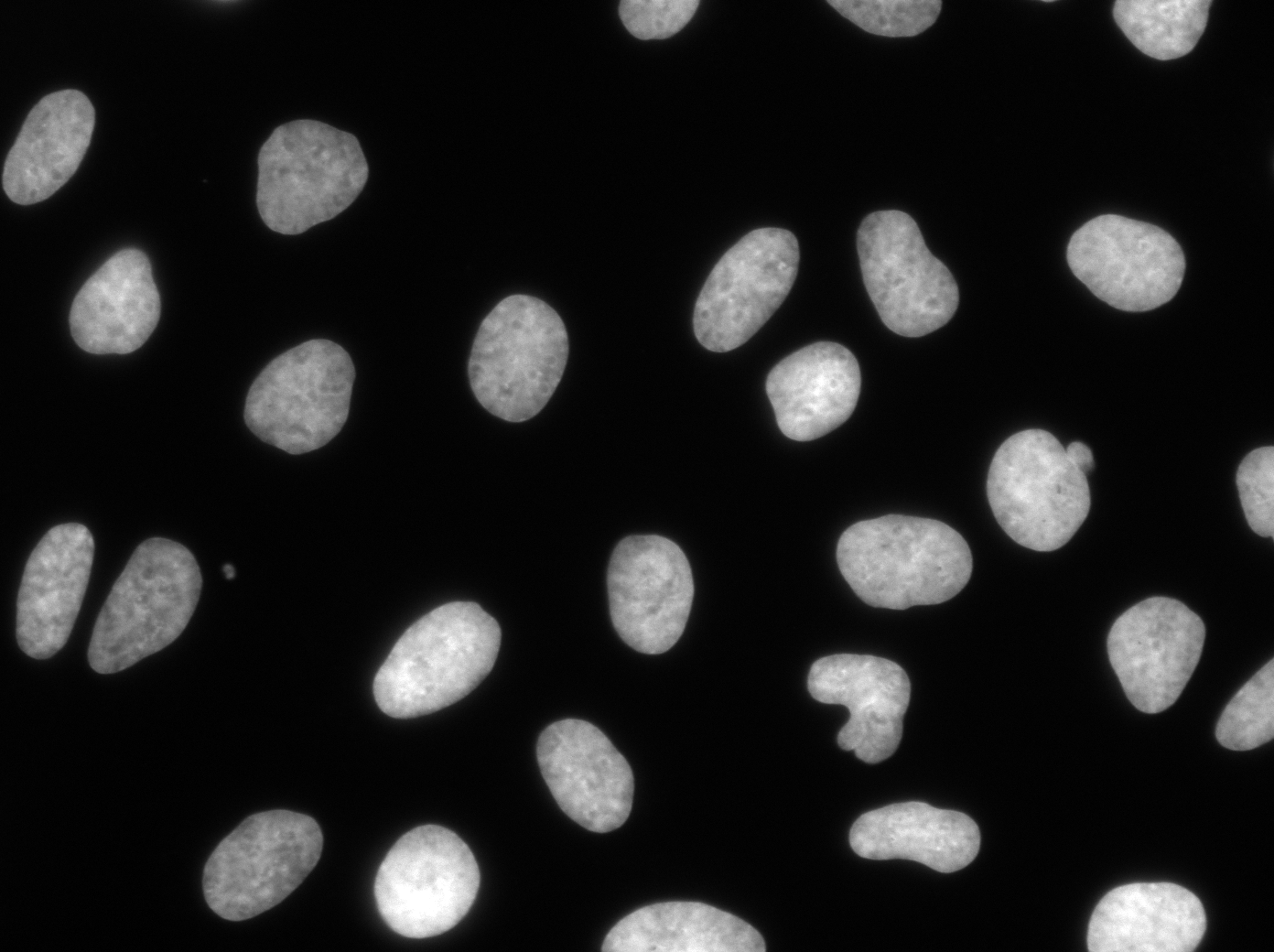

Supplement: Supplementary file 8 — Source Data [file 41467_2021_24153_MOESM8_ESM.zip › RawData/Supplementary Figures/FigS2/a/U2OS_500J5mnTx_siLUC_07_w1DAPI.TIF]

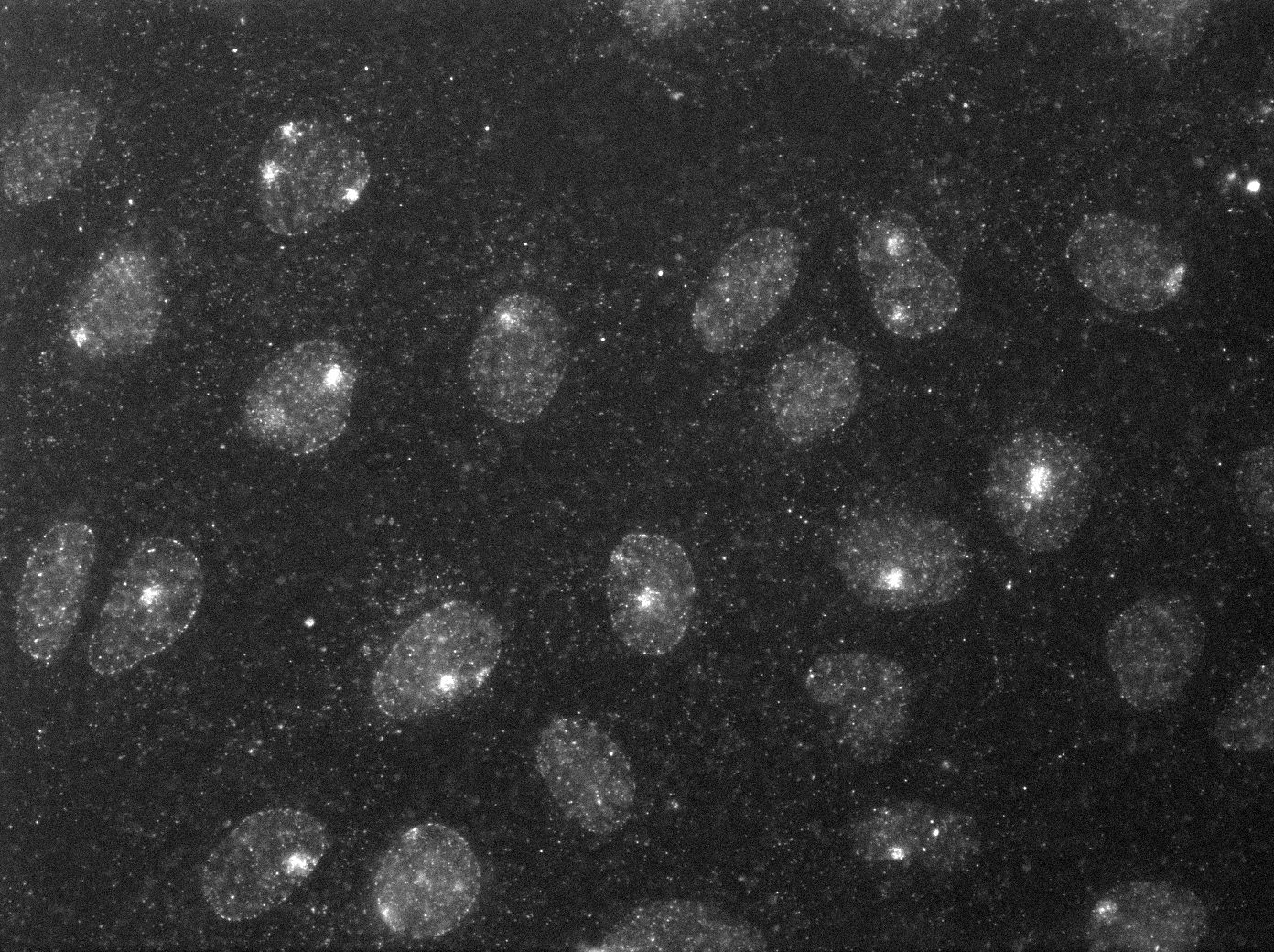

Supplement: Supplementary file 8 — Source Data [file 41467_2021_24153_MOESM8_ESM.zip › RawData/Supplementary Figures/FigS2/a/U2OS_500J5mnTx_siLUC_07_w2GFP.TIF]

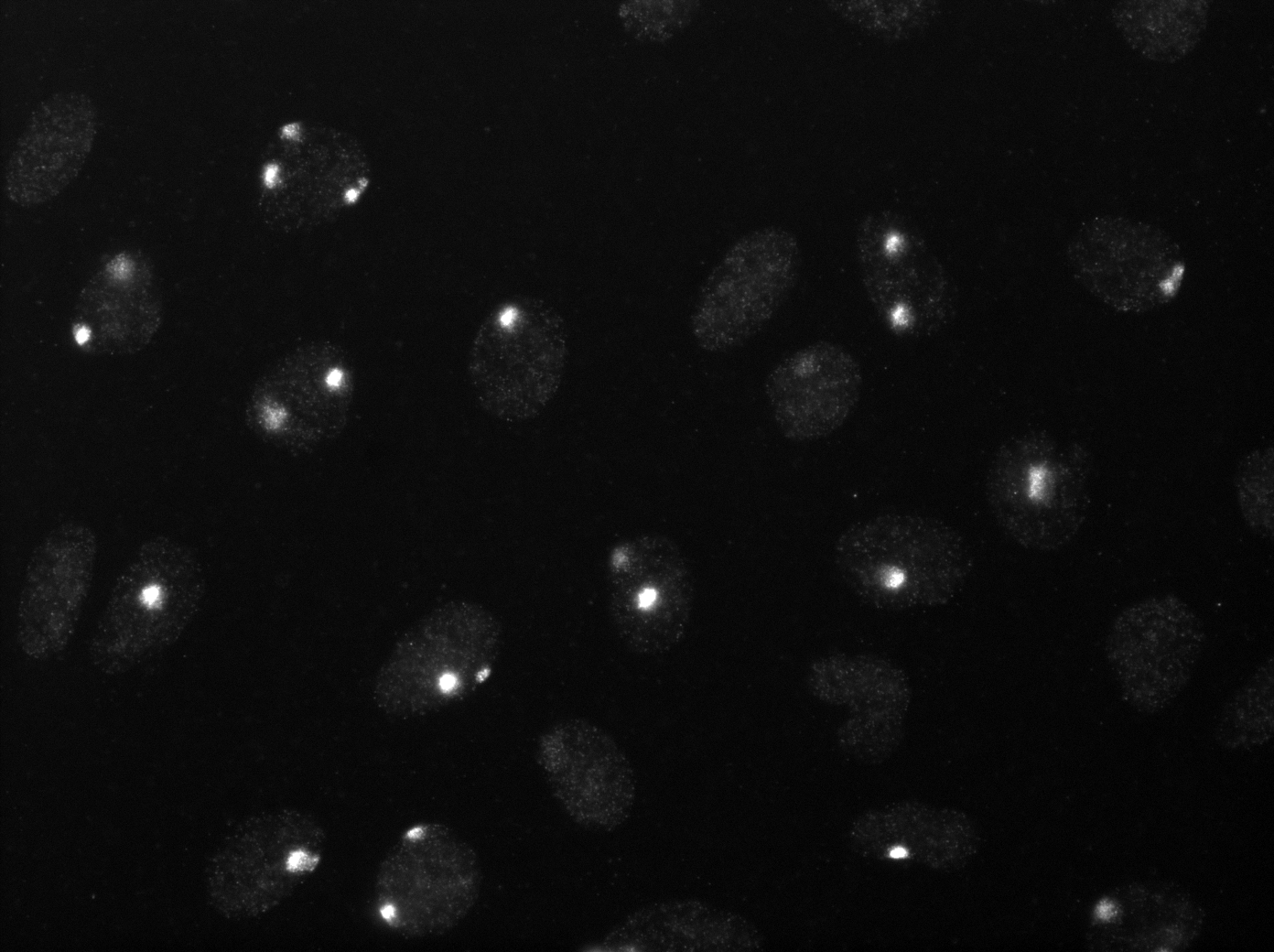

Supplement: Supplementary file 8 — Source Data [file 41467_2021_24153_MOESM8_ESM.zip › RawData/Supplementary Figures/FigS2/a/U2OS_500J5mnTx_siLUC_07_w3TX.TIF]

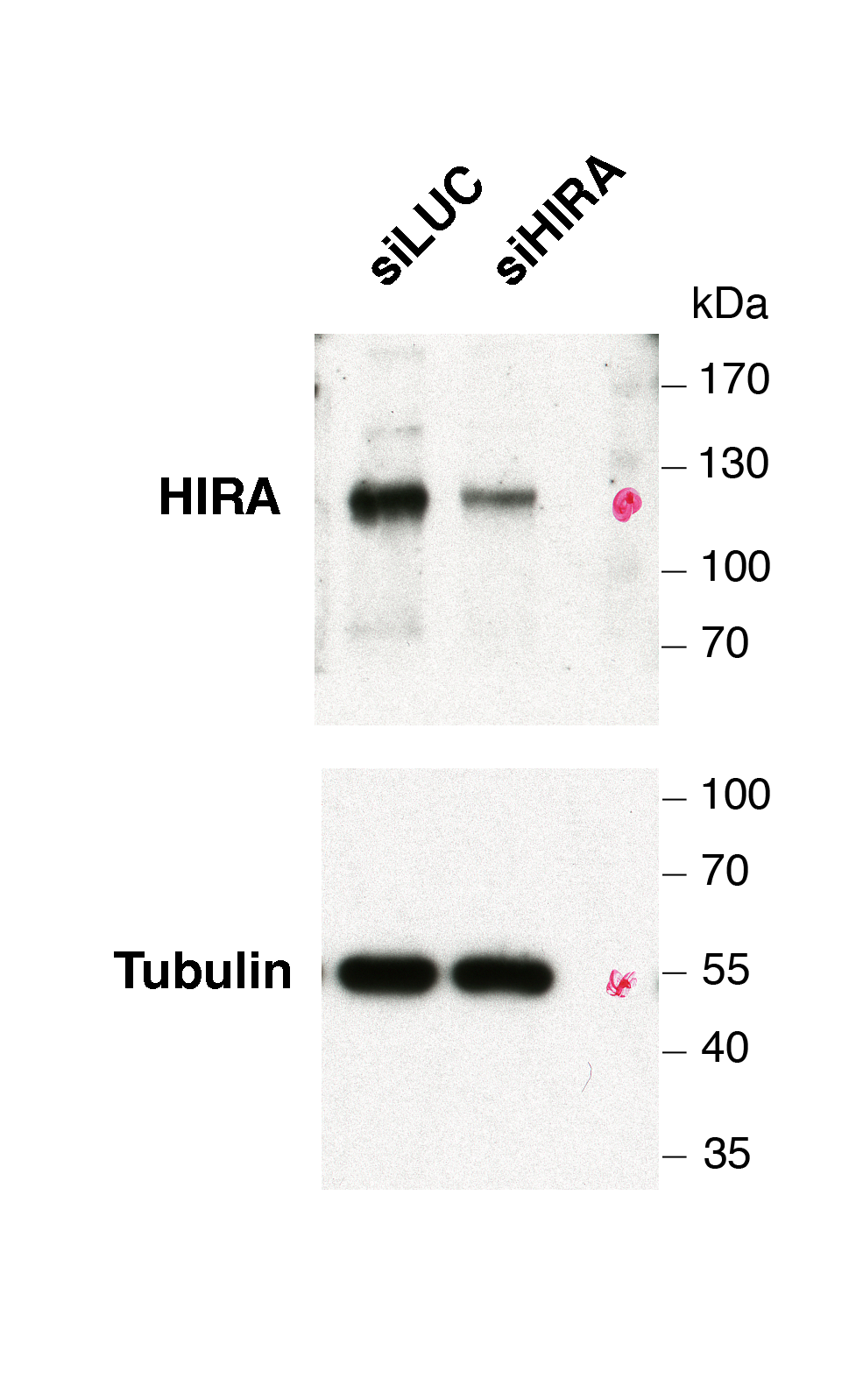

Supplement: Supplementary file 8 — Source Data [file 41467_2021_24153_MOESM8_ESM.zip › RawData/Supplementary Figures/FigS2/a/WB.tif]

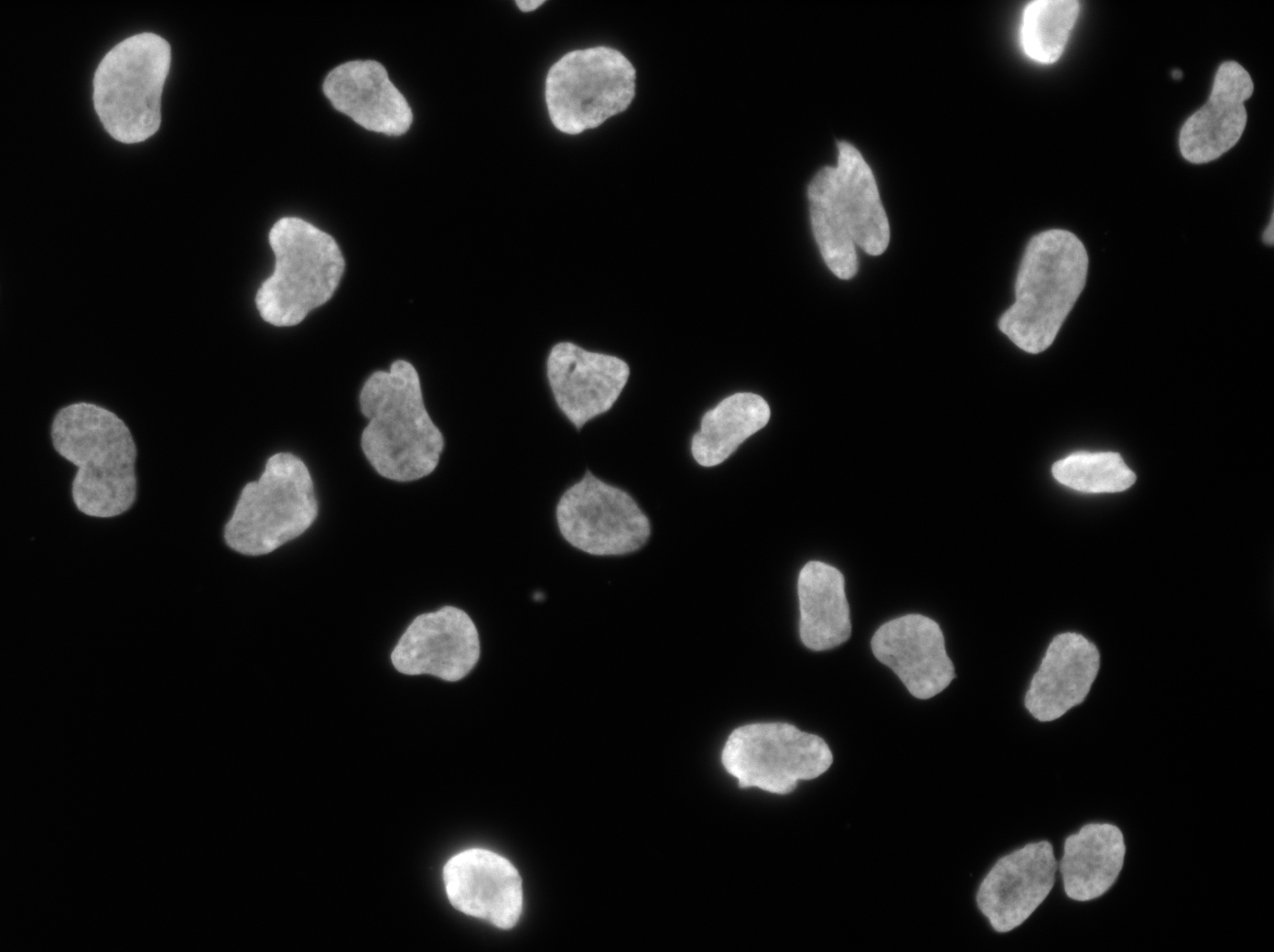

Supplement: Supplementary file 8 — Source Data [file 41467_2021_24153_MOESM8_ESM.zip › RawData/Supplementary Figures/FigS2/b/U3S_48h_siHIRA_03_w1DAPI.TIF]

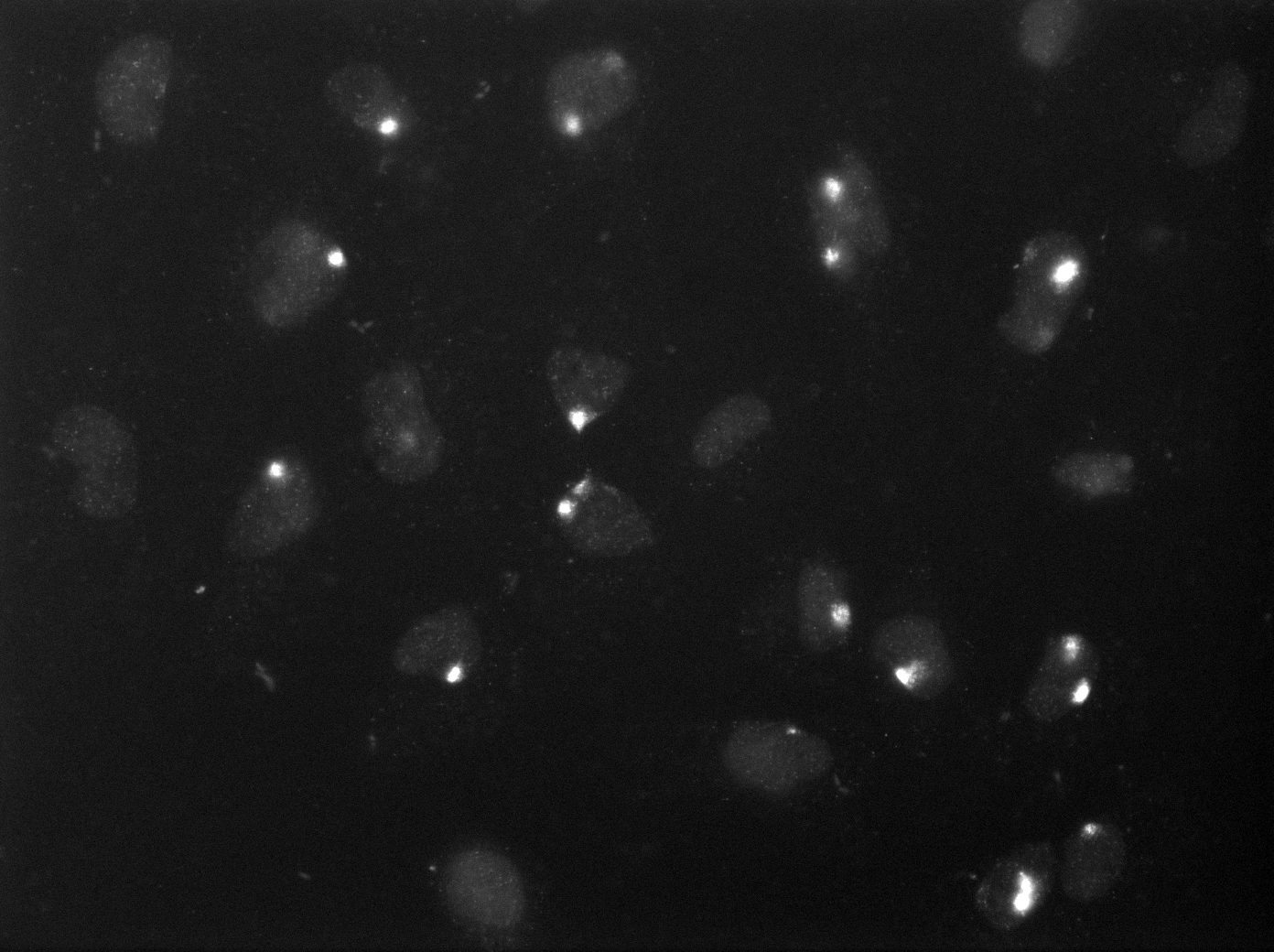

Supplement: Supplementary file 8 — Source Data [file 41467_2021_24153_MOESM8_ESM.zip › RawData/Supplementary Figures/FigS2/b/U3S_48h_siHIRA_03_w2GFP.TIF]

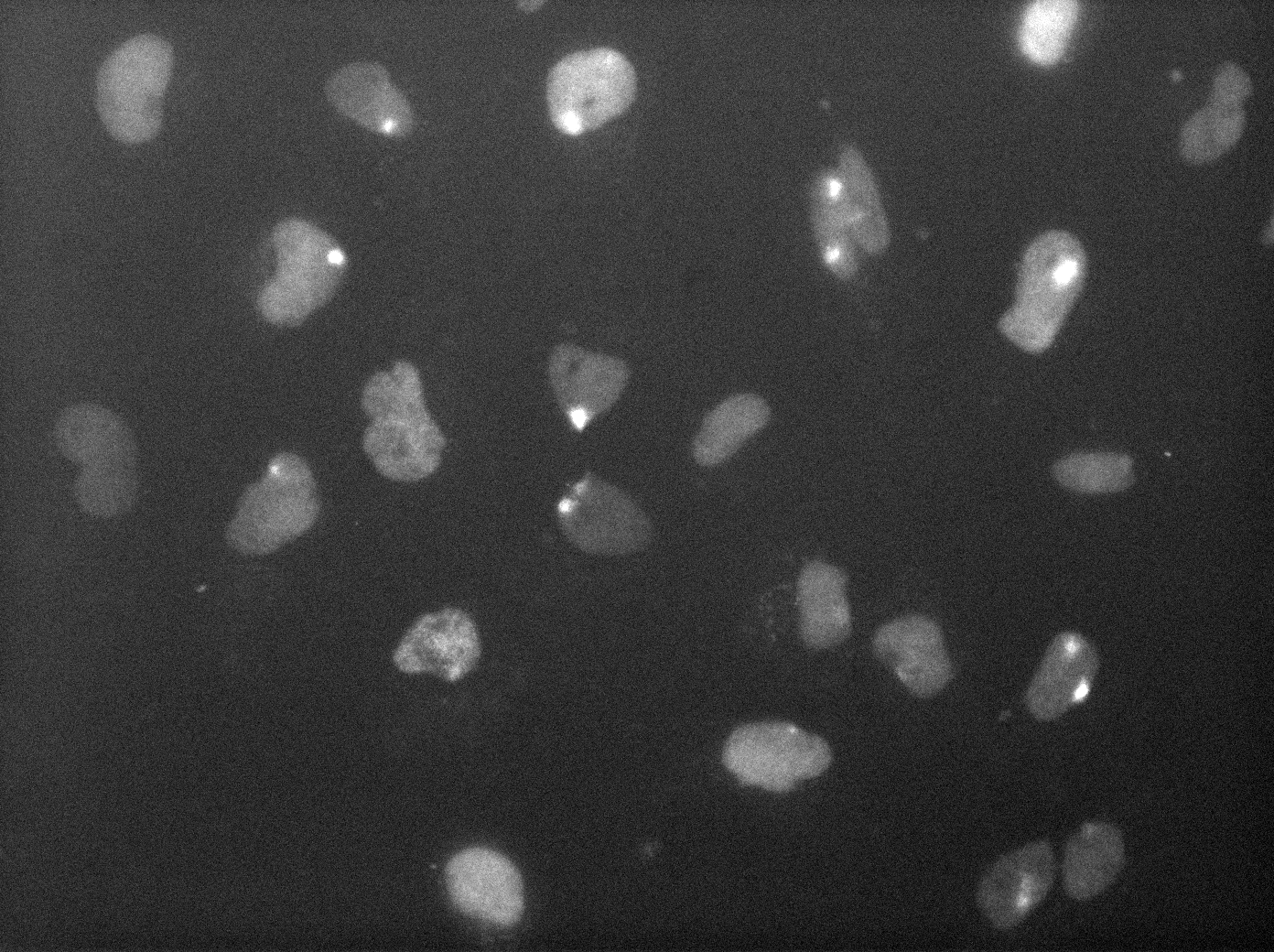

Supplement: Supplementary file 8 — Source Data [file 41467_2021_24153_MOESM8_ESM.zip › RawData/Supplementary Figures/FigS2/b/U3S_48h_siHIRA_03_w3CY3.TIF]

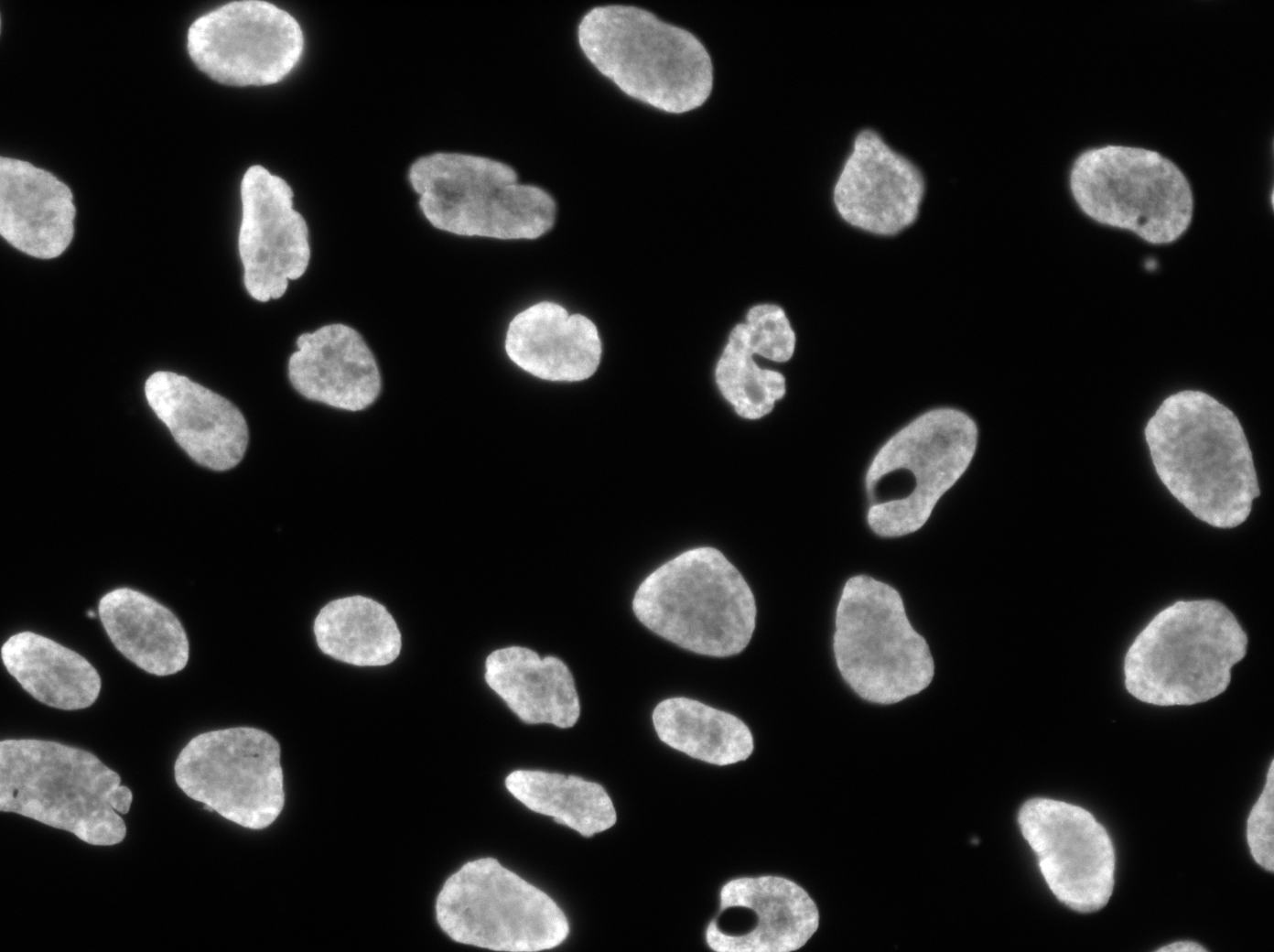

Supplement: Supplementary file 8 — Source Data [file 41467_2021_24153_MOESM8_ESM.zip › RawData/Supplementary Figures/FigS2/b/U3S_48h_siLUC_03_w1DAPI.TIF]

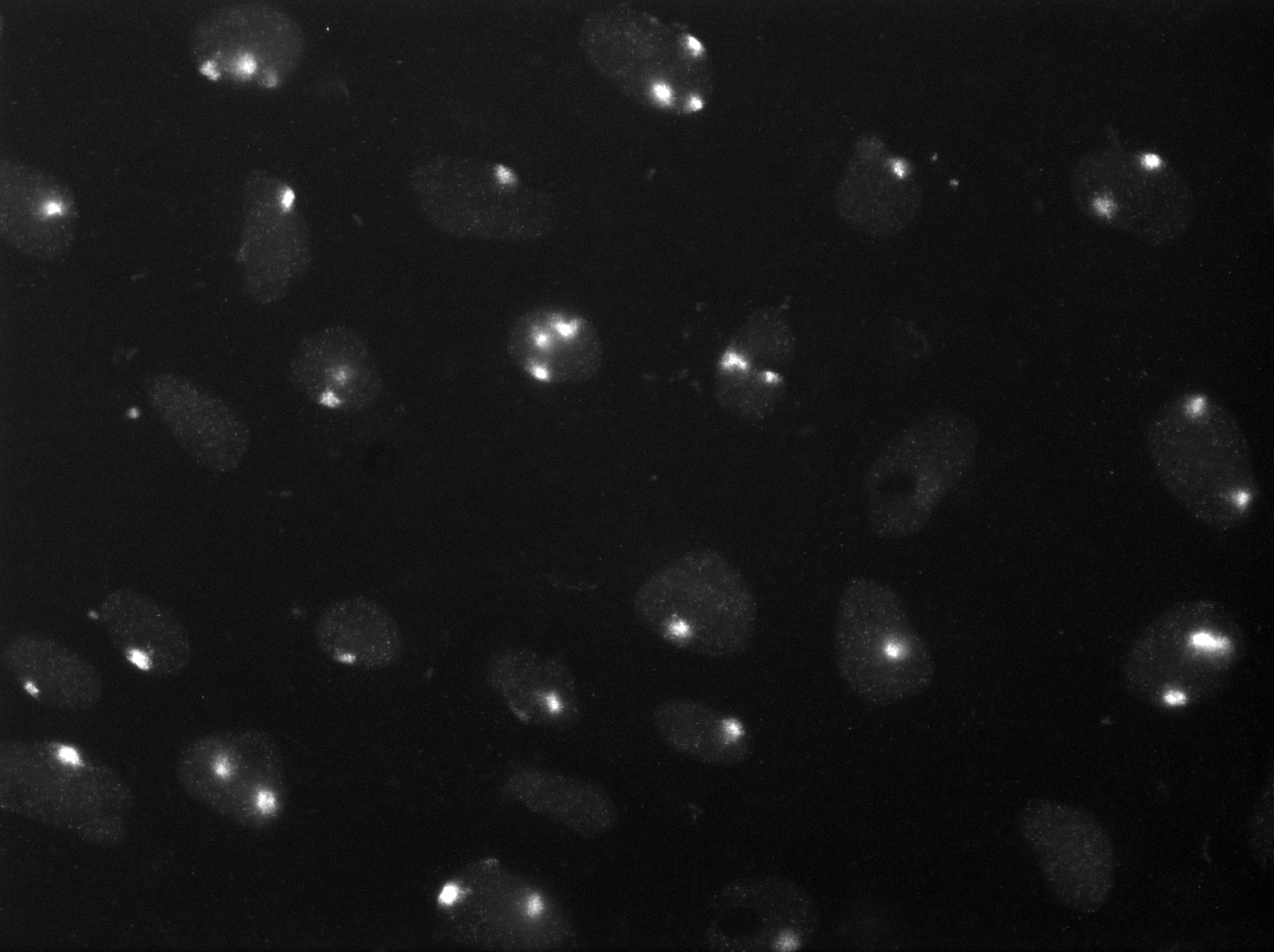

Supplement: Supplementary file 8 — Source Data [file 41467_2021_24153_MOESM8_ESM.zip › RawData/Supplementary Figures/FigS2/b/U3S_48h_siLUC_03_w2GFP.TIF]

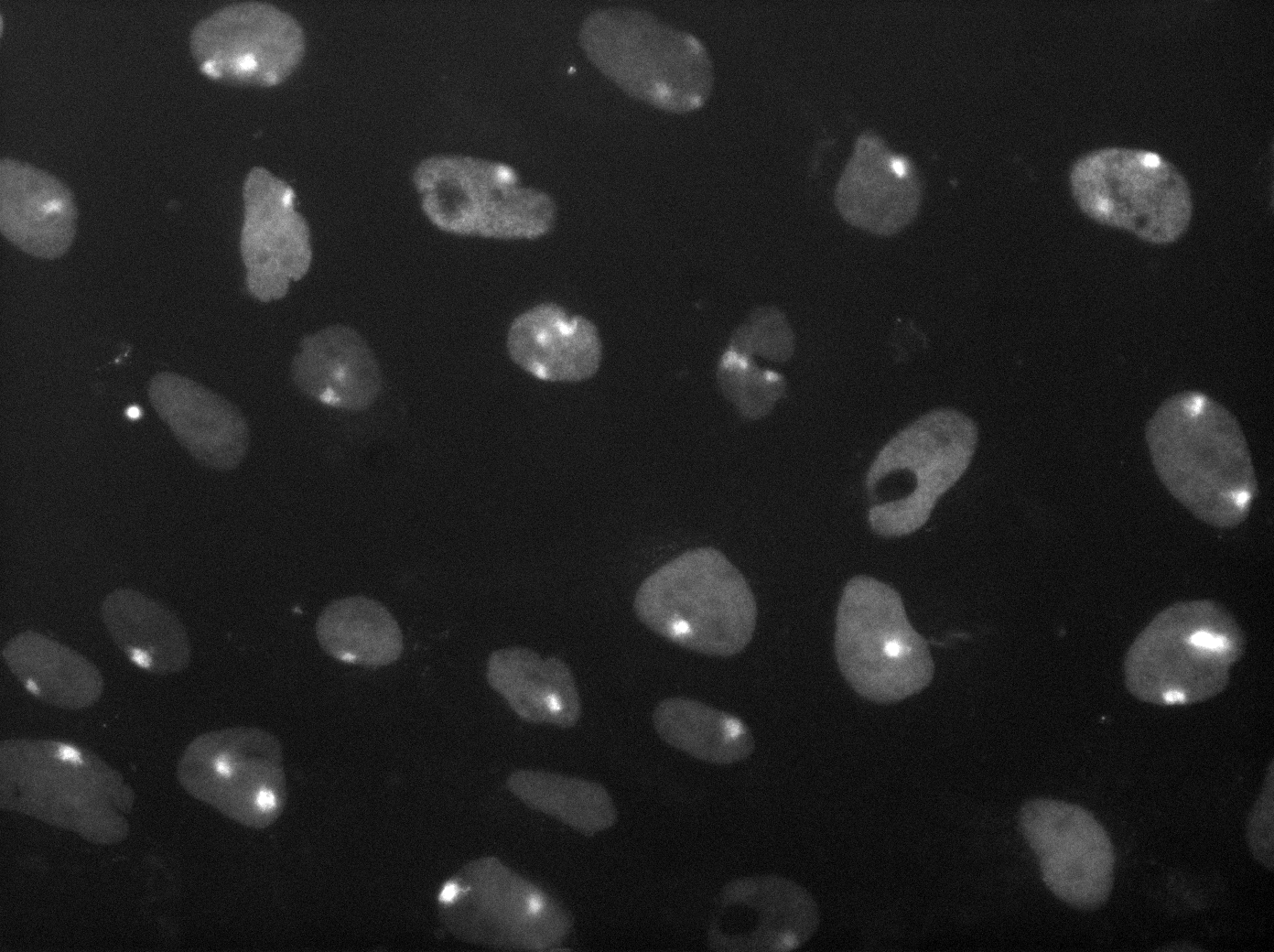

Supplement: Supplementary file 8 — Source Data [file 41467_2021_24153_MOESM8_ESM.zip › RawData/Supplementary Figures/FigS2/b/U3S_48h_siLUC_03_w3CY3.TIF]

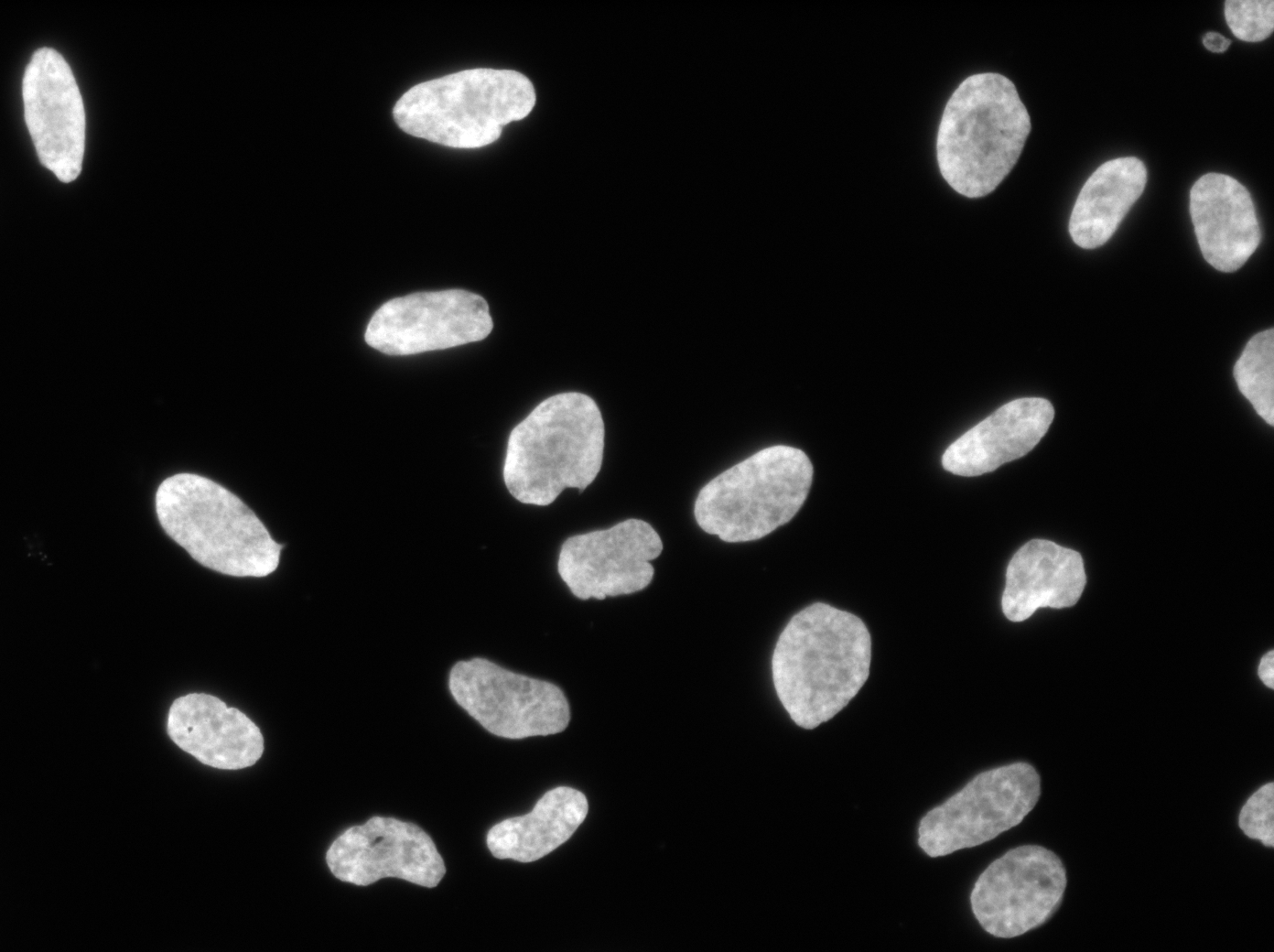

Supplement: Supplementary file 8 — Source Data [file 41467_2021_24153_MOESM8_ESM.zip › RawData/Supplementary Figures/FigS2/b/U3S_48h_siVCP_04_w1DAPI.TIF]

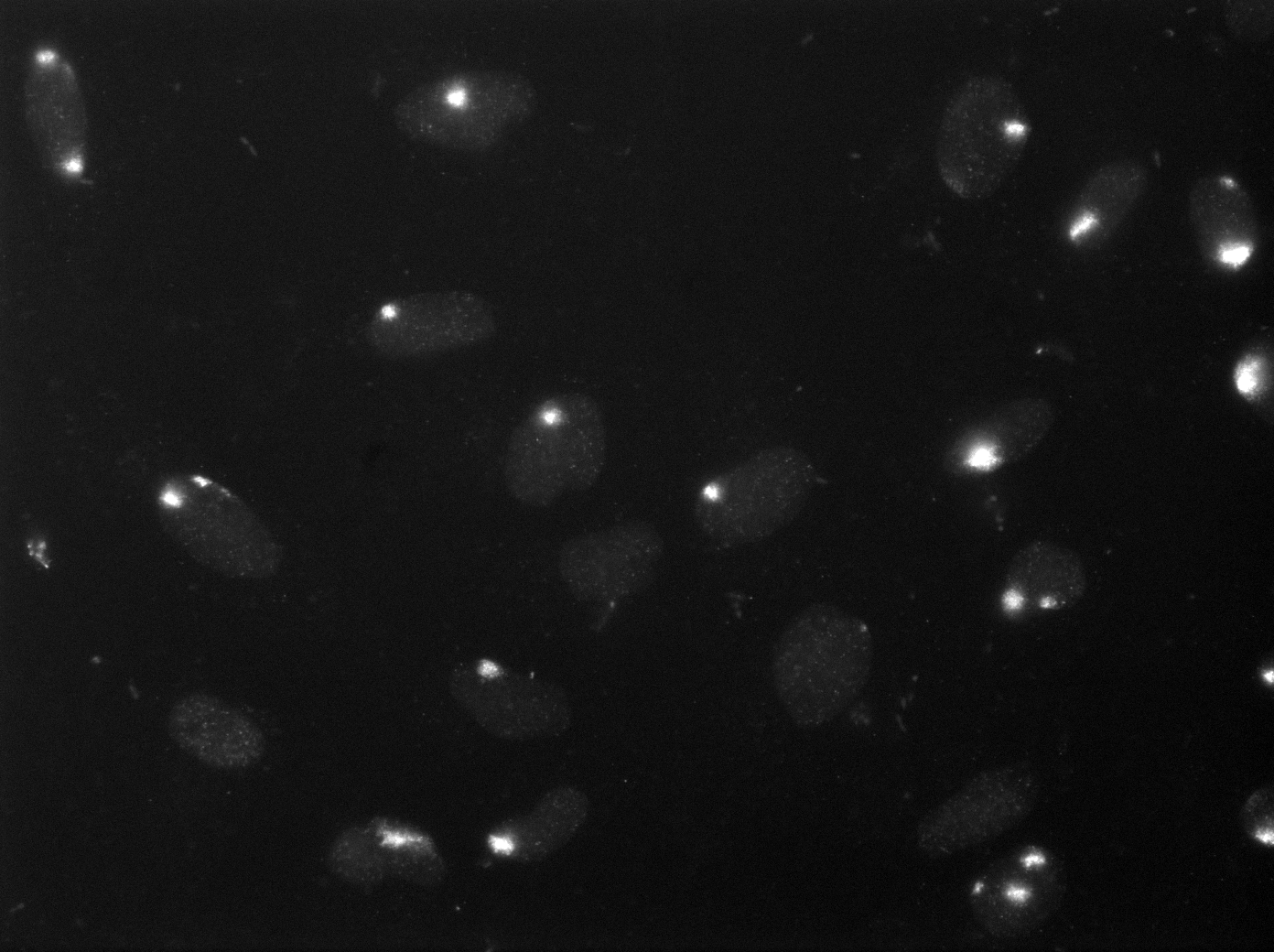

Supplement: Supplementary file 8 — Source Data [file 41467_2021_24153_MOESM8_ESM.zip › RawData/Supplementary Figures/FigS2/b/U3S_48h_siVCP_04_w2GFP.TIF]

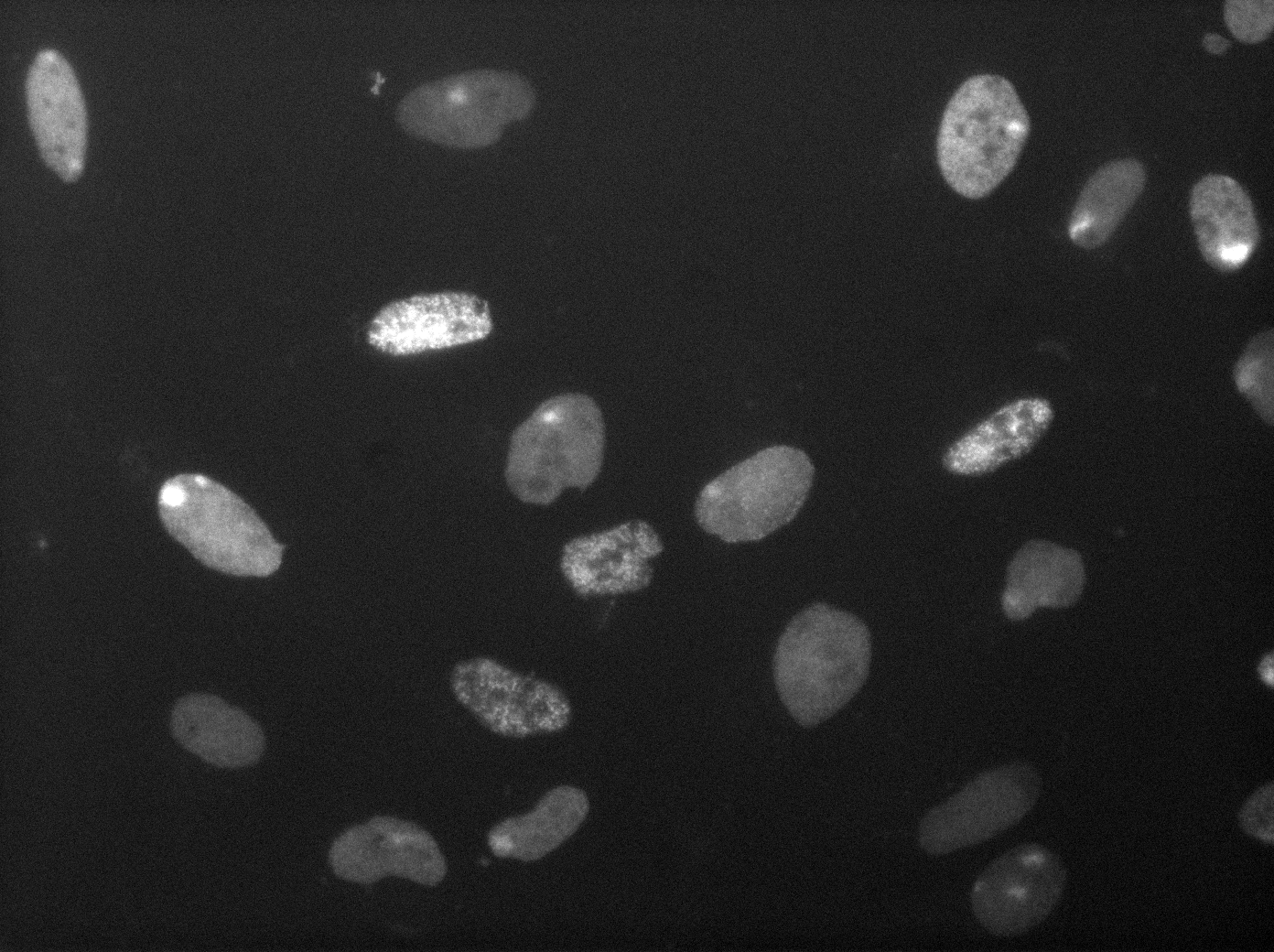

Supplement: Supplementary file 8 — Source Data [file 41467_2021_24153_MOESM8_ESM.zip › RawData/Supplementary Figures/FigS2/b/U3S_48h_siVCP_04_w3CY3.TIF]

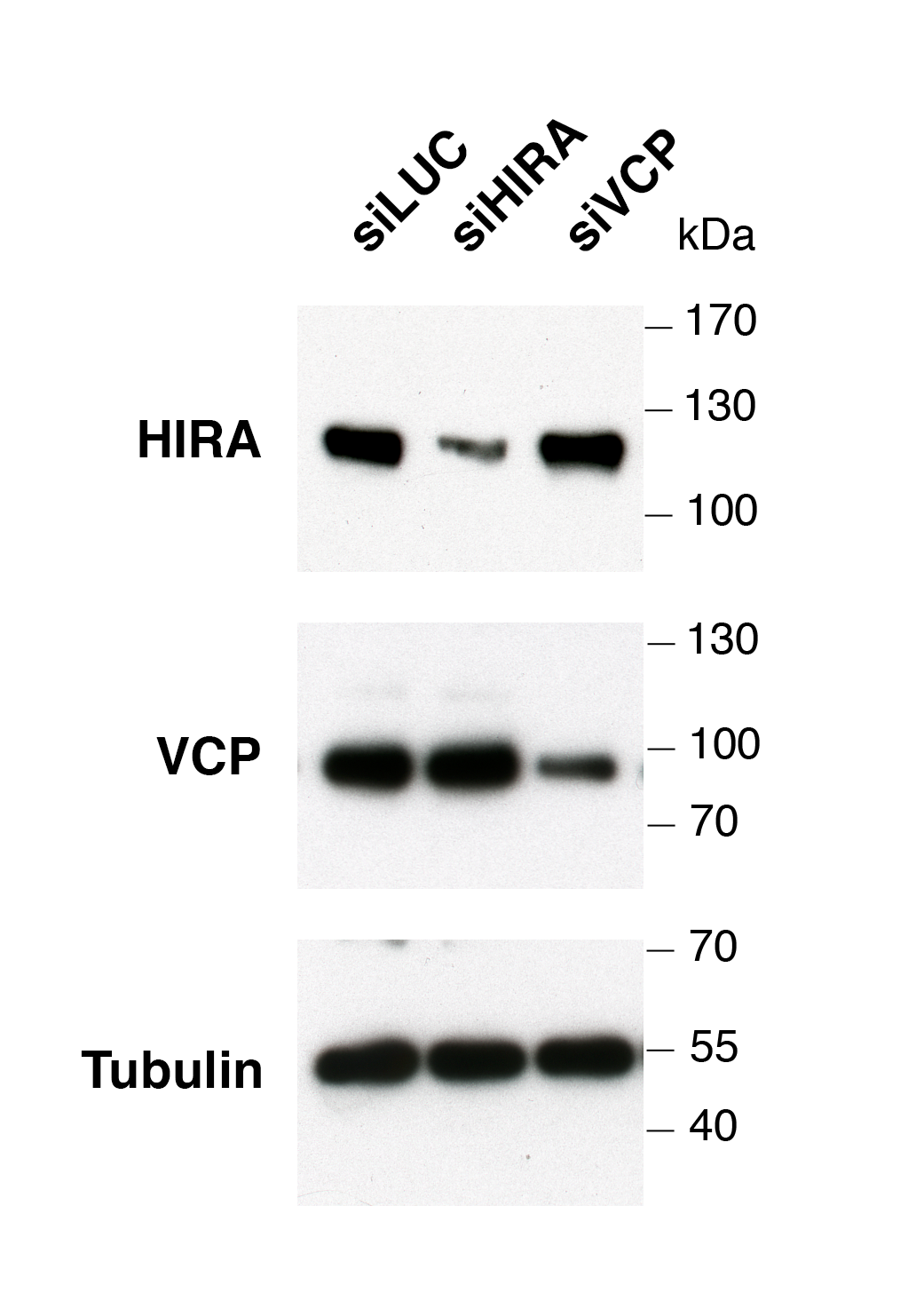

Supplement: Supplementary file 8 — Source Data [file 41467_2021_24153_MOESM8_ESM.zip › RawData/Supplementary Figures/FigS2/b/WB.tif]

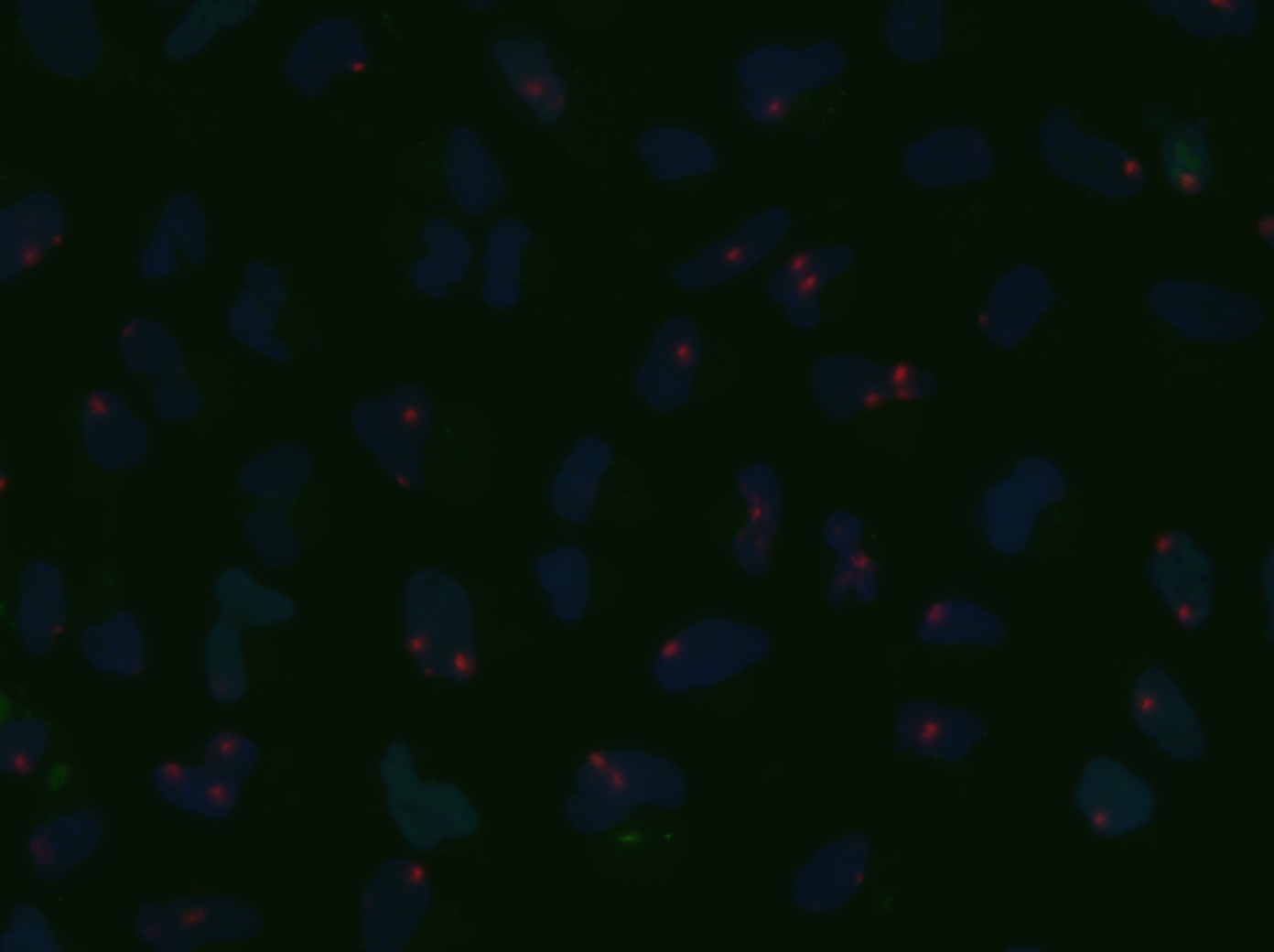

Supplement: Supplementary file 8 — Source Data [file 41467_2021_24153_MOESM8_ESM.zip › RawData/Supplementary Figures/FigS2/c/siDDB2_1hpostUVC_newH33-O488_CPD-AF568_04.tif]

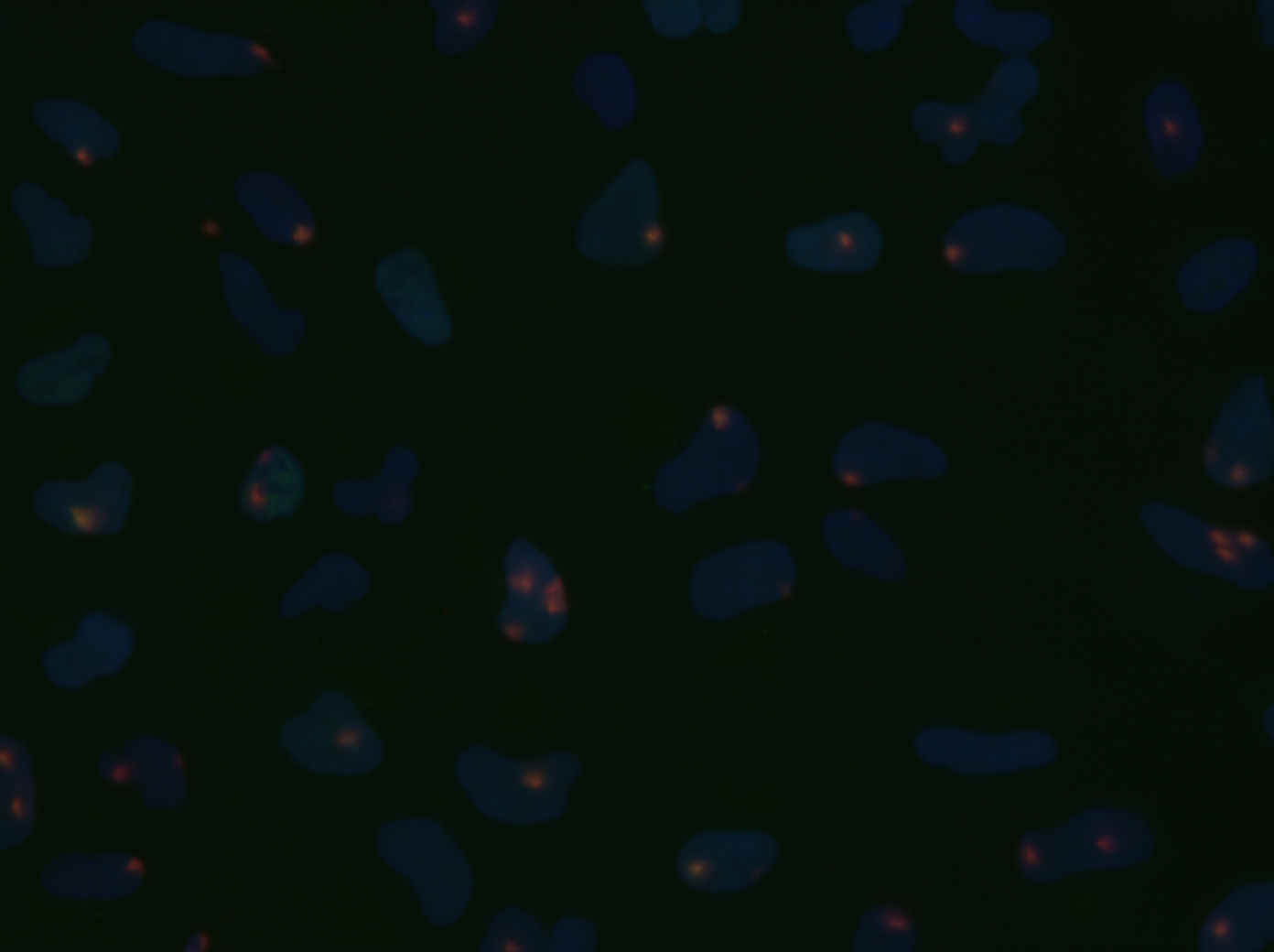

Supplement: Supplementary file 8 — Source Data [file 41467_2021_24153_MOESM8_ESM.zip › RawData/Supplementary Figures/FigS2/c/siLUC_1hpostUVC_newH33-O488_CPD-AF568_04.tif]

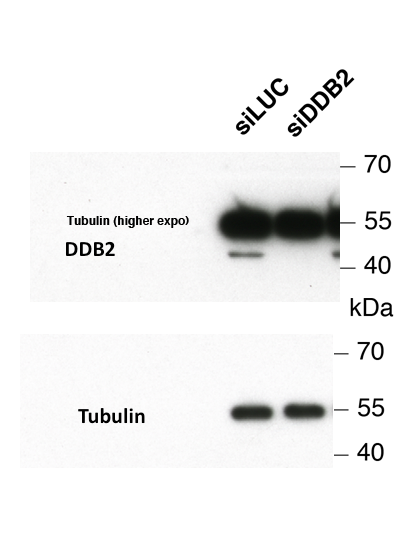

Supplement: Supplementary file 8 — Source Data [file 41467_2021_24153_MOESM8_ESM.zip › RawData/Supplementary Figures/FigS2/c/WB.tif]

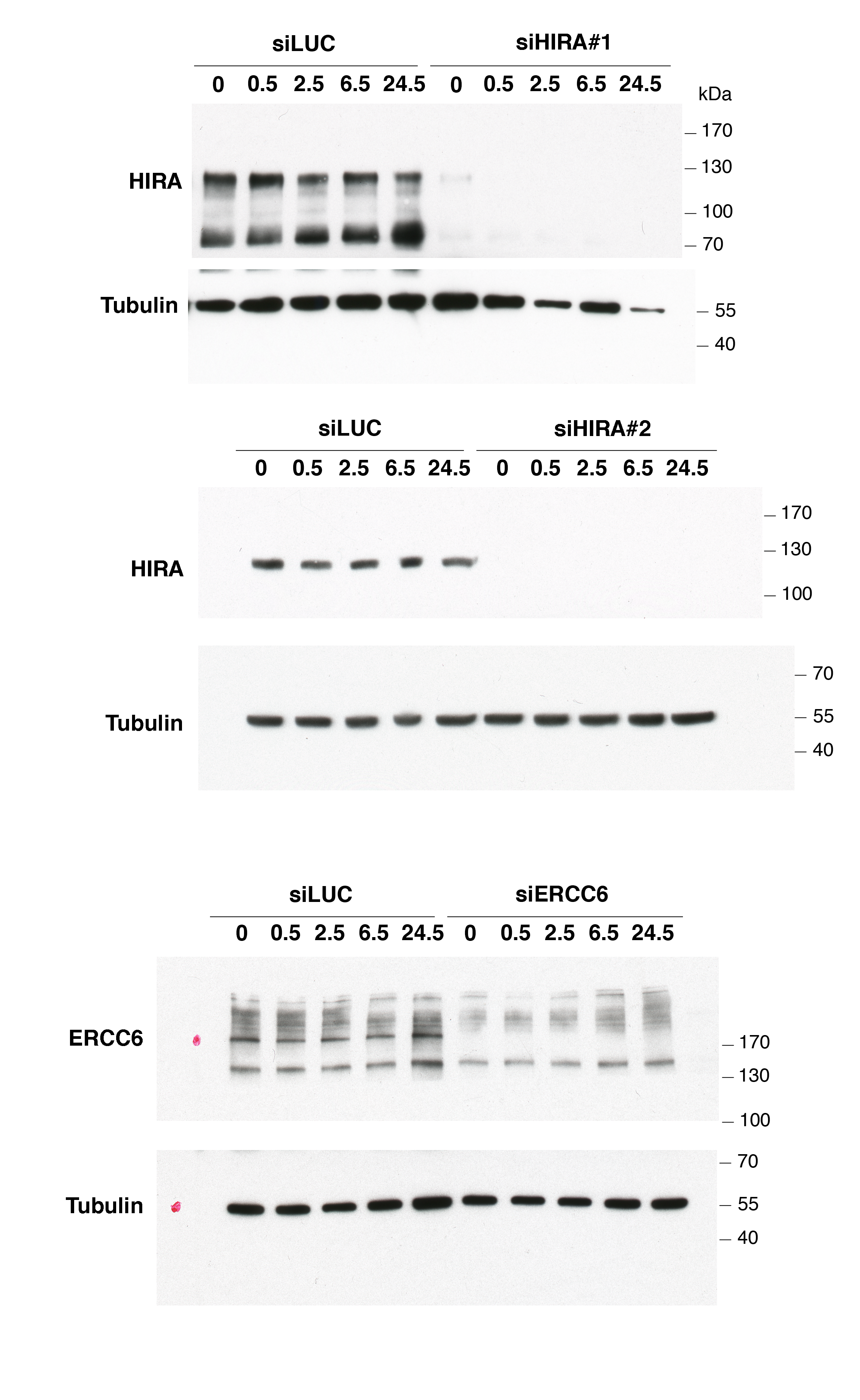

Supplement: Supplementary file 8 — Source Data [file 41467_2021_24153_MOESM8_ESM.zip › RawData/Supplementary Figures/FigS3/a/WB.tif]

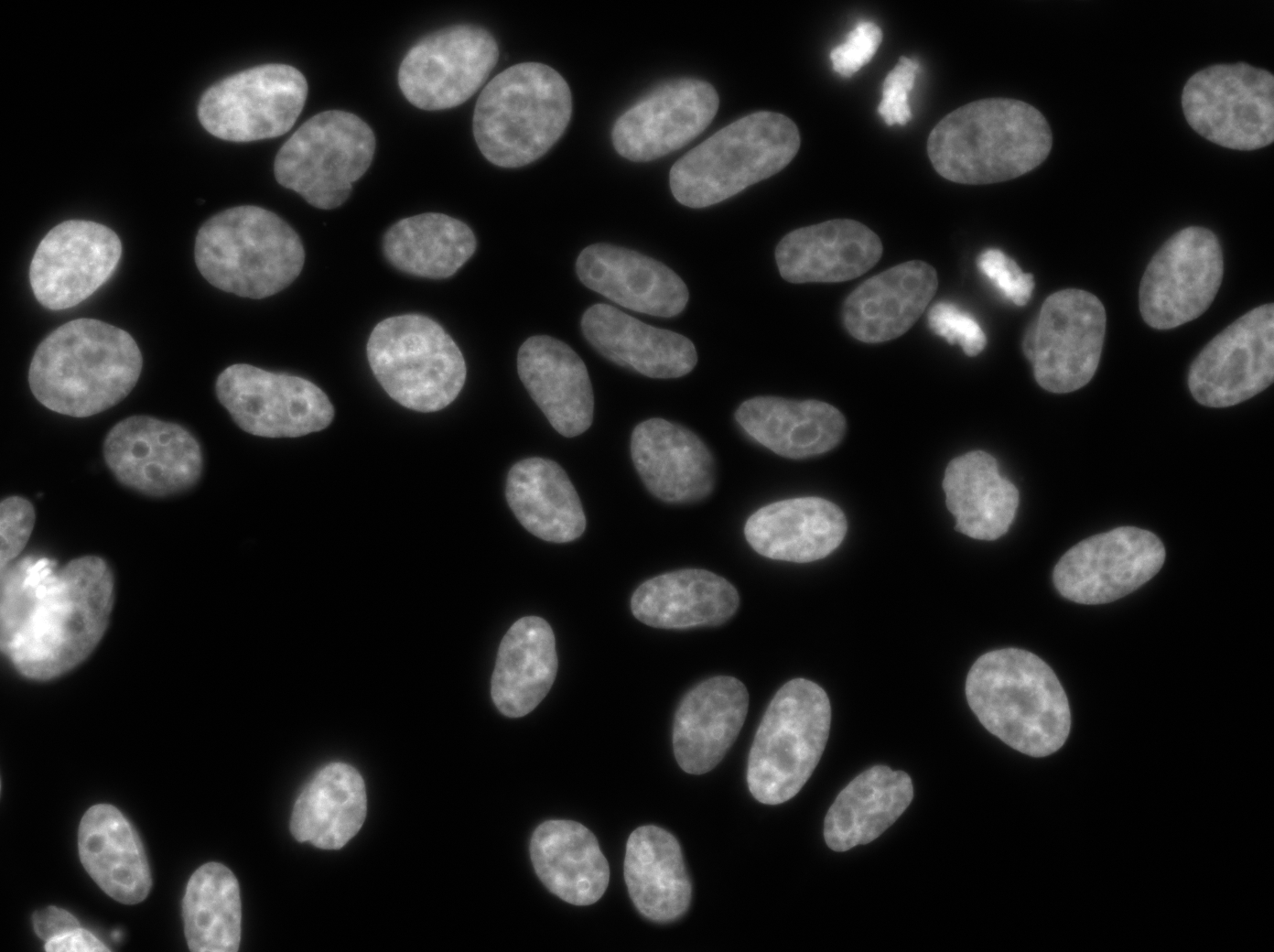

Supplement: Supplementary file 8 — Source Data [file 41467_2021_24153_MOESM8_ESM.zip › RawData/Supplementary Figures/FigS3/b/IF/siERCC6_0h_4_w1DAPI.TIF]

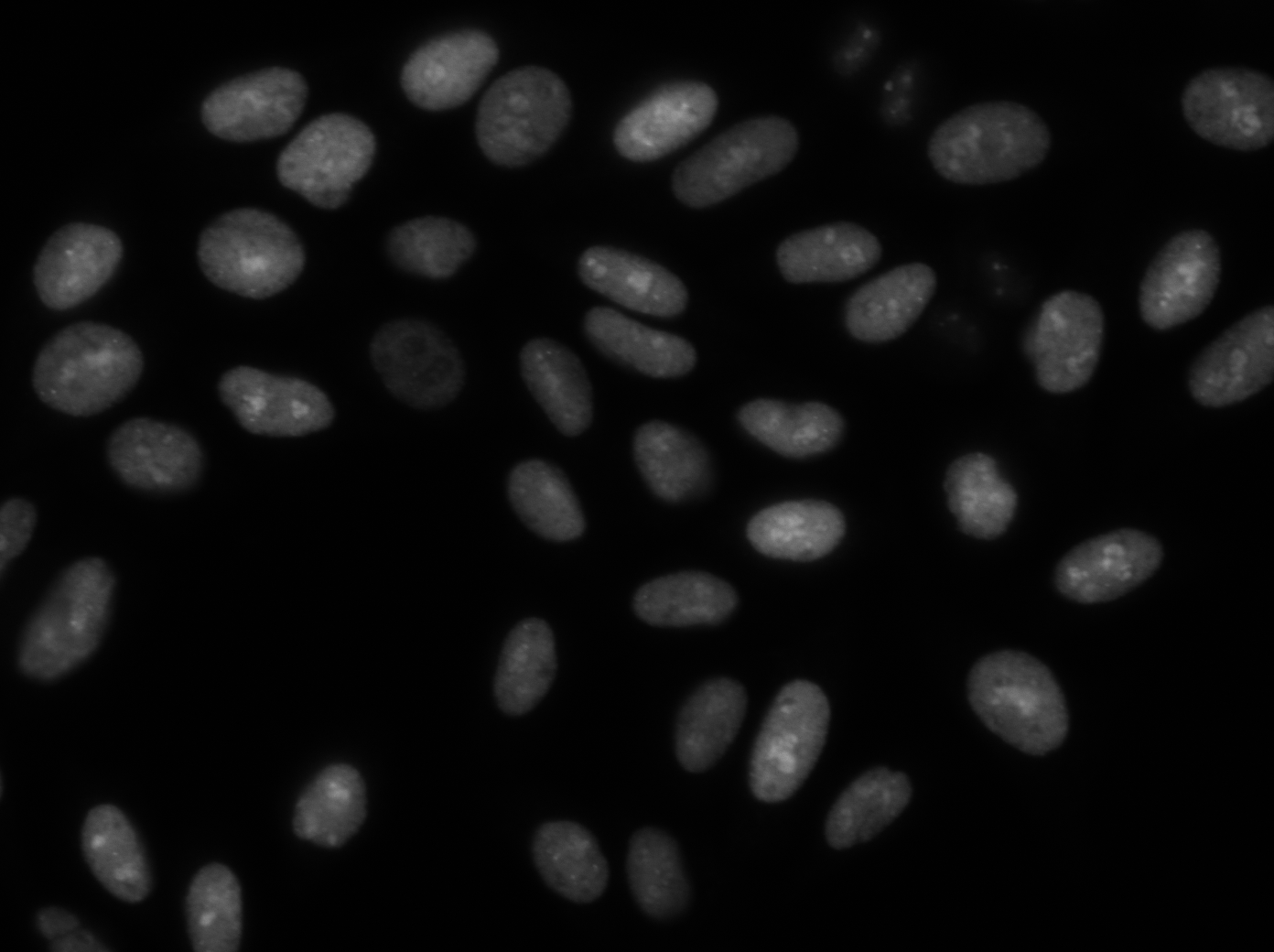

Supplement: Supplementary file 8 — Source Data [file 41467_2021_24153_MOESM8_ESM.zip › RawData/Supplementary Figures/FigS3/b/IF/siERCC6_0h_4_w2TX.TIF]

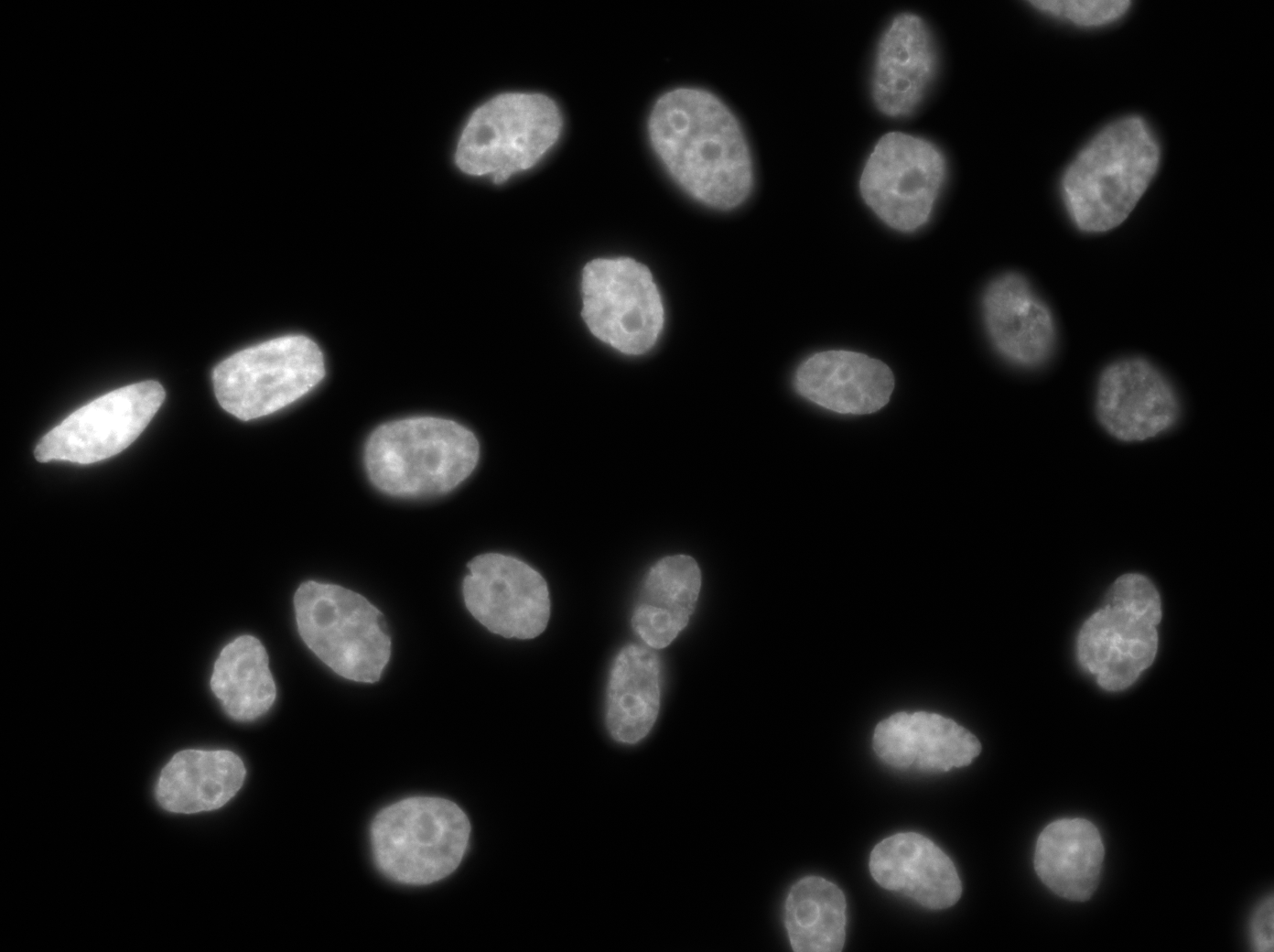

Supplement: Supplementary file 8 — Source Data [file 41467_2021_24153_MOESM8_ESM.zip › RawData/Supplementary Figures/FigS3/b/IF/siERCC6_24h_9_w1DAPI.TIF]

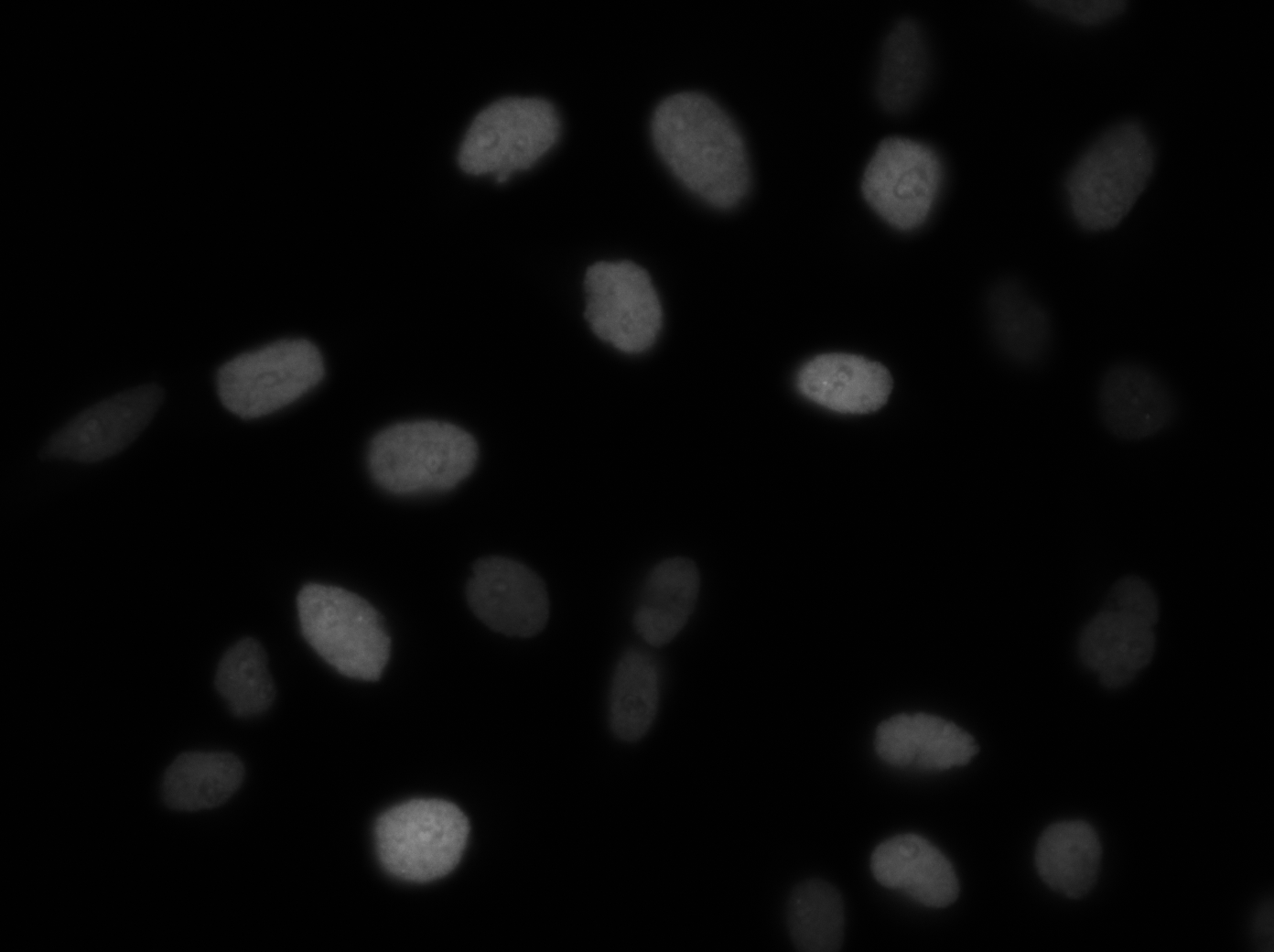

Supplement: Supplementary file 8 — Source Data [file 41467_2021_24153_MOESM8_ESM.zip › RawData/Supplementary Figures/FigS3/b/IF/siERCC6_24h_9_w2TX.TIF]

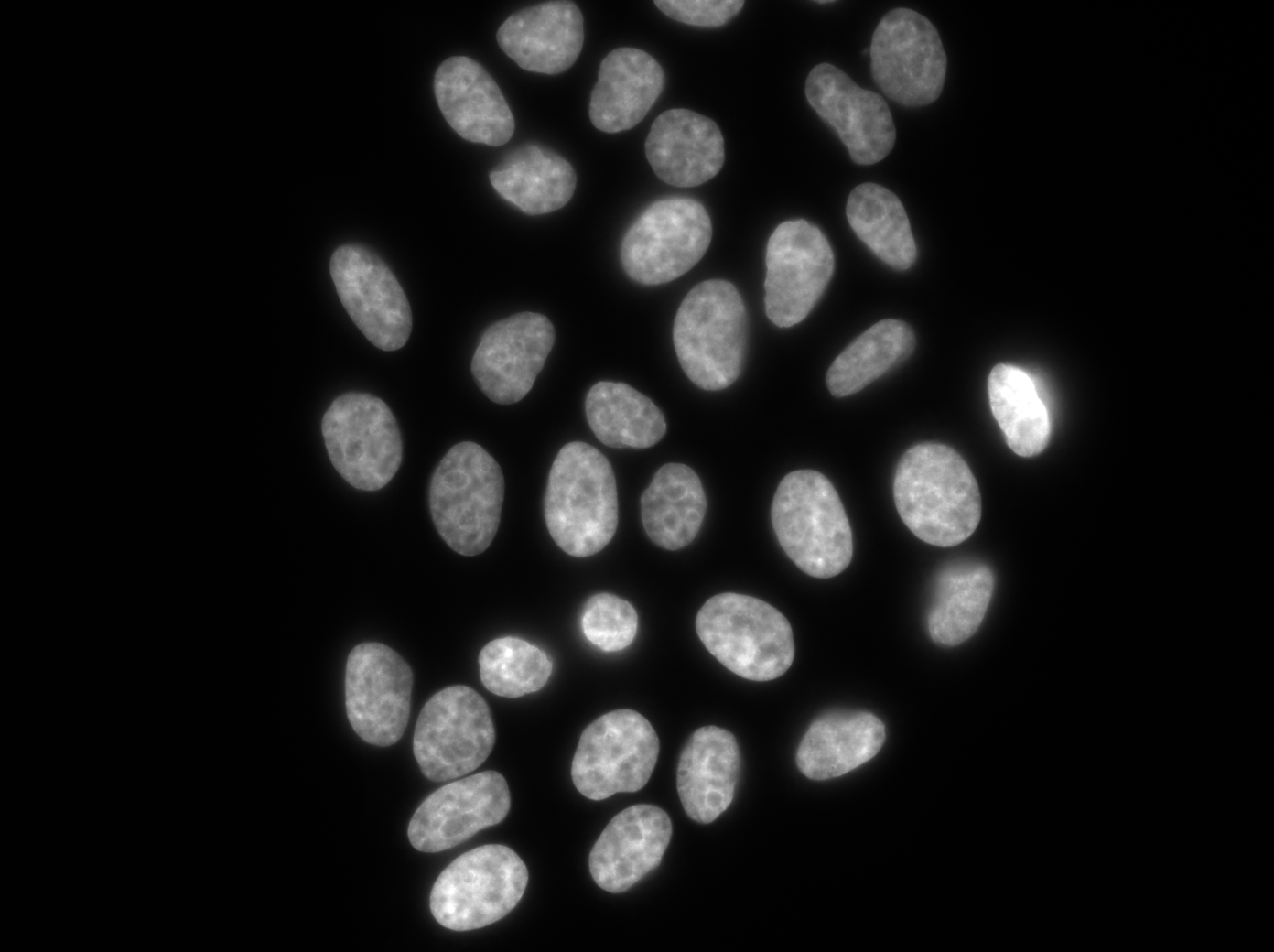

Supplement: Supplementary file 8 — Source Data [file 41467_2021_24153_MOESM8_ESM.zip › RawData/Supplementary Figures/FigS3/b/IF/siERCC6_2h_5_w1DAPI.TIF]

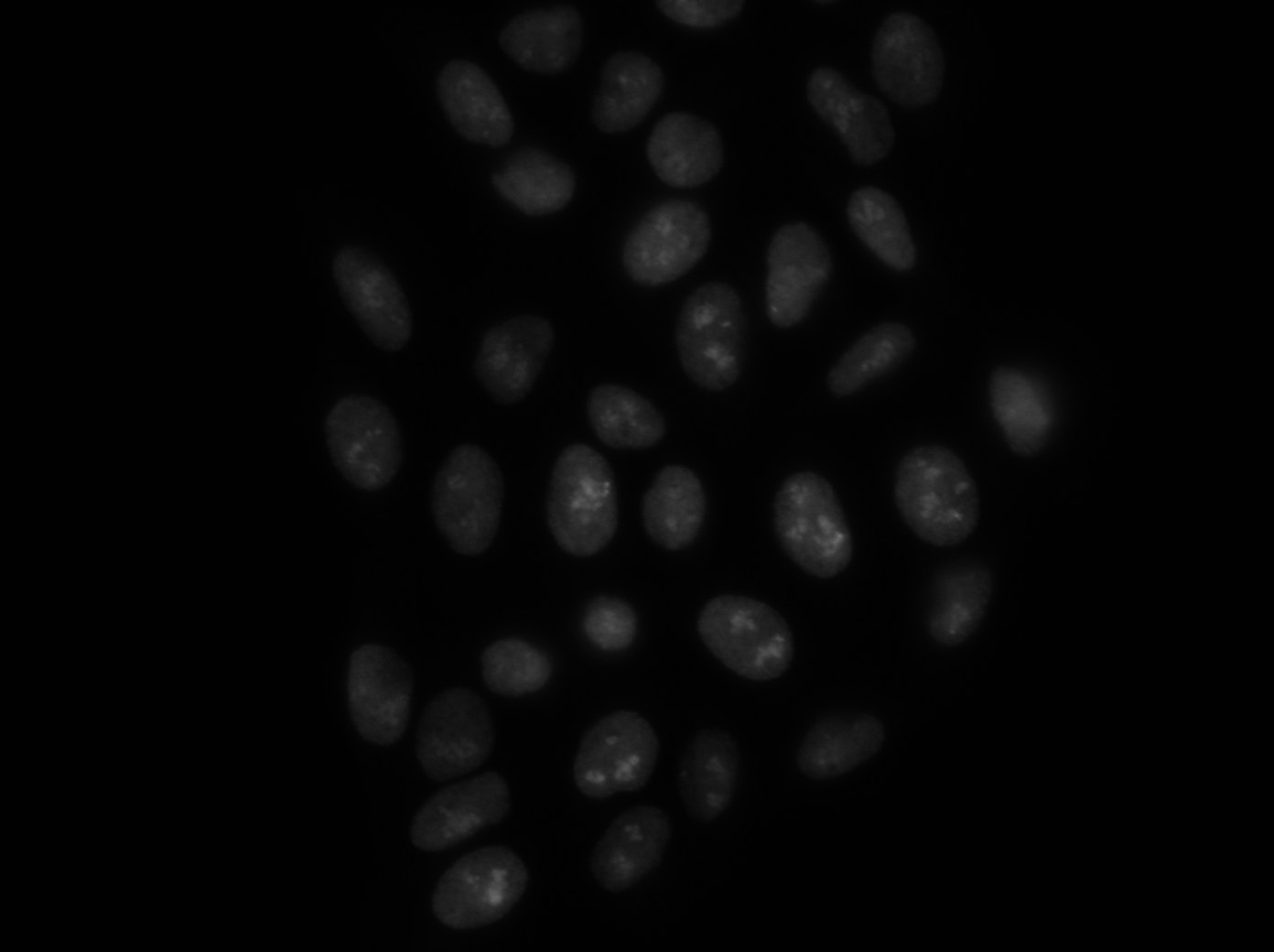

Supplement: Supplementary file 8 — Source Data [file 41467_2021_24153_MOESM8_ESM.zip › RawData/Supplementary Figures/FigS3/b/IF/siERCC6_2h_5_w2TX.TIF]

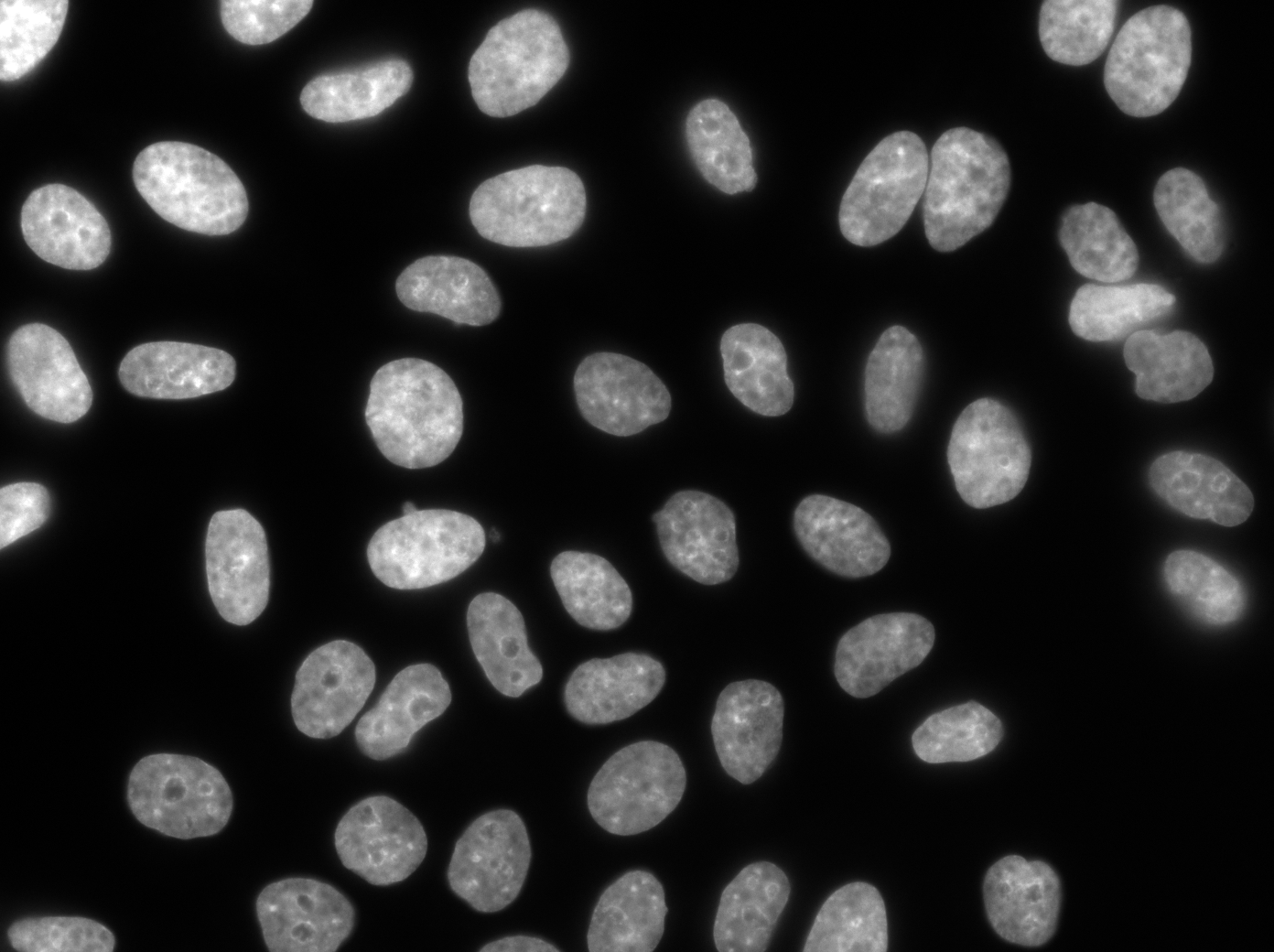

Supplement: Supplementary file 8 — Source Data [file 41467_2021_24153_MOESM8_ESM.zip › RawData/Supplementary Figures/FigS3/b/IF/siERCC6_6h_3_w1DAPI.TIF]

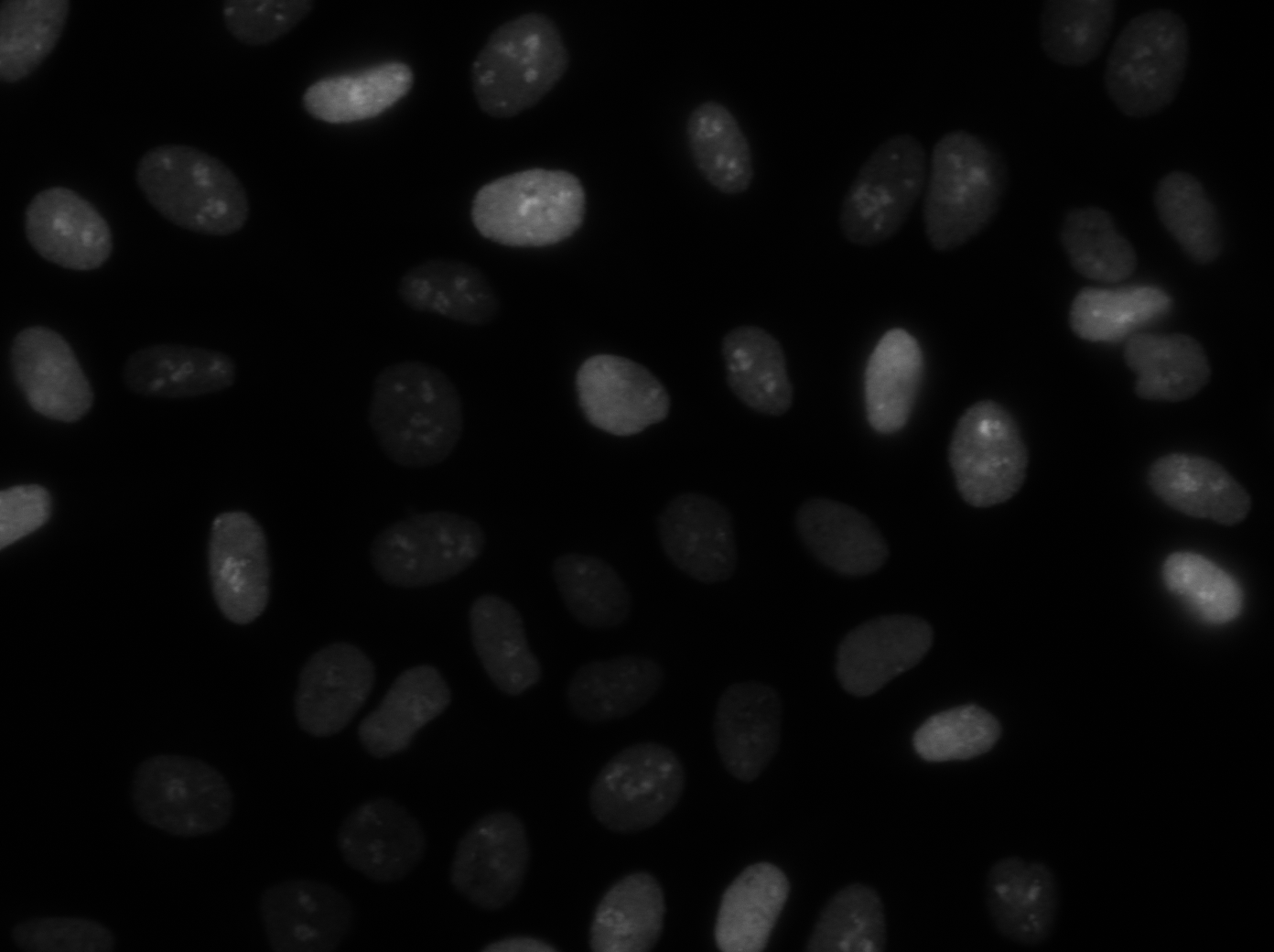

Supplement: Supplementary file 8 — Source Data [file 41467_2021_24153_MOESM8_ESM.zip › RawData/Supplementary Figures/FigS3/b/IF/siERCC6_6h_3_w2TX.TIF]

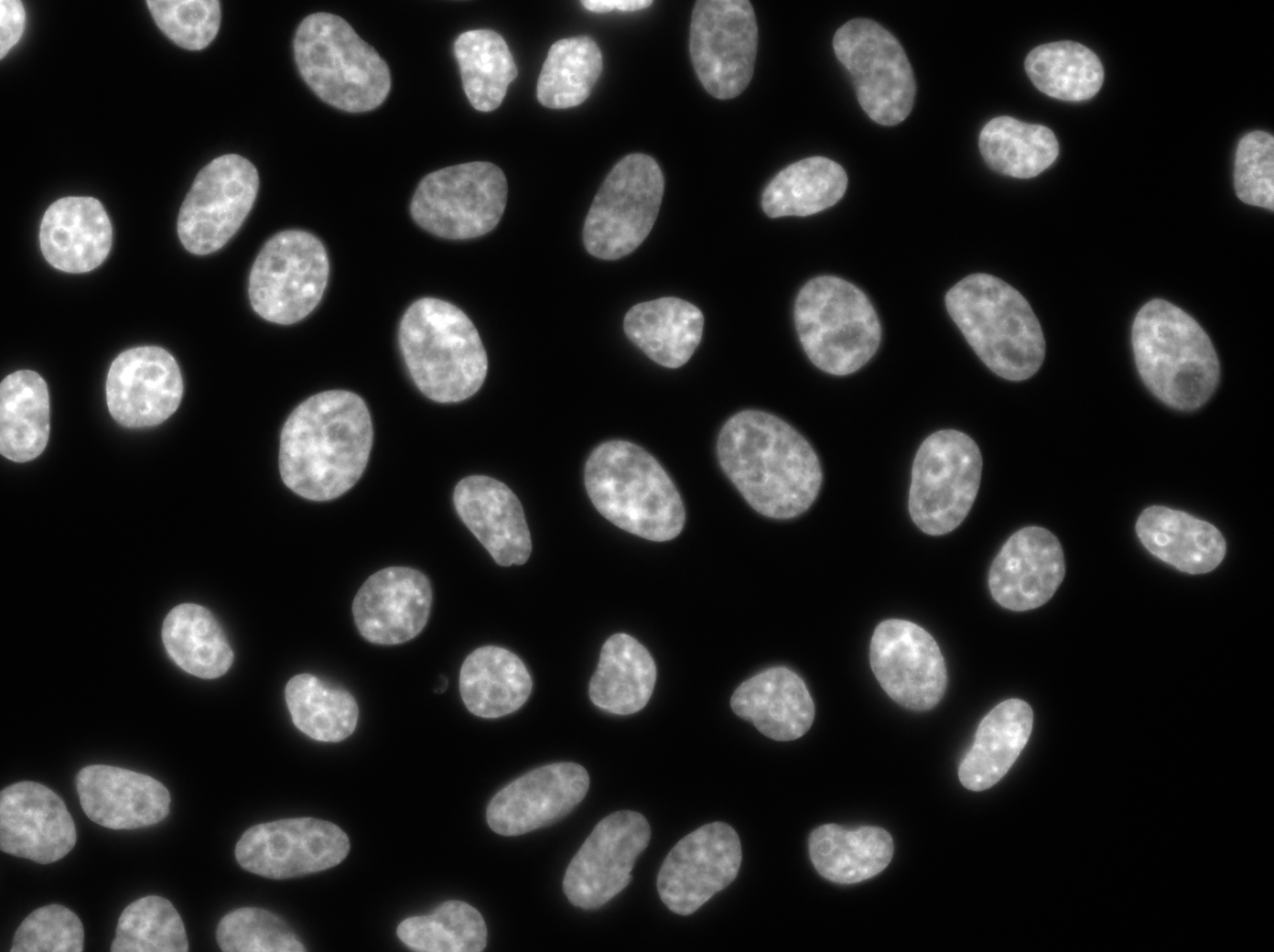

Supplement: Supplementary file 8 — Source Data [file 41467_2021_24153_MOESM8_ESM.zip › RawData/Supplementary Figures/FigS3/b/IF/siERCC6_ut_3_w1DAPI.TIF]

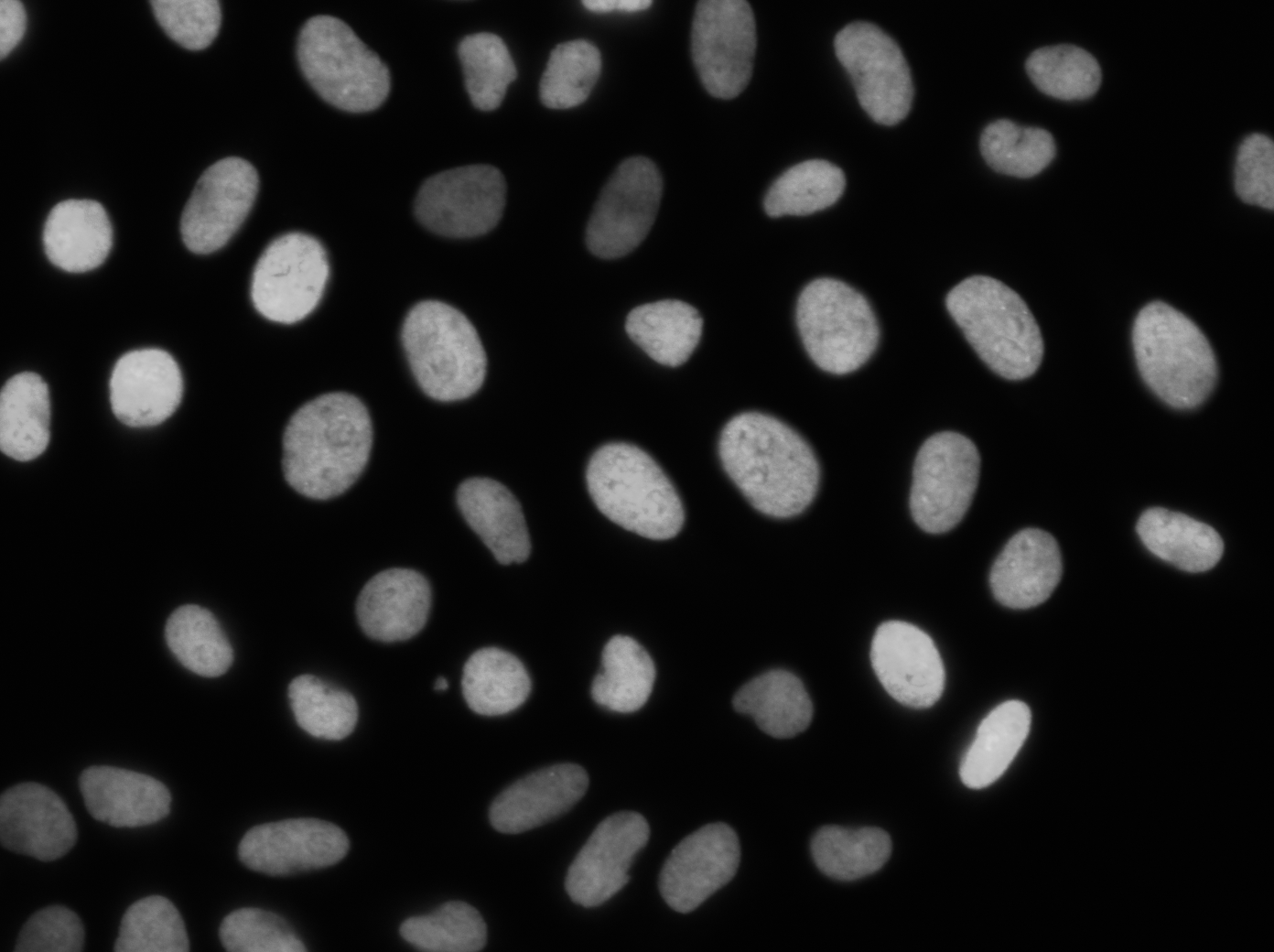

Supplement: Supplementary file 8 — Source Data [file 41467_2021_24153_MOESM8_ESM.zip › RawData/Supplementary Figures/FigS3/b/IF/siERCC6_ut_3_w2TX.TIF]

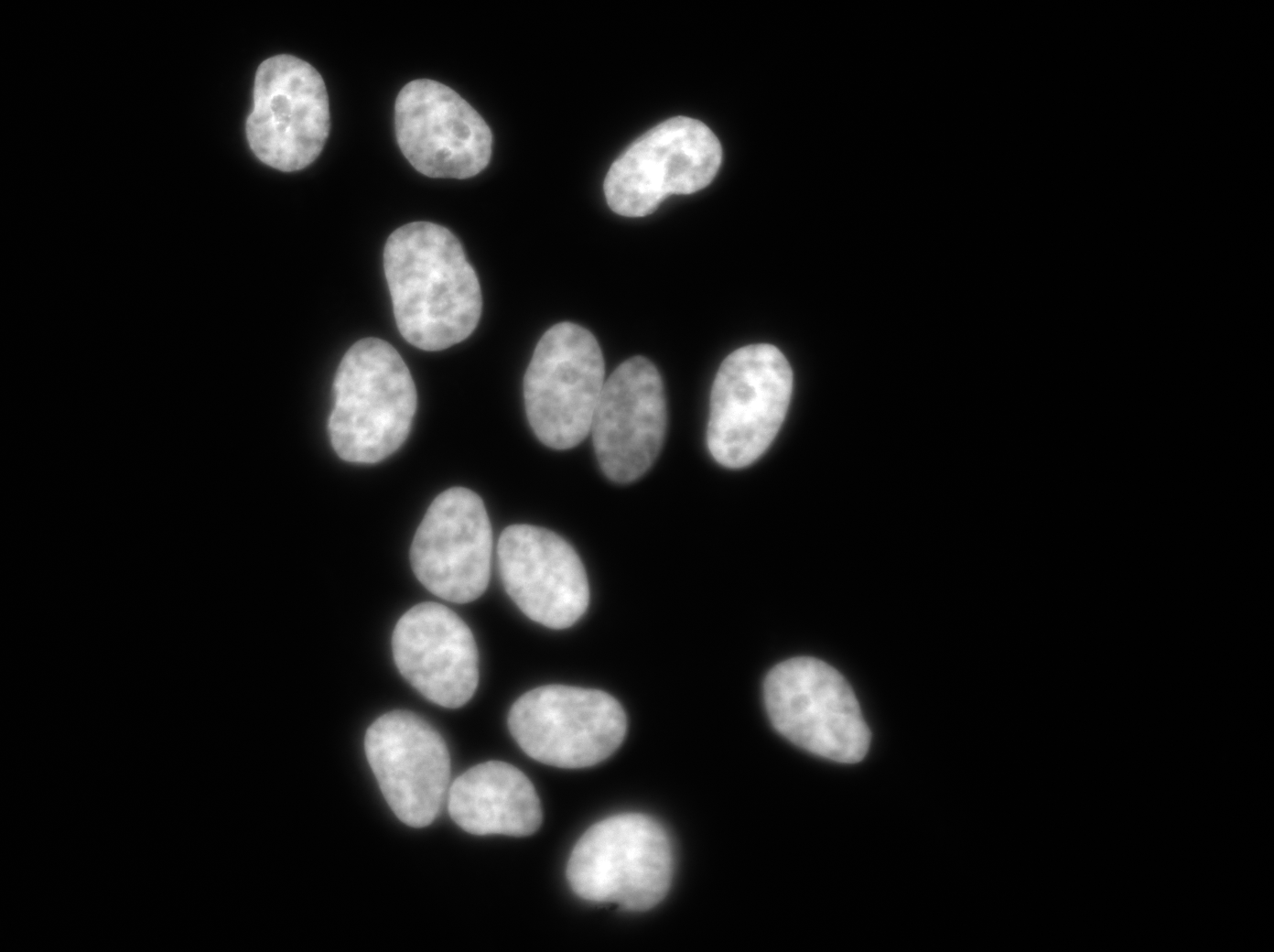

Supplement: Supplementary file 8 — Source Data [file 41467_2021_24153_MOESM8_ESM.zip › RawData/Supplementary Figures/FigS3/b/IF/siHIRA1_0h_3_w1DAPI.TIF]

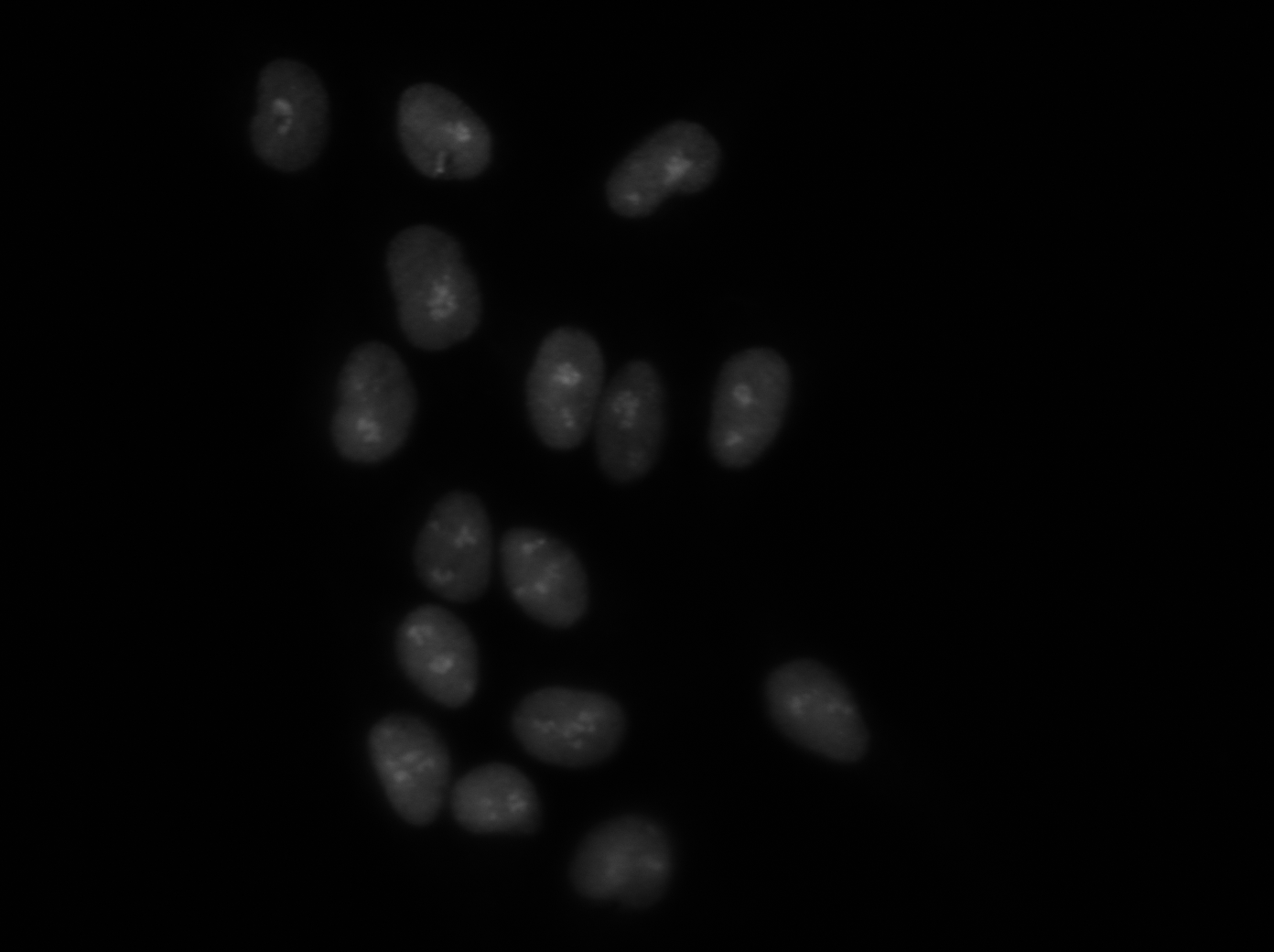

Supplement: Supplementary file 8 — Source Data [file 41467_2021_24153_MOESM8_ESM.zip › RawData/Supplementary Figures/FigS3/b/IF/siHIRA1_0h_3_w2TX.TIF]

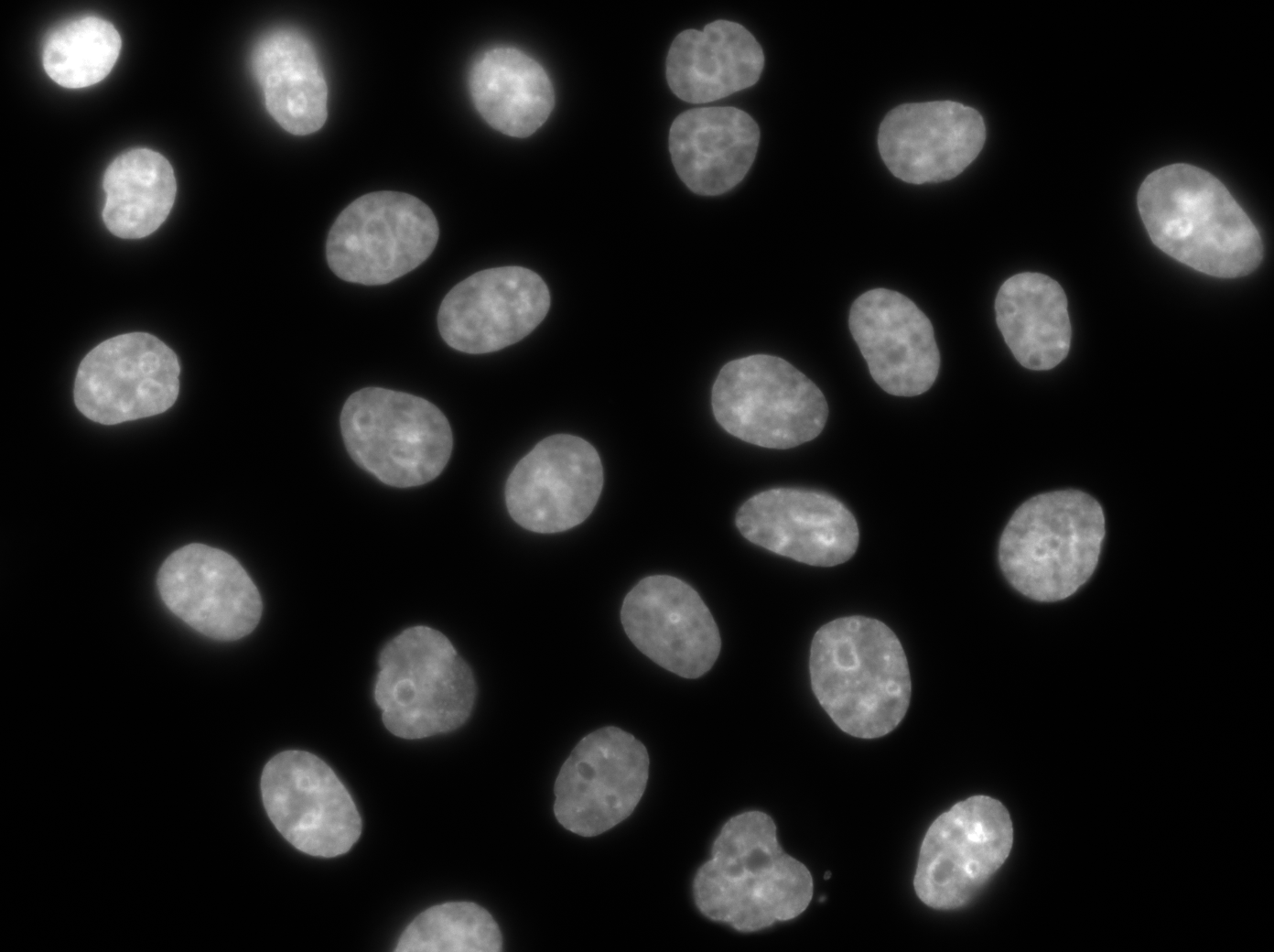

Supplement: Supplementary file 8 — Source Data [file 41467_2021_24153_MOESM8_ESM.zip › RawData/Supplementary Figures/FigS3/b/IF/siHIRA1_24h_7_w1DAPI.TIF]

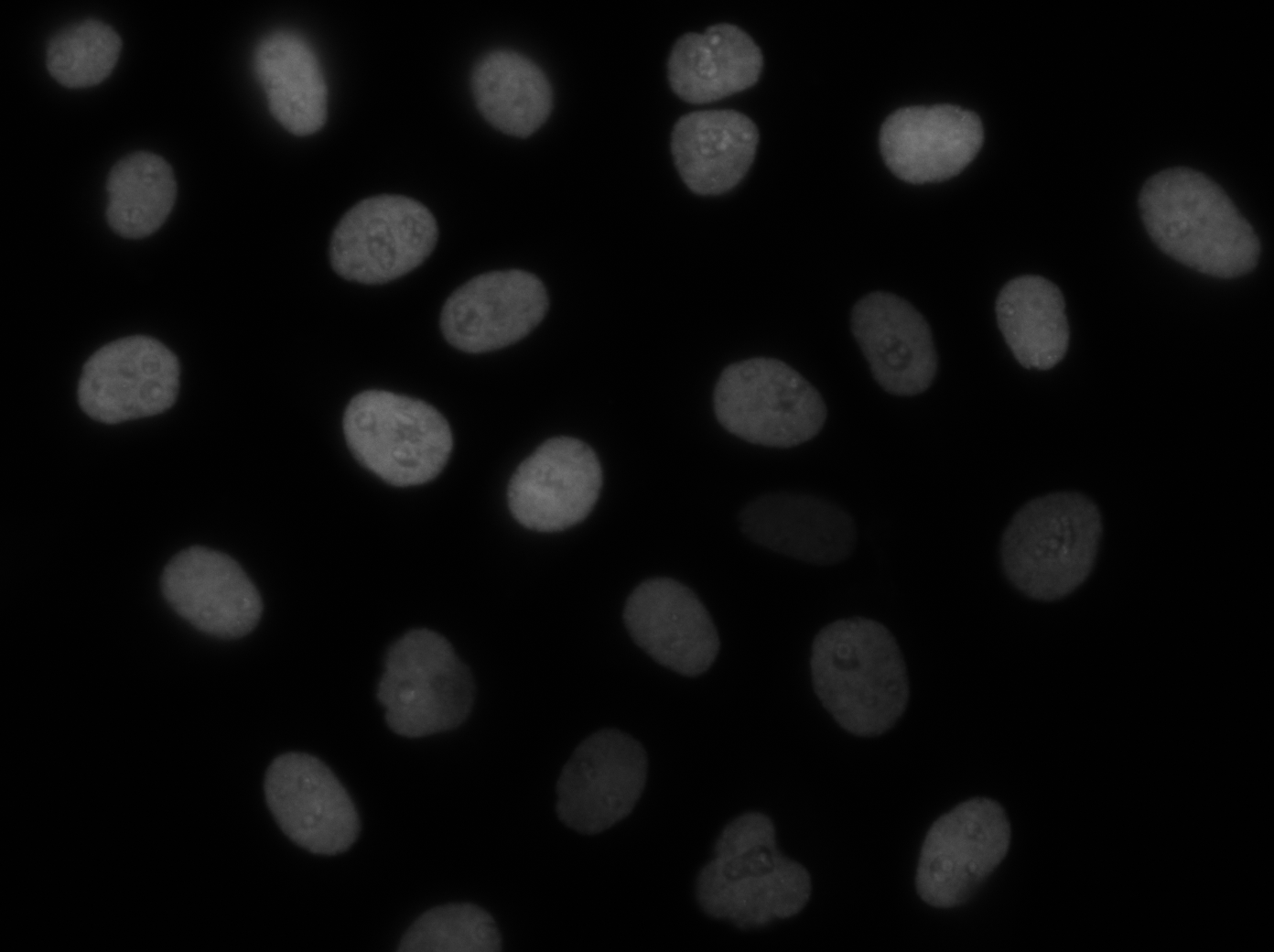

Supplement: Supplementary file 8 — Source Data [file 41467_2021_24153_MOESM8_ESM.zip › RawData/Supplementary Figures/FigS3/b/IF/siHIRA1_24h_7_w2TX.TIF]

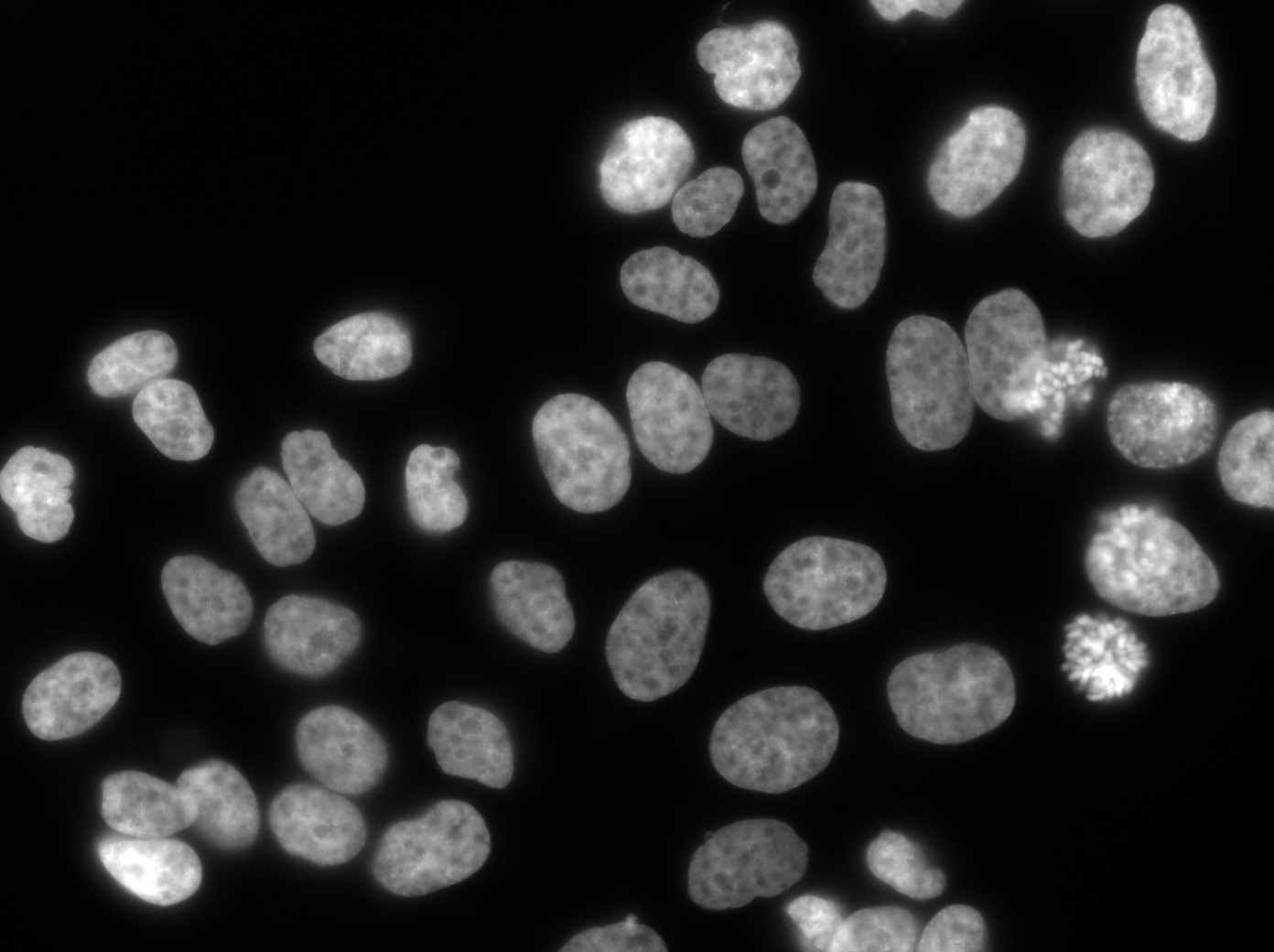

Supplement: Supplementary file 8 — Source Data [file 41467_2021_24153_MOESM8_ESM.zip › RawData/Supplementary Figures/FigS3/b/IF/siHIRA1_2h_1_w1DAPI.TIF]

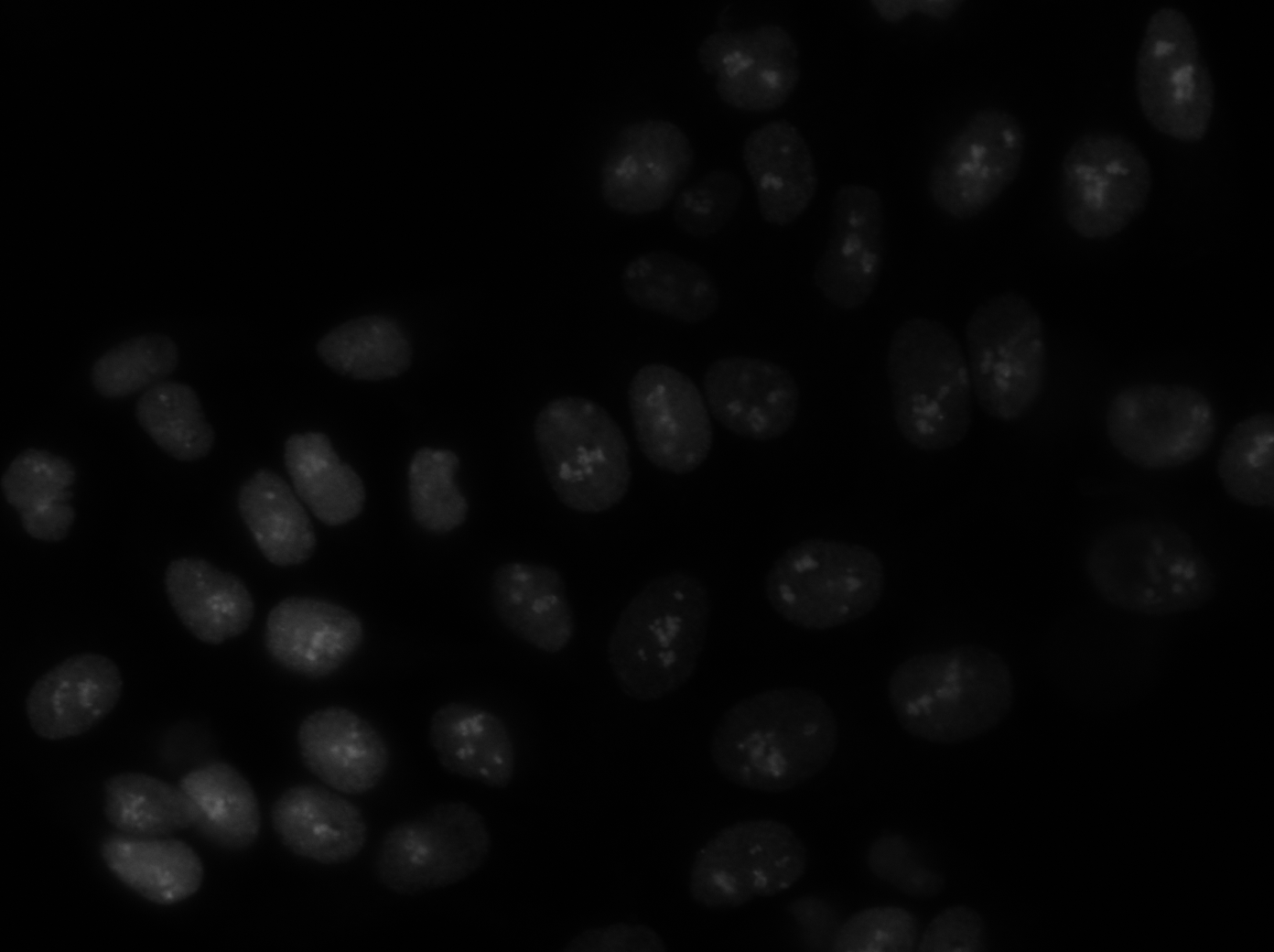

Supplement: Supplementary file 8 — Source Data [file 41467_2021_24153_MOESM8_ESM.zip › RawData/Supplementary Figures/FigS3/b/IF/siHIRA1_2h_1_w2TX.TIF]

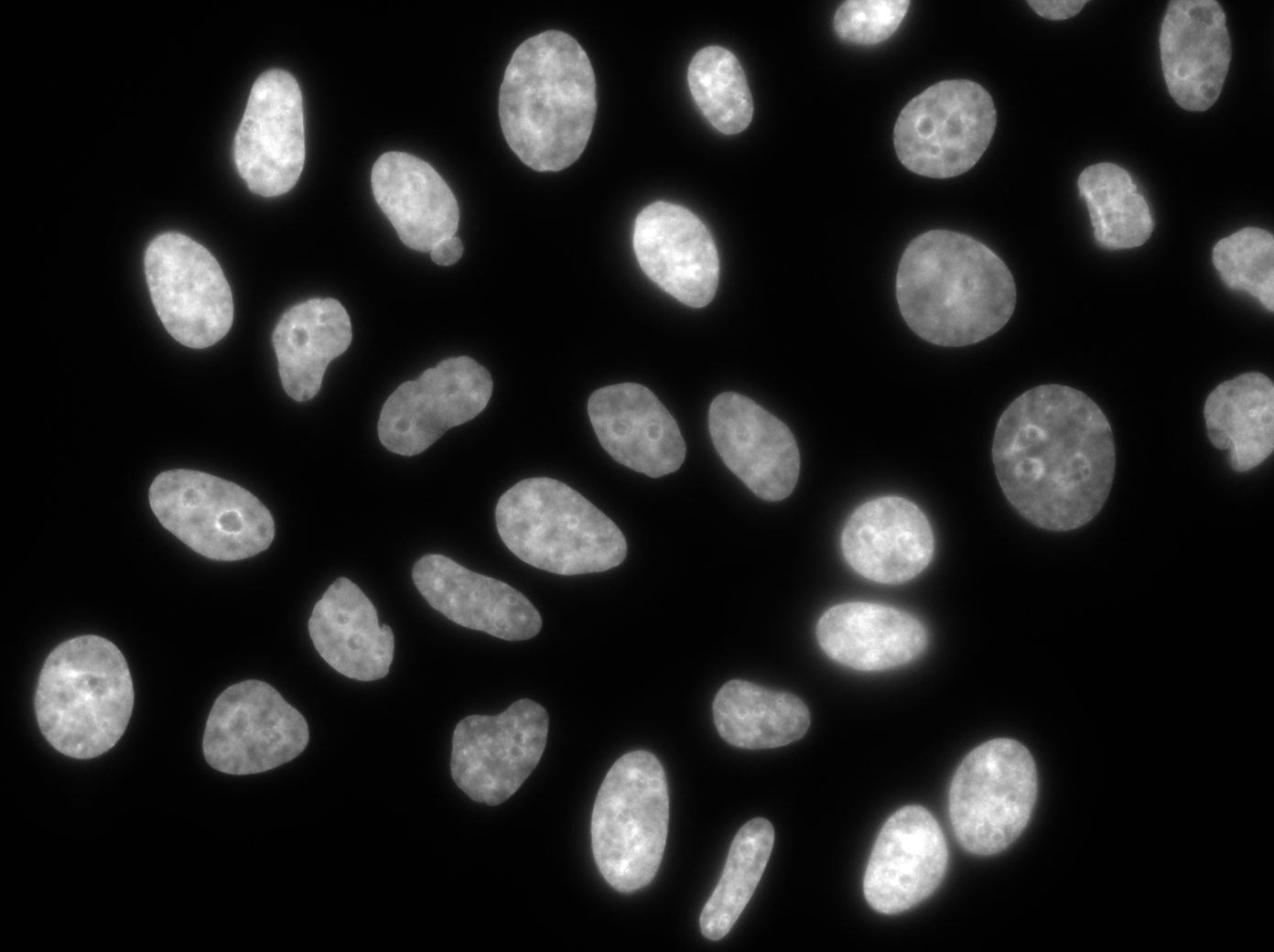

Supplement: Supplementary file 8 — Source Data [file 41467_2021_24153_MOESM8_ESM.zip › RawData/Supplementary Figures/FigS3/b/IF/siHIRA1_6h_2_w1DAPI.TIF]

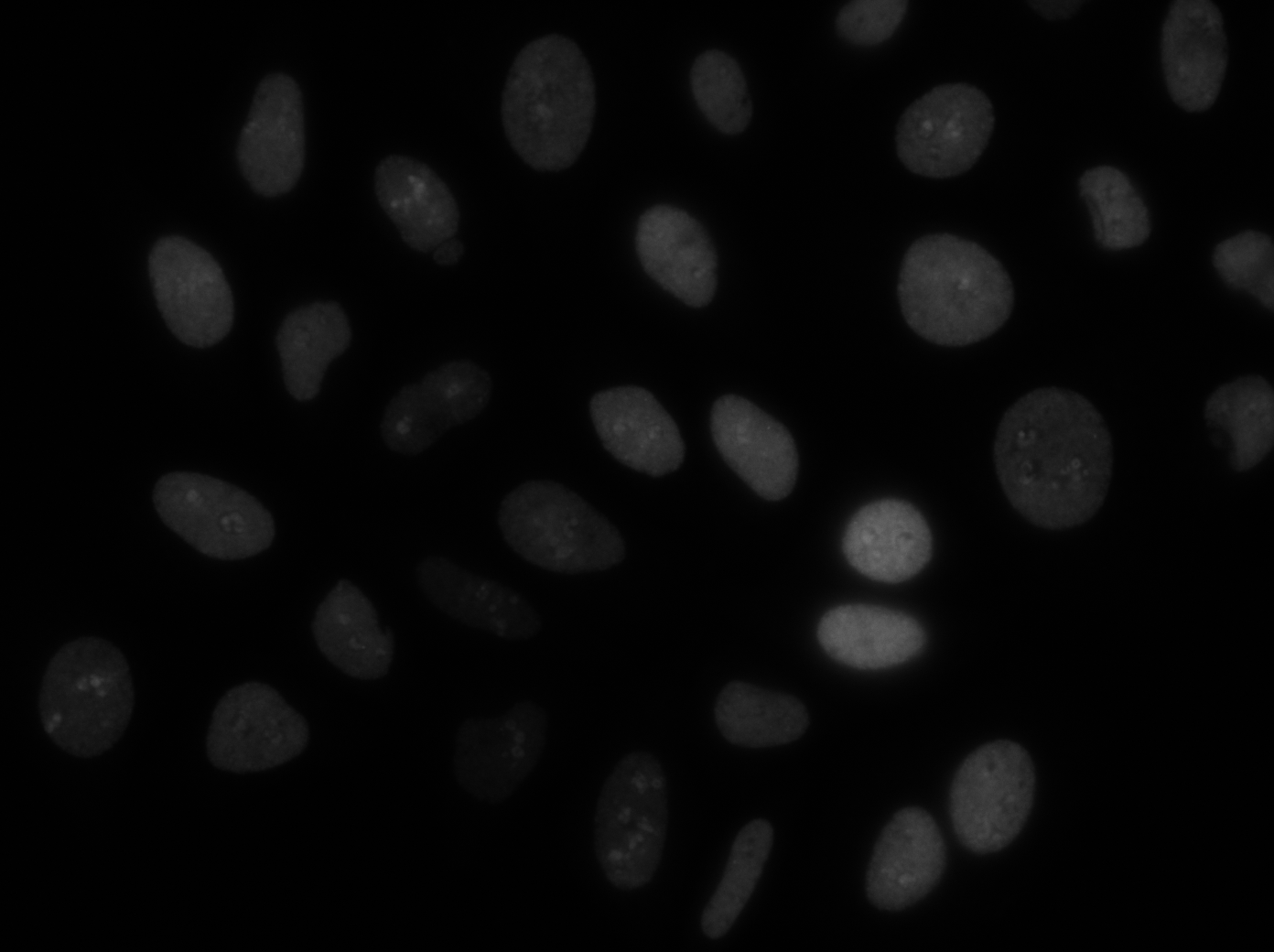

Supplement: Supplementary file 8 — Source Data [file 41467_2021_24153_MOESM8_ESM.zip › RawData/Supplementary Figures/FigS3/b/IF/siHIRA1_6h_2_w2TX.TIF]

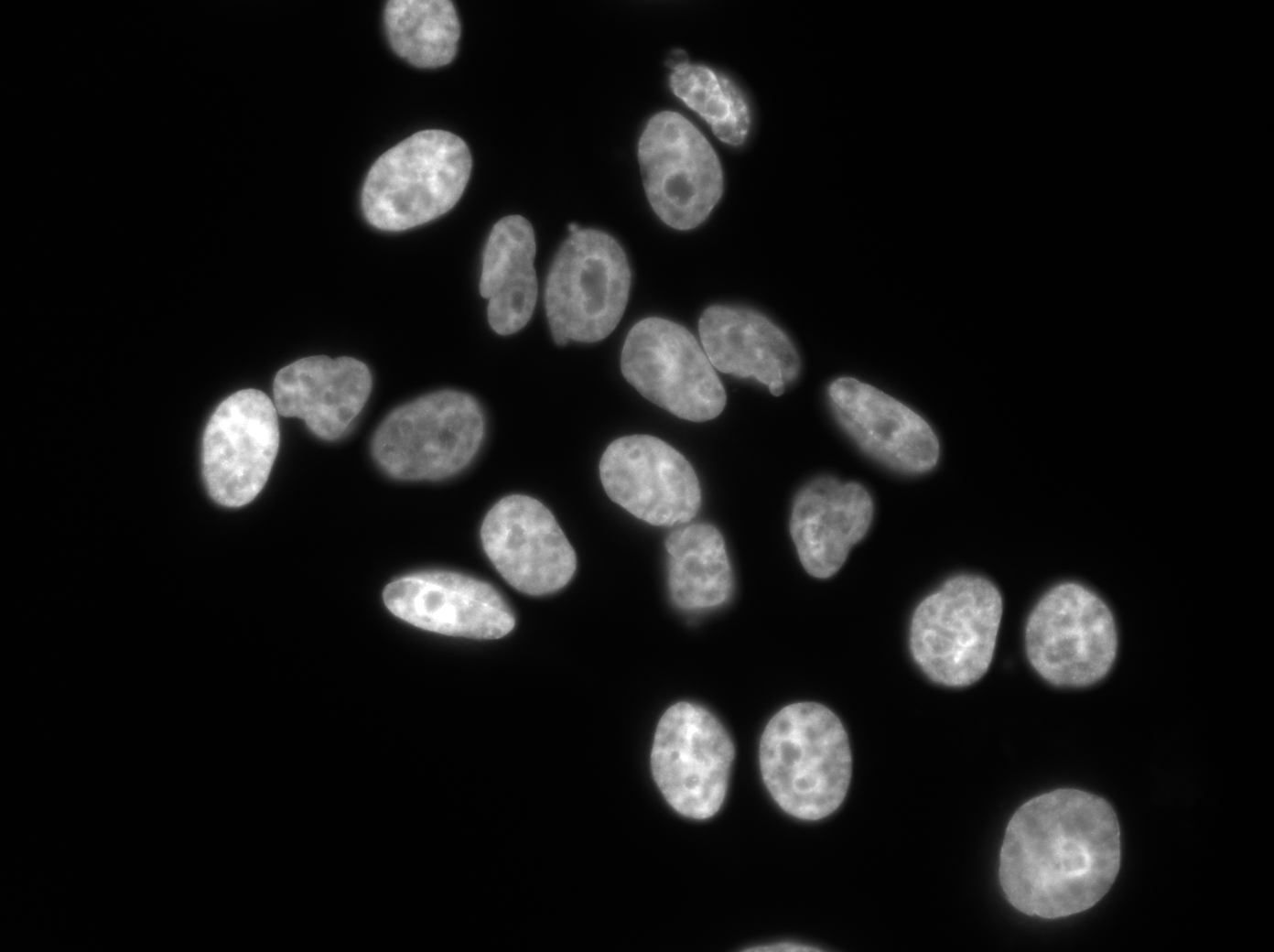

Supplement: Supplementary file 8 — Source Data [file 41467_2021_24153_MOESM8_ESM.zip › RawData/Supplementary Figures/FigS3/b/IF/siHIRA1_ut_9_w1DAPI.TIF]

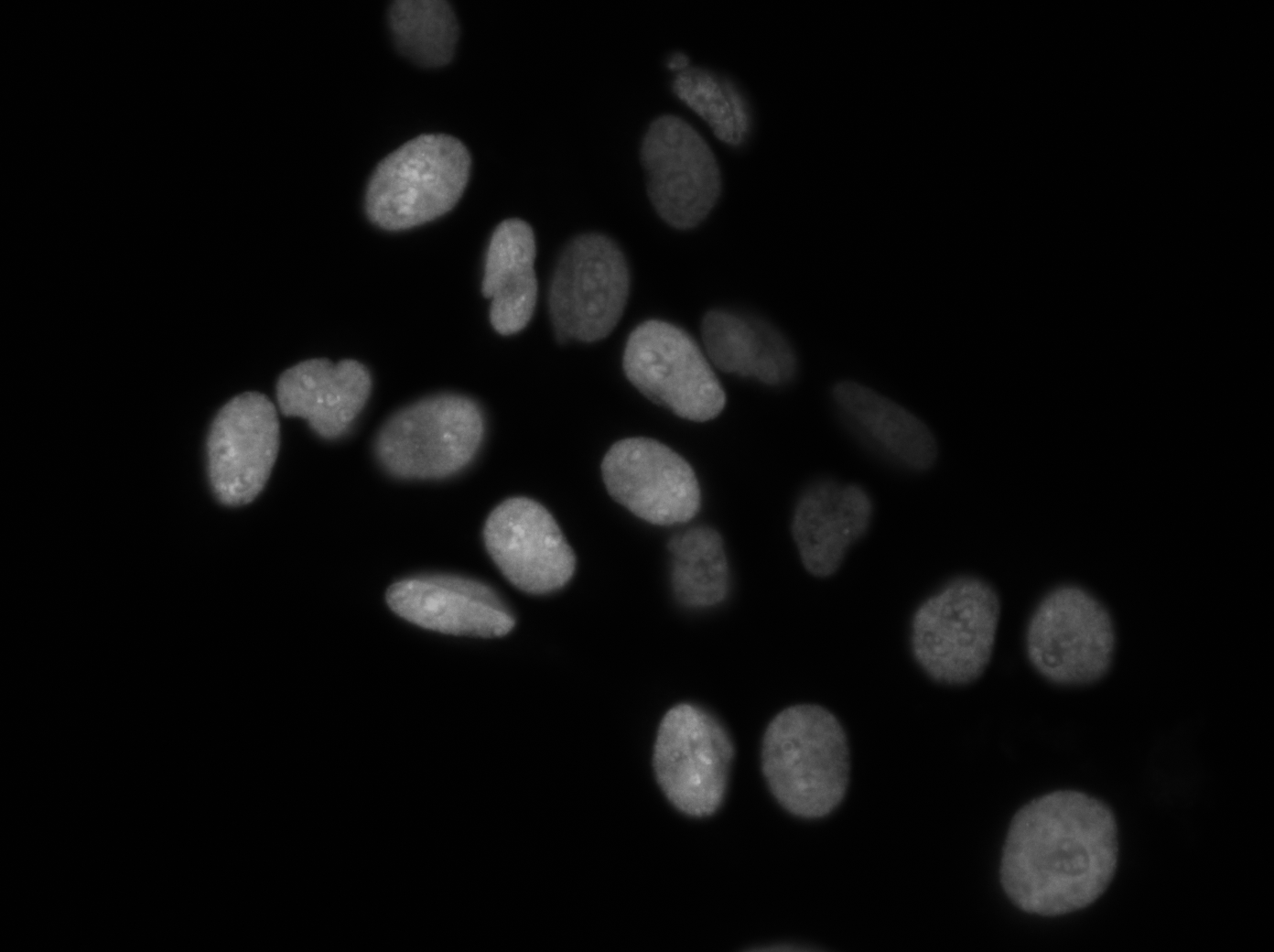

Supplement: Supplementary file 8 — Source Data [file 41467_2021_24153_MOESM8_ESM.zip › RawData/Supplementary Figures/FigS3/b/IF/siHIRA1_ut_9_w2TX.TIF]

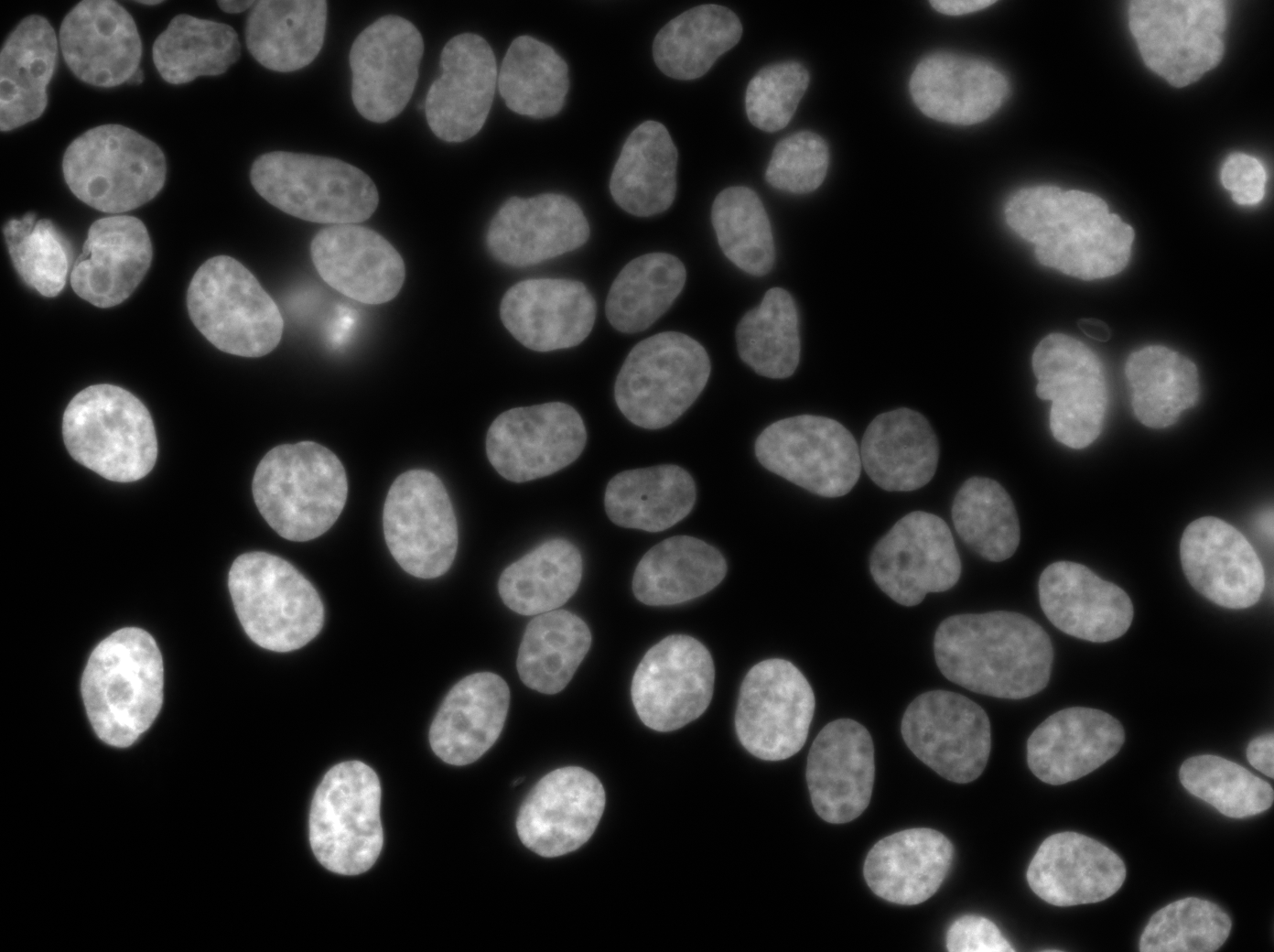

Supplement: Supplementary file 8 — Source Data [file 41467_2021_24153_MOESM8_ESM.zip › RawData/Supplementary Figures/FigS3/b/IF/siHIRA2_0h_7_w1DAPI.TIF]

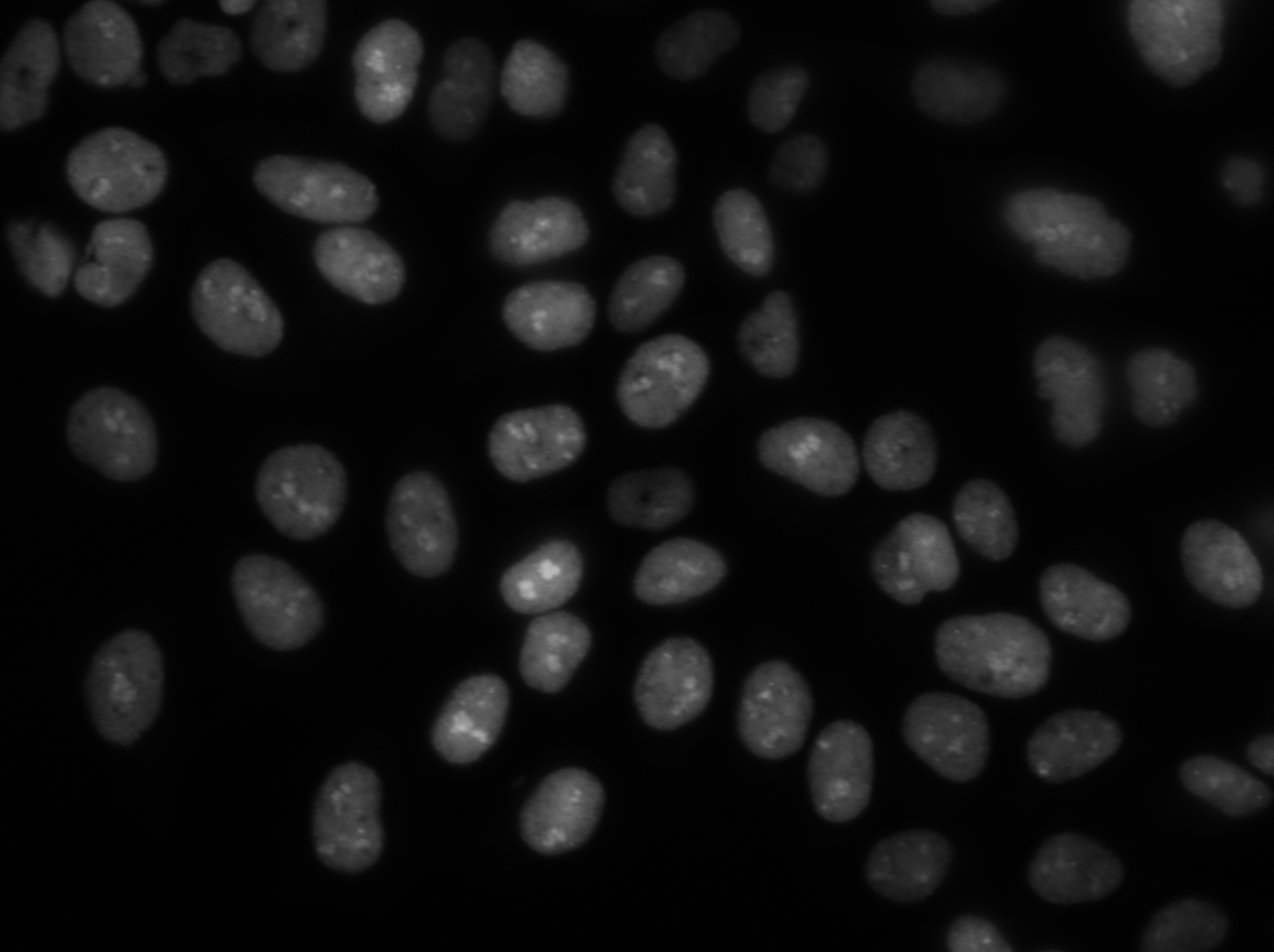

Supplement: Supplementary file 8 — Source Data [file 41467_2021_24153_MOESM8_ESM.zip › RawData/Supplementary Figures/FigS3/b/IF/siHIRA2_0h_7_w2TX.TIF]

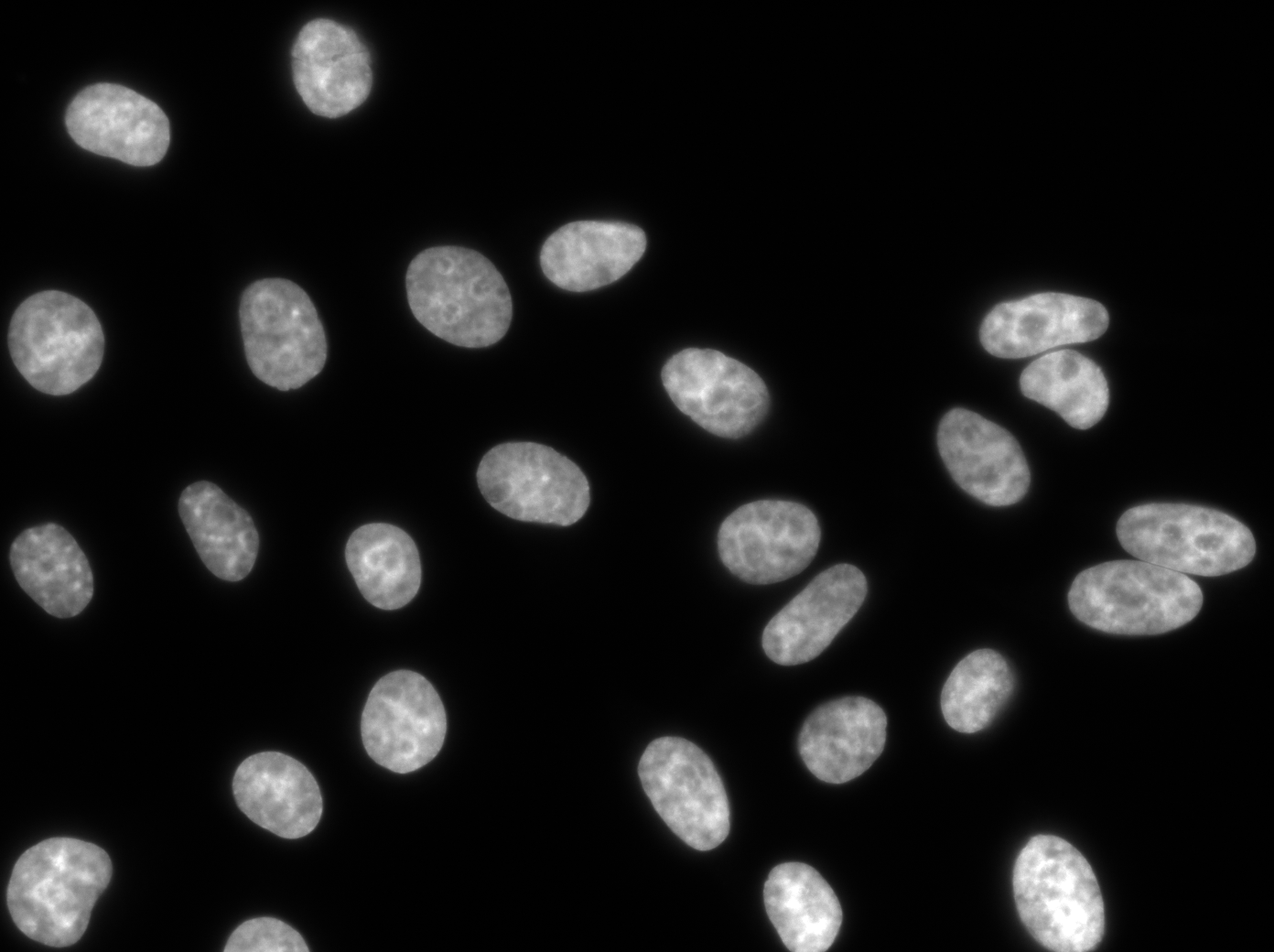

Supplement: Supplementary file 8 — Source Data [file 41467_2021_24153_MOESM8_ESM.zip › RawData/Supplementary Figures/FigS3/b/IF/siHIRA2_24h_2_w1DAPI.TIF]

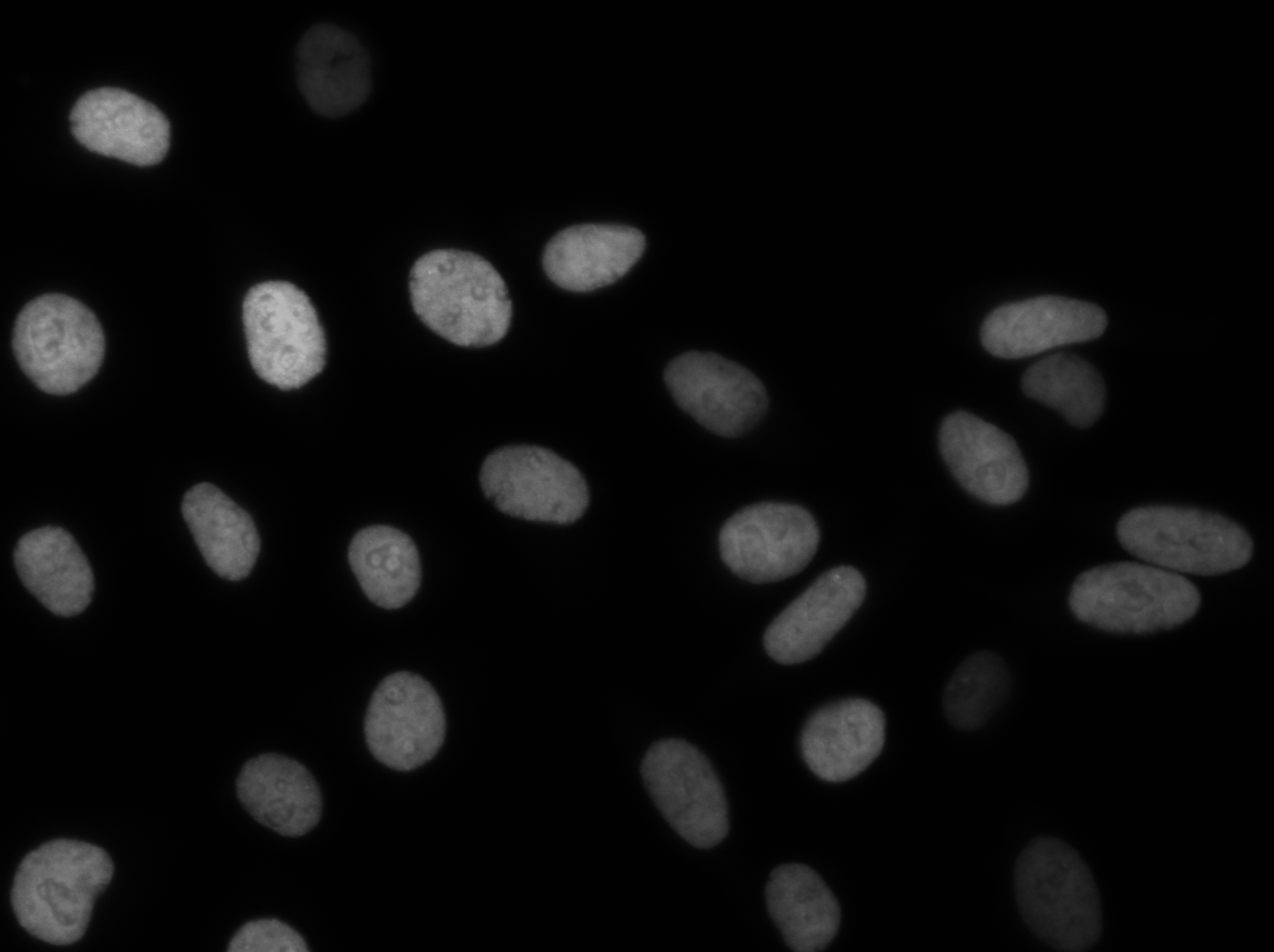

Supplement: Supplementary file 8 — Source Data [file 41467_2021_24153_MOESM8_ESM.zip › RawData/Supplementary Figures/FigS3/b/IF/siHIRA2_24h_2_w2TX.TIF]

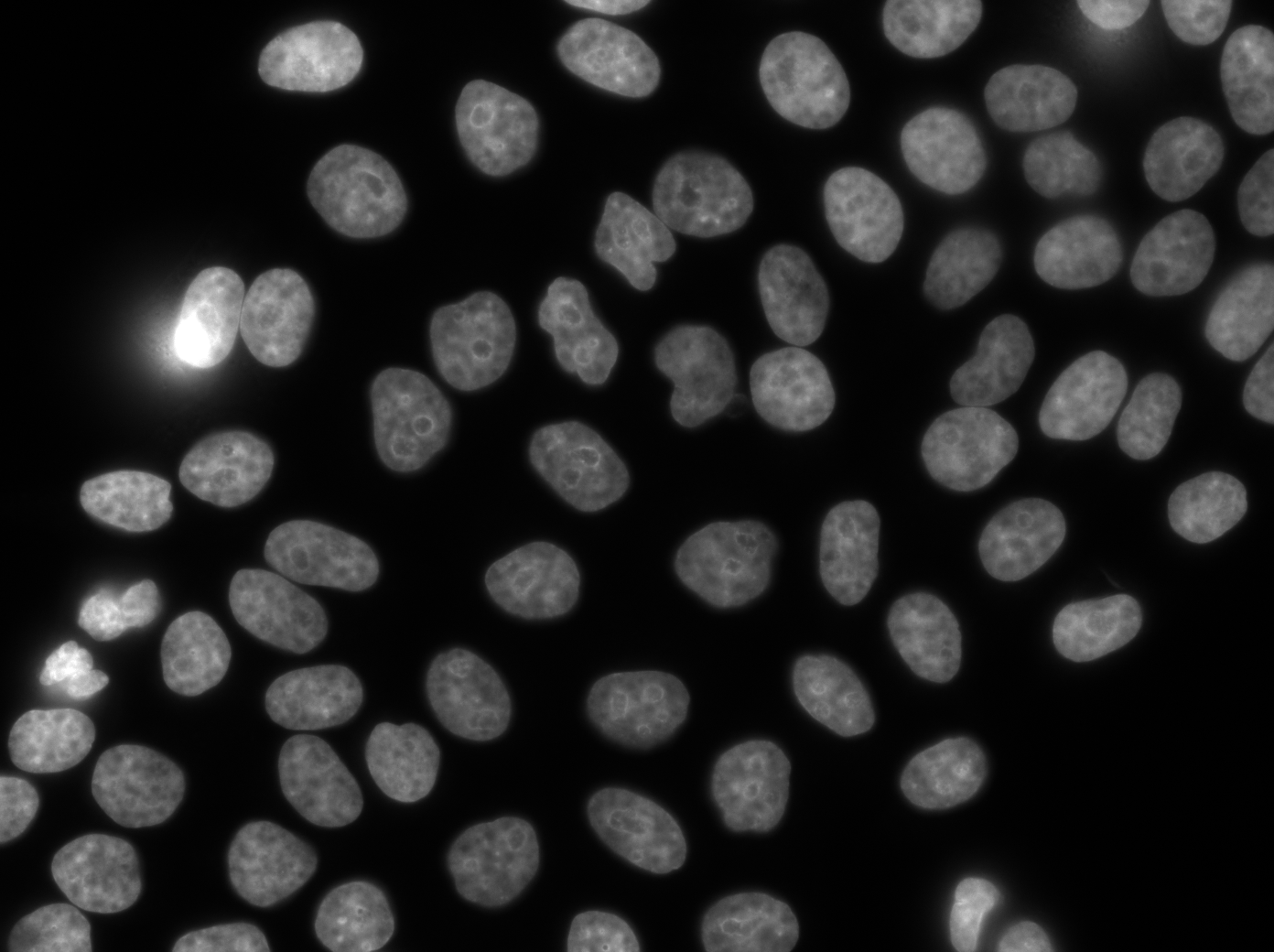

Supplement: Supplementary file 8 — Source Data [file 41467_2021_24153_MOESM8_ESM.zip › RawData/Supplementary Figures/FigS3/b/IF/siHIRA2_2h_4_w1DAPI.TIF]

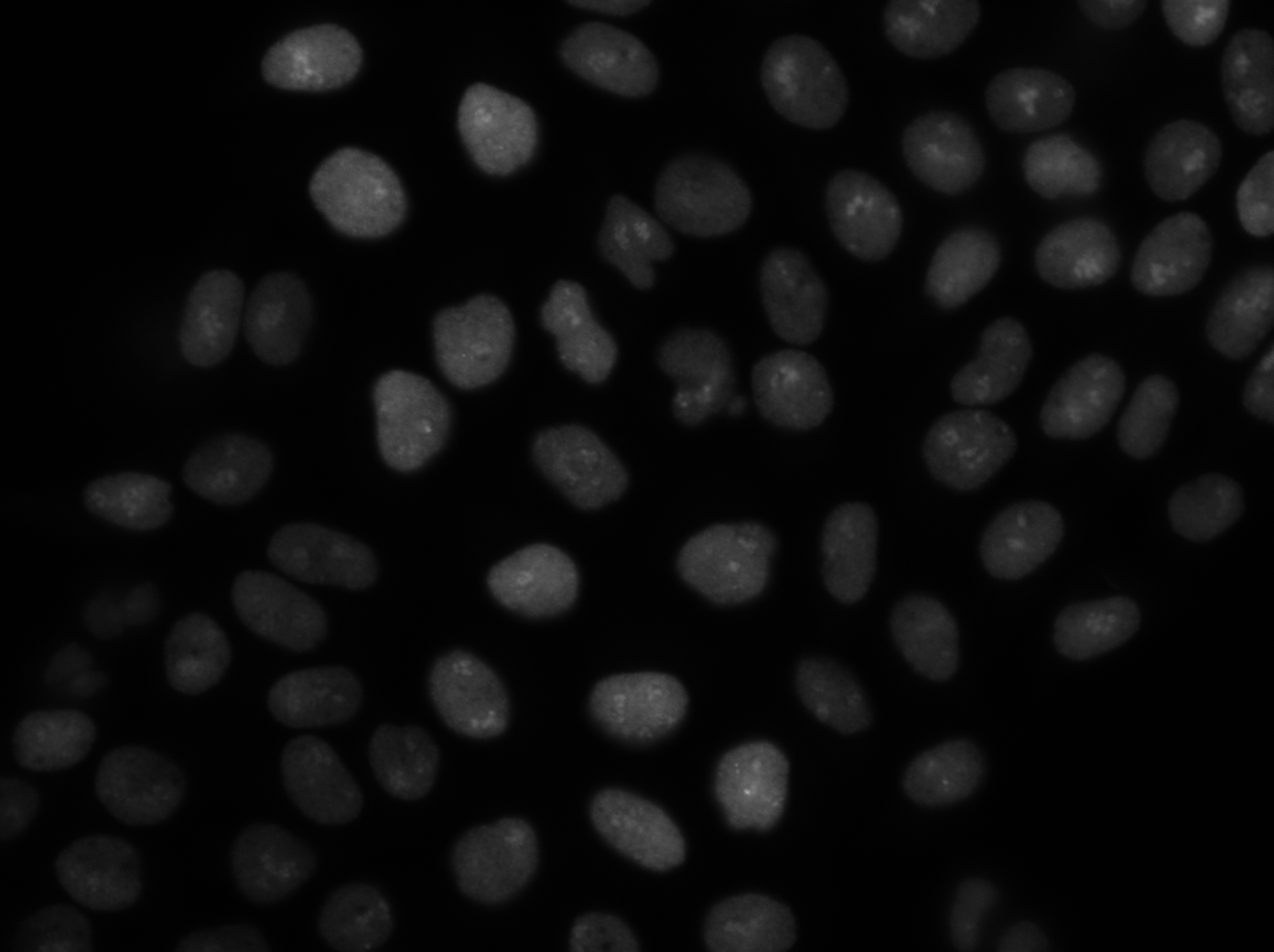

Supplement: Supplementary file 8 — Source Data [file 41467_2021_24153_MOESM8_ESM.zip › RawData/Supplementary Figures/FigS3/b/IF/siHIRA2_2h_4_w2TX.TIF]

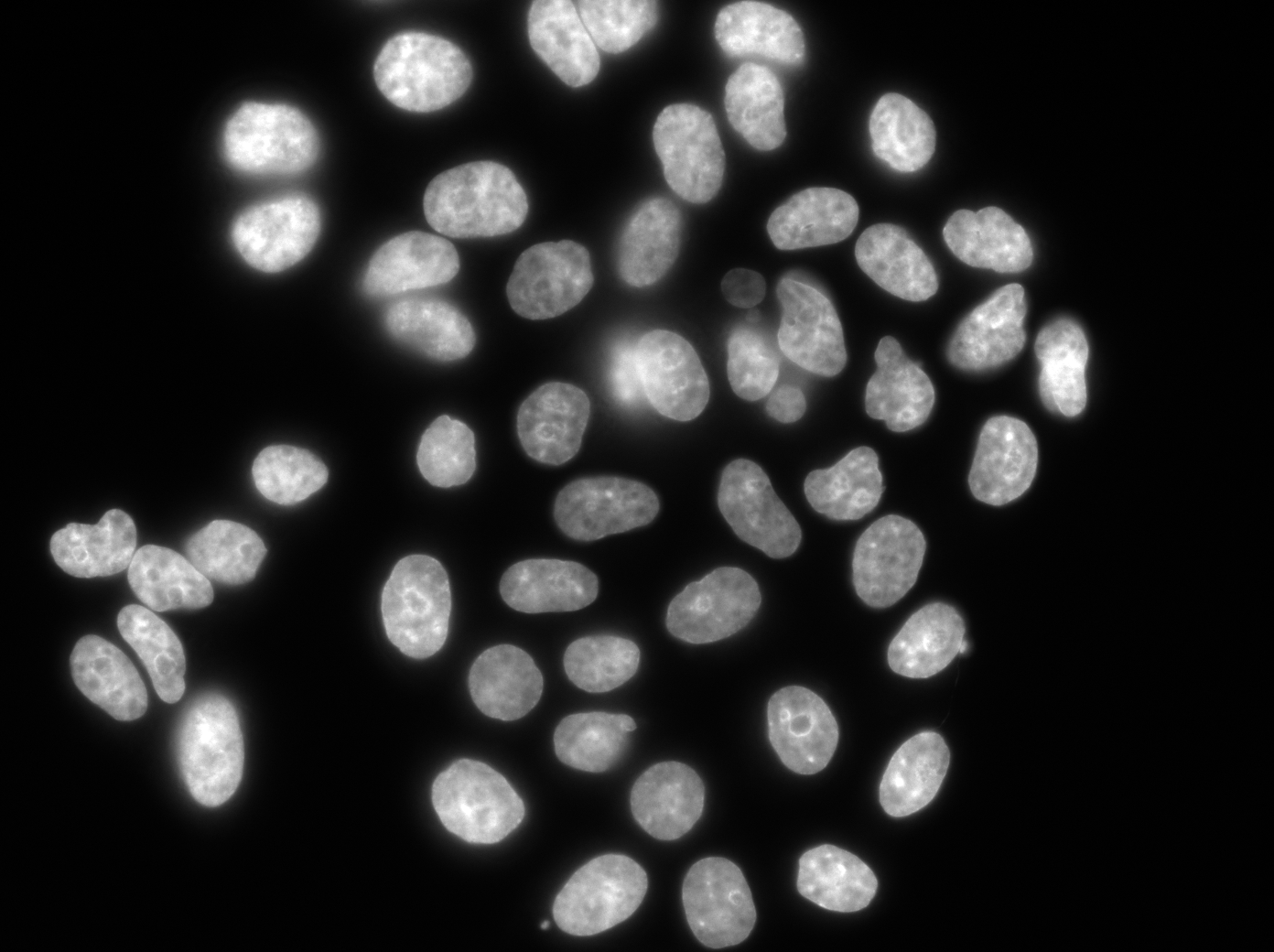

Supplement: Supplementary file 8 — Source Data [file 41467_2021_24153_MOESM8_ESM.zip › RawData/Supplementary Figures/FigS3/b/IF/siHIRA2_6h_6_w1DAPI.TIF]

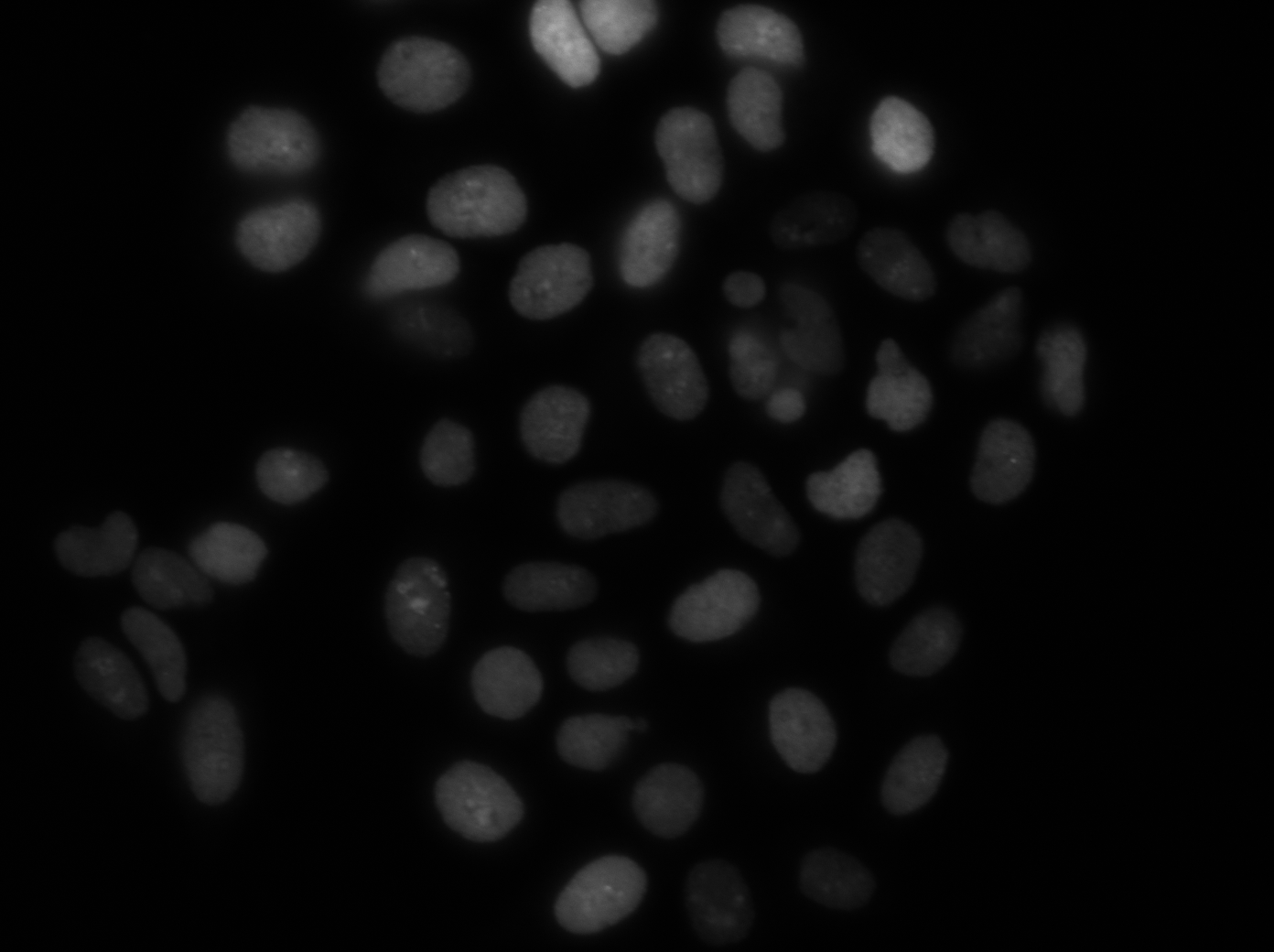

Supplement: Supplementary file 8 — Source Data [file 41467_2021_24153_MOESM8_ESM.zip › RawData/Supplementary Figures/FigS3/b/IF/siHIRA2_6h_6_w2TX.TIF]

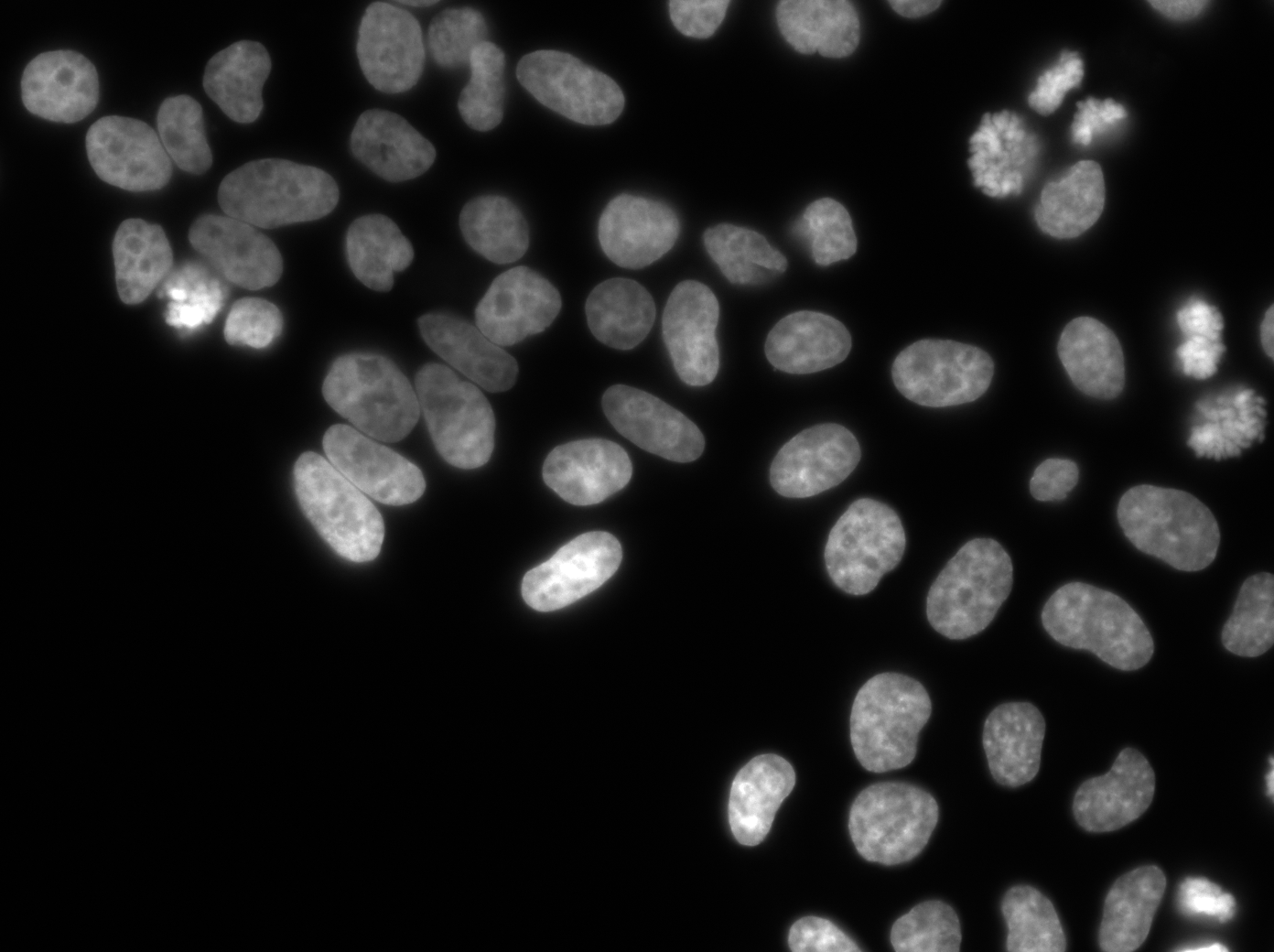

Supplement: Supplementary file 8 — Source Data [file 41467_2021_24153_MOESM8_ESM.zip › RawData/Supplementary Figures/FigS3/b/IF/siHIRA2_ut_6_w1DAPI.TIF]

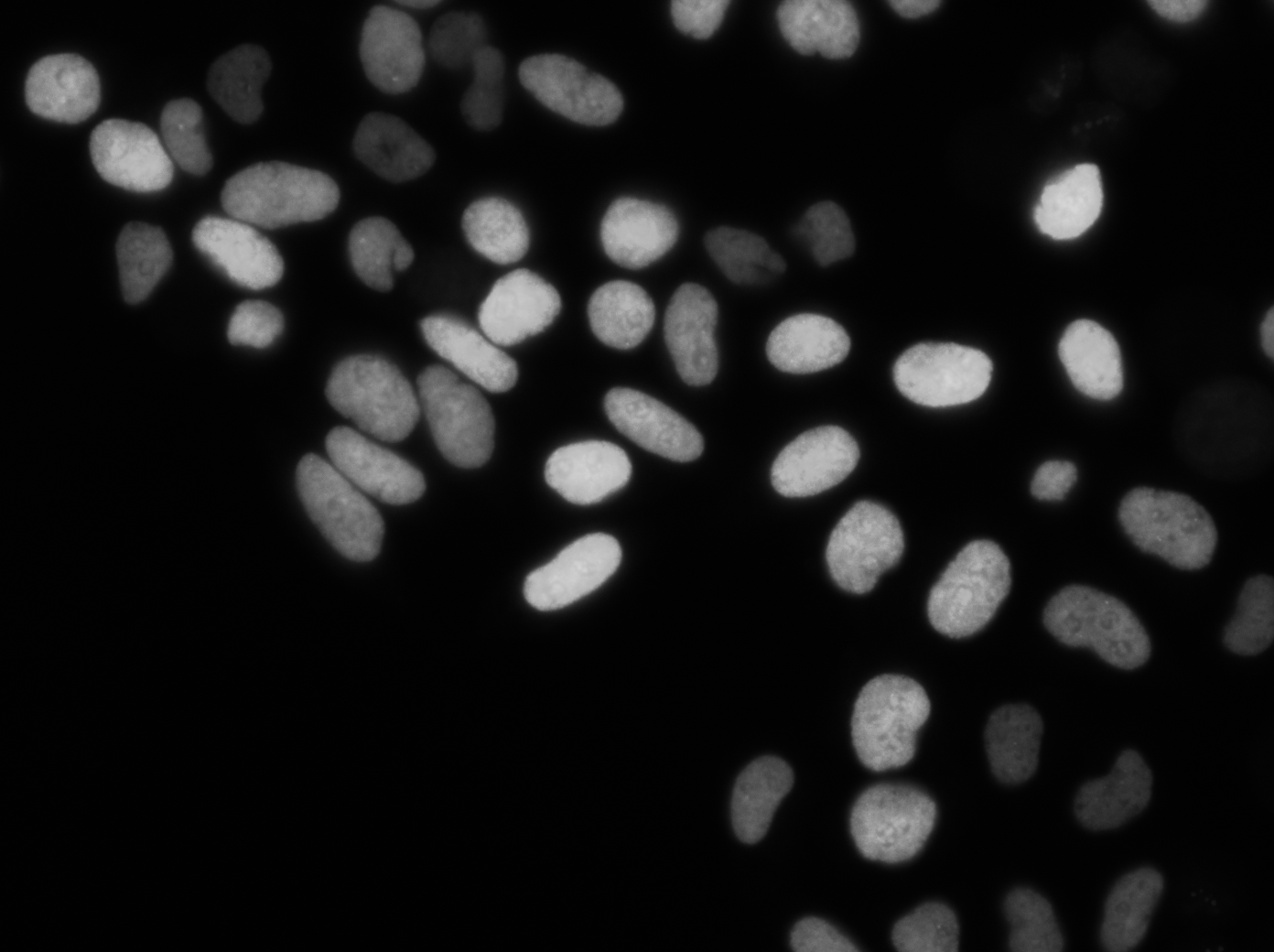

Supplement: Supplementary file 8 — Source Data [file 41467_2021_24153_MOESM8_ESM.zip › RawData/Supplementary Figures/FigS3/b/IF/siHIRA2_ut_6_w2TX.TIF]

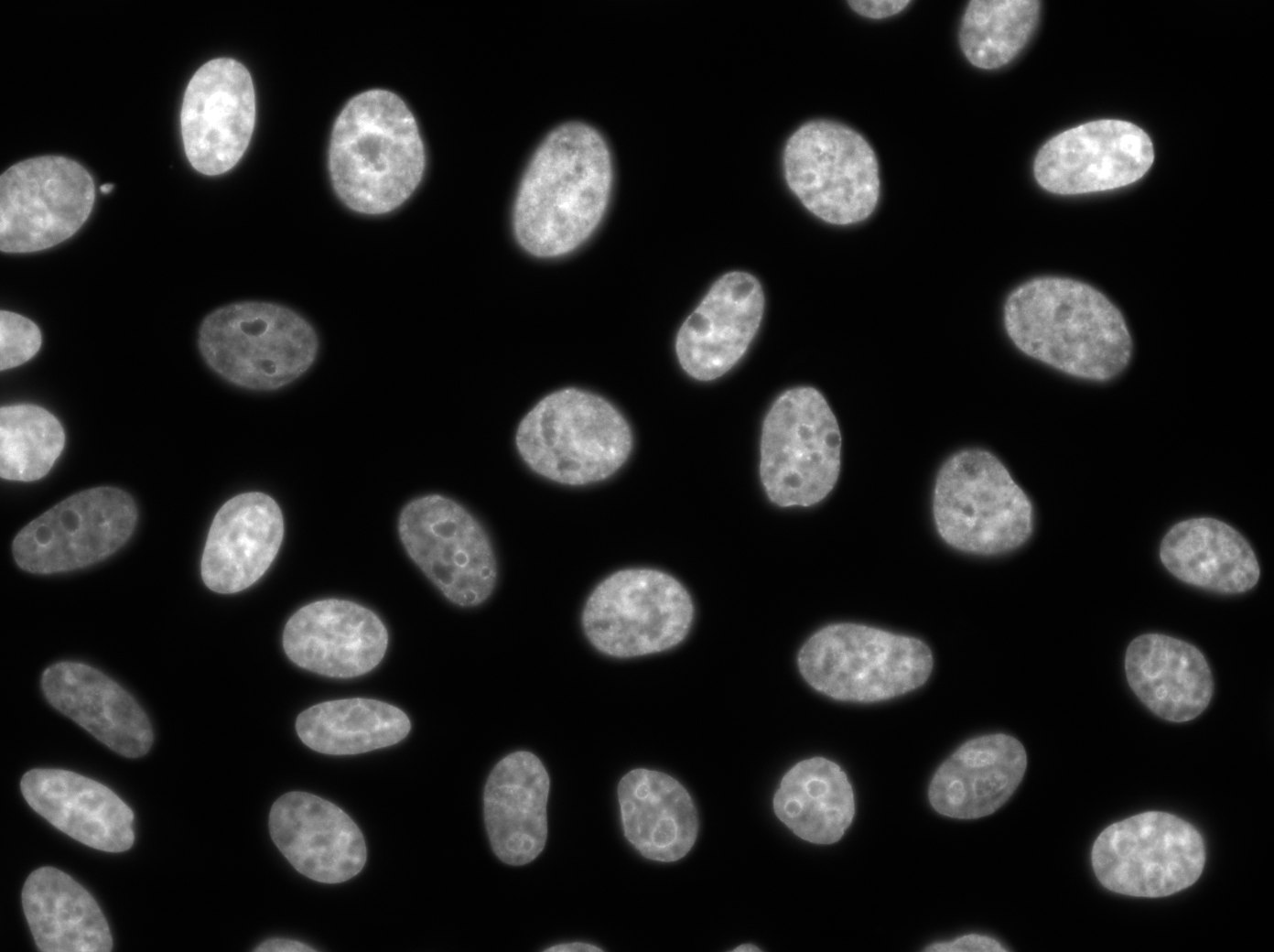

Supplement: Supplementary file 8 — Source Data [file 41467_2021_24153_MOESM8_ESM.zip › RawData/Supplementary Figures/FigS3/b/IF/siluc_0h_3_w1DAPI.TIF]

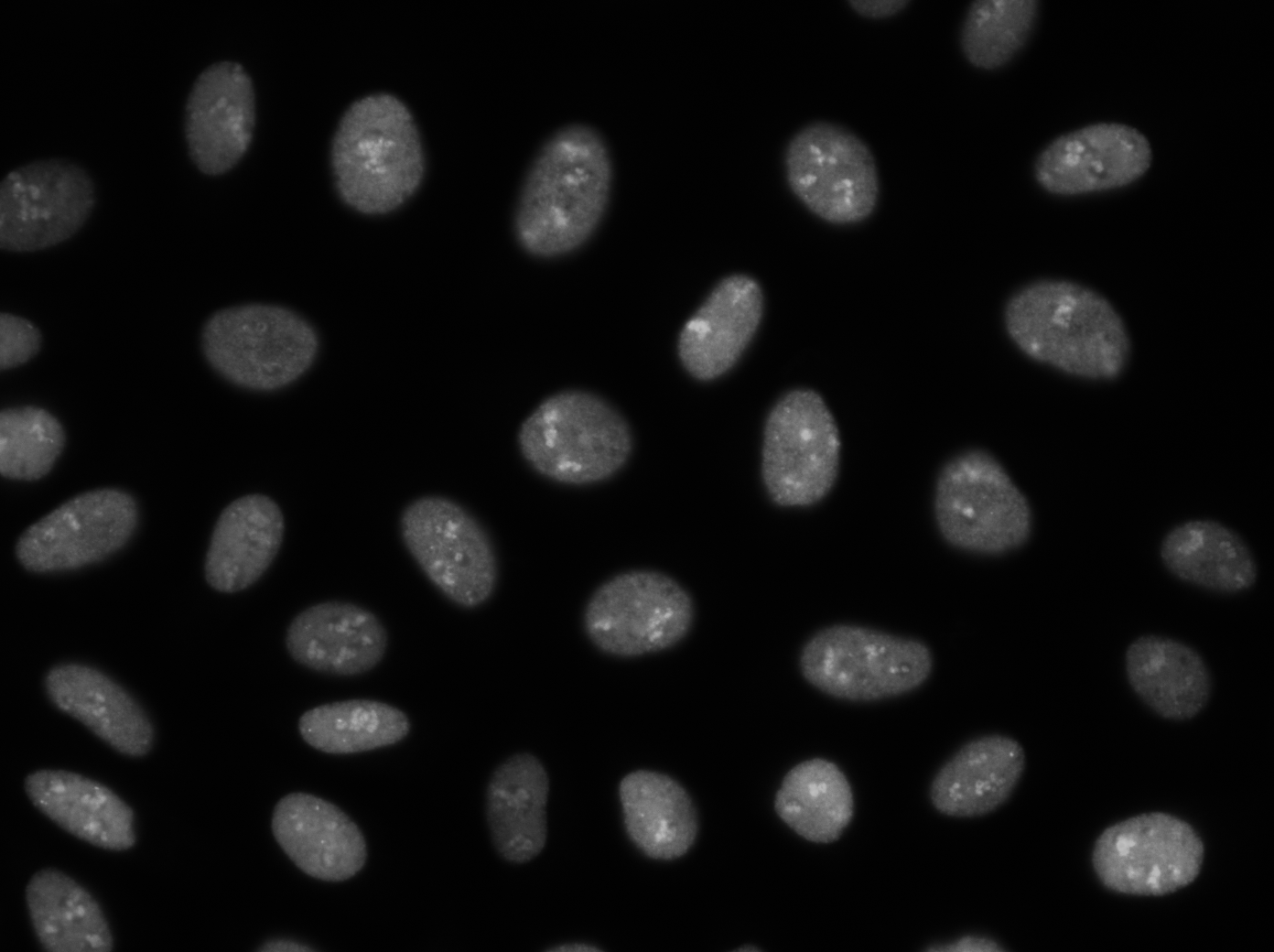

Supplement: Supplementary file 8 — Source Data [file 41467_2021_24153_MOESM8_ESM.zip › RawData/Supplementary Figures/FigS3/b/IF/siluc_0h_3_w2TX.TIF]

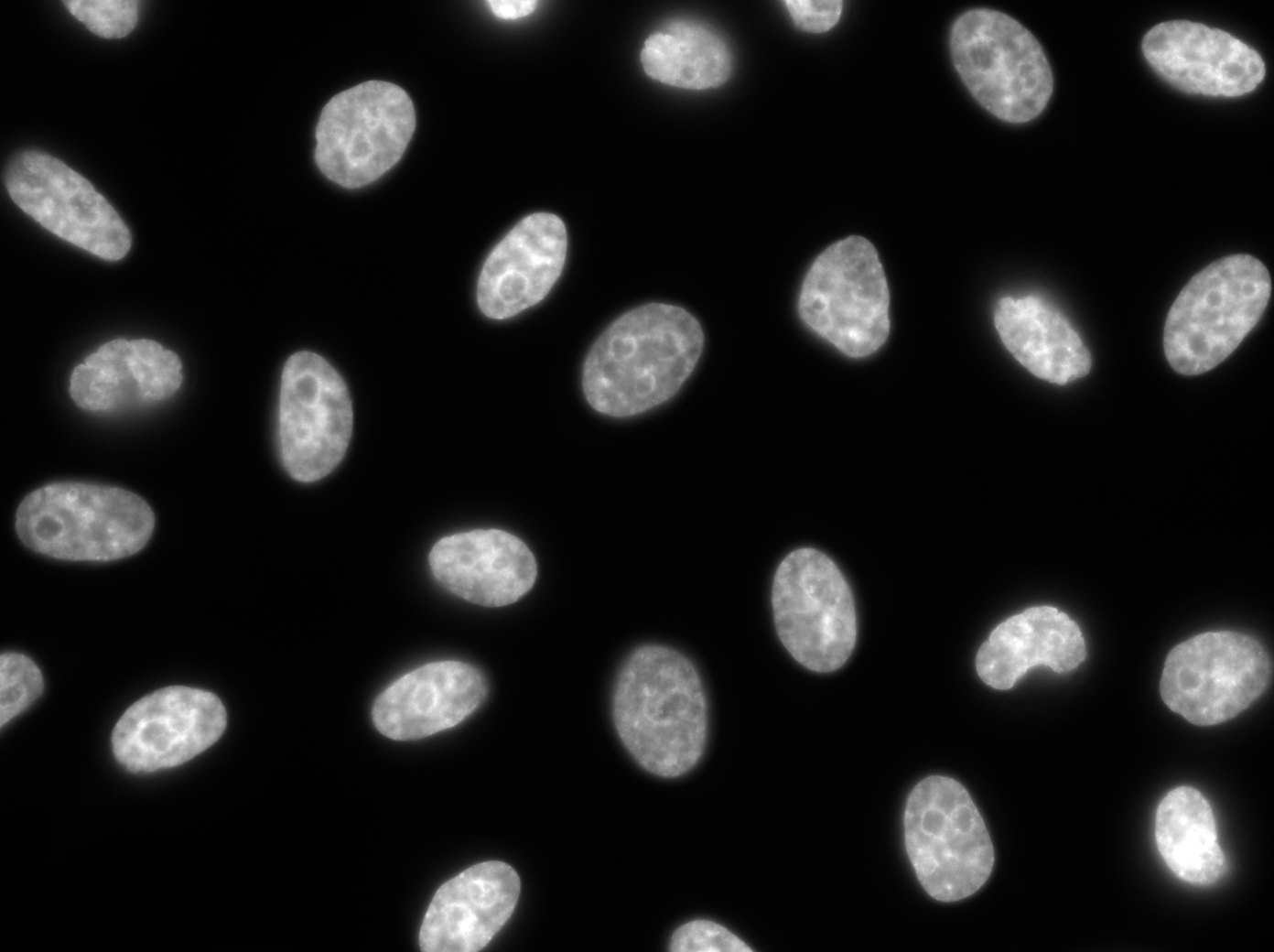

Supplement: Supplementary file 8 — Source Data [file 41467_2021_24153_MOESM8_ESM.zip › RawData/Supplementary Figures/FigS3/b/IF/siluc_24h_5_w1DAPI.TIF]

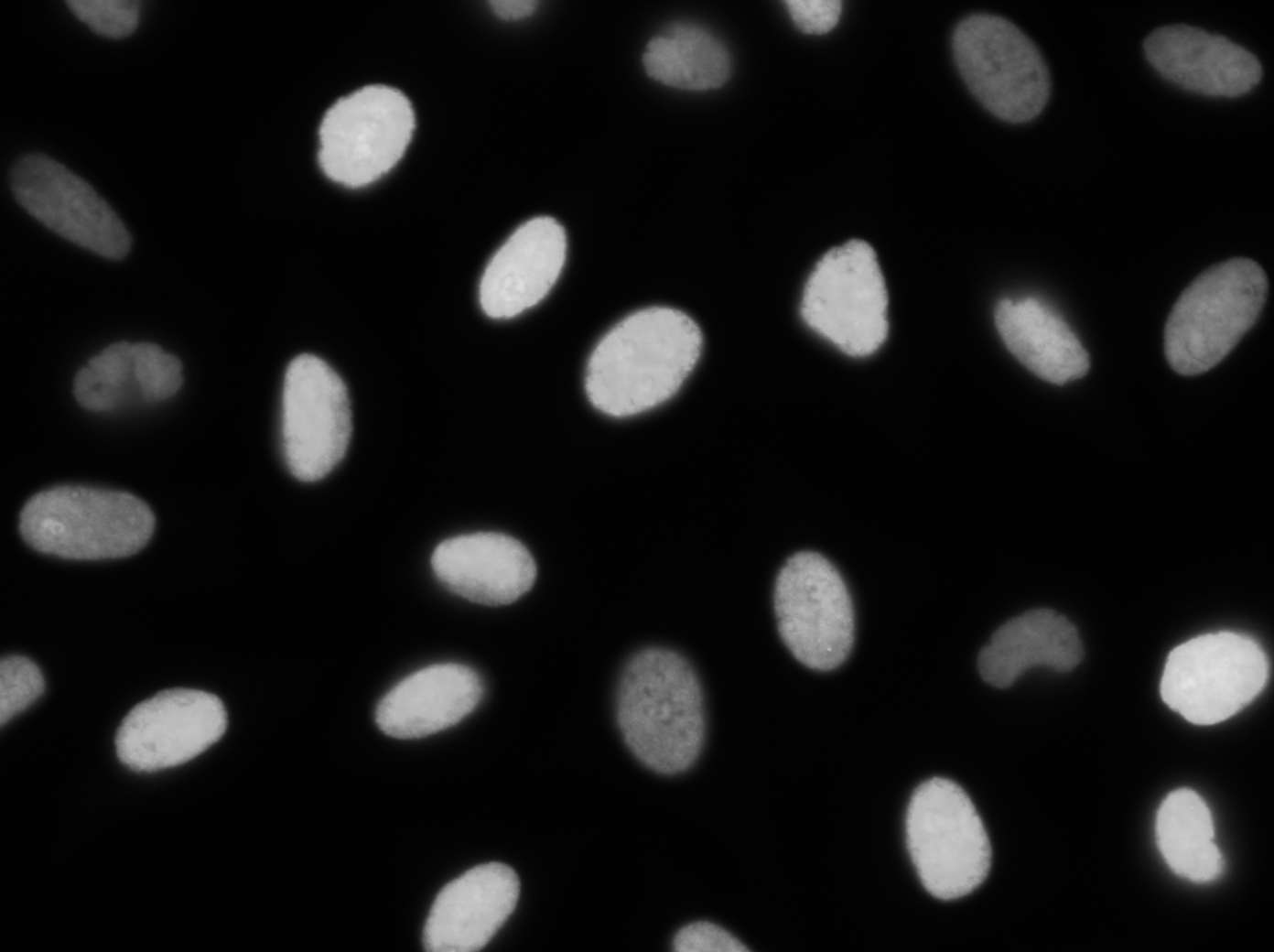

Supplement: Supplementary file 8 — Source Data [file 41467_2021_24153_MOESM8_ESM.zip › RawData/Supplementary Figures/FigS3/b/IF/siluc_24h_5_w2TX.TIF]

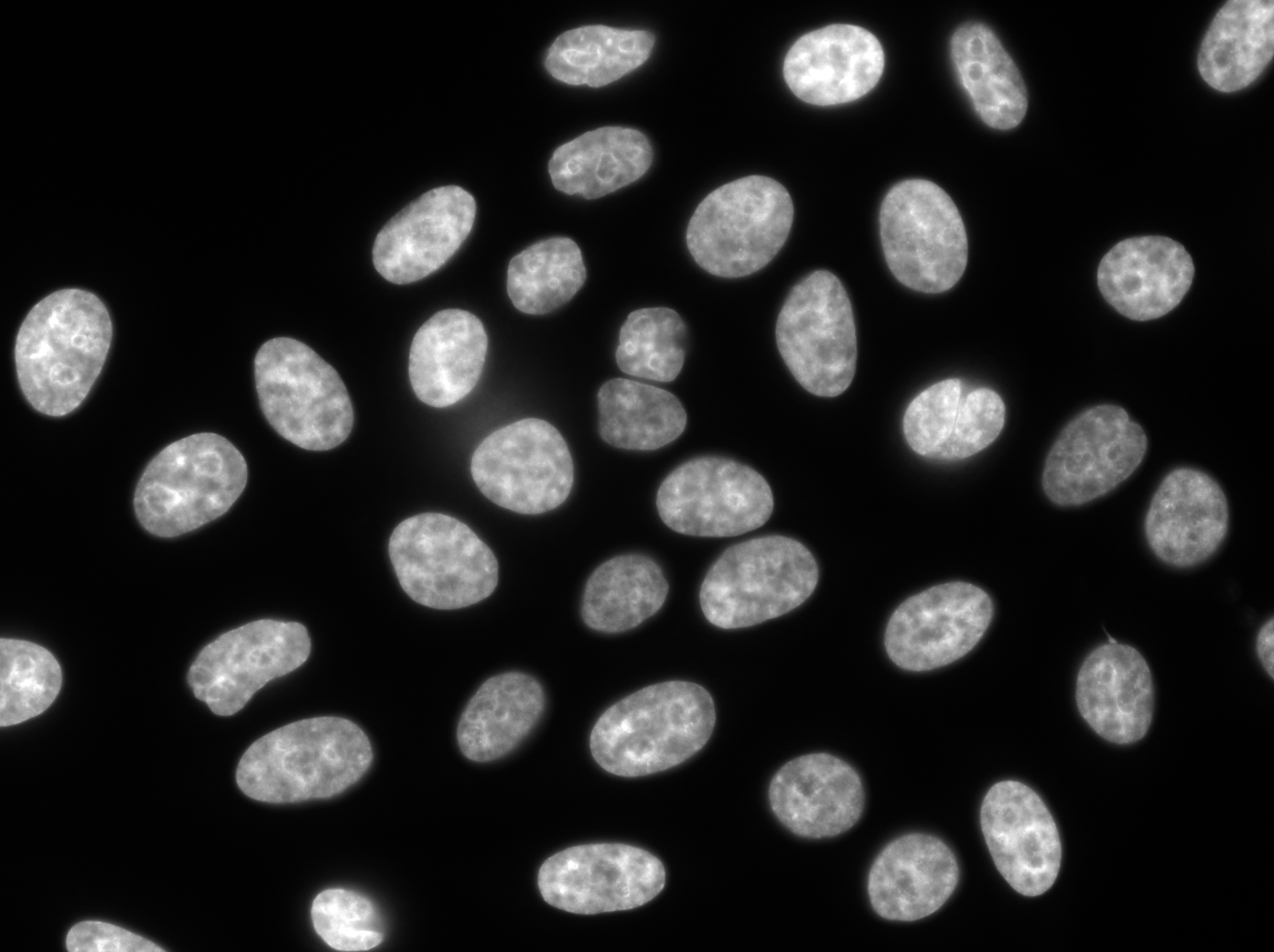

Supplement: Supplementary file 8 — Source Data [file 41467_2021_24153_MOESM8_ESM.zip › RawData/Supplementary Figures/FigS3/b/IF/siluc_2h_1_w1DAPI.TIF]

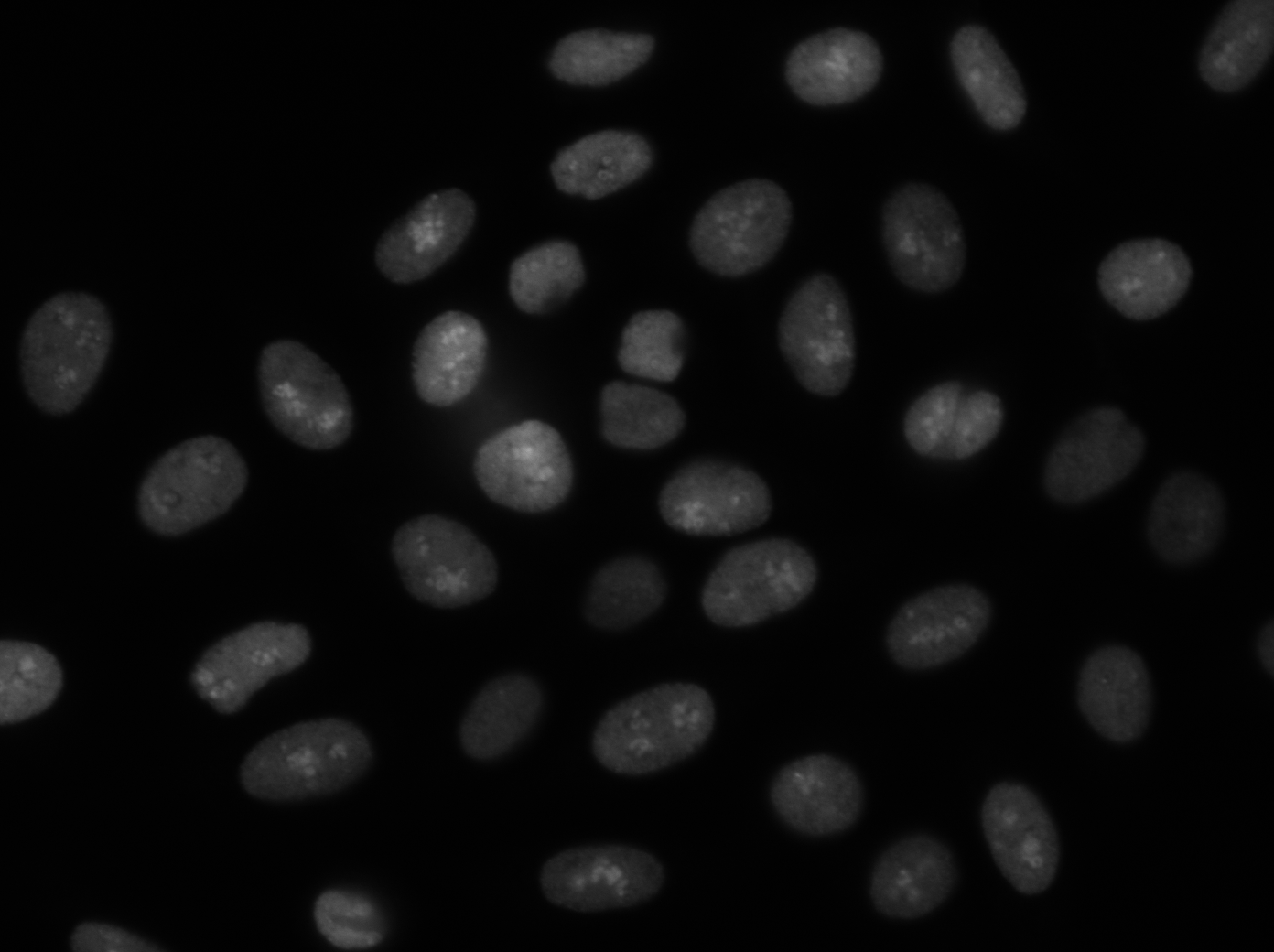

Supplement: Supplementary file 8 — Source Data [file 41467_2021_24153_MOESM8_ESM.zip › RawData/Supplementary Figures/FigS3/b/IF/siluc_2h_1_w2TX.TIF]

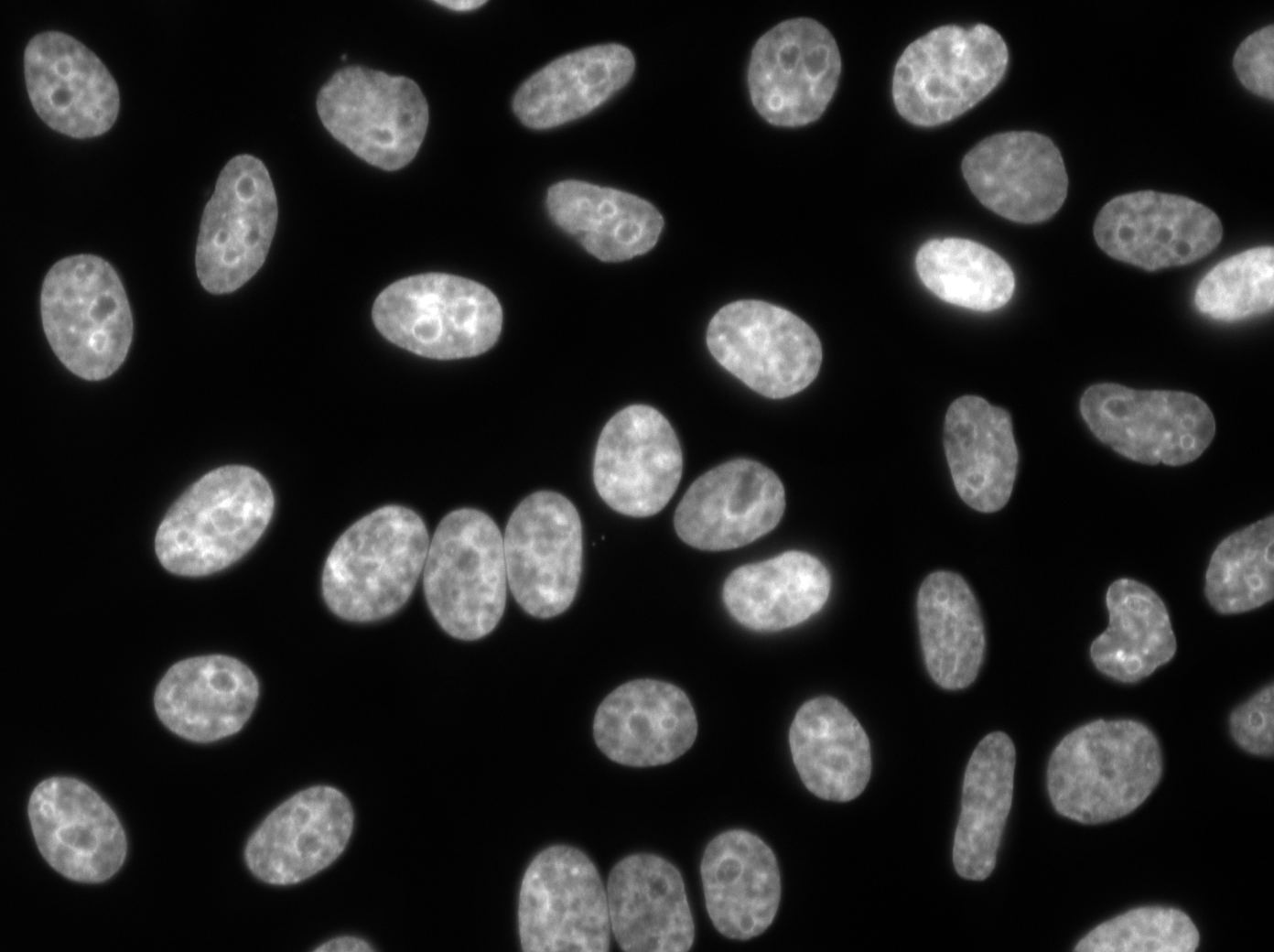

Supplement: Supplementary file 8 — Source Data [file 41467_2021_24153_MOESM8_ESM.zip › RawData/Supplementary Figures/FigS3/b/IF/siluc_6h_5_w1DAPI.TIF]

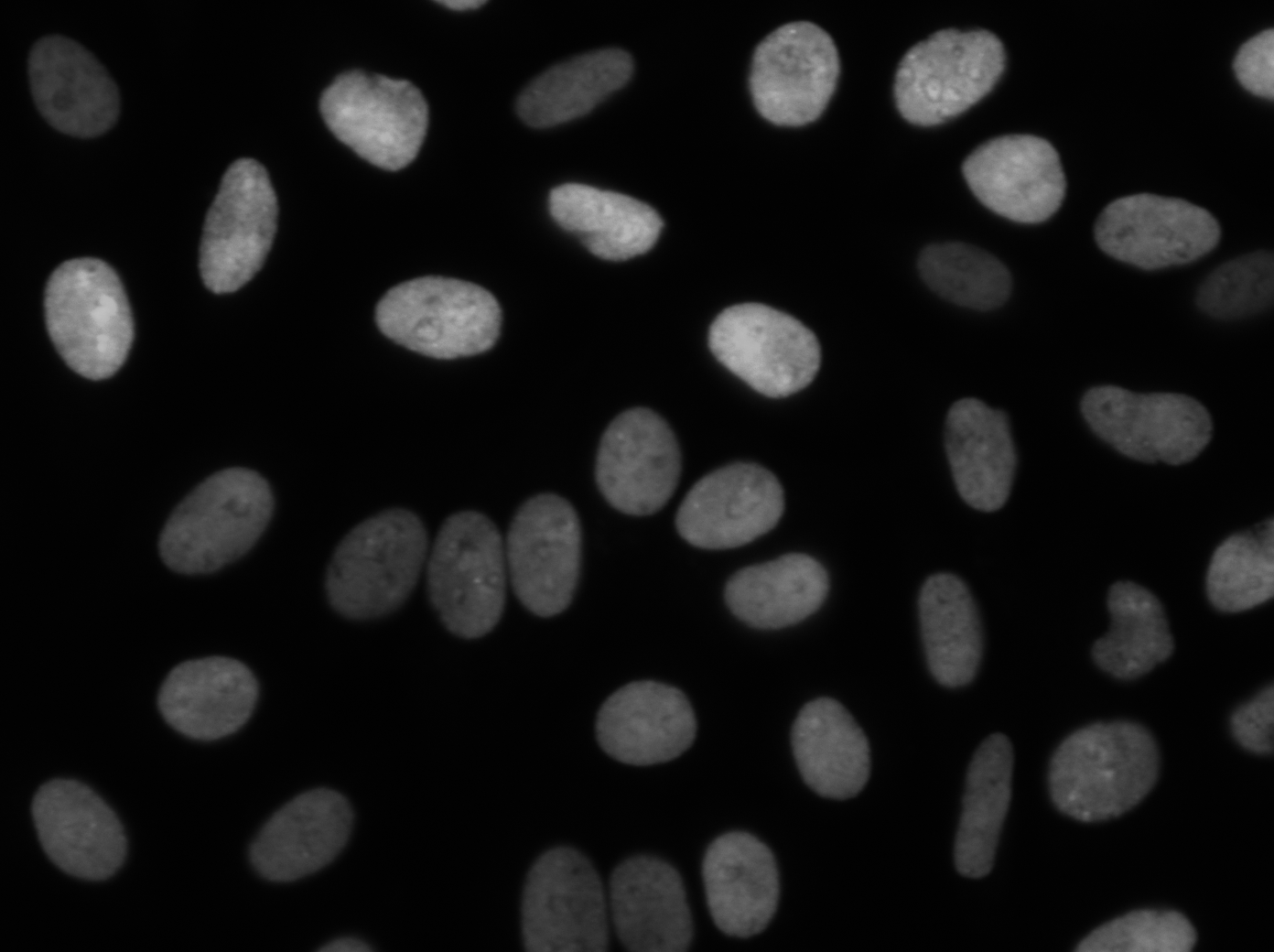

Supplement: Supplementary file 8 — Source Data [file 41467_2021_24153_MOESM8_ESM.zip › RawData/Supplementary Figures/FigS3/b/IF/siluc_6h_5_w2TX.TIF]

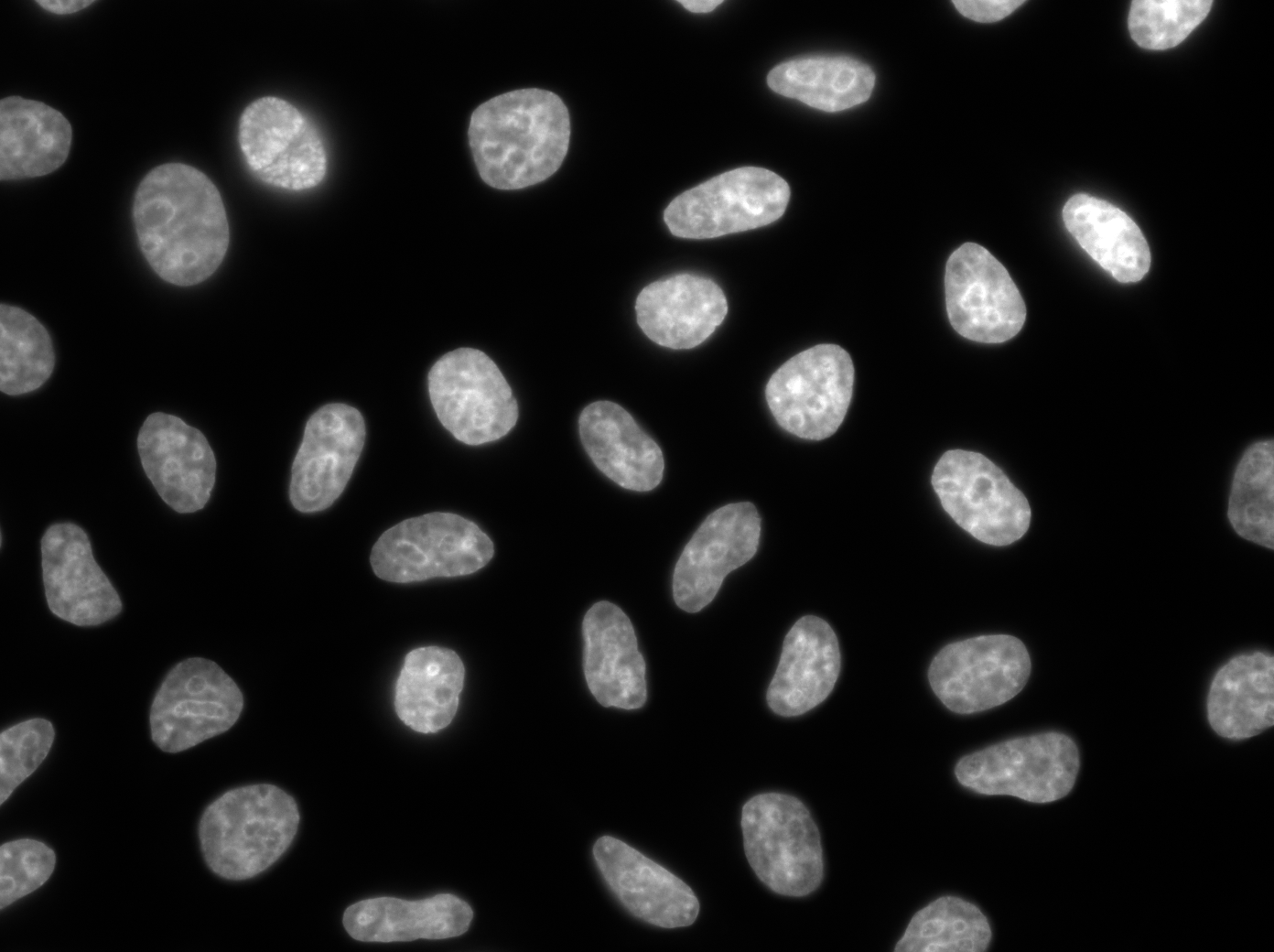

Supplement: Supplementary file 8 — Source Data [file 41467_2021_24153_MOESM8_ESM.zip › RawData/Supplementary Figures/FigS3/b/IF/siluc_ut_7_w1DAPI.TIF]

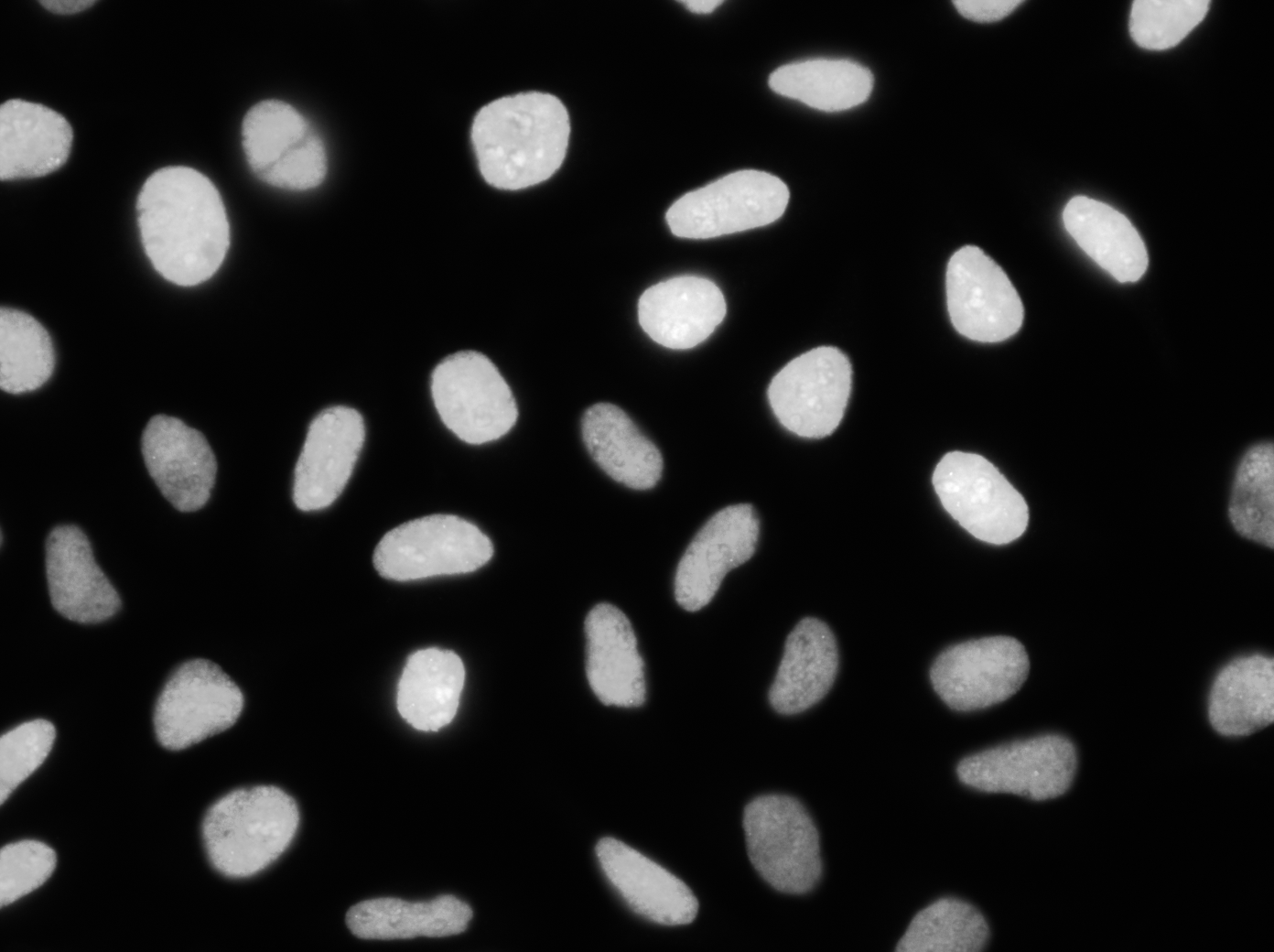

Supplement: Supplementary file 8 — Source Data [file 41467_2021_24153_MOESM8_ESM.zip › RawData/Supplementary Figures/FigS3/b/IF/siluc_ut_7_w2TX.TIF]
