# Supplementary figures and images for: Dissecting regulatory pathways for transcription recovery following DNA damage reveals a non-canonical function of the histone chaperone HIRA (part 1 of 2)
Source: Nat Commun. 2021 Jun 22;12:3835. doi: 10.1038/s41467-021-24153-1 (PMC8219801; doi:10.1038/s41467-021-24153-1)

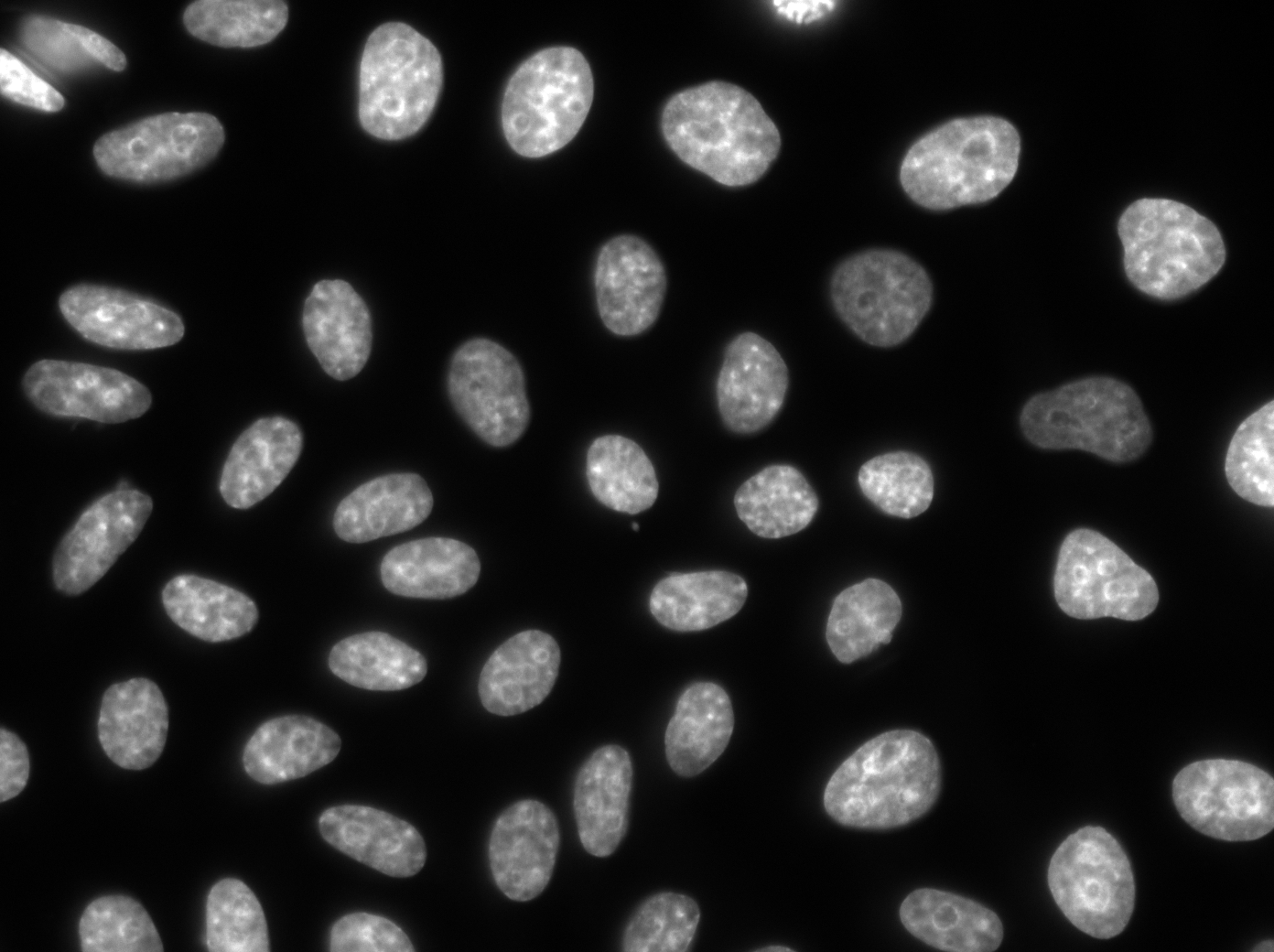

Supplement: Supplementary file 8 — Source Data [file 41467_2021_24153_MOESM8_ESM.zip › RawData/Main Figures/Fig1/a/HeLa_EU_siCABIN1_0h_05_w1DAPI.TIF]

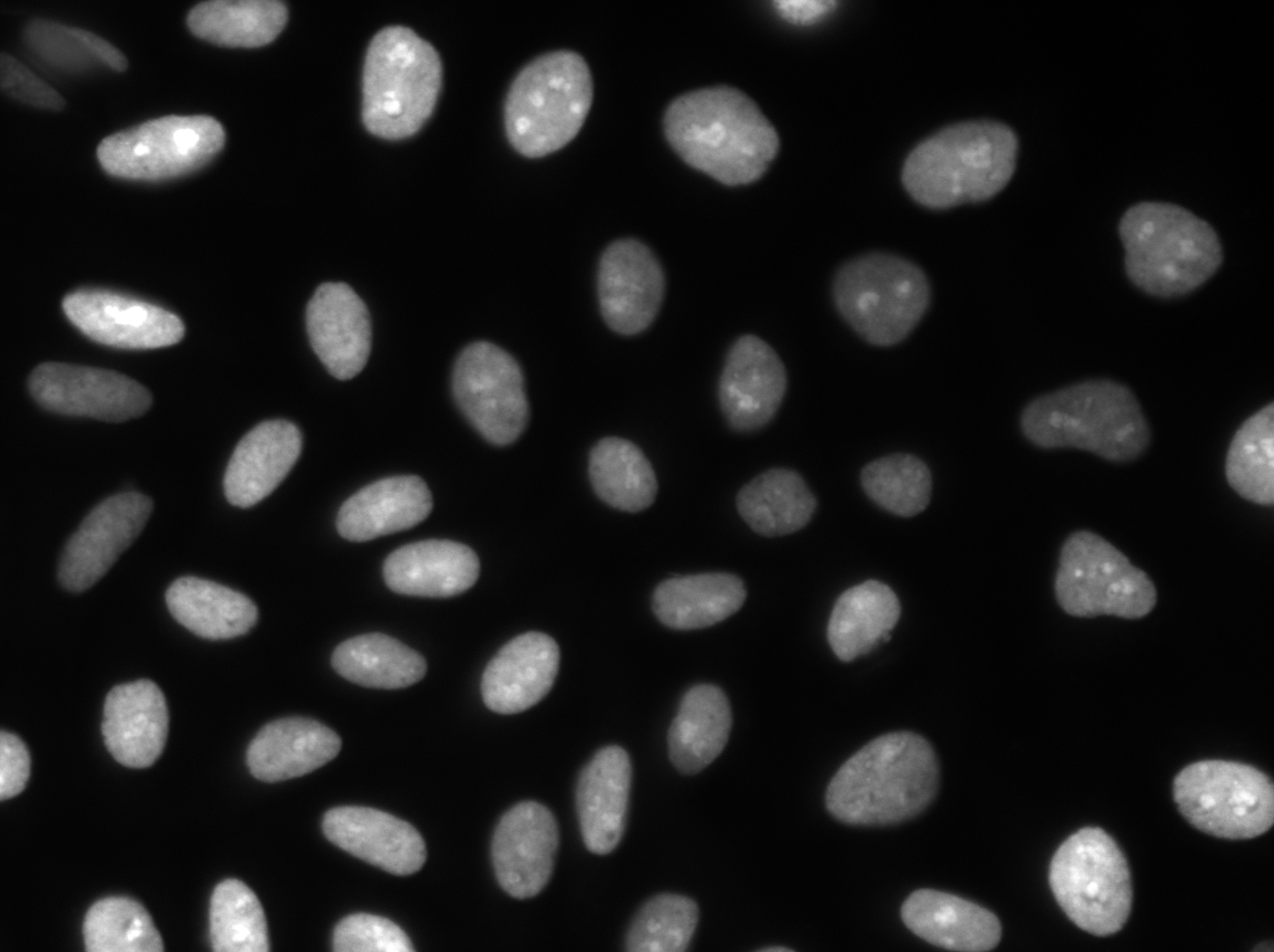

Supplement: Supplementary file 8 — Source Data [file 41467_2021_24153_MOESM8_ESM.zip › RawData/Main Figures/Fig1/a/HeLa_EU_siCABIN1_0h_05_w2TX.TIF]

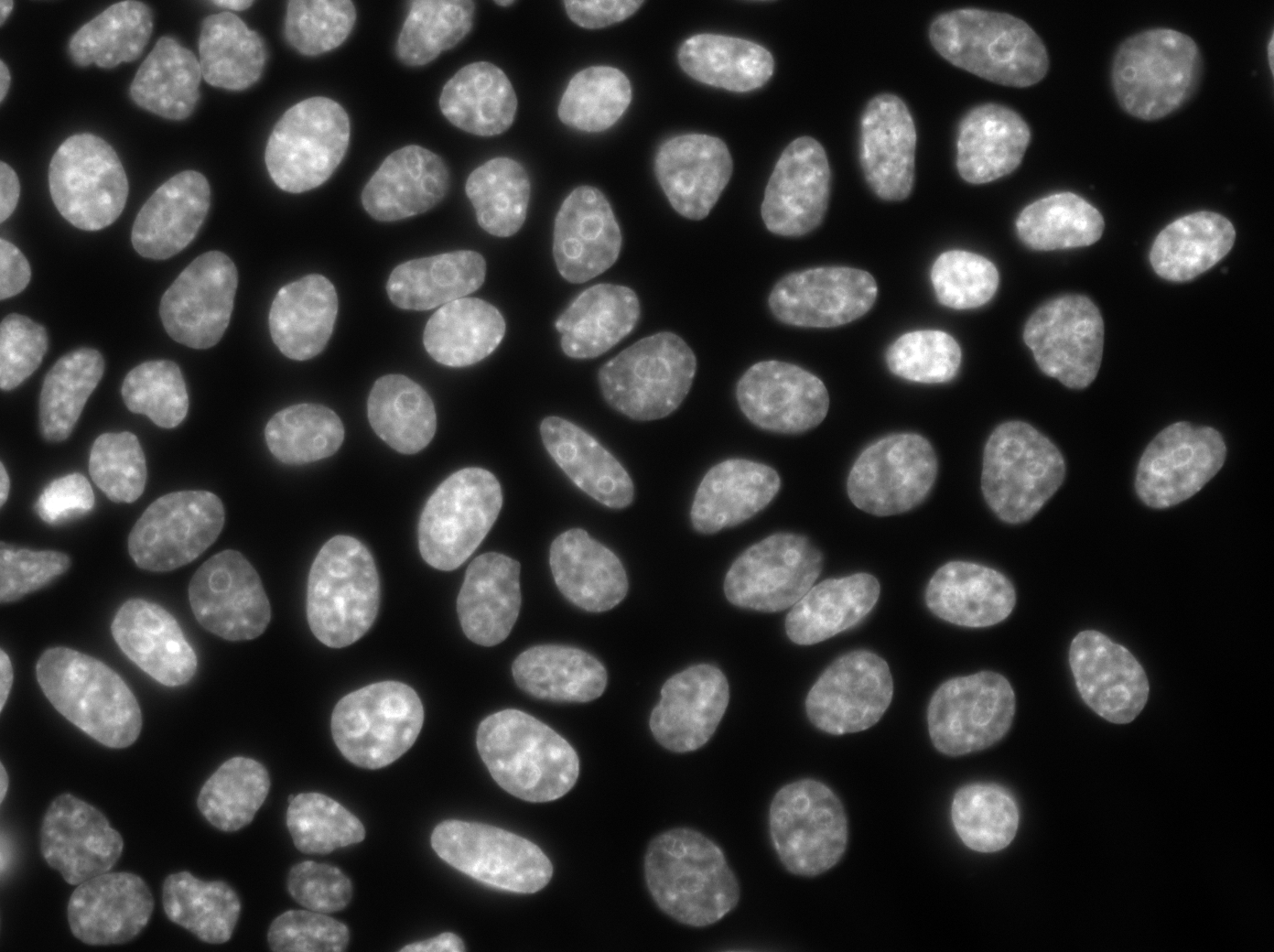

Supplement: Supplementary file 8 — Source Data [file 41467_2021_24153_MOESM8_ESM.zip › RawData/Main Figures/Fig1/a/HeLa_EU_siCABIN1_2h_08_w1DAPI.TIF]

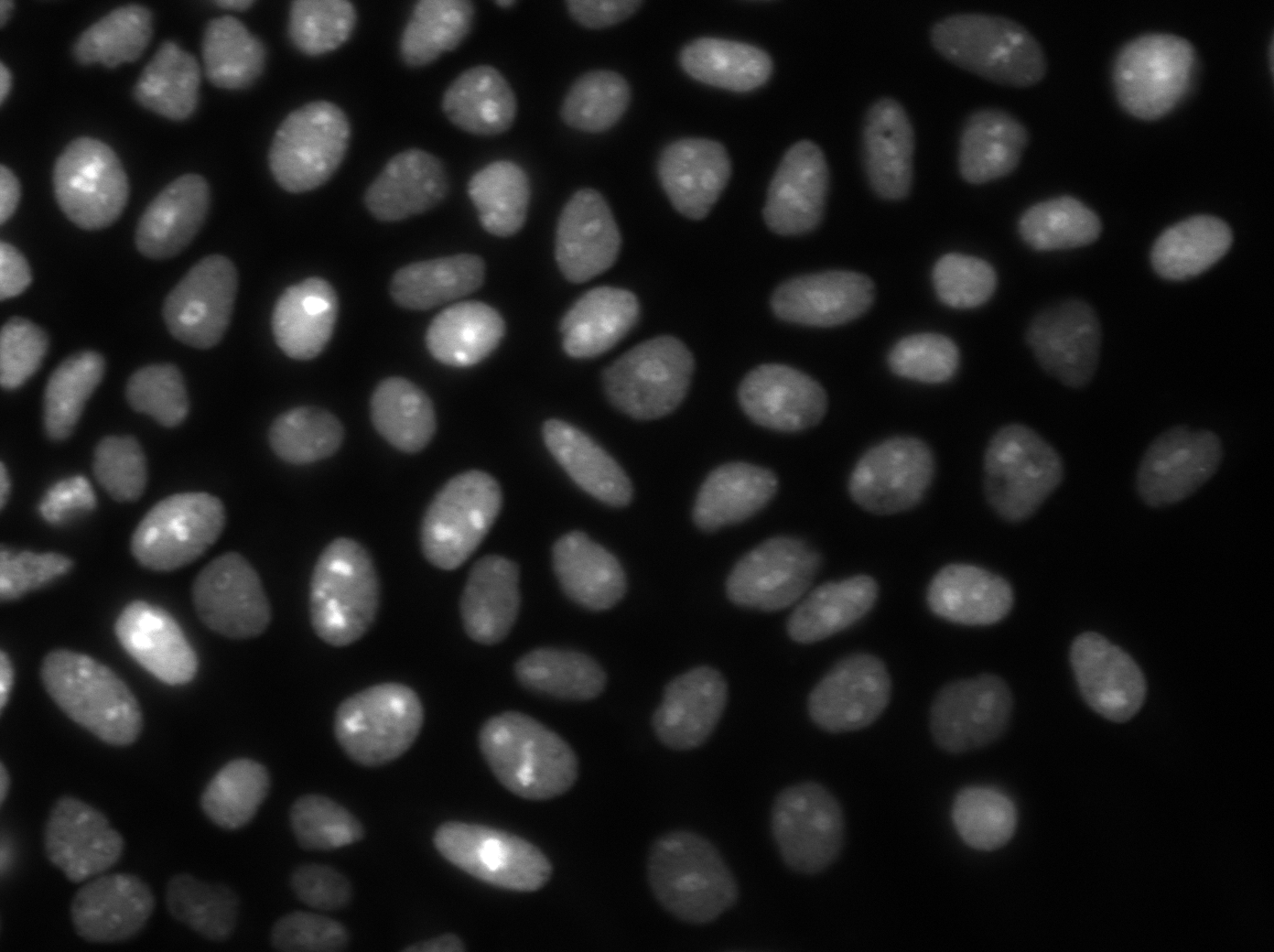

Supplement: Supplementary file 8 — Source Data [file 41467_2021_24153_MOESM8_ESM.zip › RawData/Main Figures/Fig1/a/HeLa_EU_siCABIN1_2h_08_w2TX.TIF]

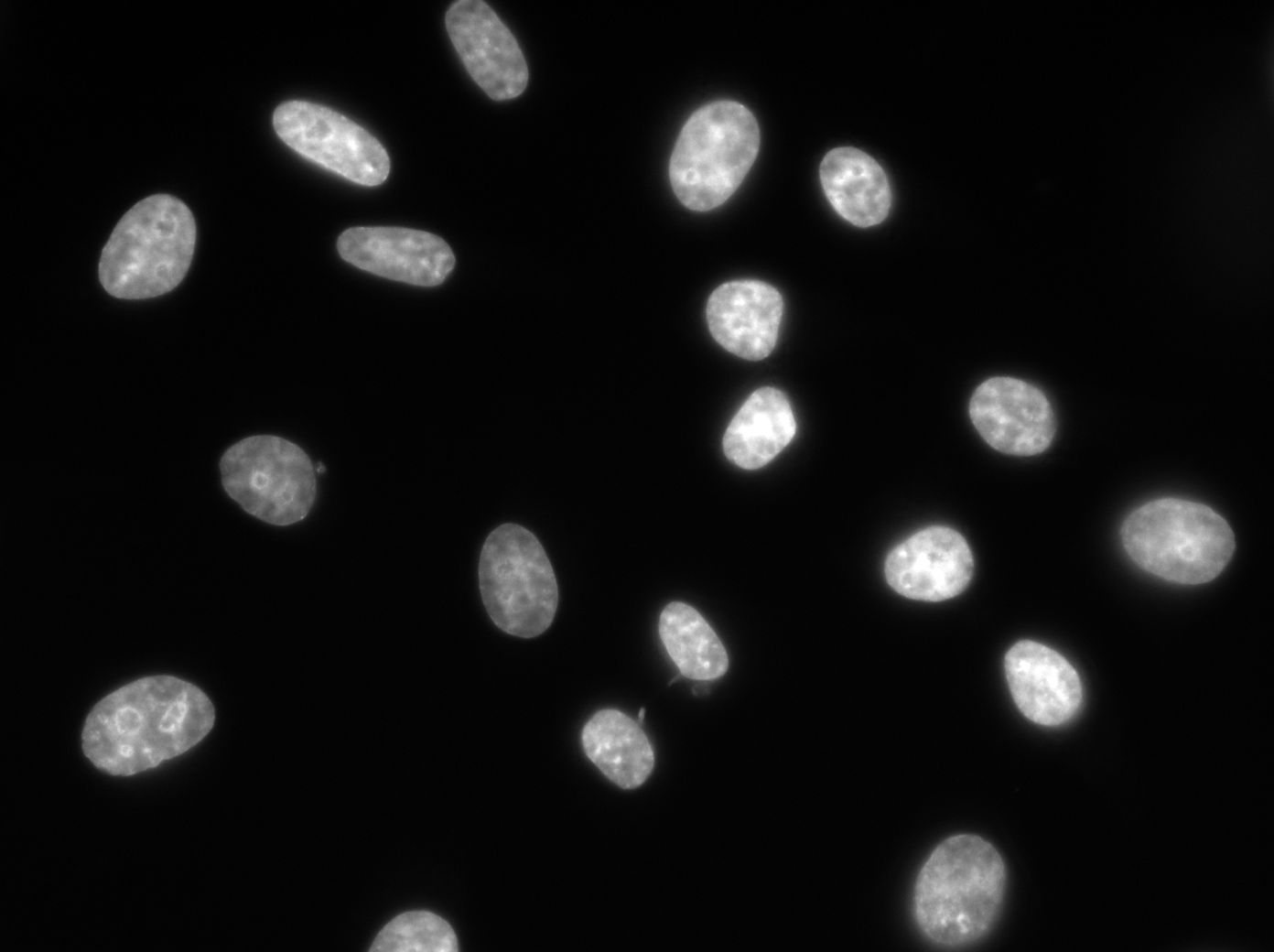

Supplement: Supplementary file 8 — Source Data [file 41467_2021_24153_MOESM8_ESM.zip › RawData/Main Figures/Fig1/a/HeLa_EU_siCABIN_24h_05_w1DAPI.TIF]

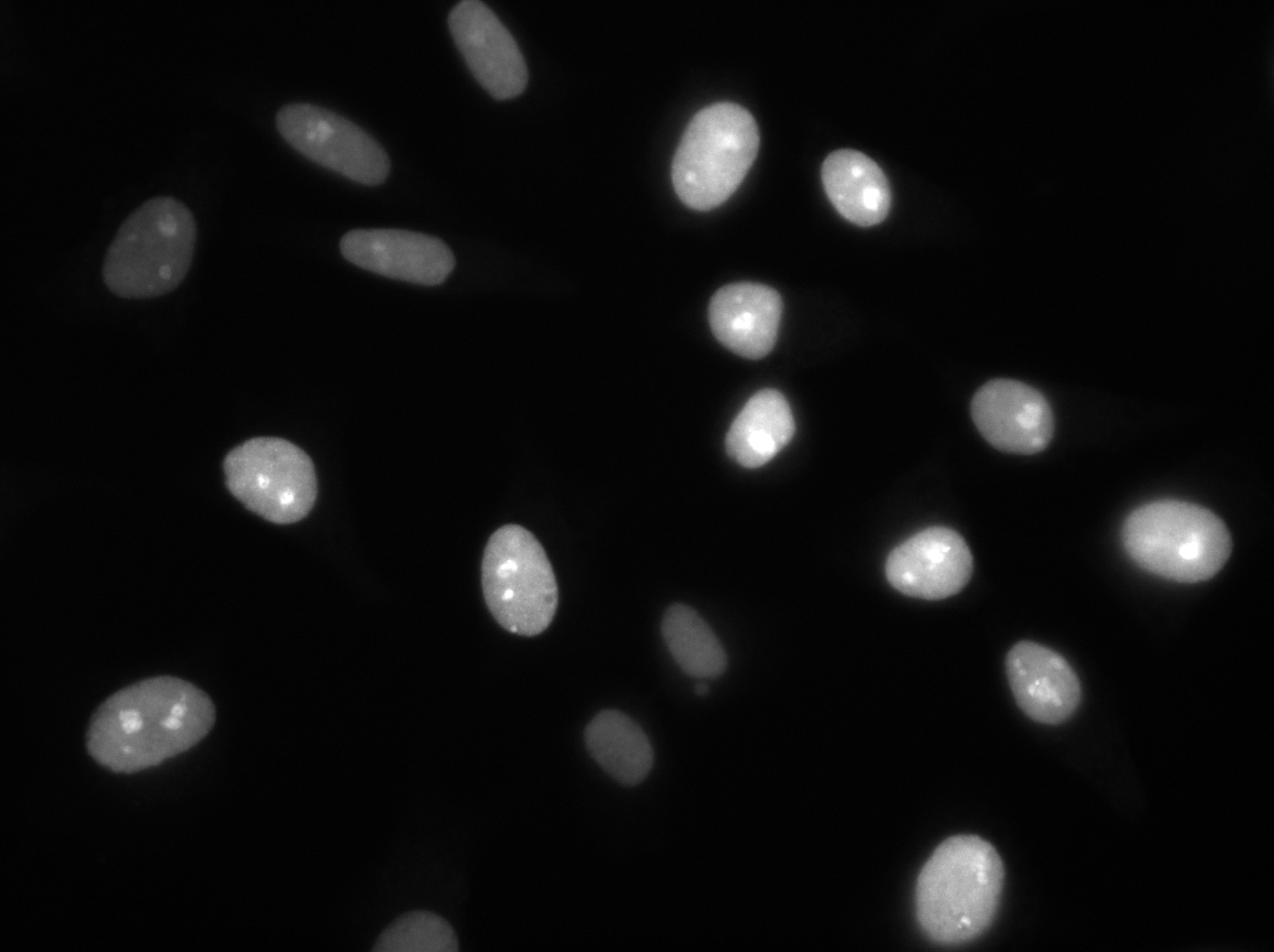

Supplement: Supplementary file 8 — Source Data [file 41467_2021_24153_MOESM8_ESM.zip › RawData/Main Figures/Fig1/a/HeLa_EU_siCABIN_24h_05_w2TX.TIF]

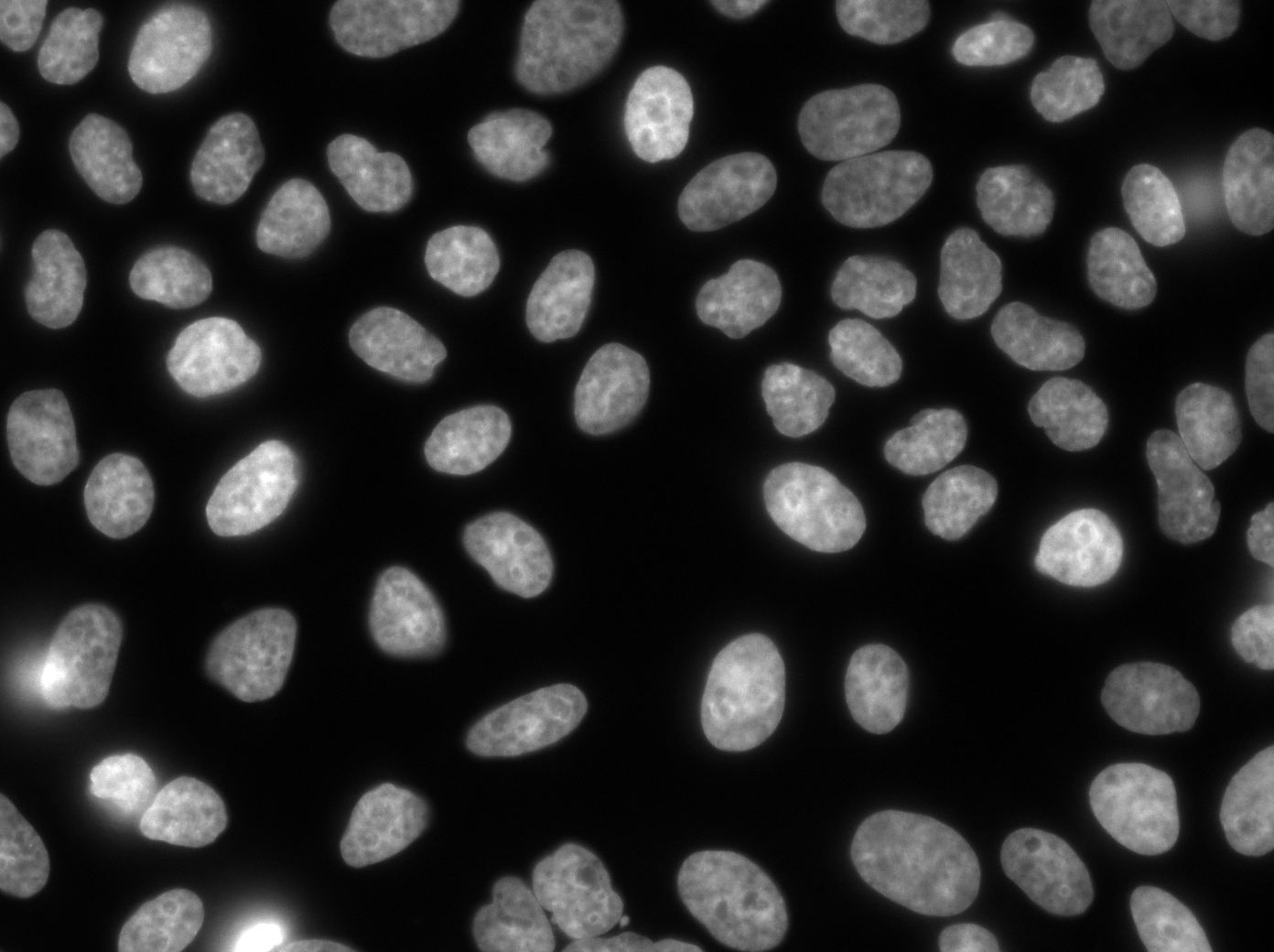

Supplement: Supplementary file 8 — Source Data [file 41467_2021_24153_MOESM8_ESM.zip › RawData/Main Figures/Fig1/a/HeLa_EU_siHIRA_0h_06_w1DAPI.TIF]

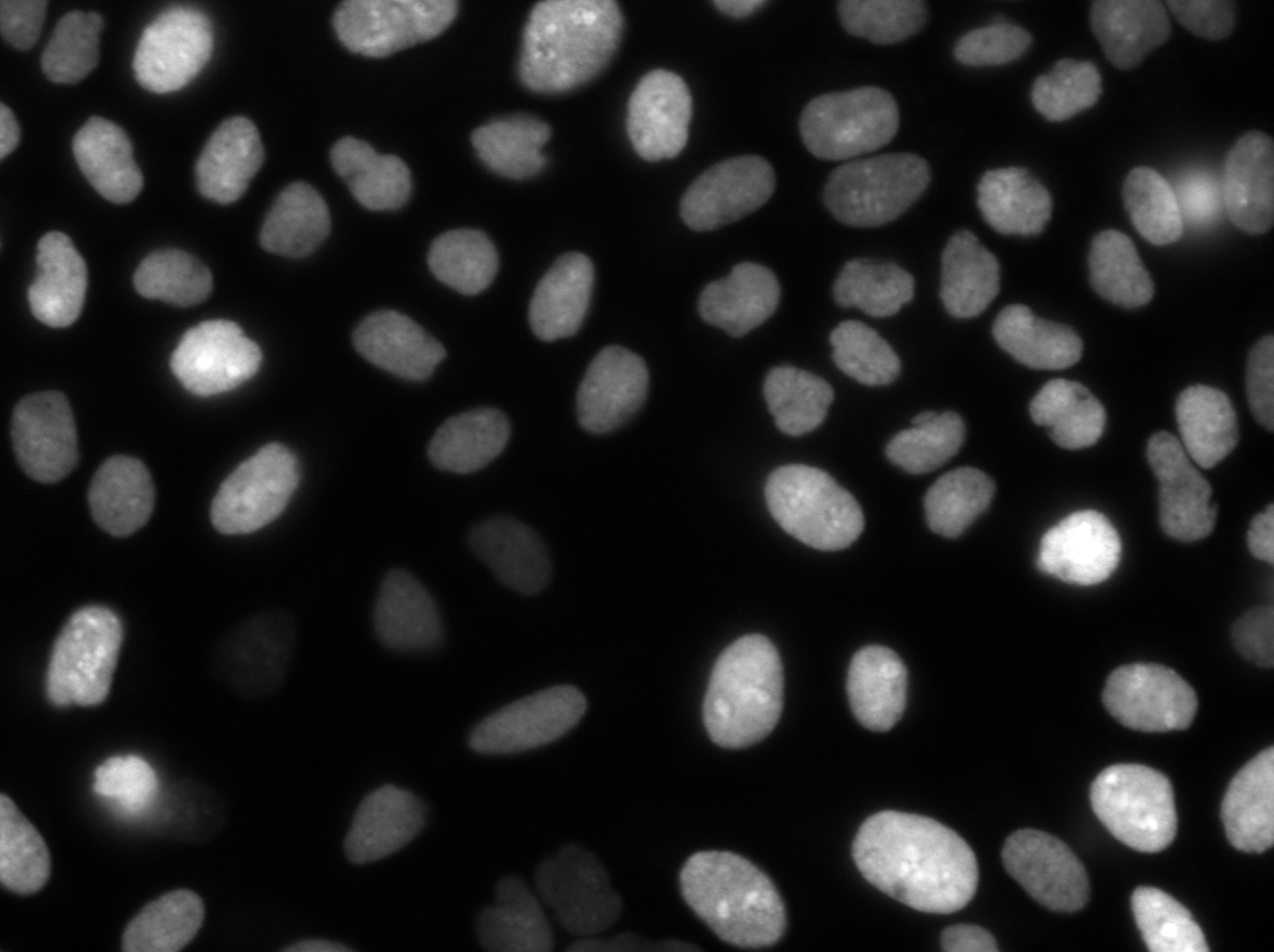

Supplement: Supplementary file 8 — Source Data [file 41467_2021_24153_MOESM8_ESM.zip › RawData/Main Figures/Fig1/a/HeLa_EU_siHIRA_0h_06_w2TX.TIF]

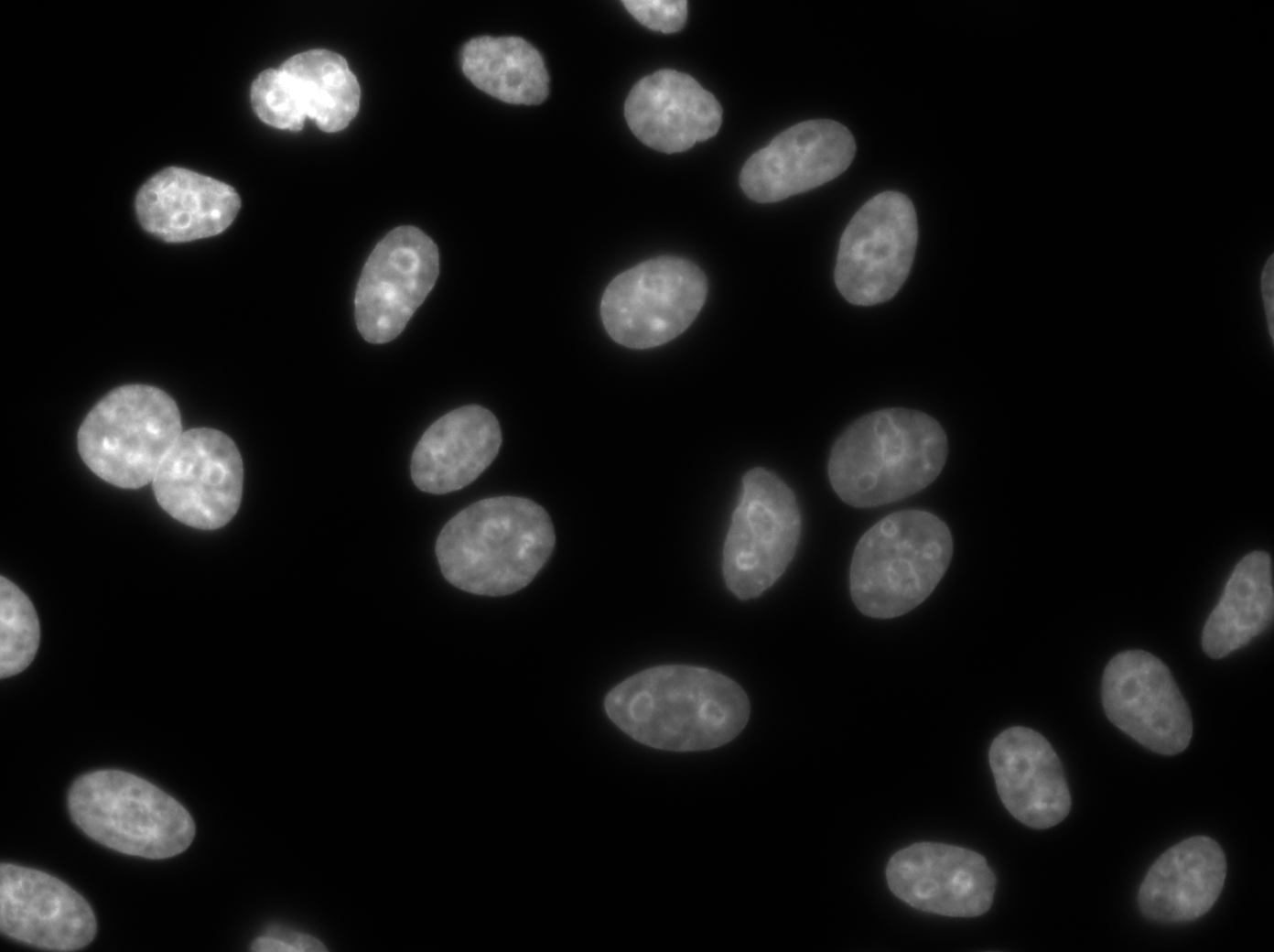

Supplement: Supplementary file 8 — Source Data [file 41467_2021_24153_MOESM8_ESM.zip › RawData/Main Figures/Fig1/a/HeLa_EU_siHIRA_24h_09_w1DAPI.TIF]

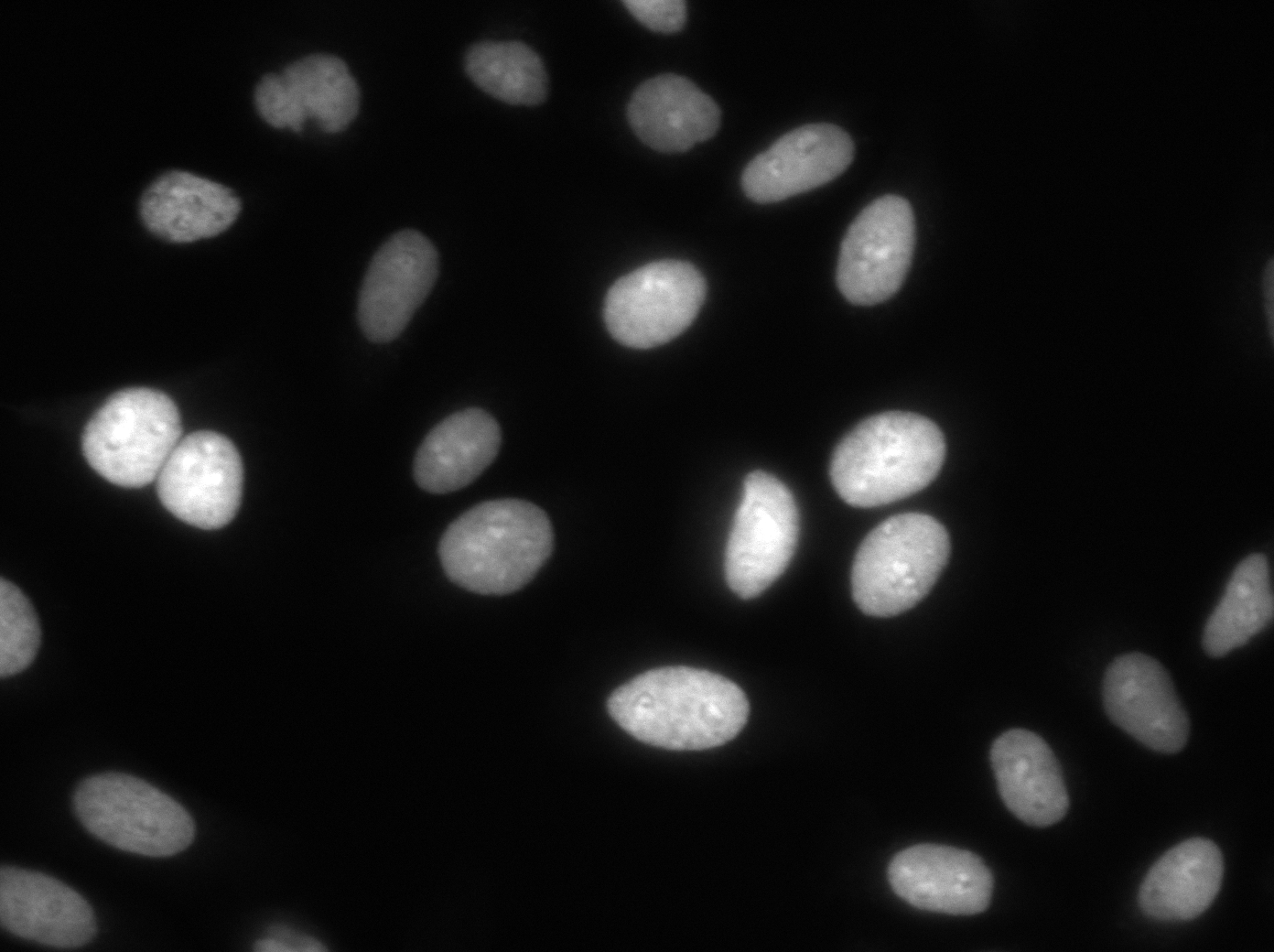

Supplement: Supplementary file 8 — Source Data [file 41467_2021_24153_MOESM8_ESM.zip › RawData/Main Figures/Fig1/a/HeLa_EU_siHIRA_24h_09_w2TX.TIF]

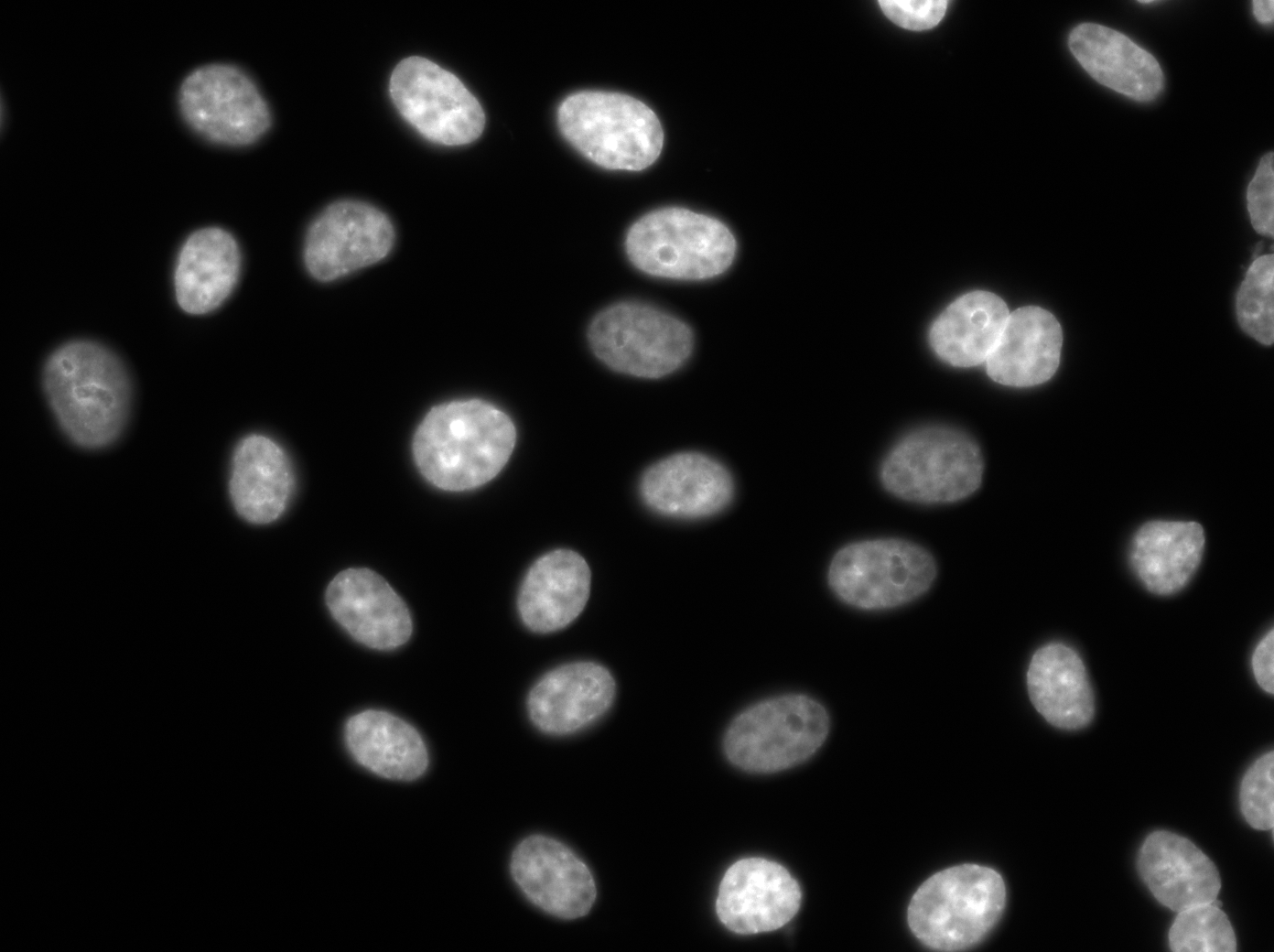

Supplement: Supplementary file 8 — Source Data [file 41467_2021_24153_MOESM8_ESM.zip › RawData/Main Figures/Fig1/a/HeLa_EU_siHIRA_2h_07_w1DAPI.TIF]

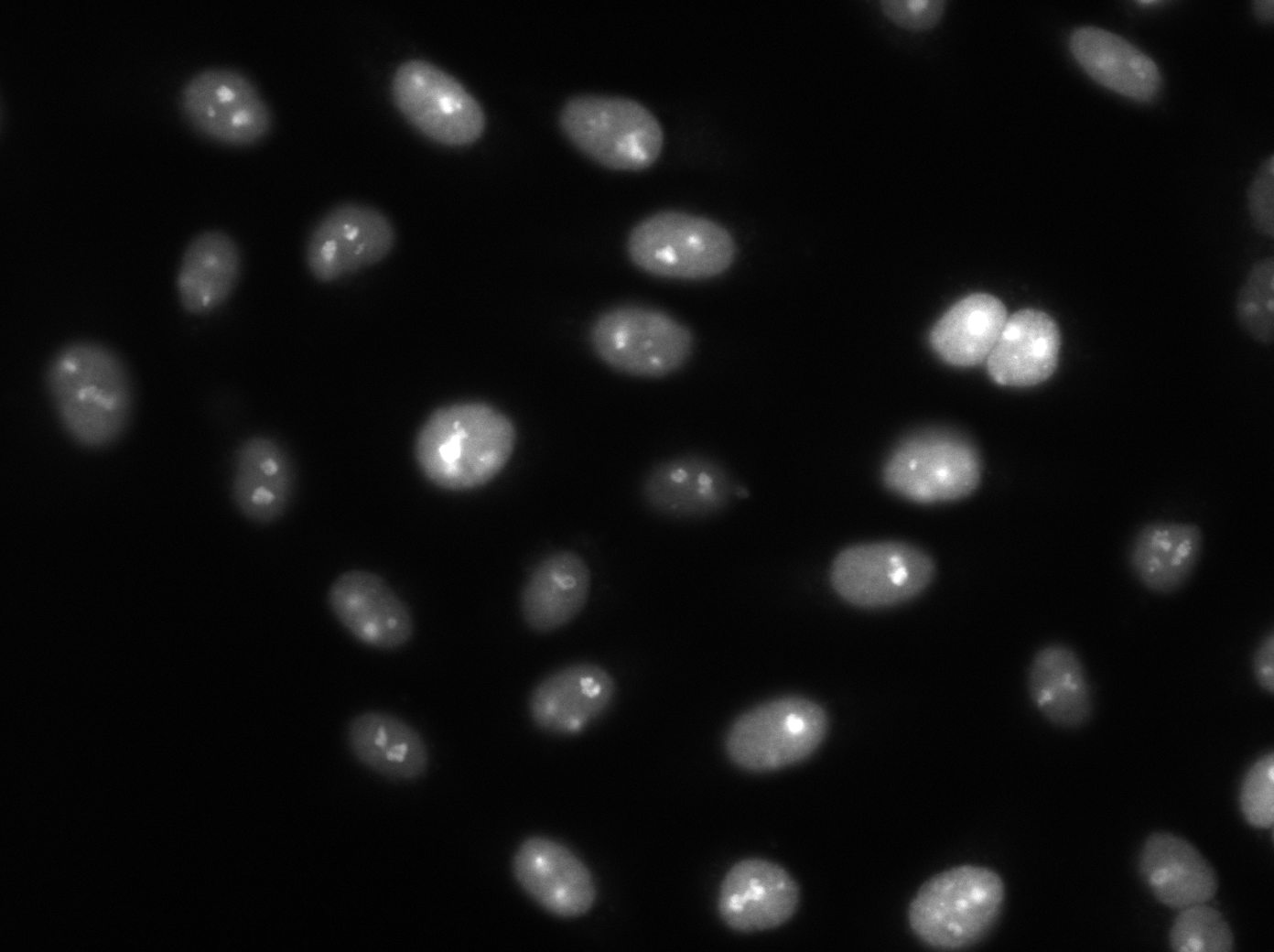

Supplement: Supplementary file 8 — Source Data [file 41467_2021_24153_MOESM8_ESM.zip › RawData/Main Figures/Fig1/a/HeLa_EU_siHIRA_2h_07_w2TX.TIF]

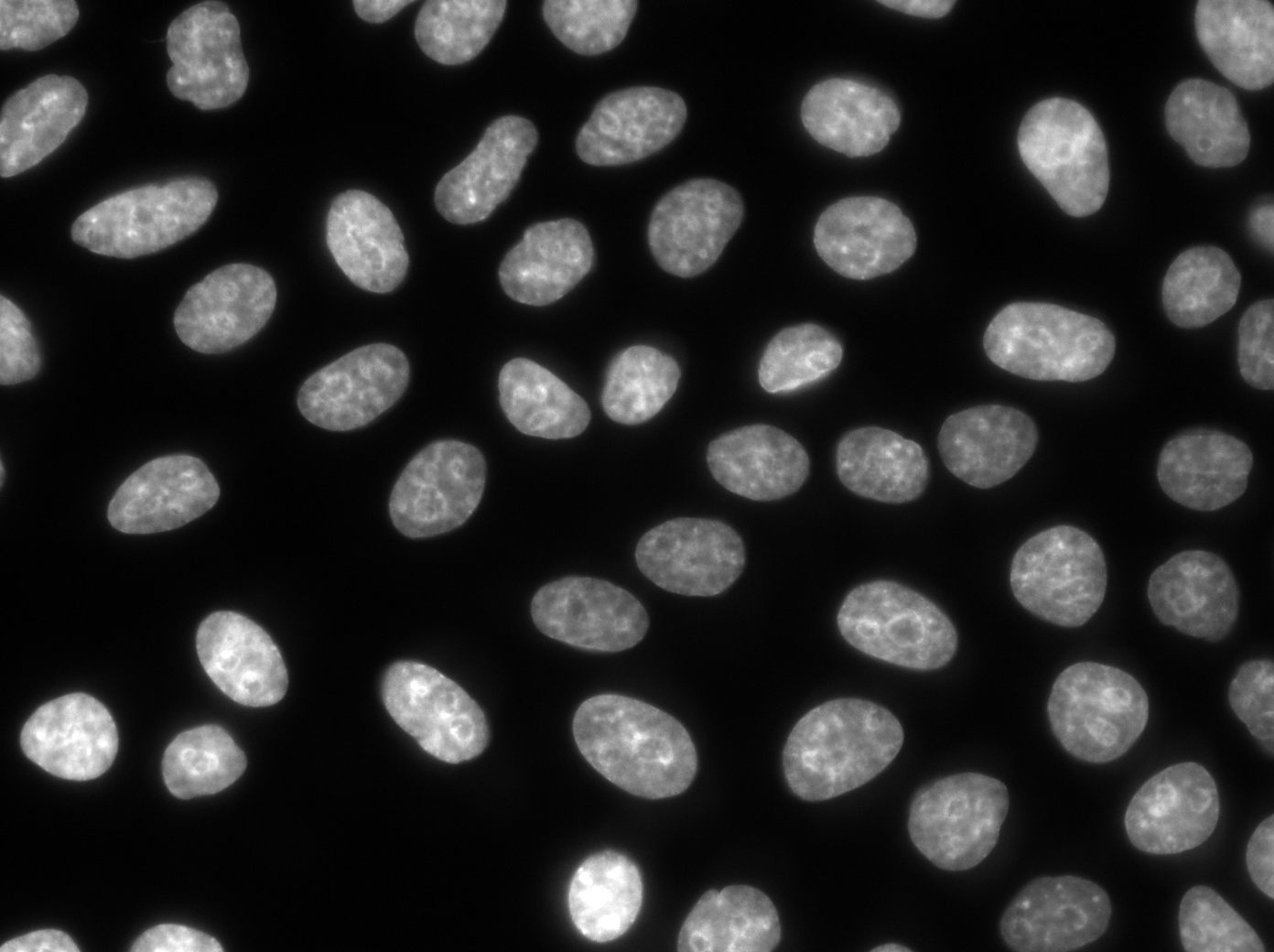

Supplement: Supplementary file 8 — Source Data [file 41467_2021_24153_MOESM8_ESM.zip › RawData/Main Figures/Fig1/a/HeLa_EU_siLUC_0h_06_w1DAPI.TIF]

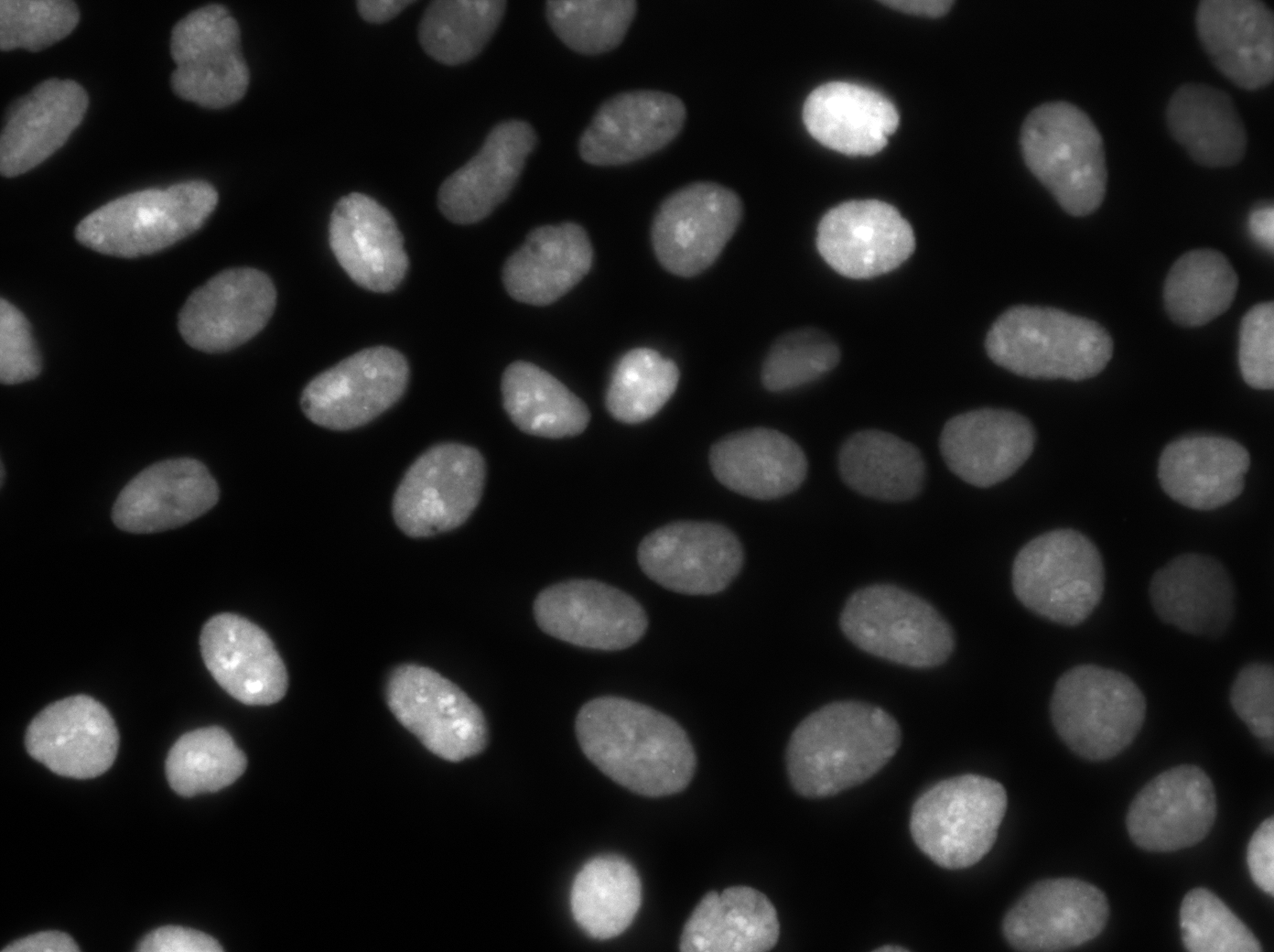

Supplement: Supplementary file 8 — Source Data [file 41467_2021_24153_MOESM8_ESM.zip › RawData/Main Figures/Fig1/a/HeLa_EU_siLUC_0h_06_w2TX.TIF]

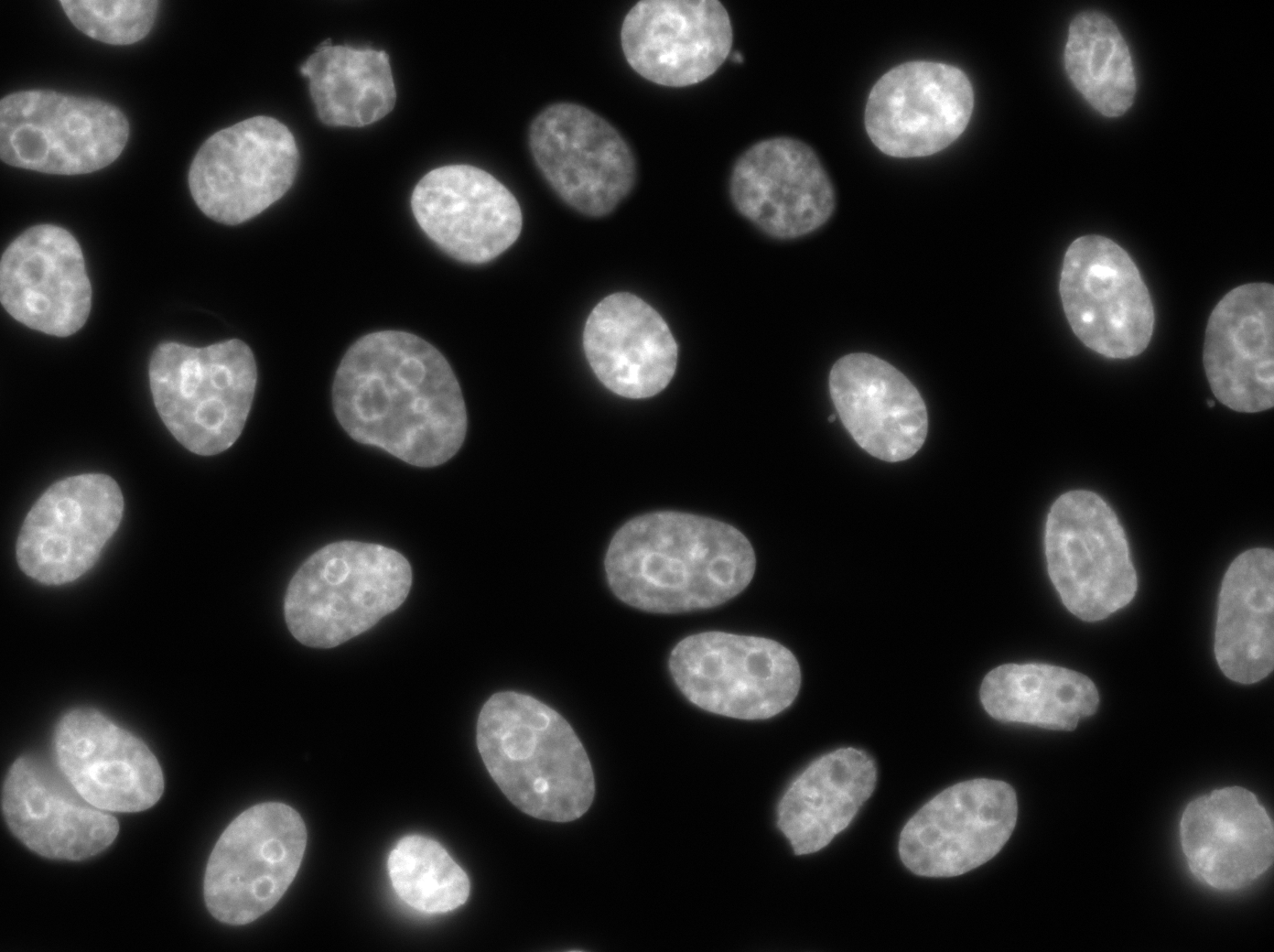

Supplement: Supplementary file 8 — Source Data [file 41467_2021_24153_MOESM8_ESM.zip › RawData/Main Figures/Fig1/a/HeLa_EU_siLUC_24h_02_w1DAPI.TIF]

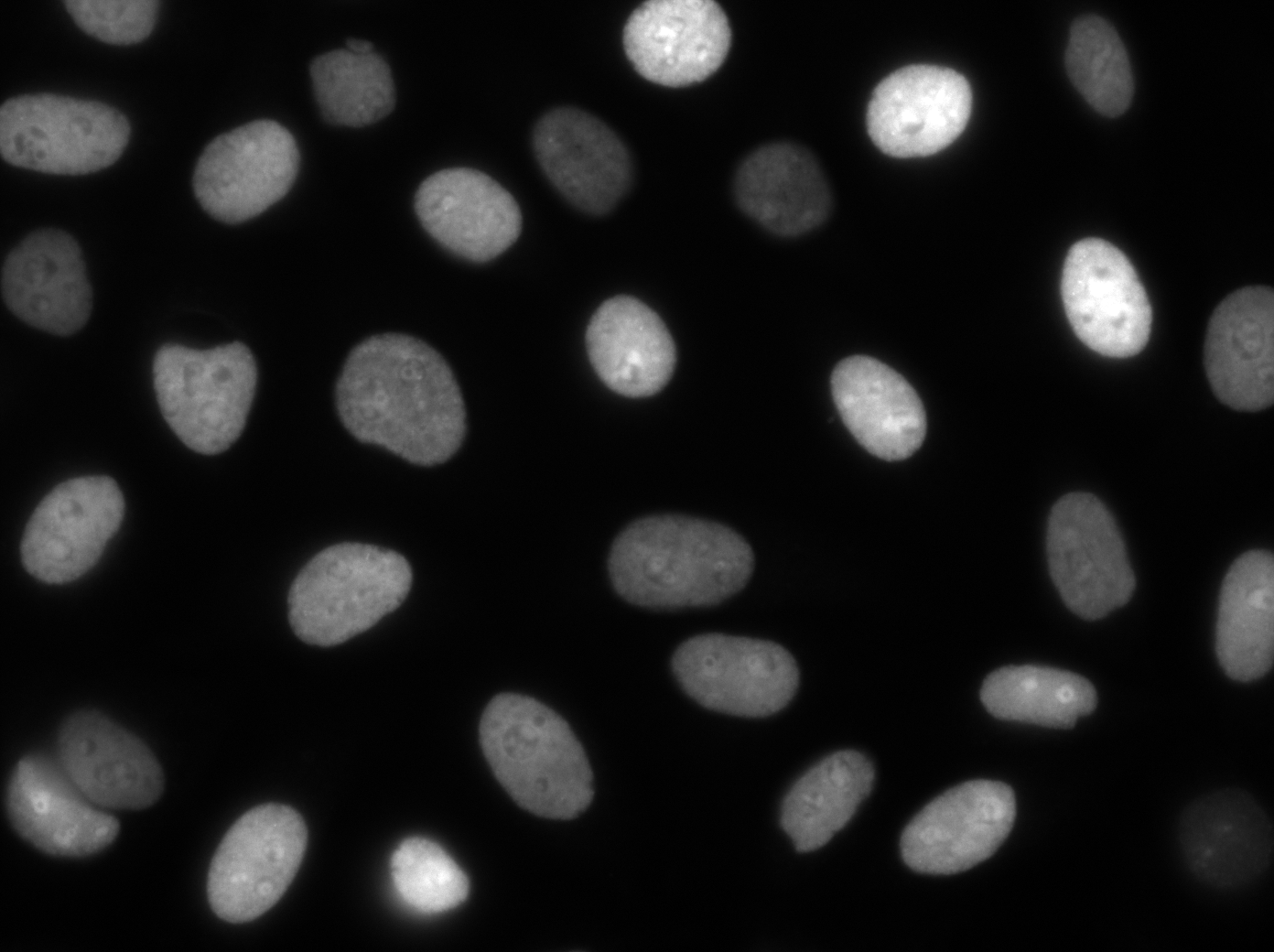

Supplement: Supplementary file 8 — Source Data [file 41467_2021_24153_MOESM8_ESM.zip › RawData/Main Figures/Fig1/a/HeLa_EU_siLUC_24h_02_w2TX.TIF]

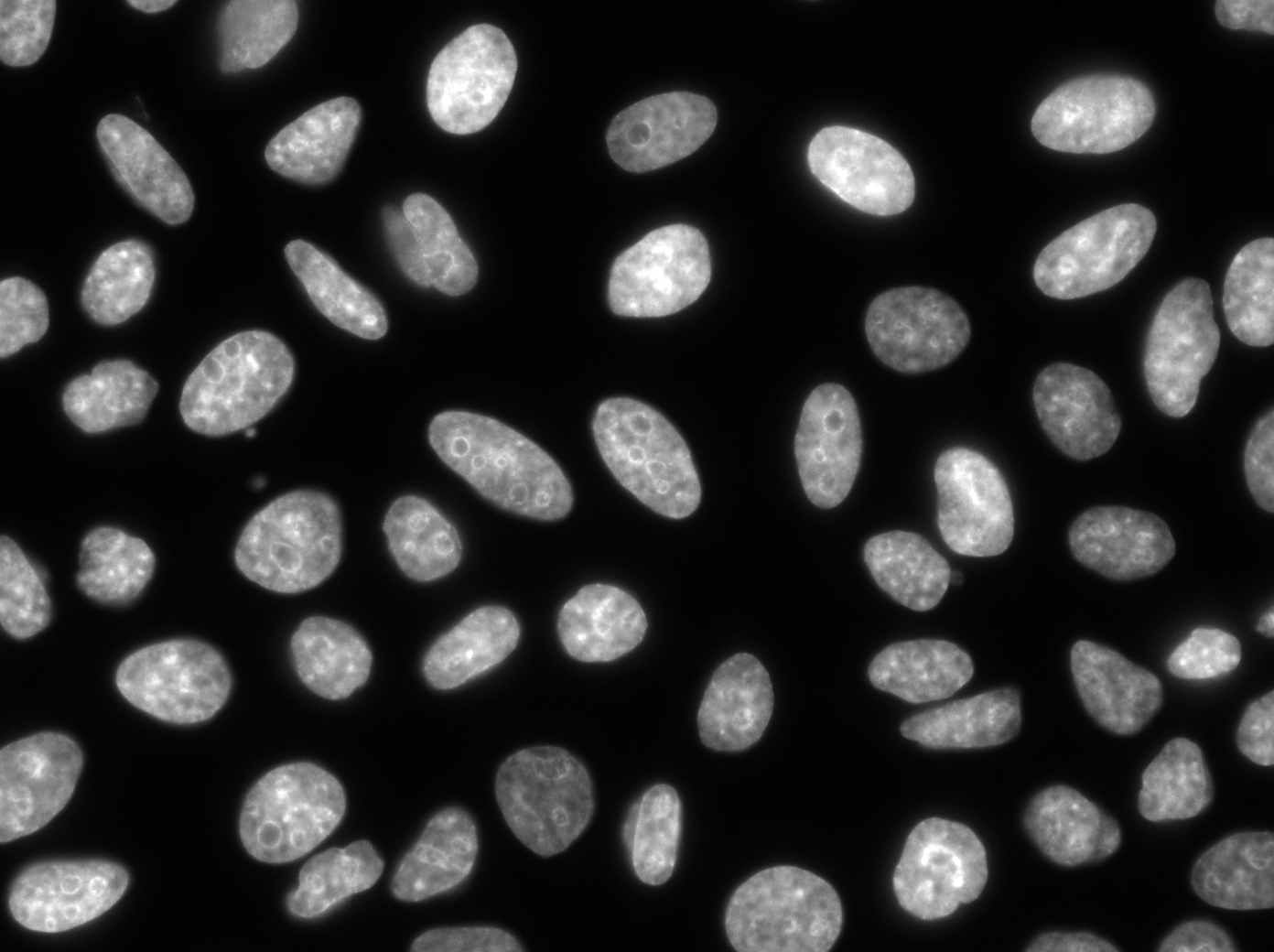

Supplement: Supplementary file 8 — Source Data [file 41467_2021_24153_MOESM8_ESM.zip › RawData/Main Figures/Fig1/a/HeLa_EU_siLUC_2h_05_w1DAPI.TIF]

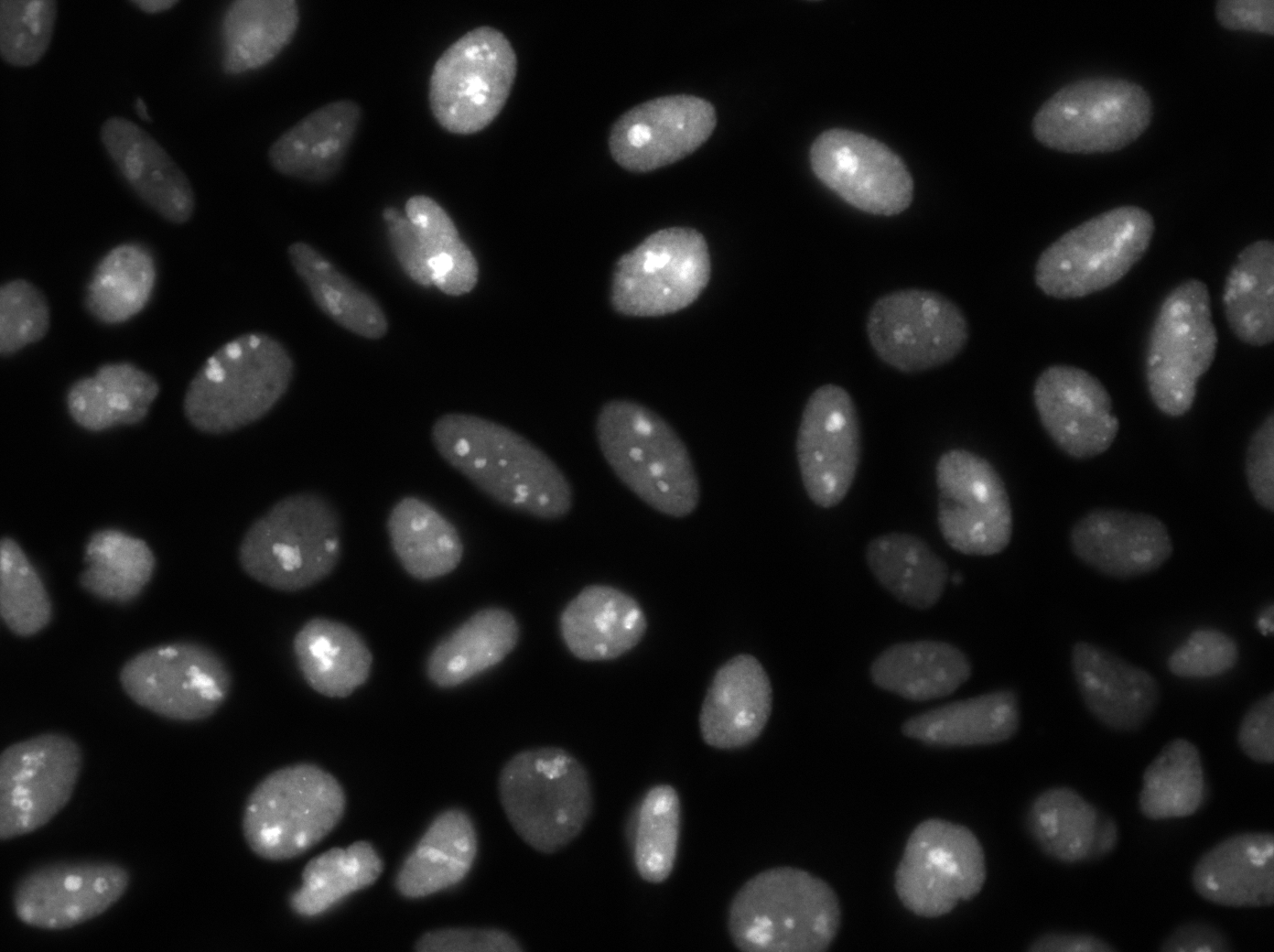

Supplement: Supplementary file 8 — Source Data [file 41467_2021_24153_MOESM8_ESM.zip › RawData/Main Figures/Fig1/a/HeLa_EU_siLUC_2h_05_w2TX.TIF]

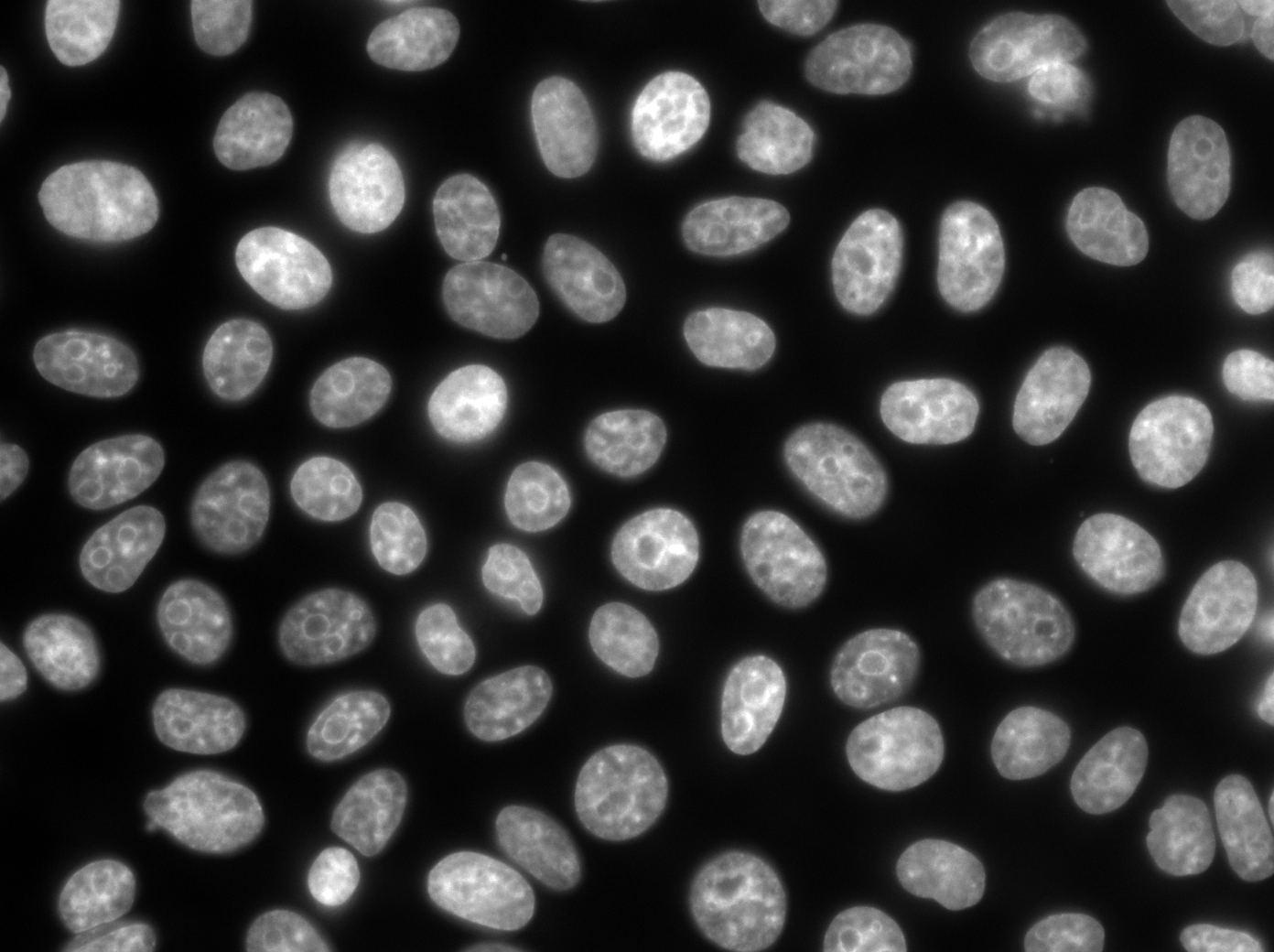

Supplement: Supplementary file 8 — Source Data [file 41467_2021_24153_MOESM8_ESM.zip › RawData/Main Figures/Fig1/a/HeLa_EU_siUBN1_0h_05_w1DAPI.TIF]

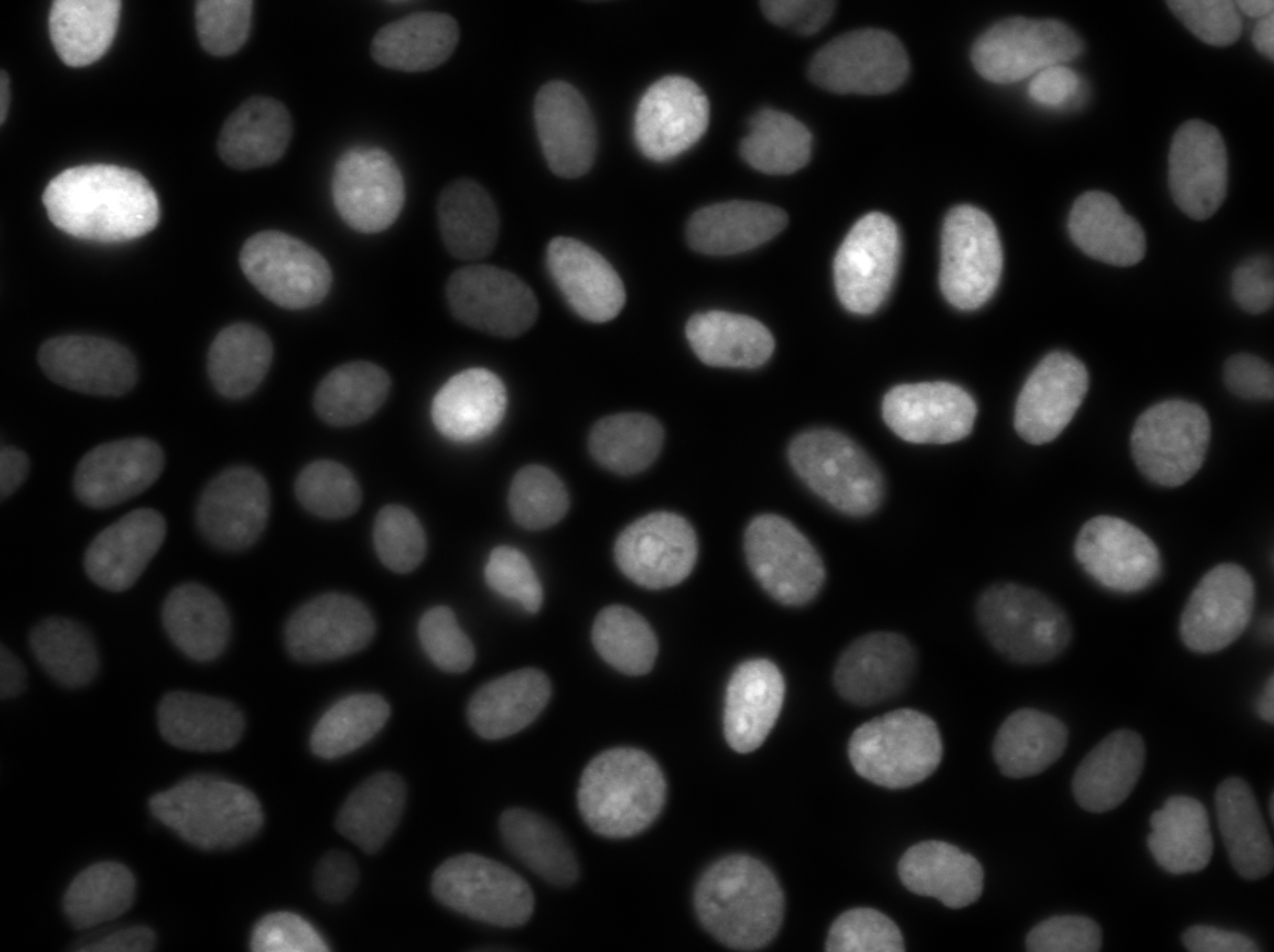

Supplement: Supplementary file 8 — Source Data [file 41467_2021_24153_MOESM8_ESM.zip › RawData/Main Figures/Fig1/a/HeLa_EU_siUBN1_0h_05_w2TX.TIF]

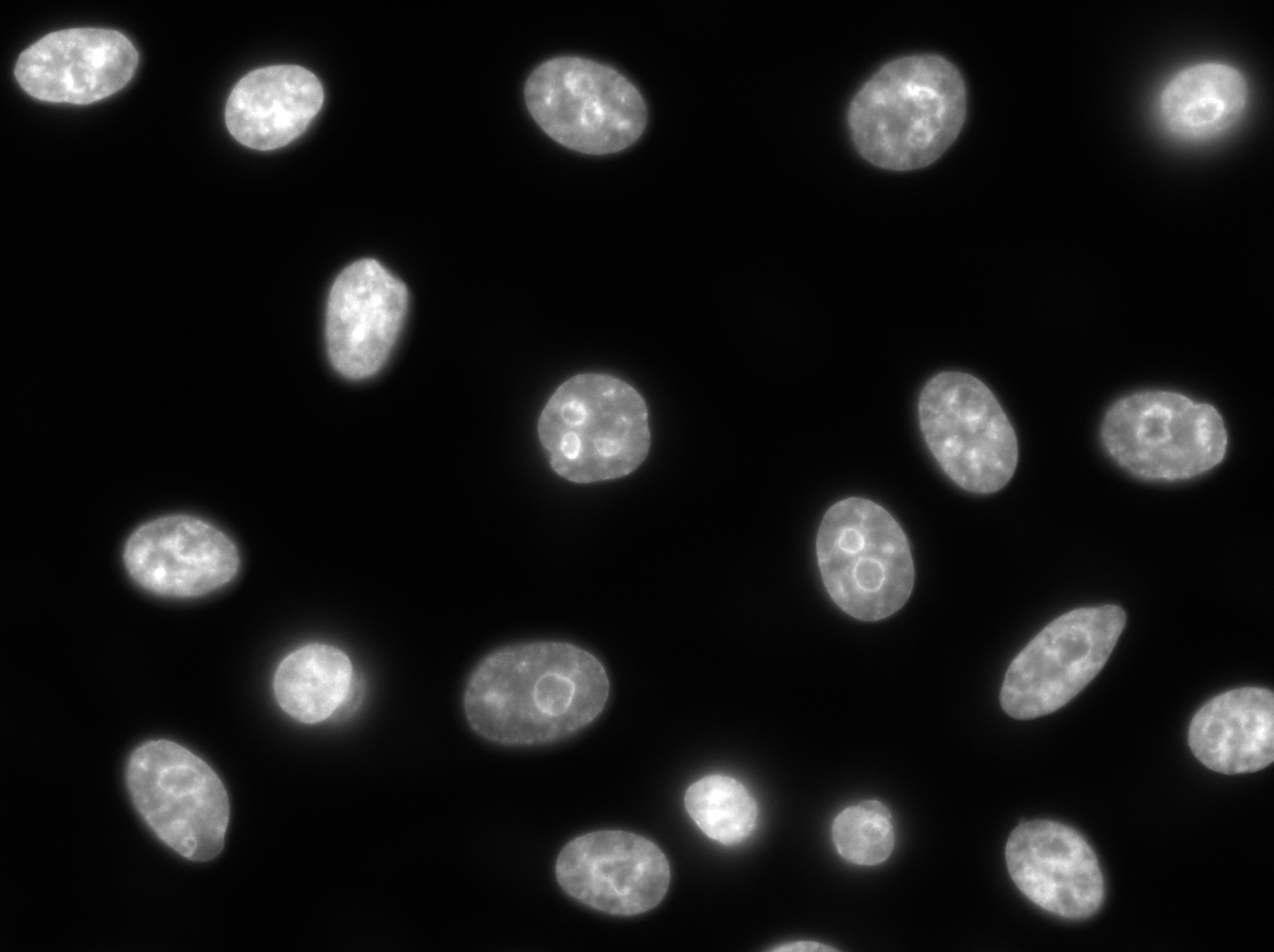

Supplement: Supplementary file 8 — Source Data [file 41467_2021_24153_MOESM8_ESM.zip › RawData/Main Figures/Fig1/a/HeLa_EU_siUBN1_24h_01_w1DAPI.TIF]

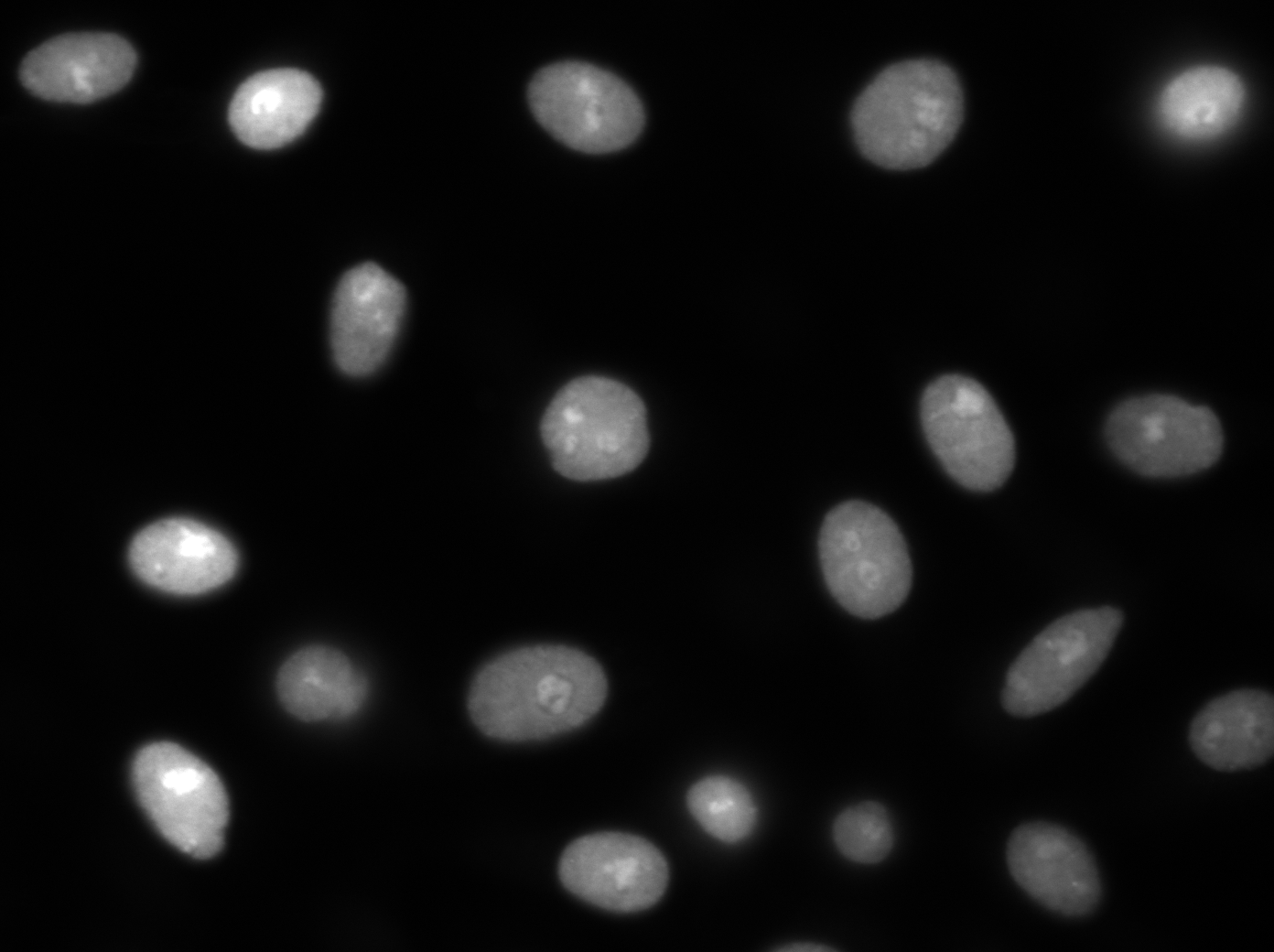

Supplement: Supplementary file 8 — Source Data [file 41467_2021_24153_MOESM8_ESM.zip › RawData/Main Figures/Fig1/a/HeLa_EU_siUBN1_24h_01_w2TX.TIF]

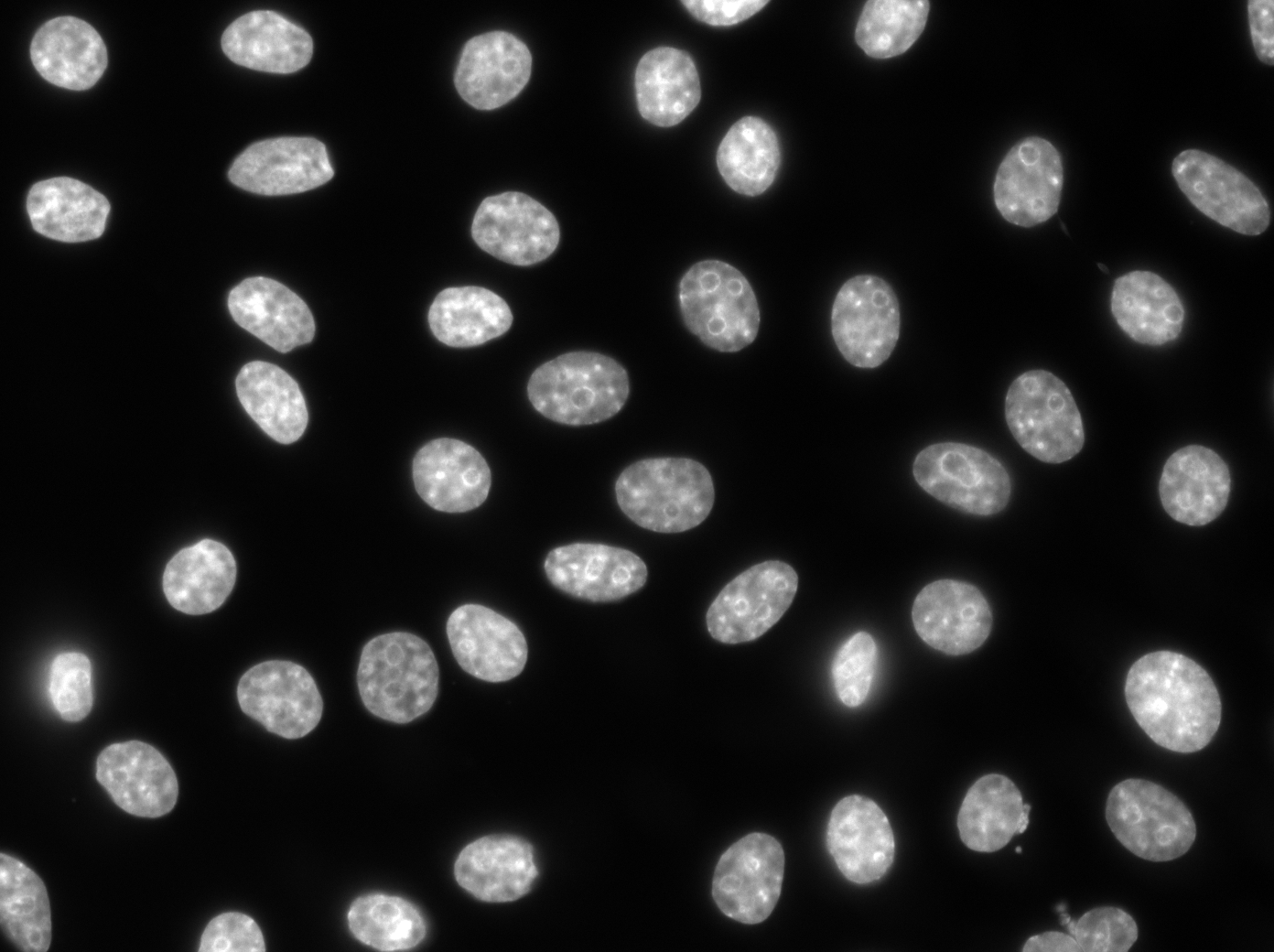

Supplement: Supplementary file 8 — Source Data [file 41467_2021_24153_MOESM8_ESM.zip › RawData/Main Figures/Fig1/a/HeLa_EU_siUBN1_2h_08_w1DAPI.TIF]

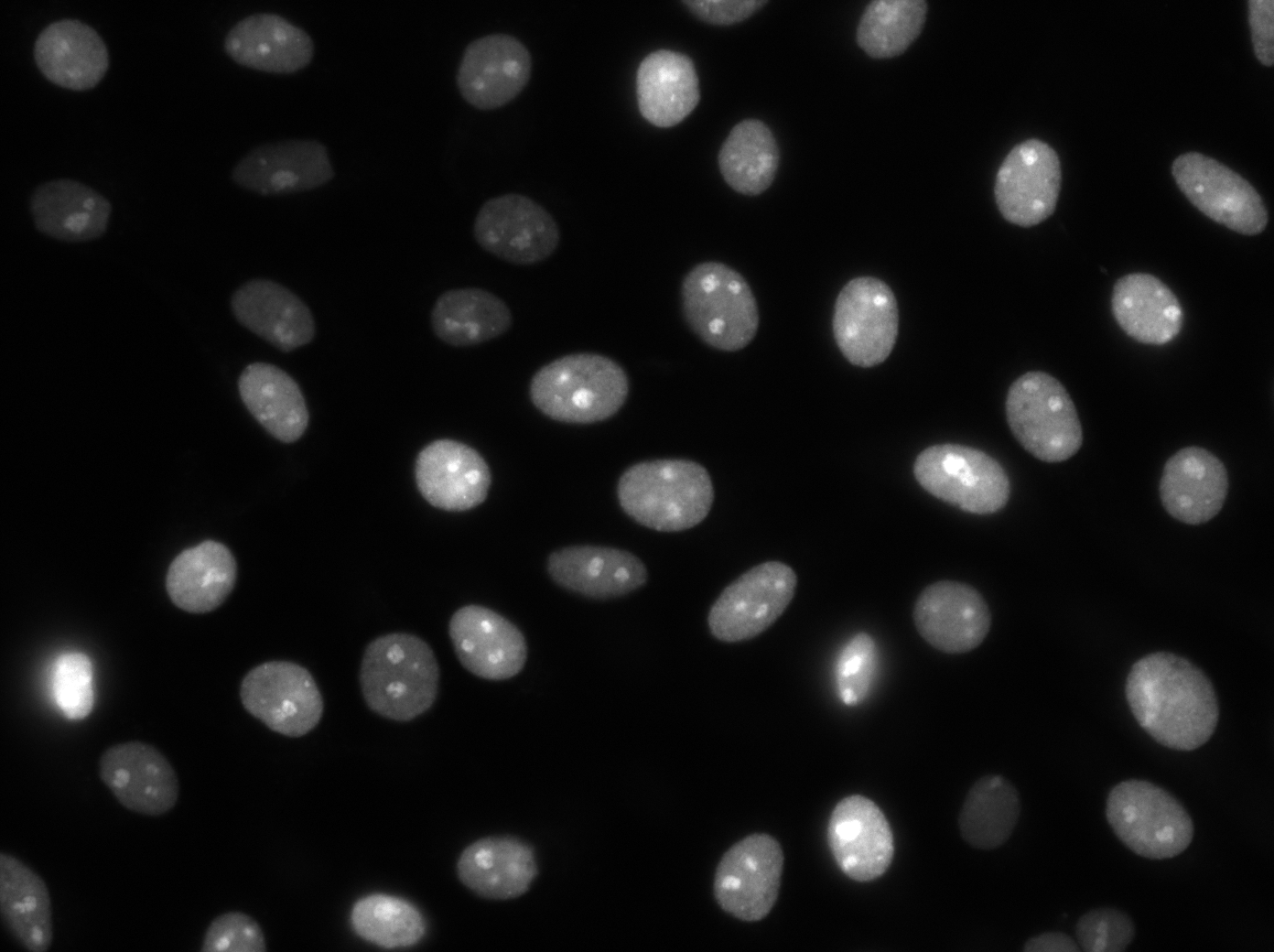

Supplement: Supplementary file 8 — Source Data [file 41467_2021_24153_MOESM8_ESM.zip › RawData/Main Figures/Fig1/a/HeLa_EU_siUBN1_2h_08_w2TX.TIF]

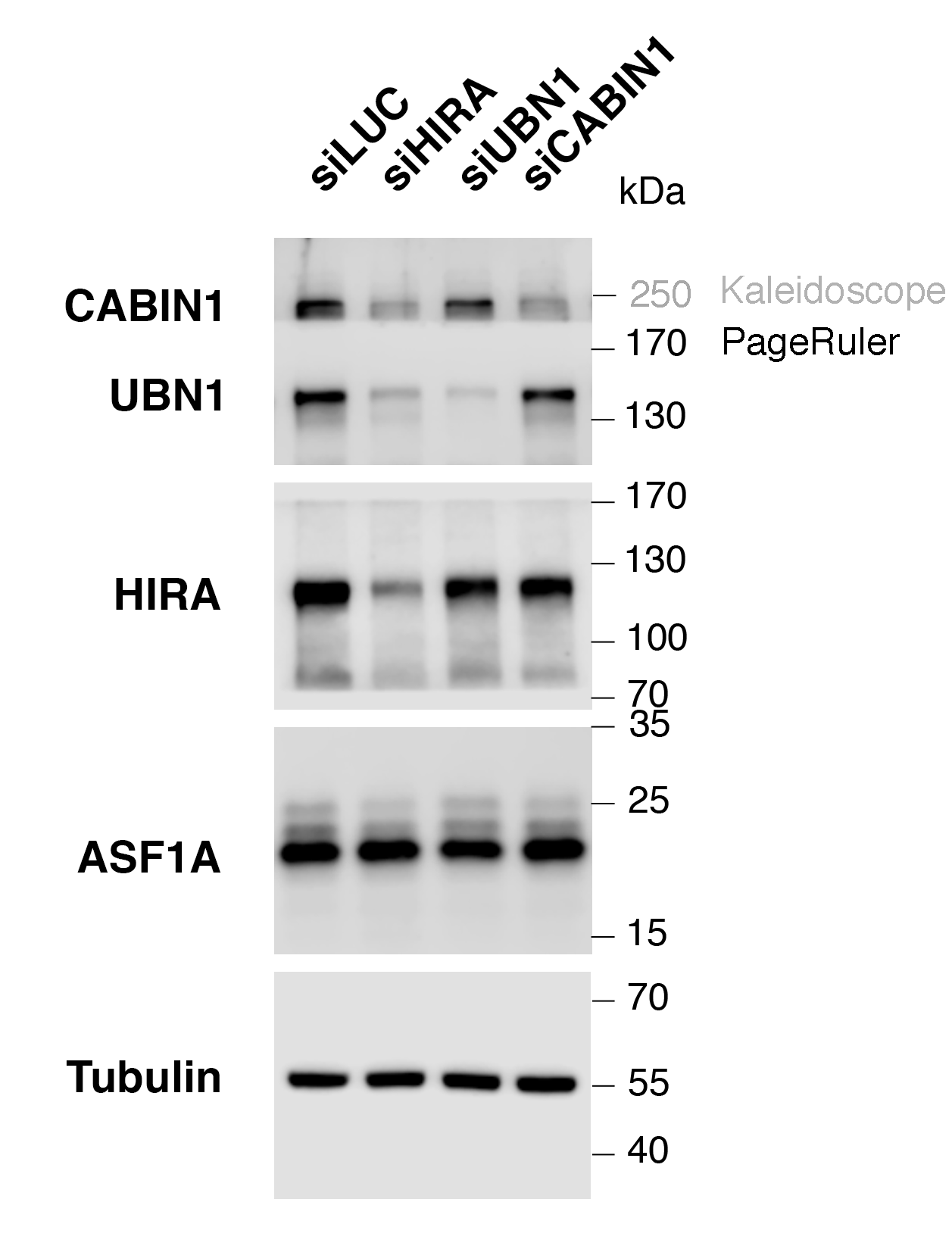

Supplement: Supplementary file 8 — Source Data [file 41467_2021_24153_MOESM8_ESM.zip › RawData/Main Figures/Fig1/a/WB.tif]

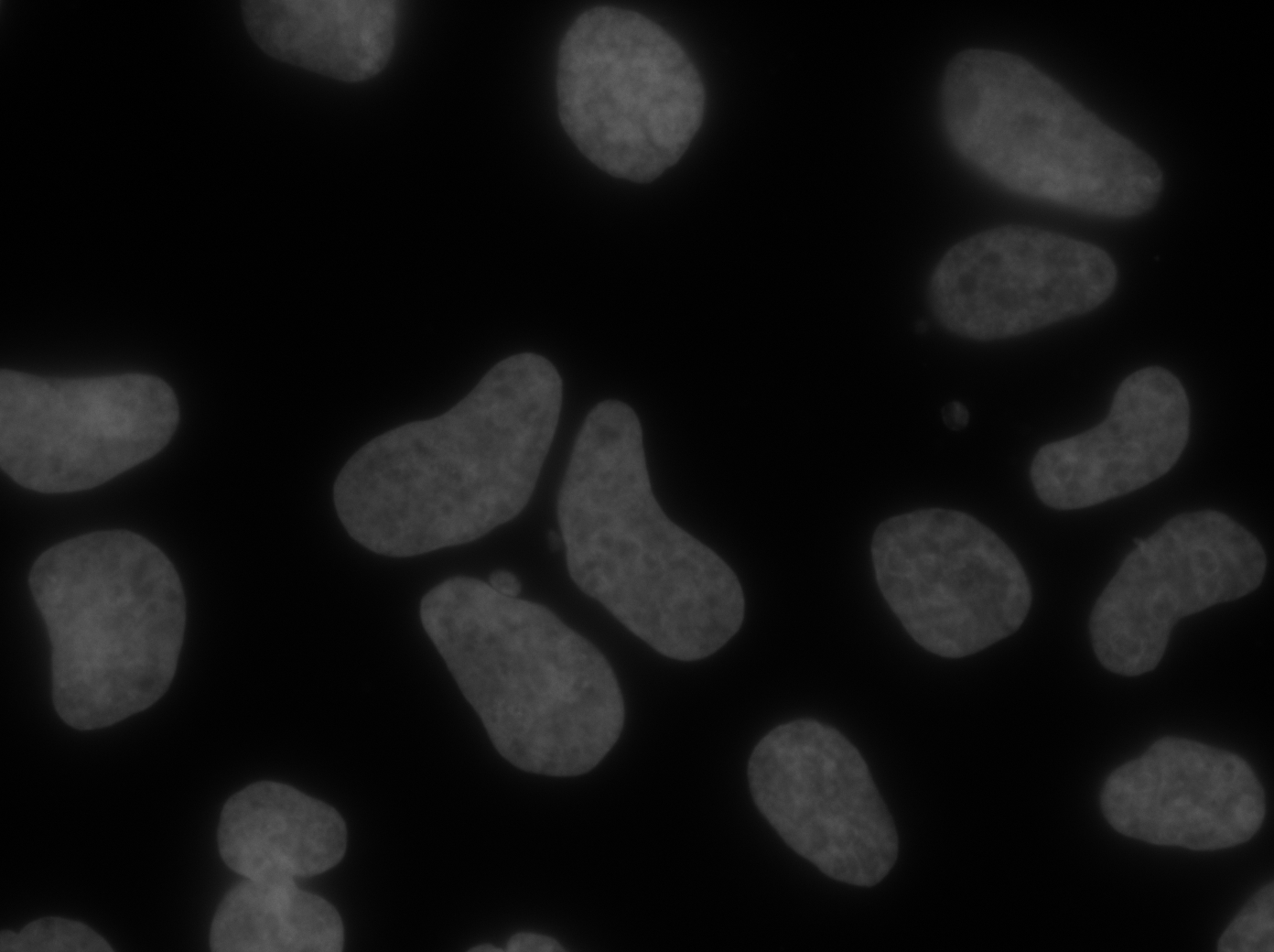

Supplement: Supplementary file 8 — Source Data [file 41467_2021_24153_MOESM8_ESM.zip › RawData/Main Figures/Fig1/b/U2OSH33SNAP_150J_siCABIN1_03_w1DAPI.TIF]

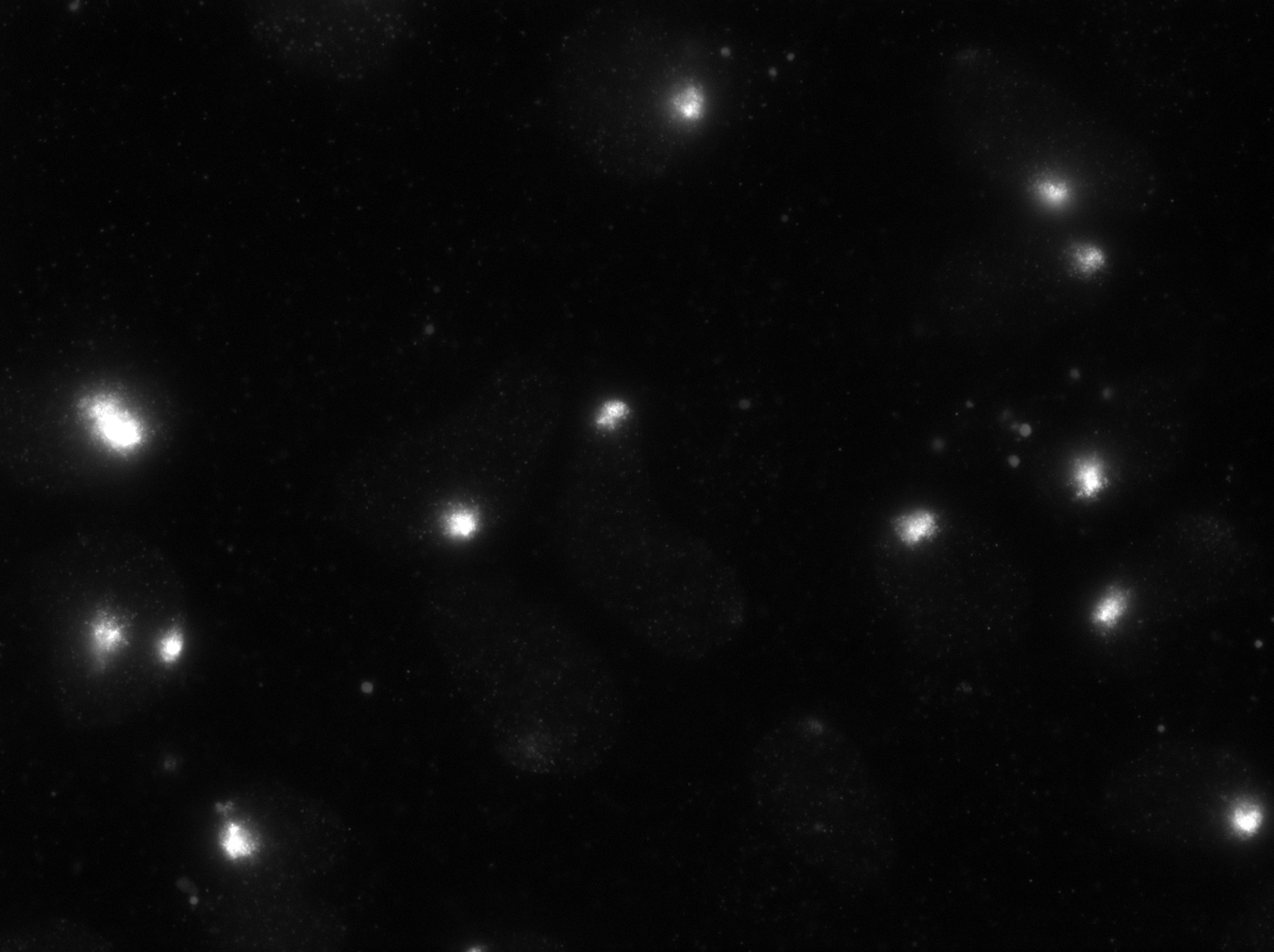

Supplement: Supplementary file 8 — Source Data [file 41467_2021_24153_MOESM8_ESM.zip › RawData/Main Figures/Fig1/b/U2OSH33SNAP_150J_siCABIN1_03_w2GFP.TIF]

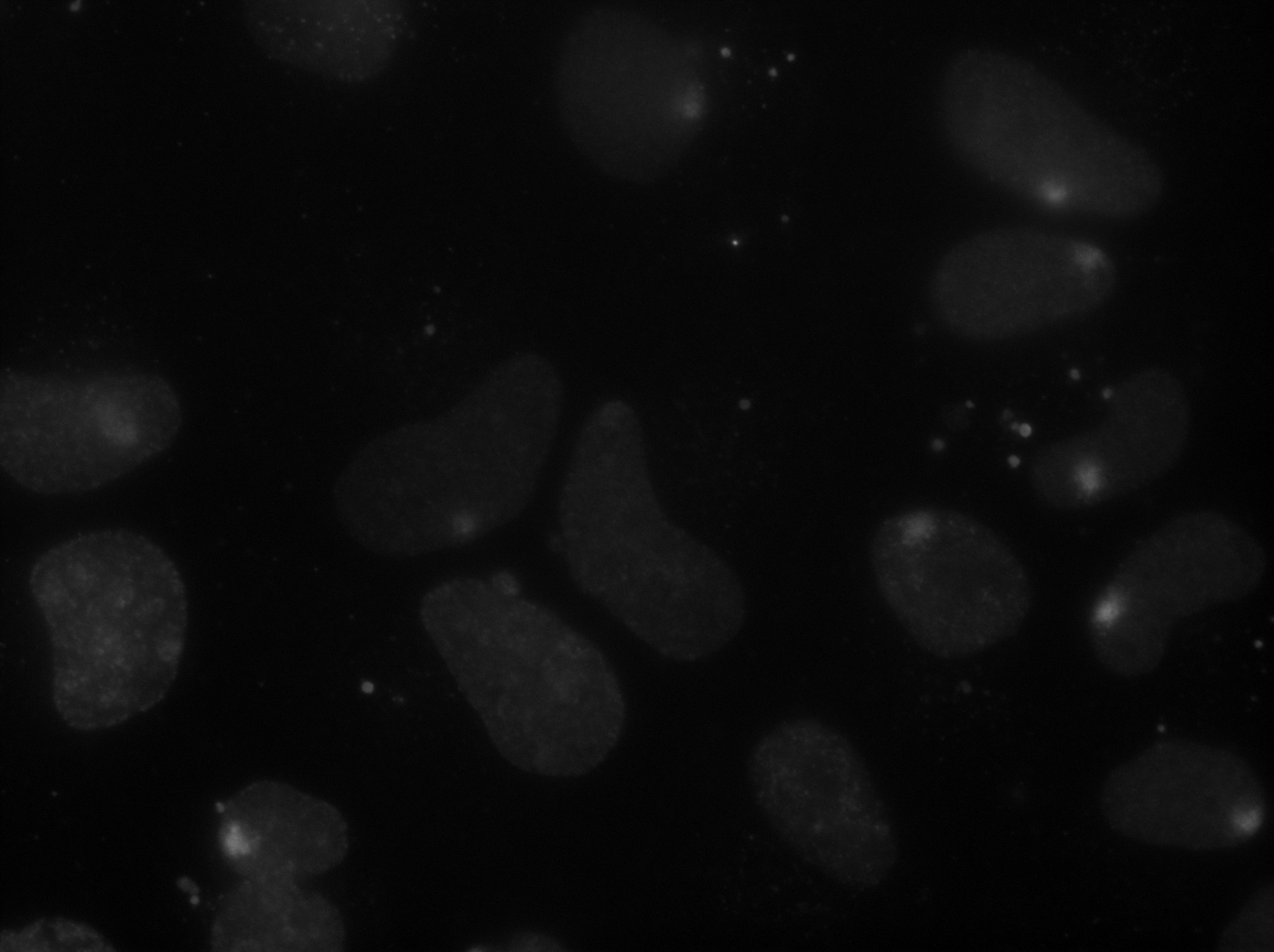

Supplement: Supplementary file 8 — Source Data [file 41467_2021_24153_MOESM8_ESM.zip › RawData/Main Figures/Fig1/b/U2OSH33SNAP_150J_siCABIN1_03_w3CY3.TIF]

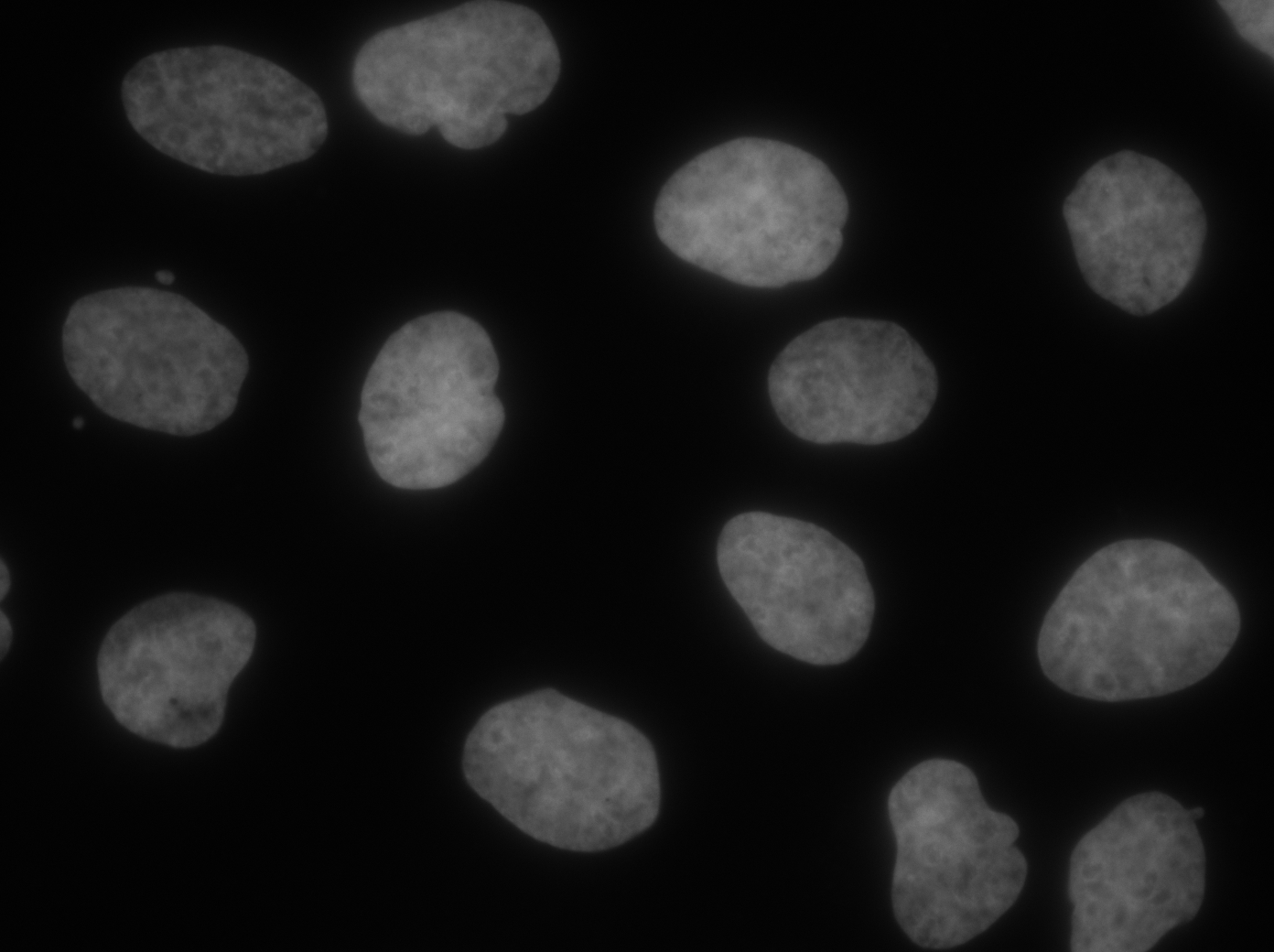

Supplement: Supplementary file 8 — Source Data [file 41467_2021_24153_MOESM8_ESM.zip › RawData/Main Figures/Fig1/b/U2OSH33SNAP_150J_siHIRA_01_w1DAPI.TIF]

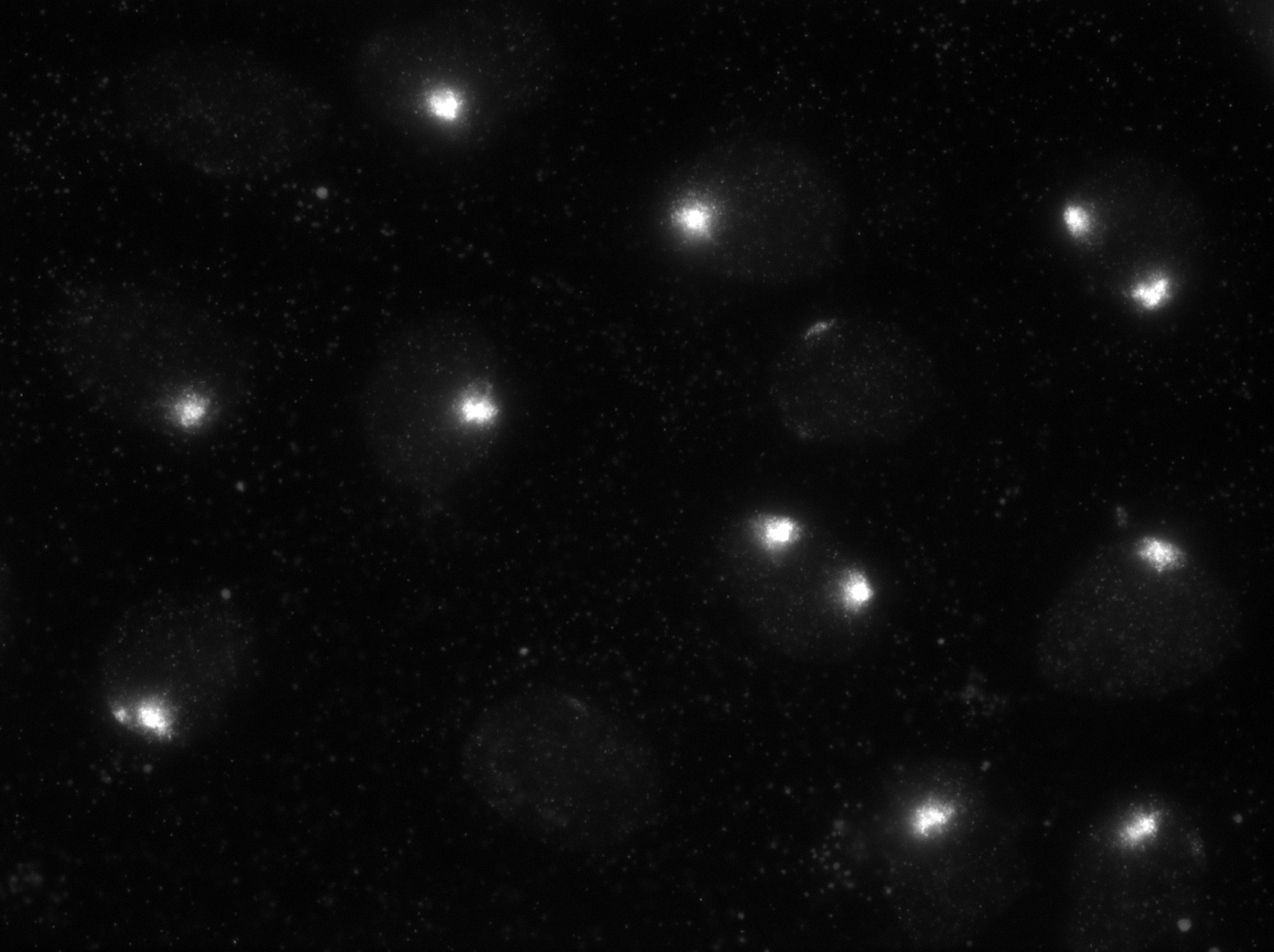

Supplement: Supplementary file 8 — Source Data [file 41467_2021_24153_MOESM8_ESM.zip › RawData/Main Figures/Fig1/b/U2OSH33SNAP_150J_siHIRA_01_w2GFP.TIF]

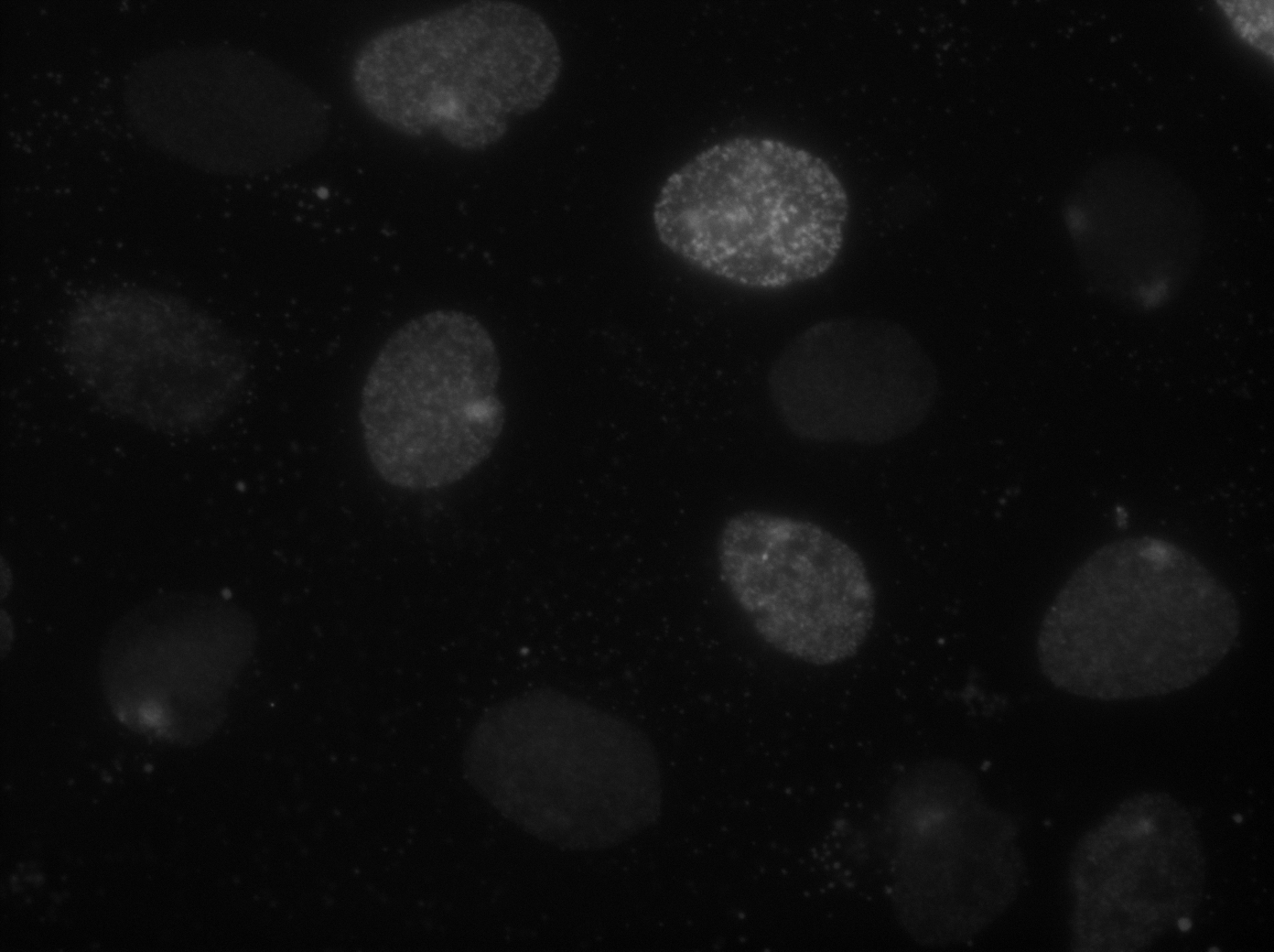

Supplement: Supplementary file 8 — Source Data [file 41467_2021_24153_MOESM8_ESM.zip › RawData/Main Figures/Fig1/b/U2OSH33SNAP_150J_siHIRA_01_w3CY3.TIF]

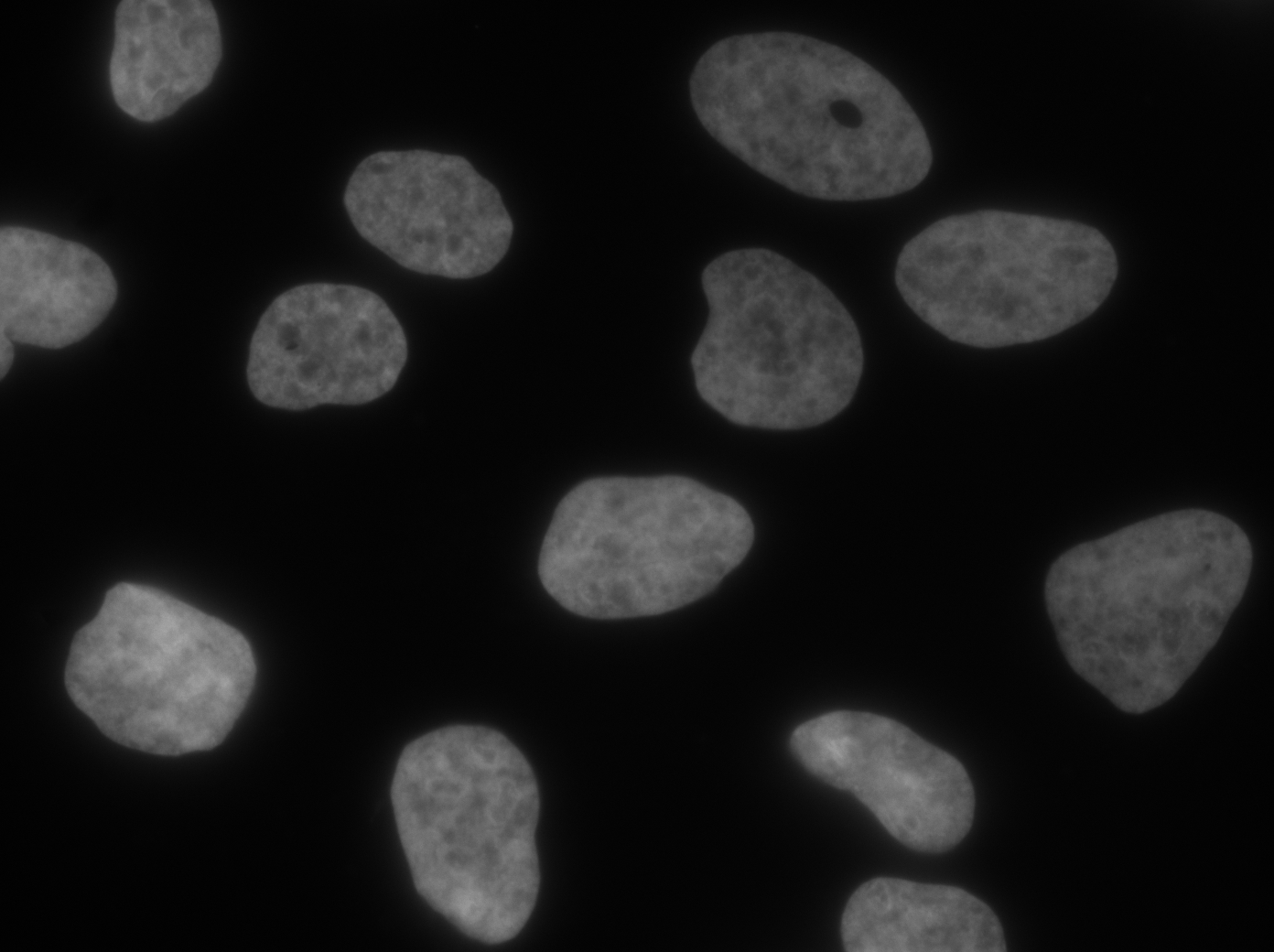

Supplement: Supplementary file 8 — Source Data [file 41467_2021_24153_MOESM8_ESM.zip › RawData/Main Figures/Fig1/b/U2OSH33SNAP_150J_siLUC_13_w1DAPI.TIF]

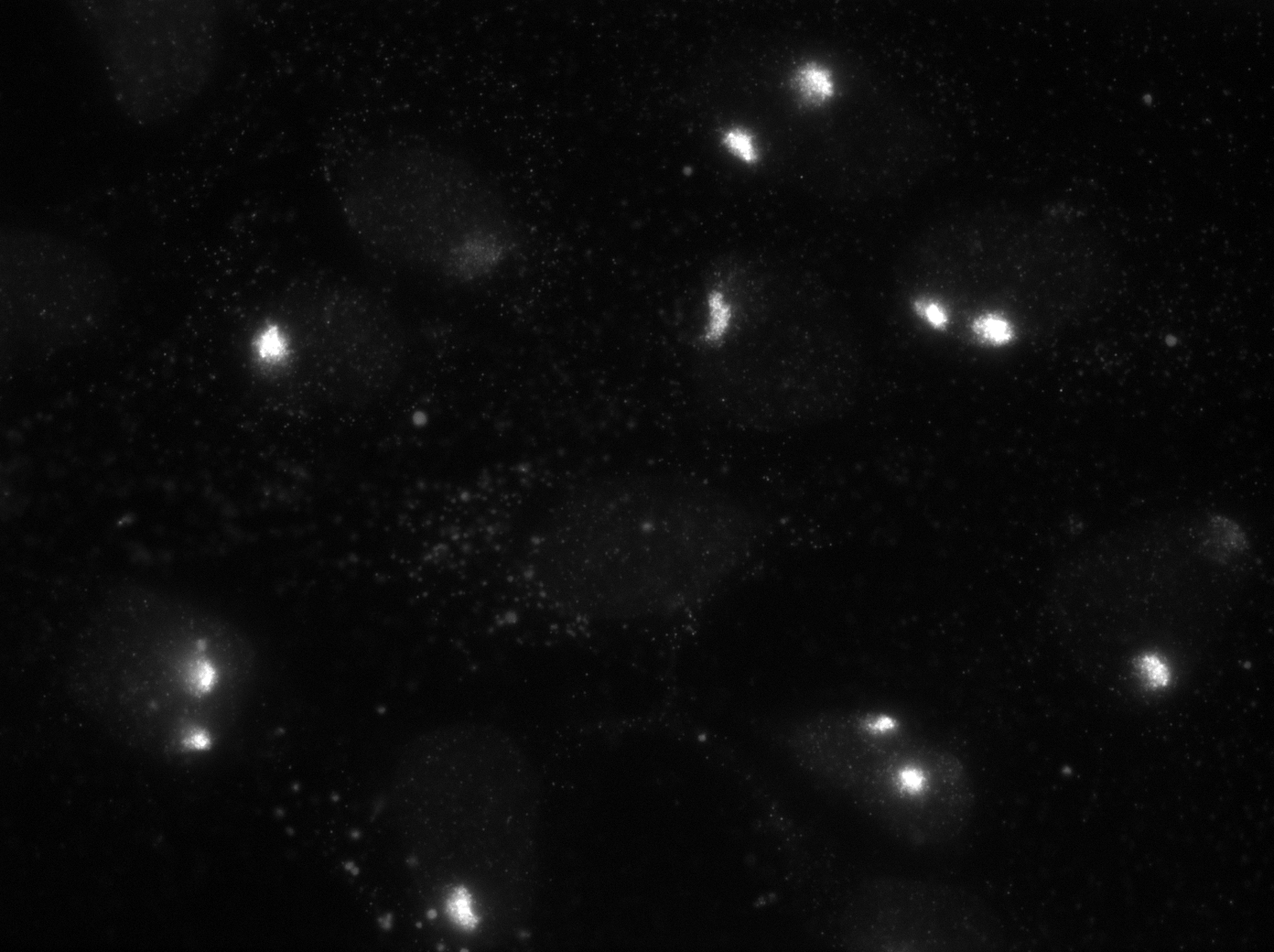

Supplement: Supplementary file 8 — Source Data [file 41467_2021_24153_MOESM8_ESM.zip › RawData/Main Figures/Fig1/b/U2OSH33SNAP_150J_siLUC_13_w2GFP.TIF]

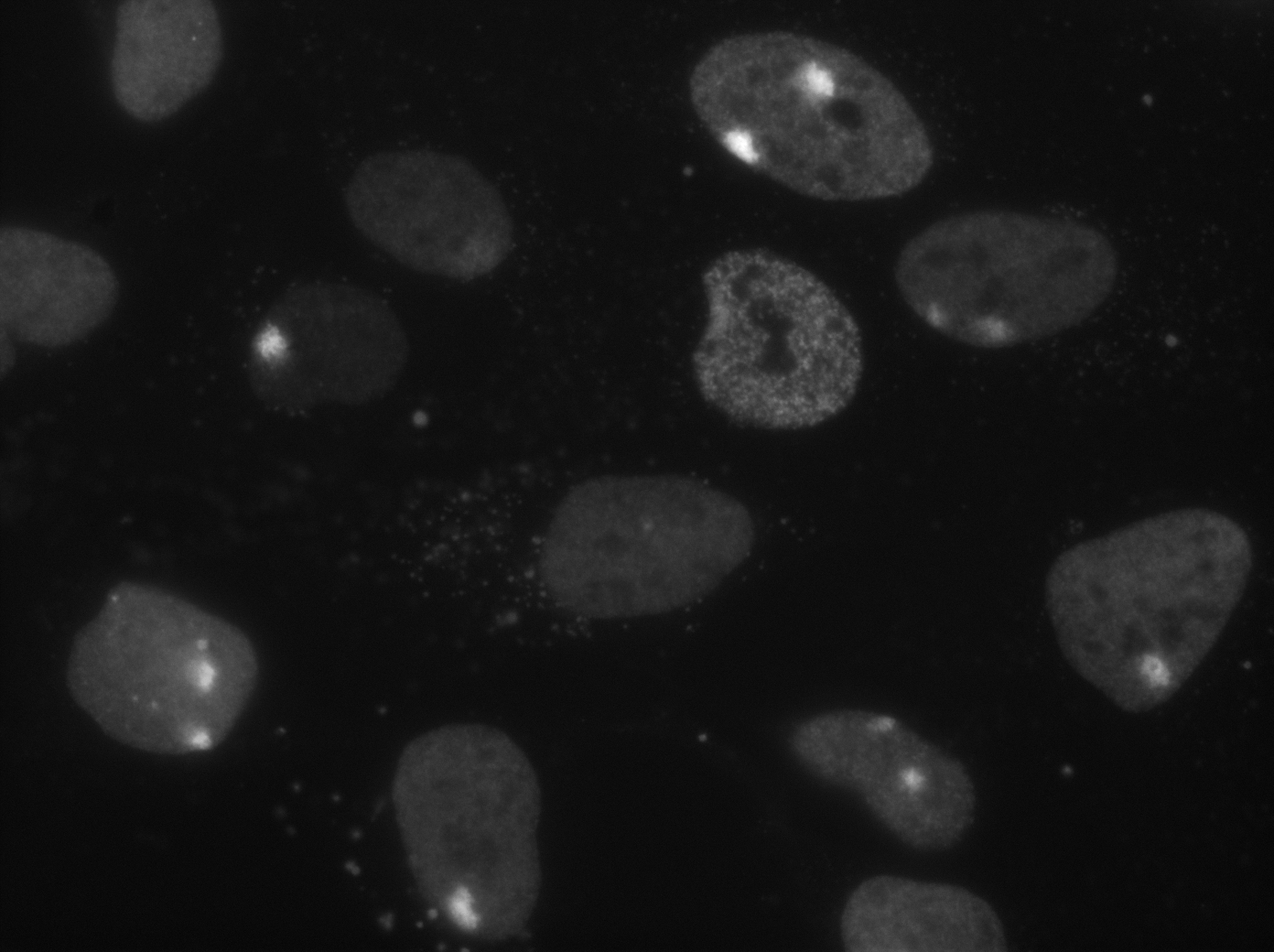

Supplement: Supplementary file 8 — Source Data [file 41467_2021_24153_MOESM8_ESM.zip › RawData/Main Figures/Fig1/b/U2OSH33SNAP_150J_siLUC_13_w3CY3.TIF]

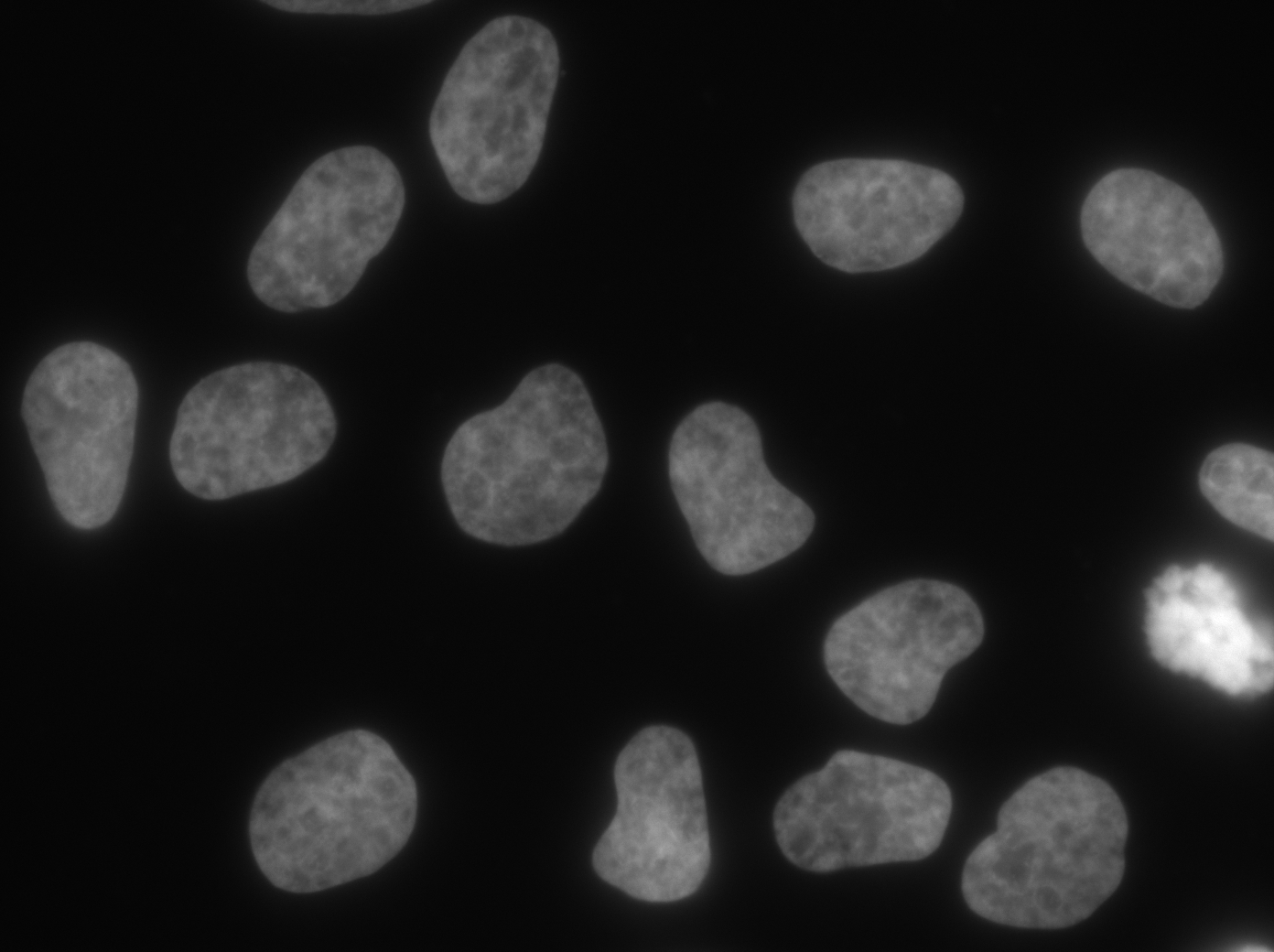

Supplement: Supplementary file 8 — Source Data [file 41467_2021_24153_MOESM8_ESM.zip › RawData/Main Figures/Fig1/b/U2OSH33SNAP_150J_siUBN1_09_w1DAPI.TIF]

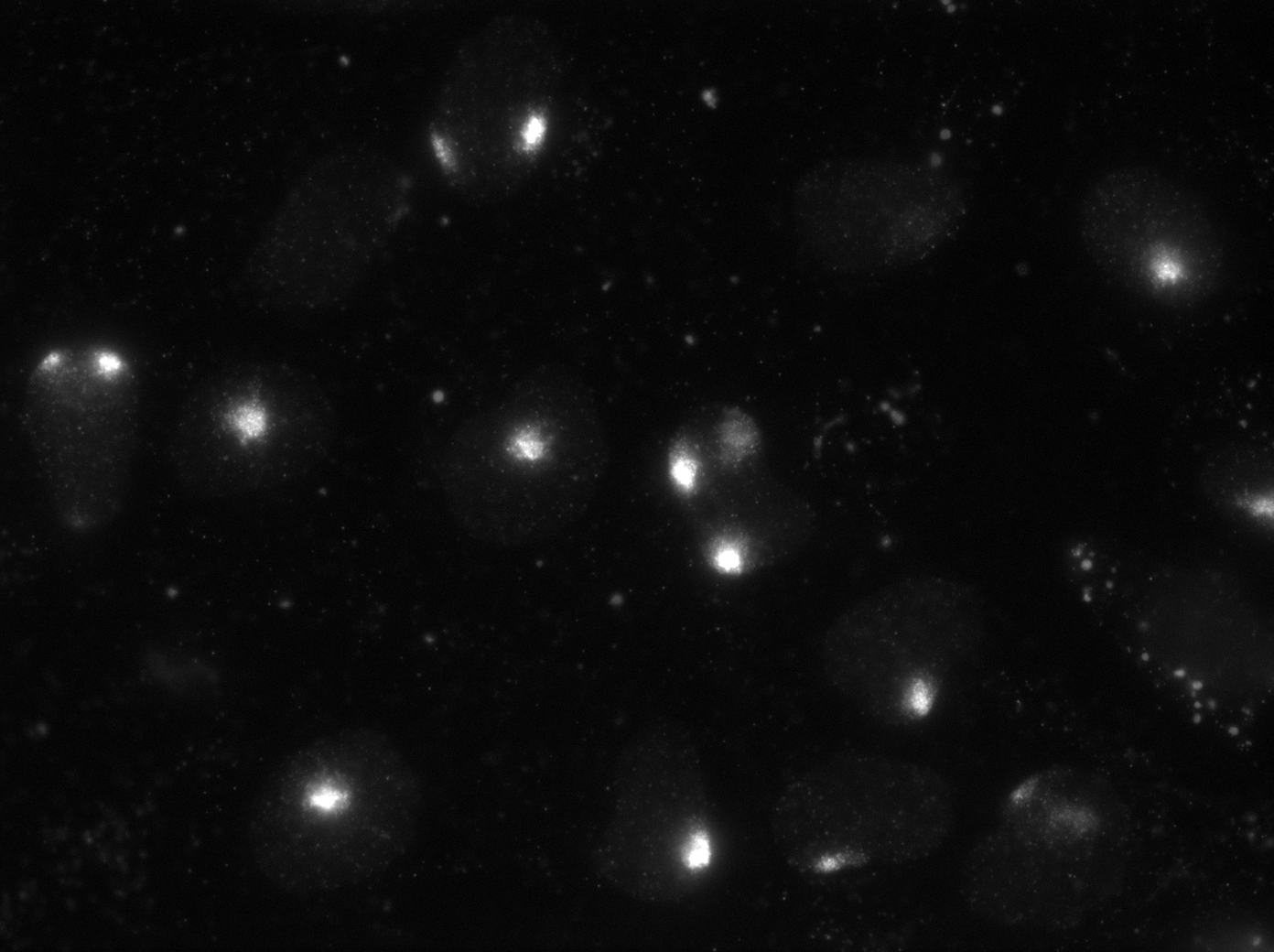

Supplement: Supplementary file 8 — Source Data [file 41467_2021_24153_MOESM8_ESM.zip › RawData/Main Figures/Fig1/b/U2OSH33SNAP_150J_siUBN1_09_w2GFP.TIF]

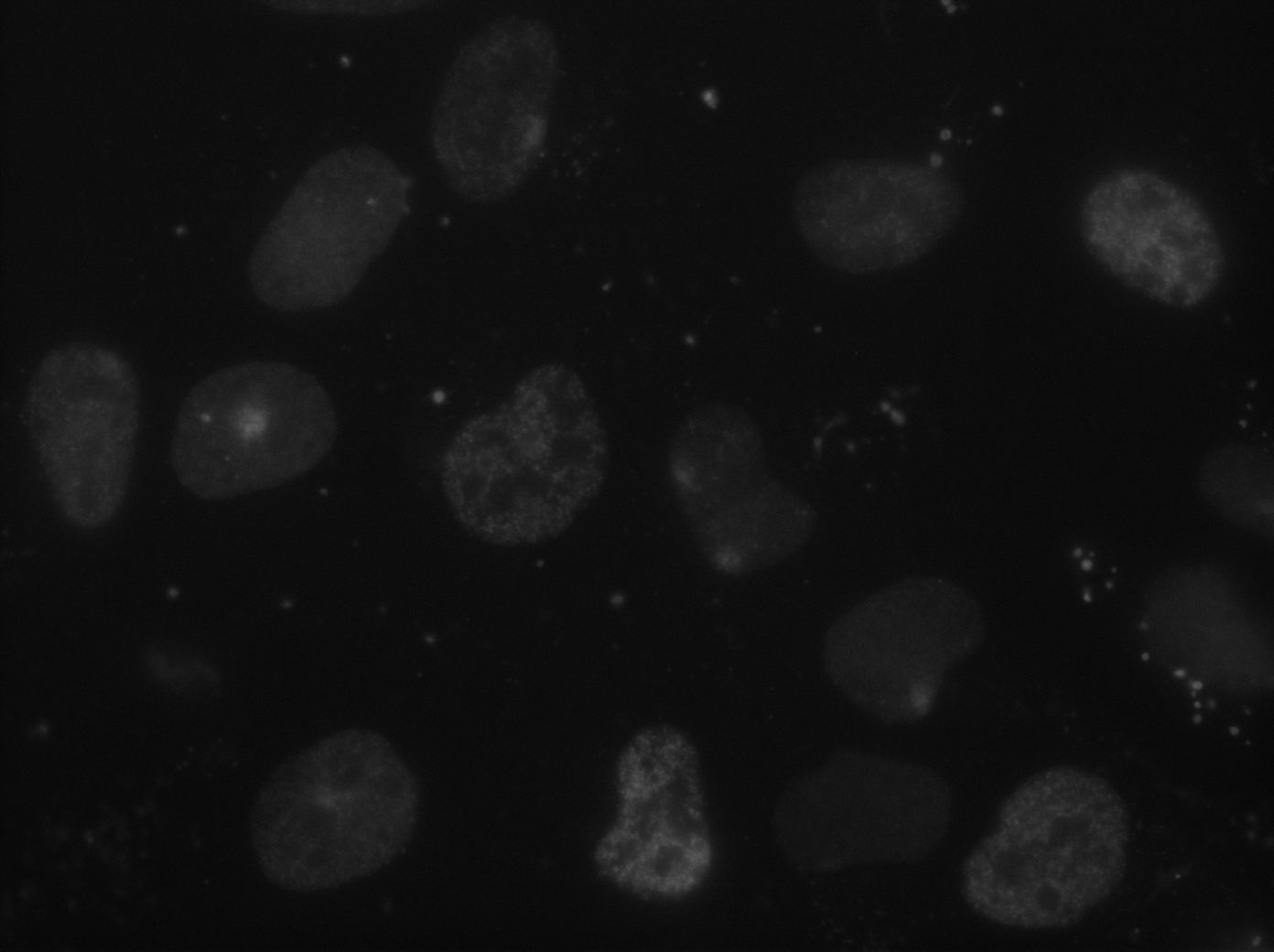

Supplement: Supplementary file 8 — Source Data [file 41467_2021_24153_MOESM8_ESM.zip › RawData/Main Figures/Fig1/b/U2OSH33SNAP_150J_siUBN1_09_w3CY3.TIF]

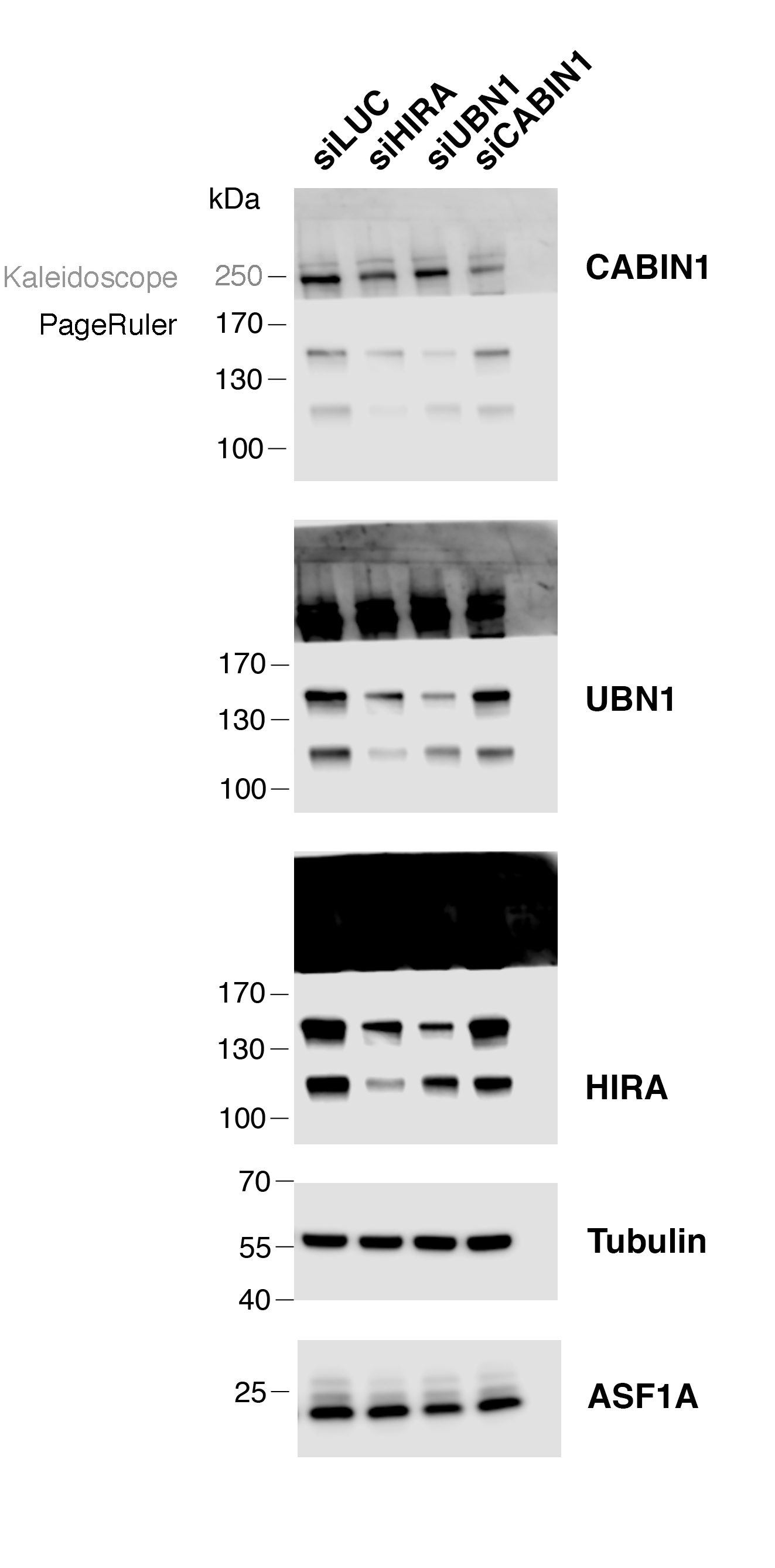

Supplement: Supplementary file 8 — Source Data [file 41467_2021_24153_MOESM8_ESM.zip › RawData/Main Figures/Fig1/b/WB.tif]

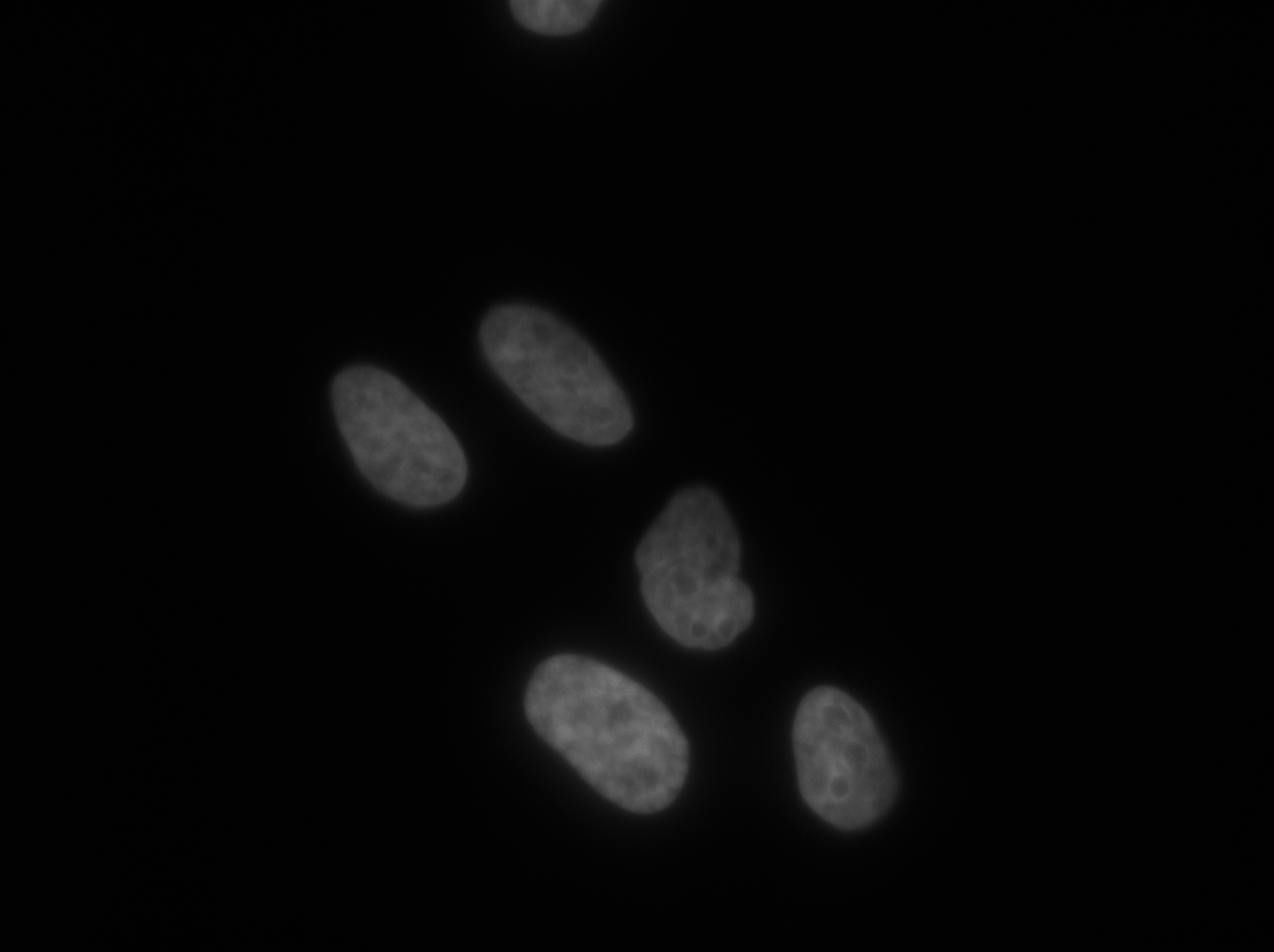

Supplement: Supplementary file 8 — Source Data [file 41467_2021_24153_MOESM8_ESM.zip › RawData/Main Figures/Fig1/c/QCP_siH3.3_1_03_w1DAPI.TIF]

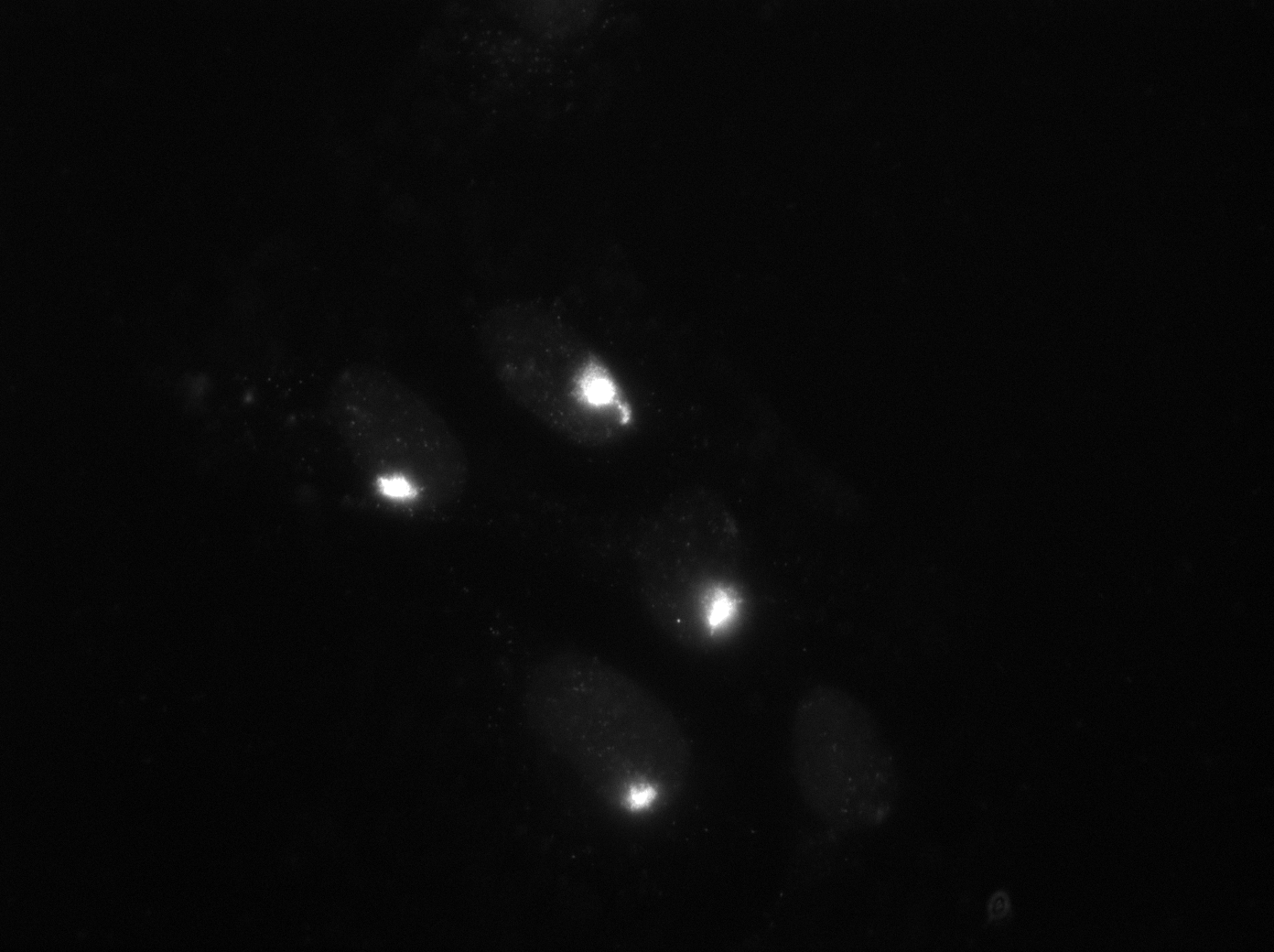

Supplement: Supplementary file 8 — Source Data [file 41467_2021_24153_MOESM8_ESM.zip › RawData/Main Figures/Fig1/c/QCP_siH3.3_1_03_w2GFP.TIF]

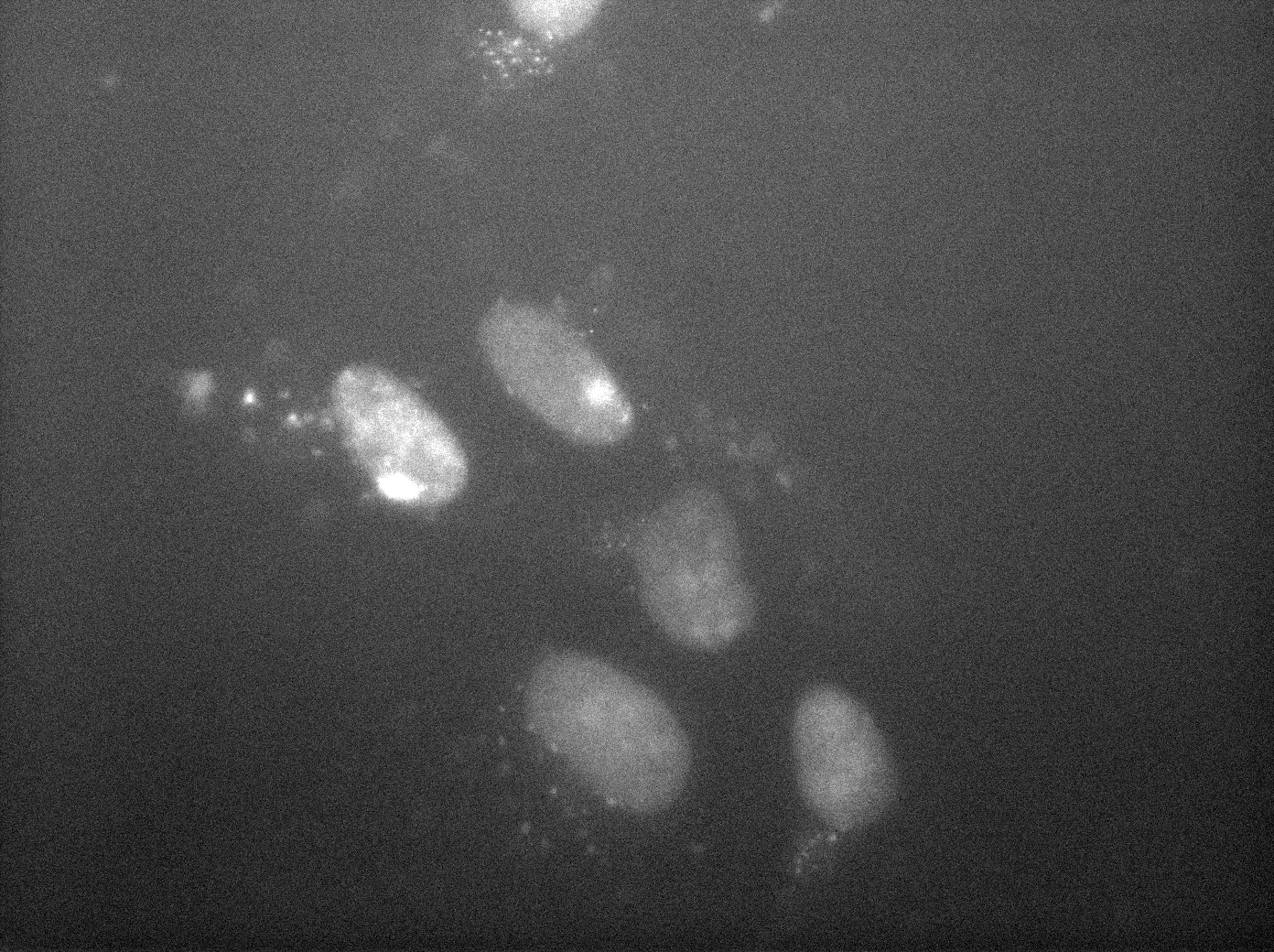

Supplement: Supplementary file 8 — Source Data [file 41467_2021_24153_MOESM8_ESM.zip › RawData/Main Figures/Fig1/c/QCP_siH3.3_1_03_w3CY3.TIF]

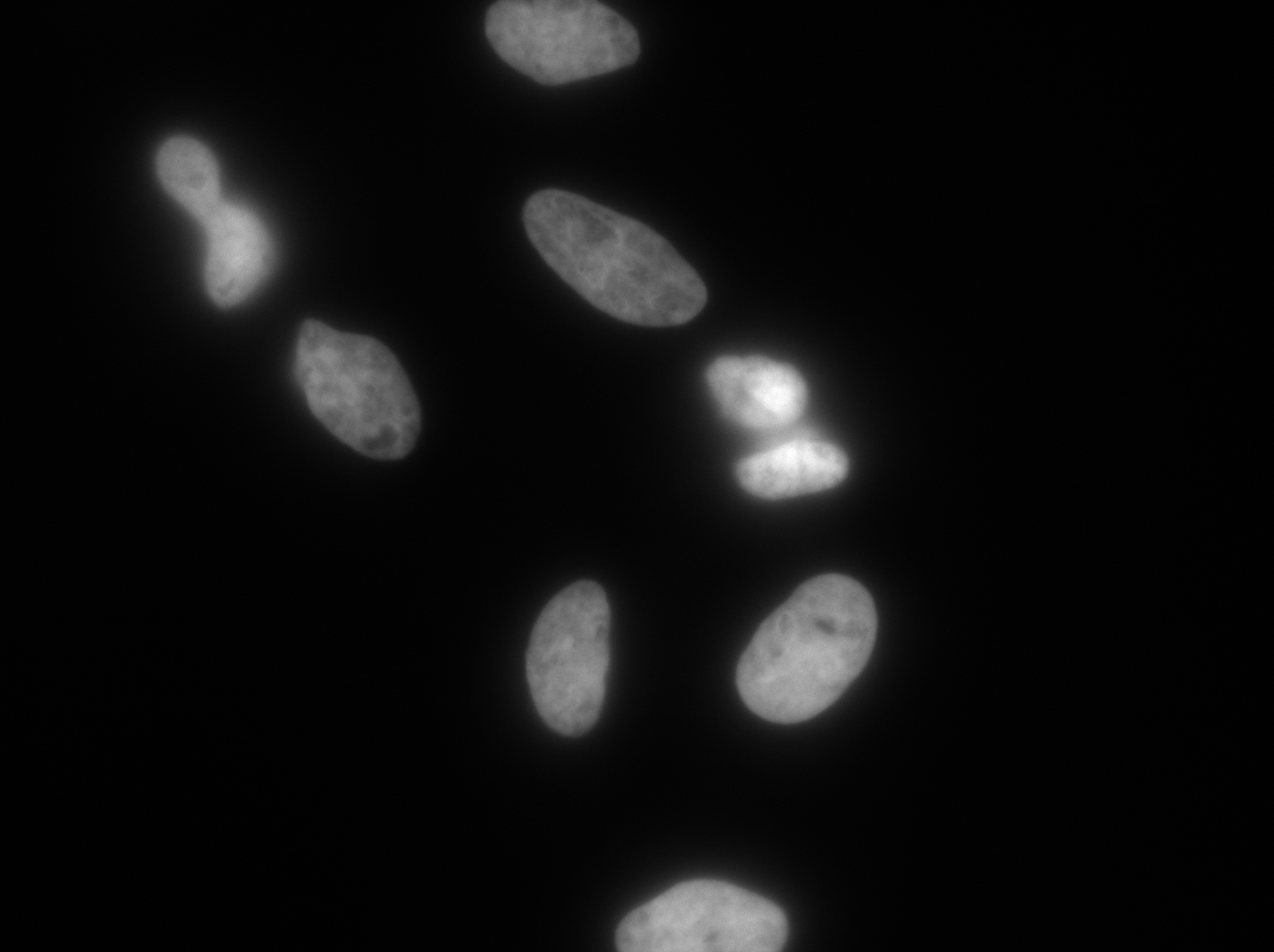

Supplement: Supplementary file 8 — Source Data [file 41467_2021_24153_MOESM8_ESM.zip › RawData/Main Figures/Fig1/c/QCP_siLUC_2_03_w1DAPI.TIF]

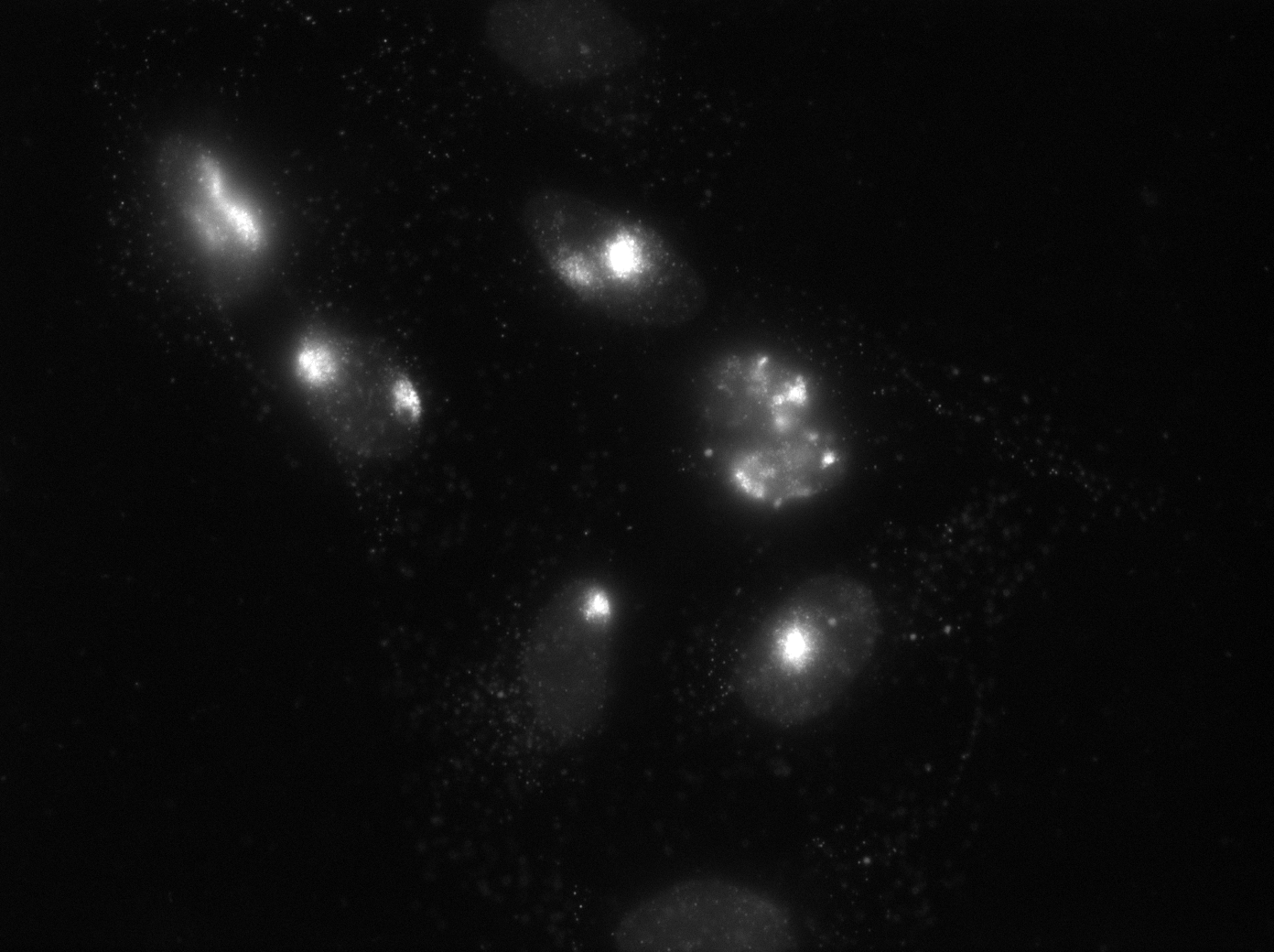

Supplement: Supplementary file 8 — Source Data [file 41467_2021_24153_MOESM8_ESM.zip › RawData/Main Figures/Fig1/c/QCP_siLUC_2_03_w2GFP.TIF]

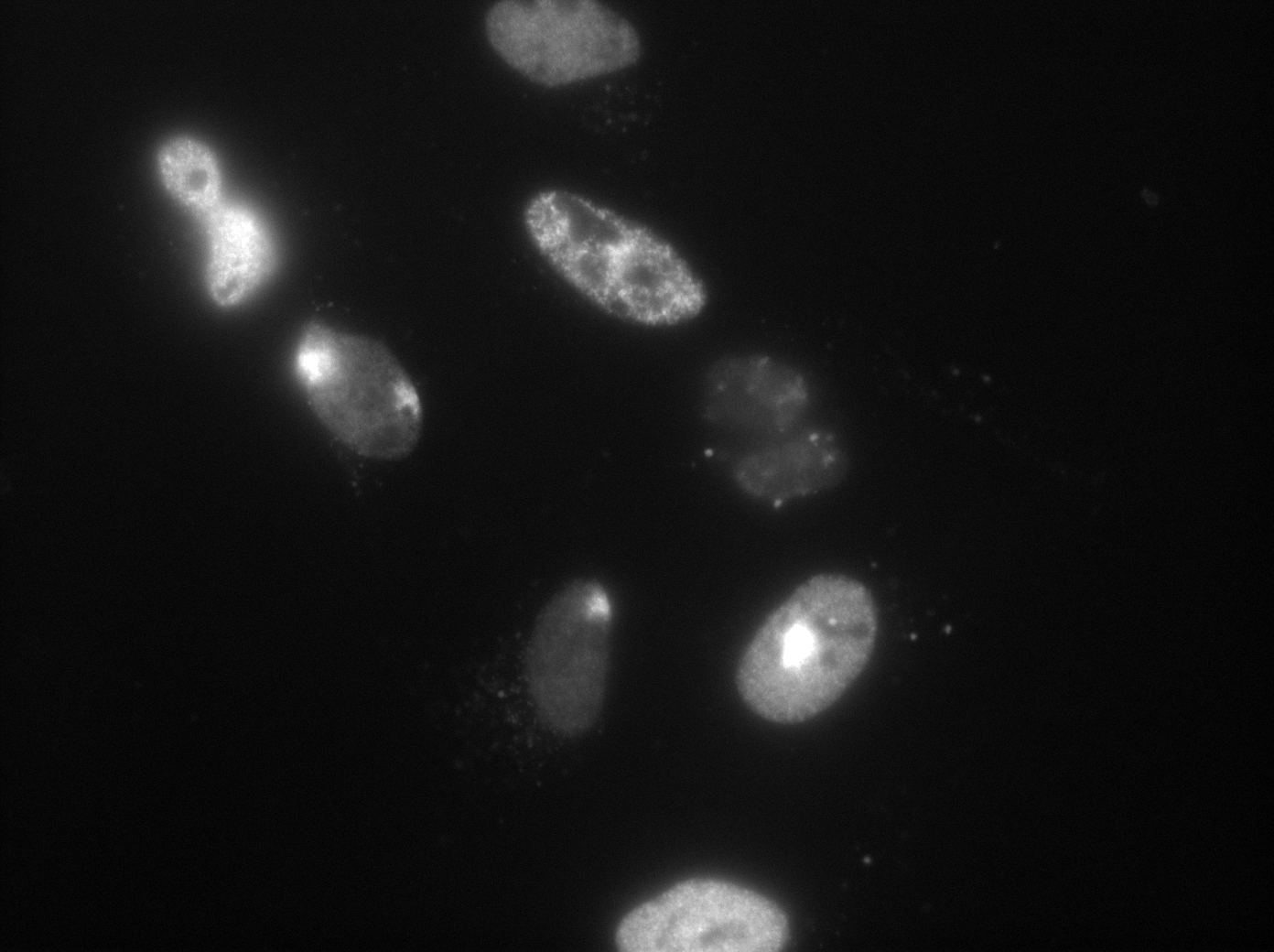

Supplement: Supplementary file 8 — Source Data [file 41467_2021_24153_MOESM8_ESM.zip › RawData/Main Figures/Fig1/c/QCP_siLUC_2_03_w3CY3.TIF]

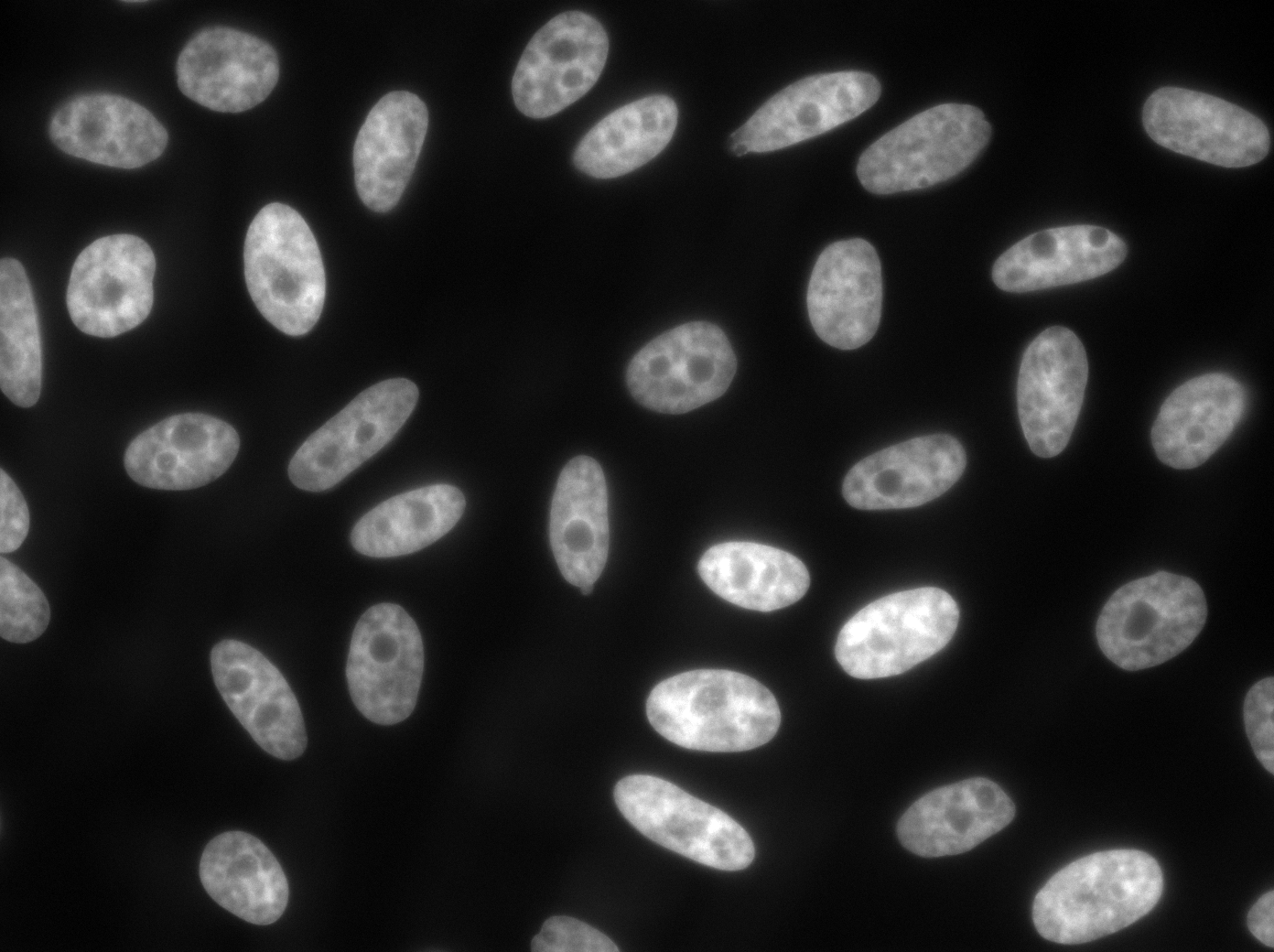

Supplement: Supplementary file 8 — Source Data [file 41467_2021_24153_MOESM8_ESM.zip › RawData/Main Figures/Fig1/d/HeLa_EU_siH33_0h_06_w1DAPI.TIF]

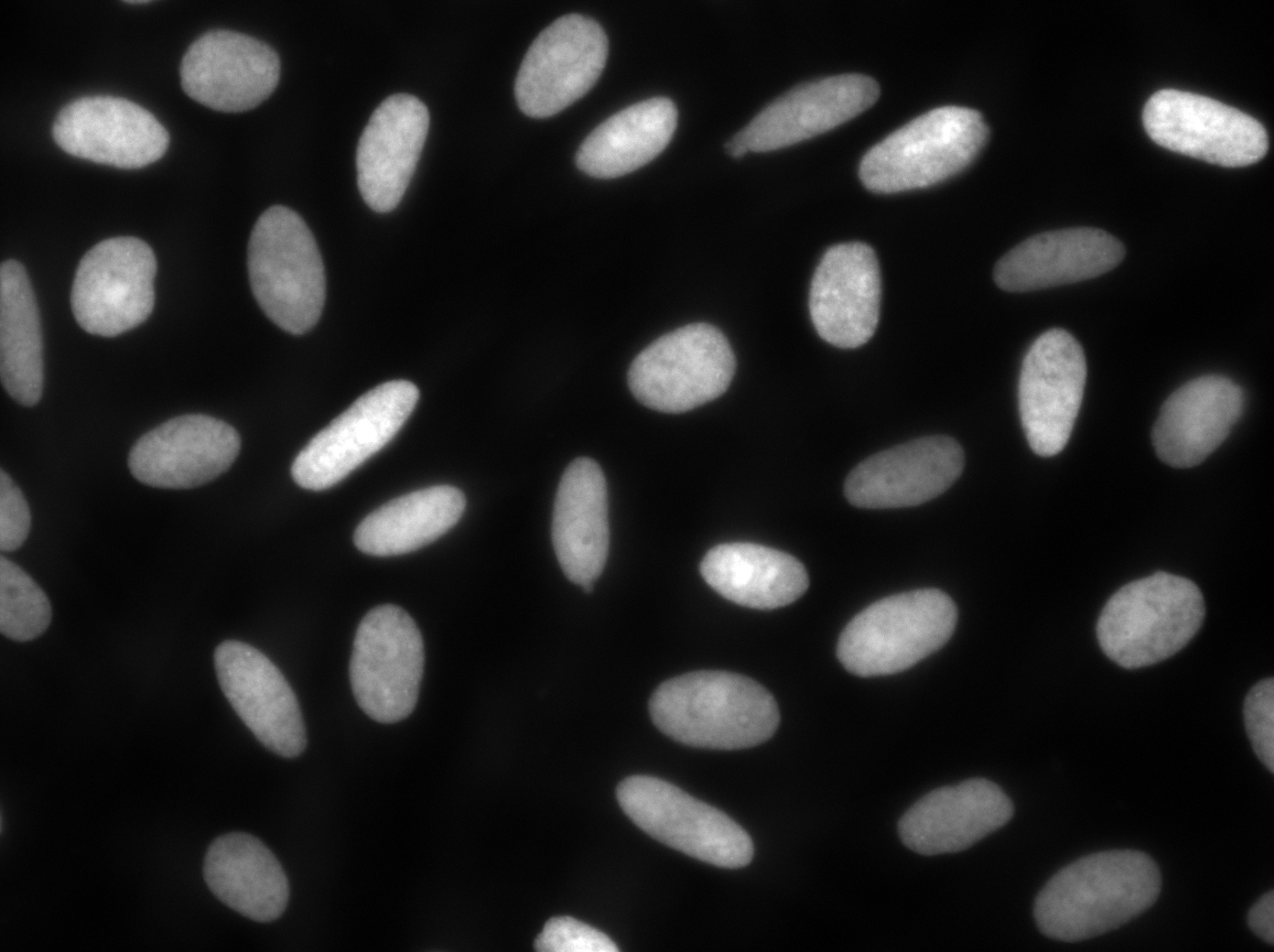

Supplement: Supplementary file 8 — Source Data [file 41467_2021_24153_MOESM8_ESM.zip › RawData/Main Figures/Fig1/d/HeLa_EU_siH33_0h_06_w2TX.TIF]

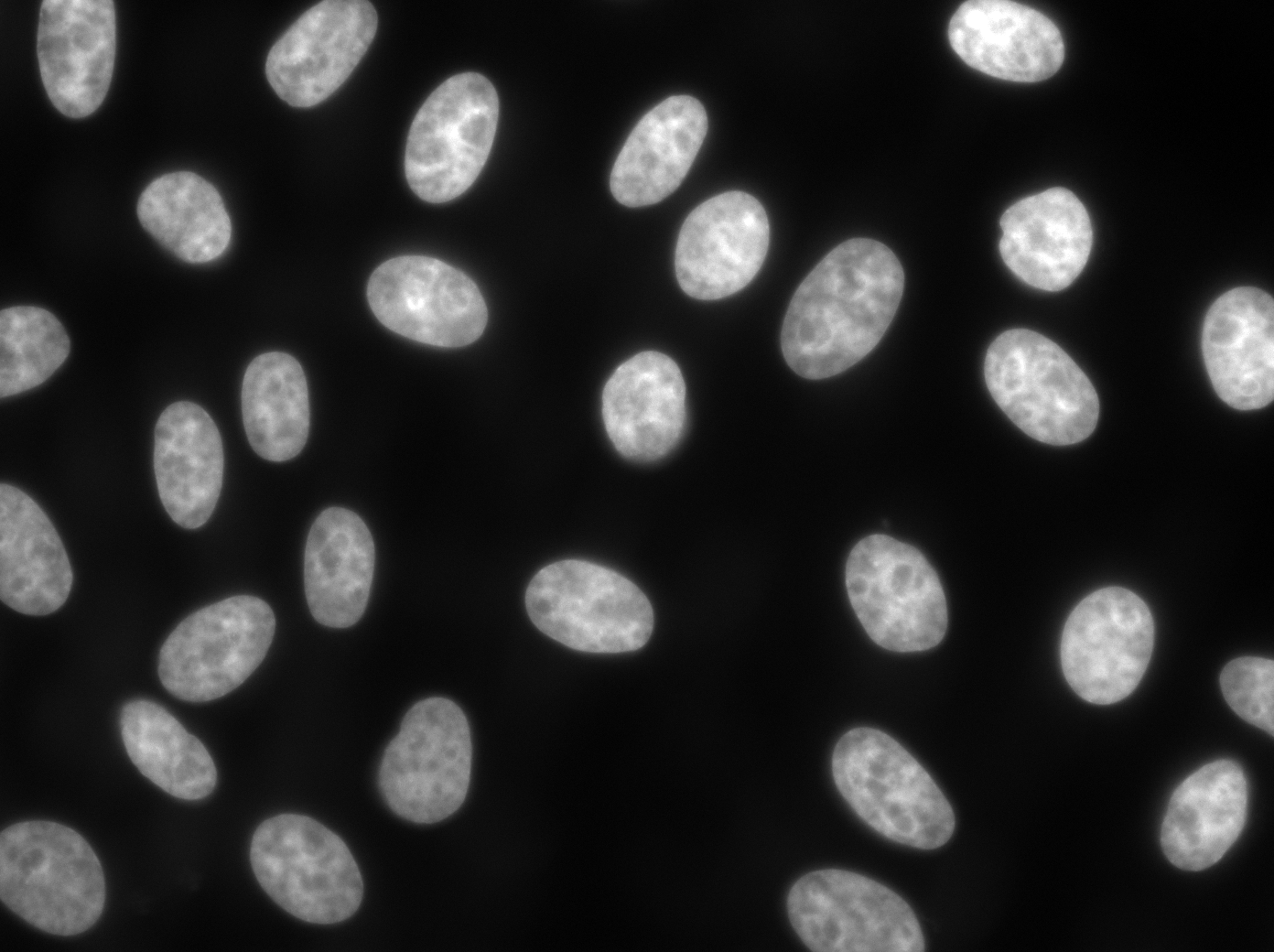

Supplement: Supplementary file 8 — Source Data [file 41467_2021_24153_MOESM8_ESM.zip › RawData/Main Figures/Fig1/d/HeLa_EU_siH33_24h_08_w1DAPI.TIF]

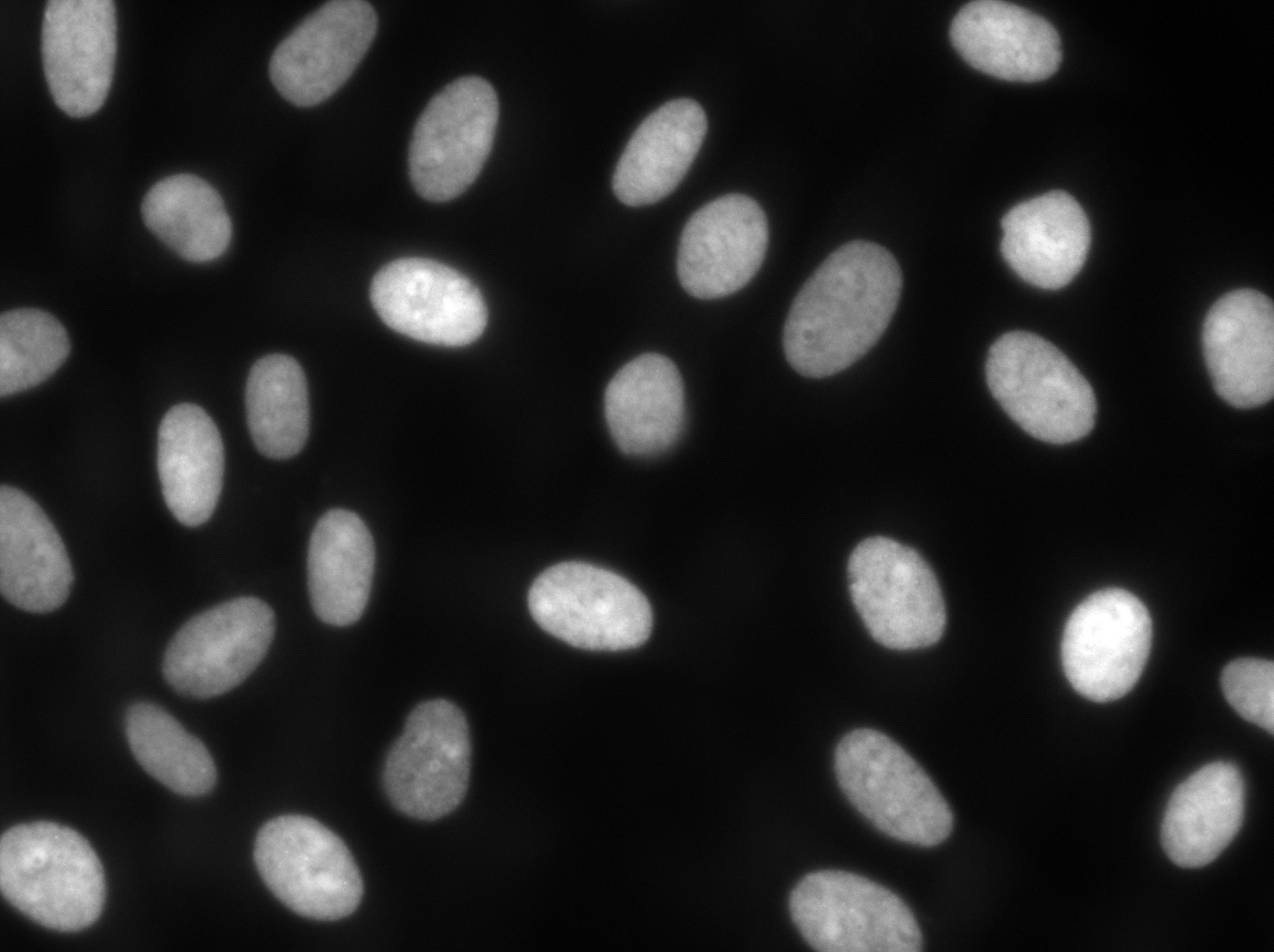

Supplement: Supplementary file 8 — Source Data [file 41467_2021_24153_MOESM8_ESM.zip › RawData/Main Figures/Fig1/d/HeLa_EU_siH33_24h_08_w2TX.TIF]

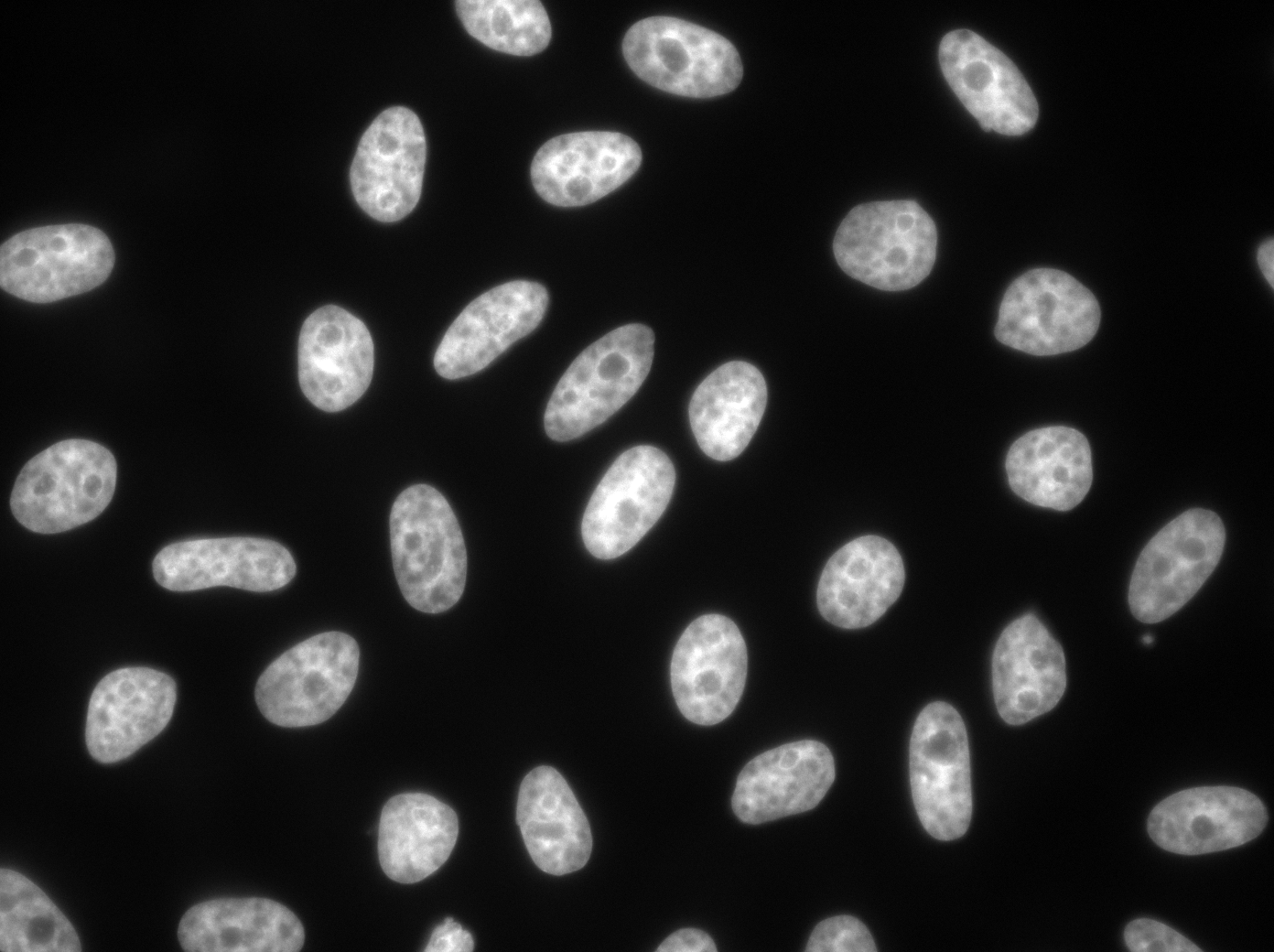

Supplement: Supplementary file 8 — Source Data [file 41467_2021_24153_MOESM8_ESM.zip › RawData/Main Figures/Fig1/d/HeLa_EU_siH33_2h_03_w1DAPI.TIF]

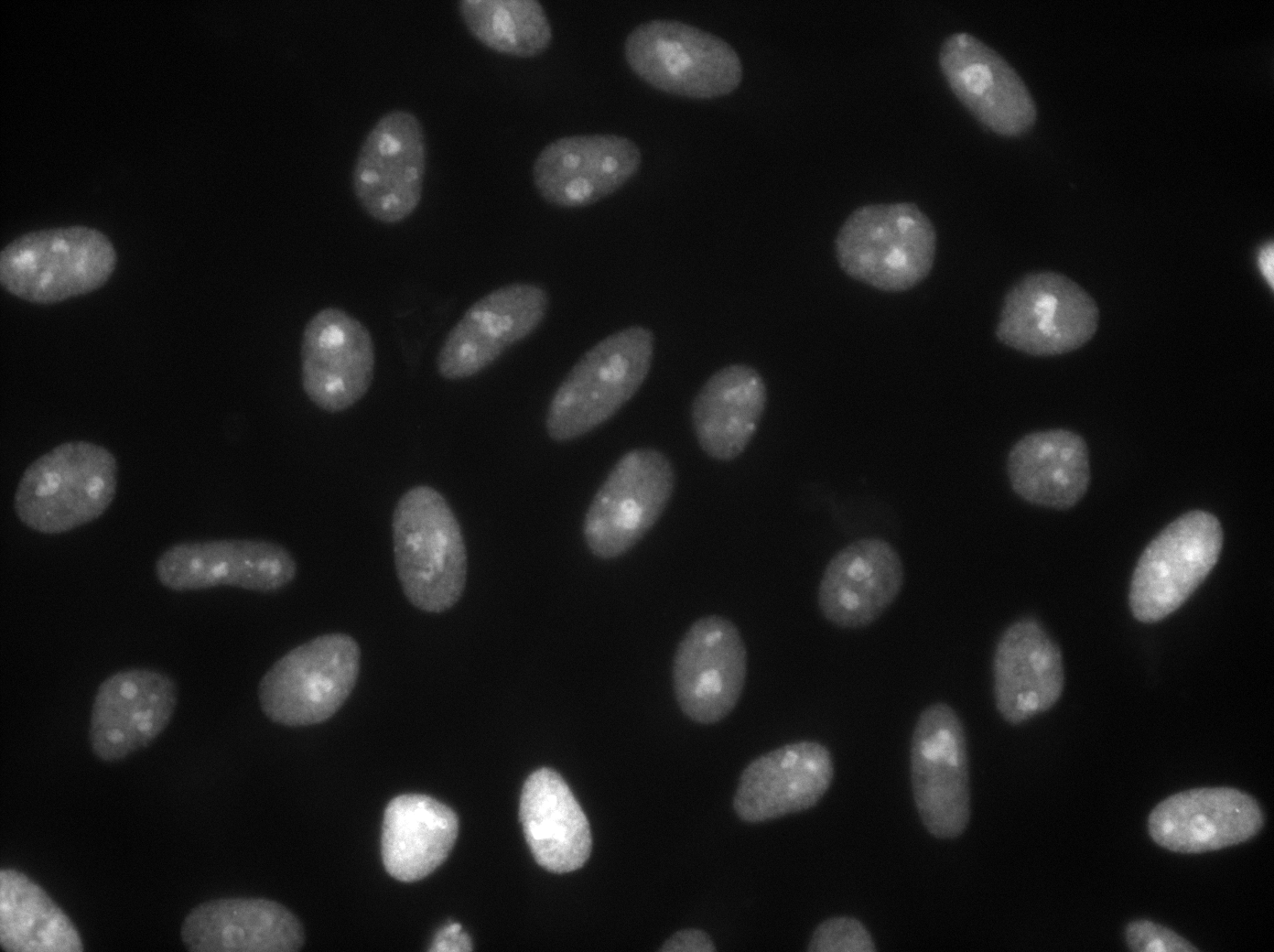

Supplement: Supplementary file 8 — Source Data [file 41467_2021_24153_MOESM8_ESM.zip › RawData/Main Figures/Fig1/d/HeLa_EU_siH33_2h_03_w2TX.TIF]

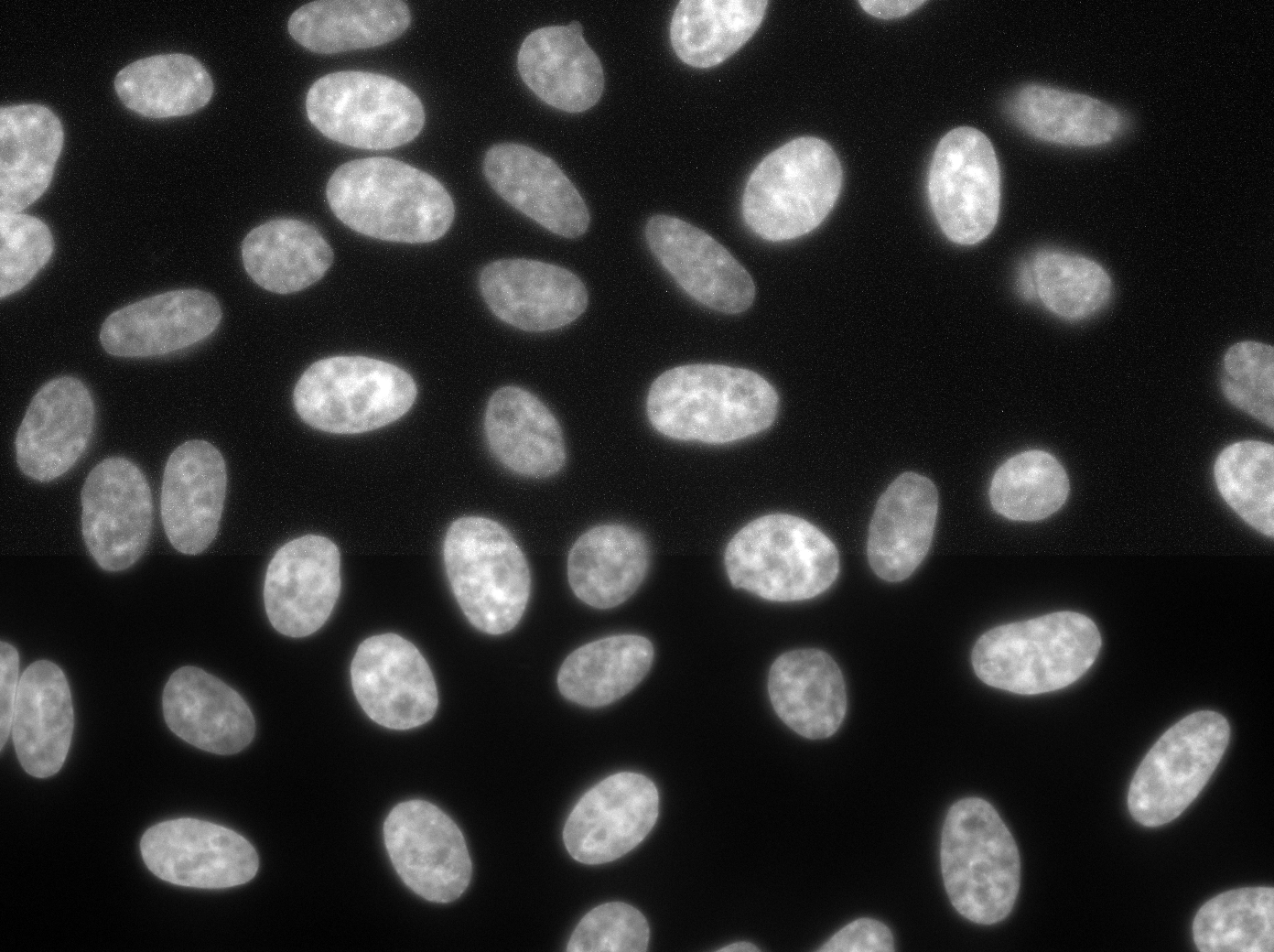

Supplement: Supplementary file 8 — Source Data [file 41467_2021_24153_MOESM8_ESM.zip › RawData/Main Figures/Fig1/d/HeLa_EU_siLUC_0h_03_w1DAPI.TIF]

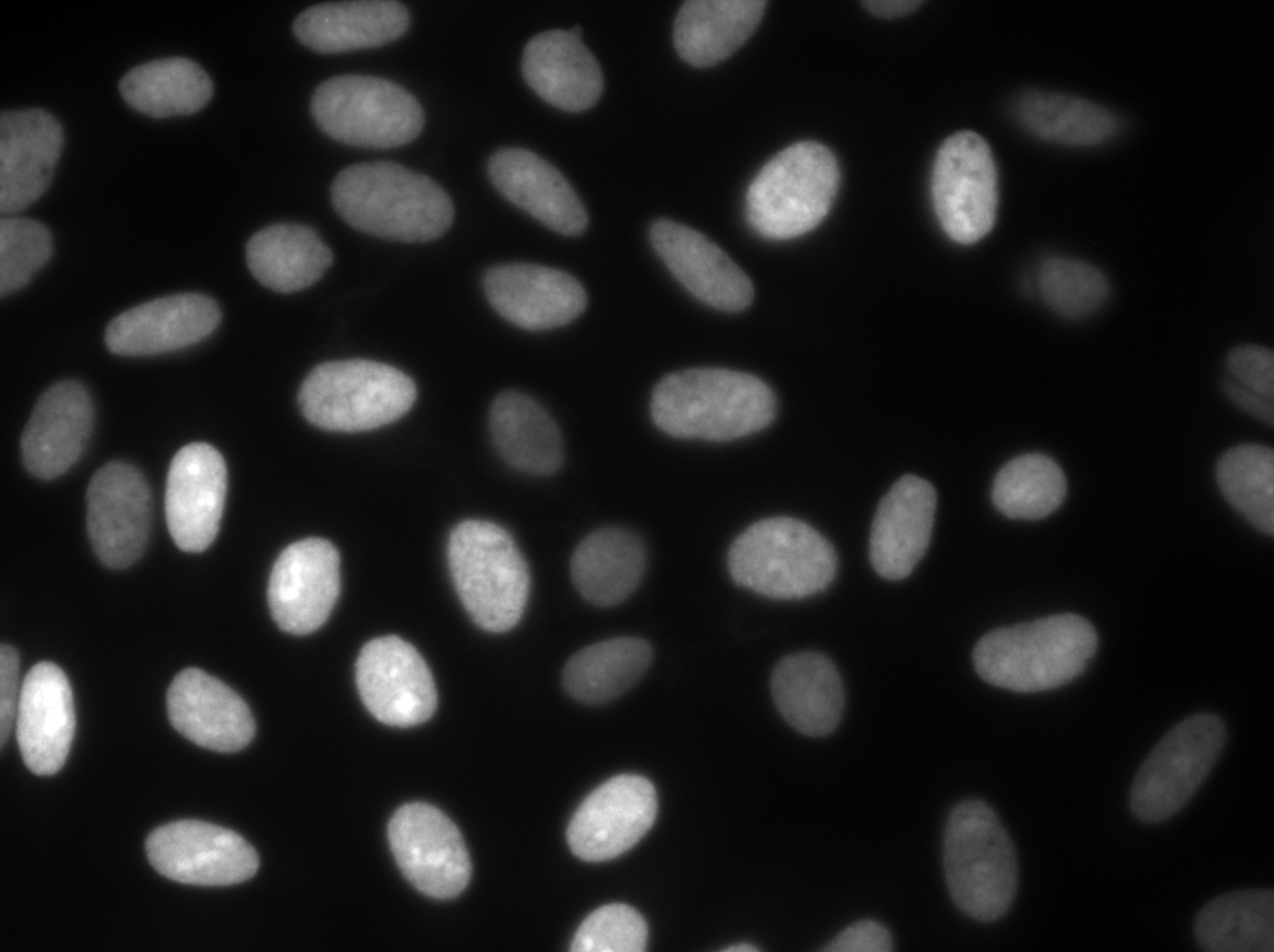

Supplement: Supplementary file 8 — Source Data [file 41467_2021_24153_MOESM8_ESM.zip › RawData/Main Figures/Fig1/d/HeLa_EU_siLUC_0h_03_w2TX.TIF]

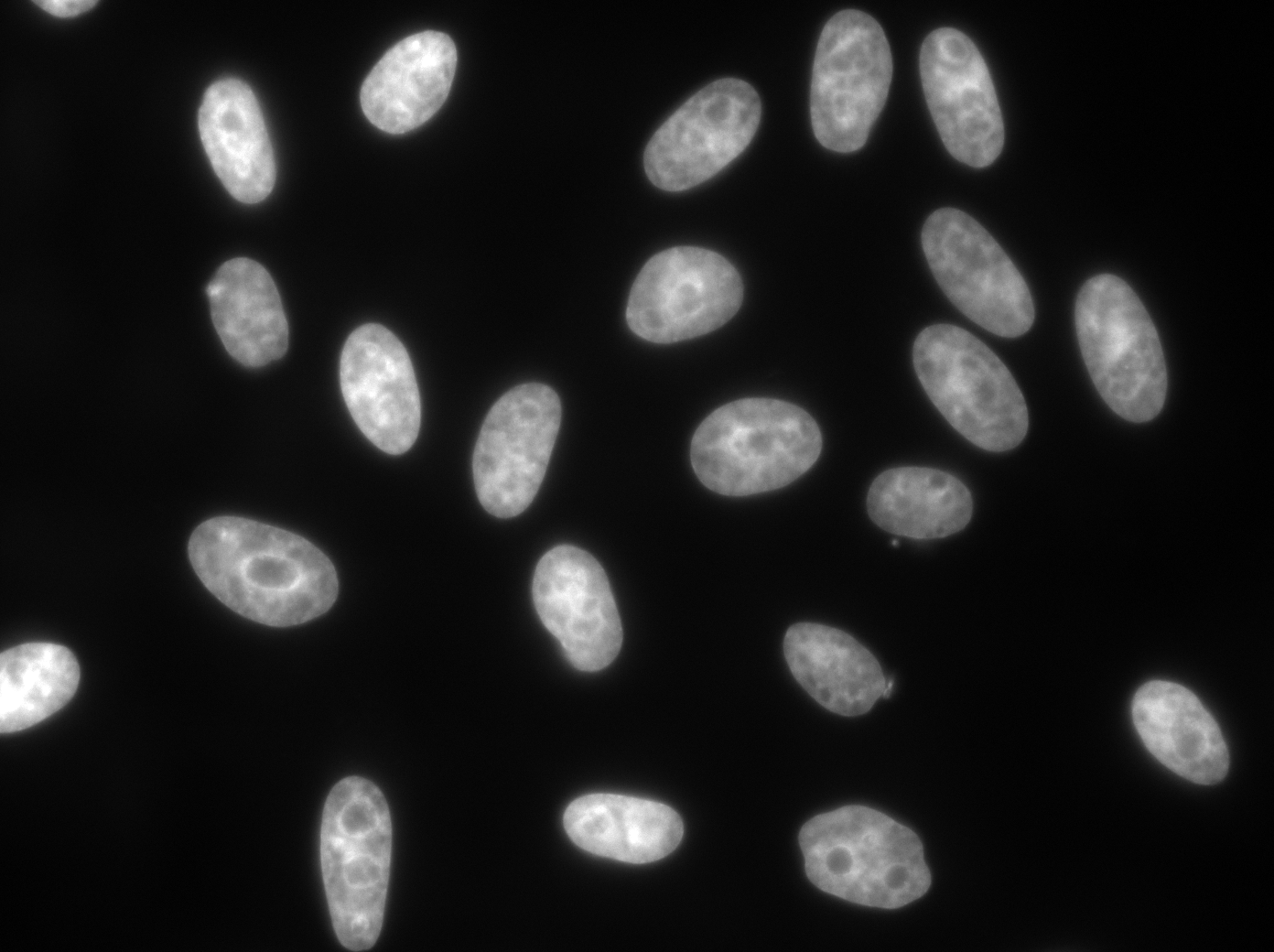

Supplement: Supplementary file 8 — Source Data [file 41467_2021_24153_MOESM8_ESM.zip › RawData/Main Figures/Fig1/d/HeLa_EU_siLUC_24h_02_w1DAPI.TIF]

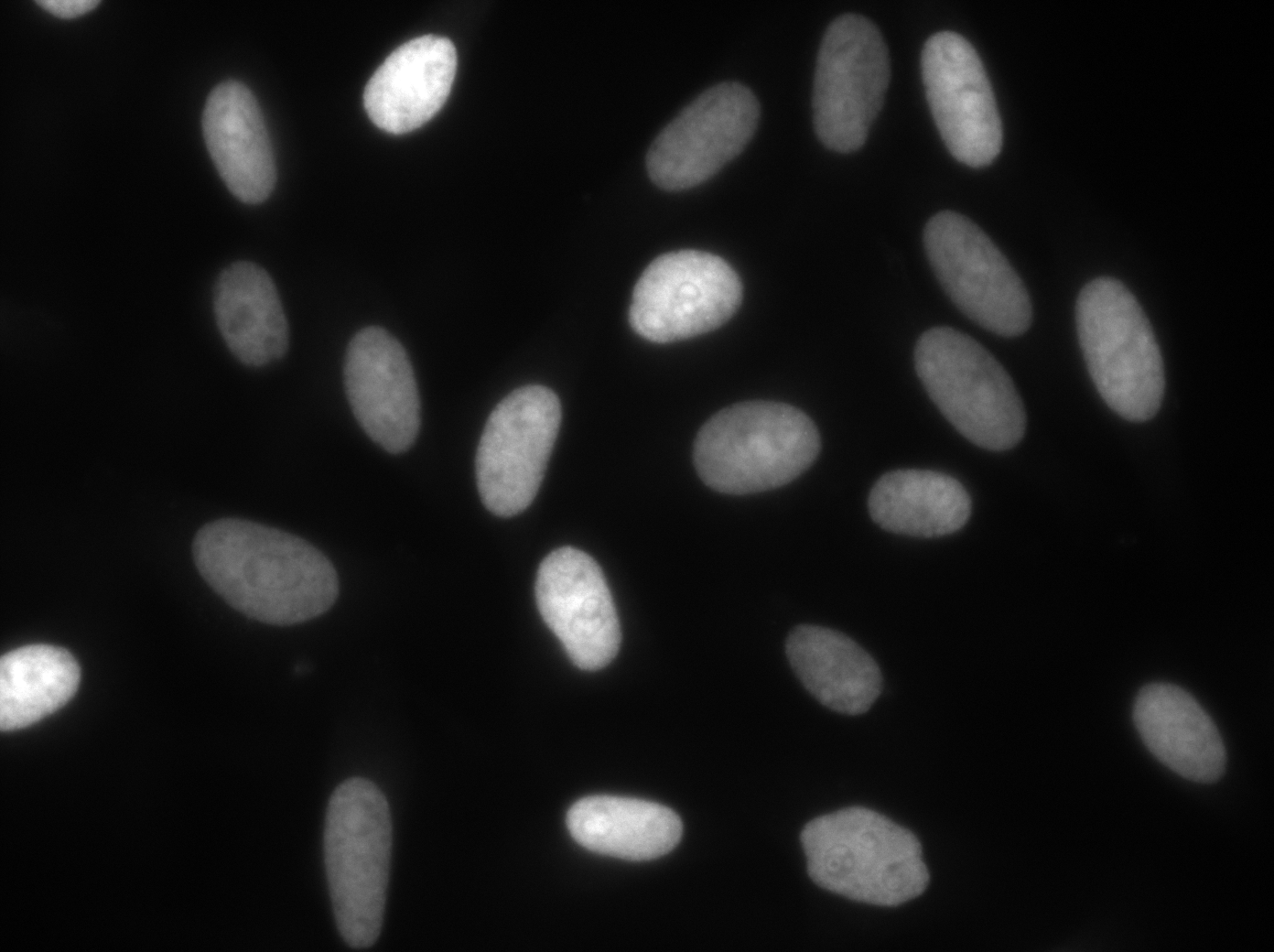

Supplement: Supplementary file 8 — Source Data [file 41467_2021_24153_MOESM8_ESM.zip › RawData/Main Figures/Fig1/d/HeLa_EU_siLUC_24h_02_w2TX.TIF]

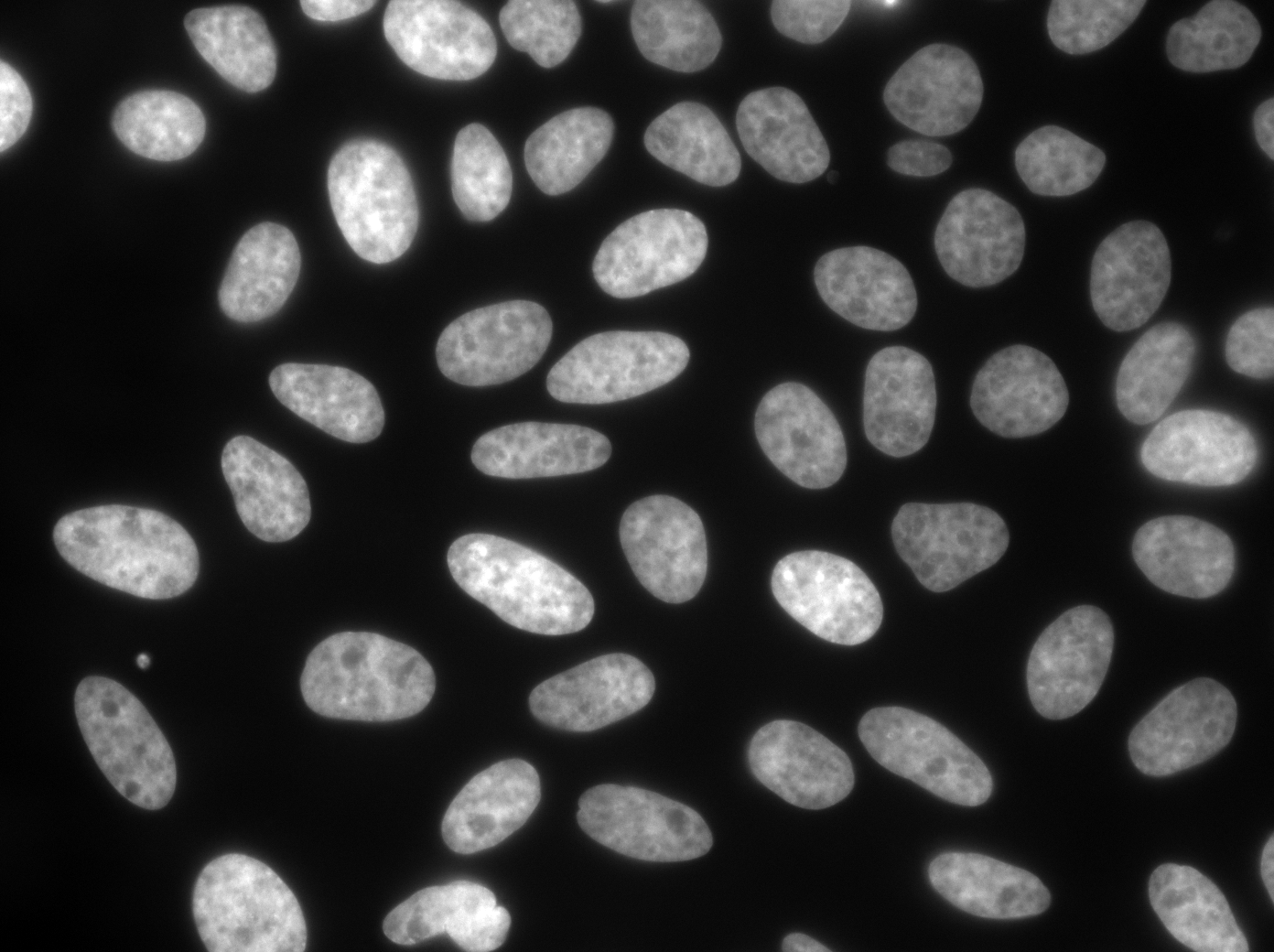

Supplement: Supplementary file 8 — Source Data [file 41467_2021_24153_MOESM8_ESM.zip › RawData/Main Figures/Fig1/d/HeLa_EU_siLUC_2h_01_w1DAPI.TIF]

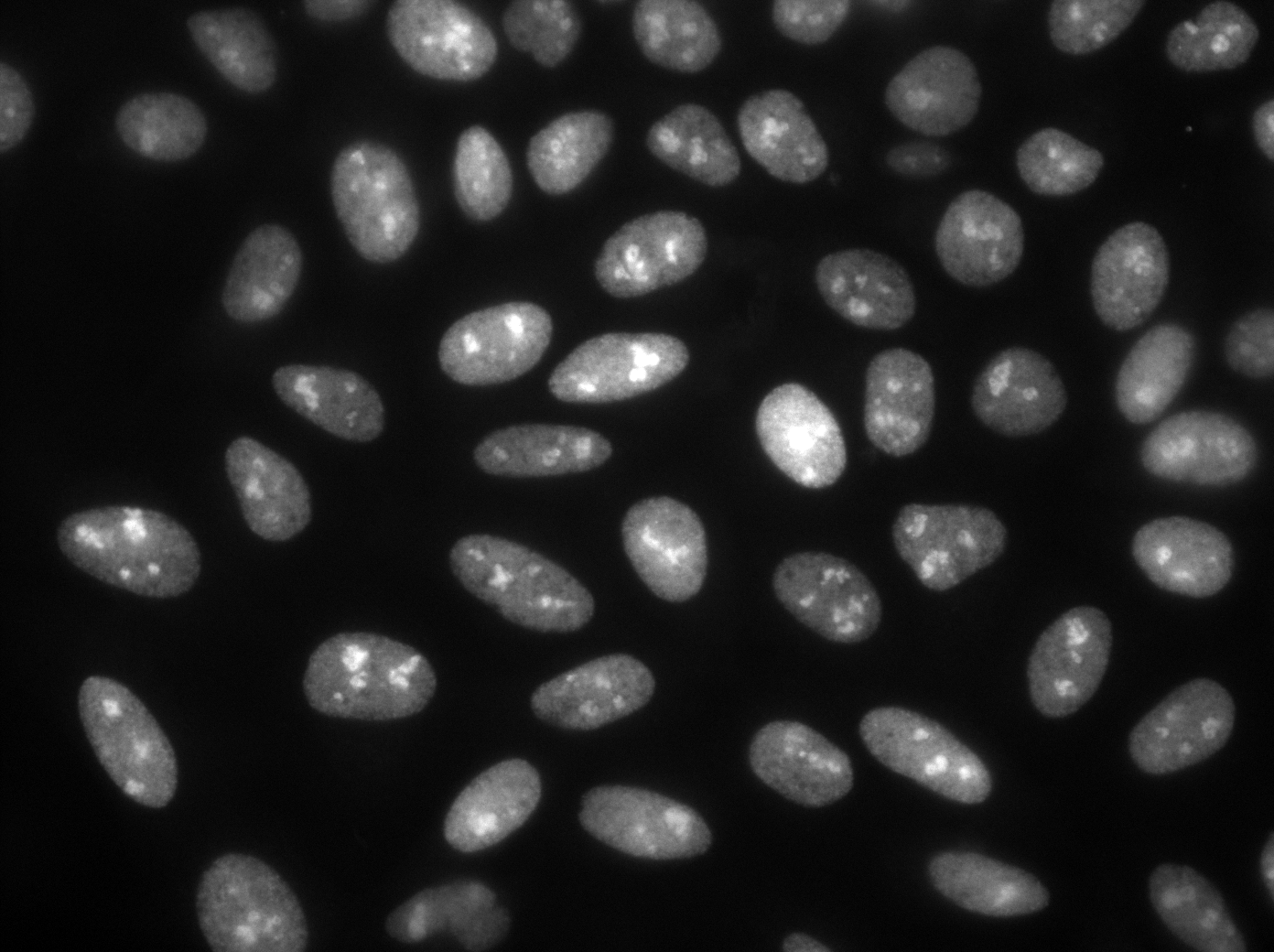

Supplement: Supplementary file 8 — Source Data [file 41467_2021_24153_MOESM8_ESM.zip › RawData/Main Figures/Fig1/d/HeLa_EU_siLUC_2h_01_w2TX.TIF]

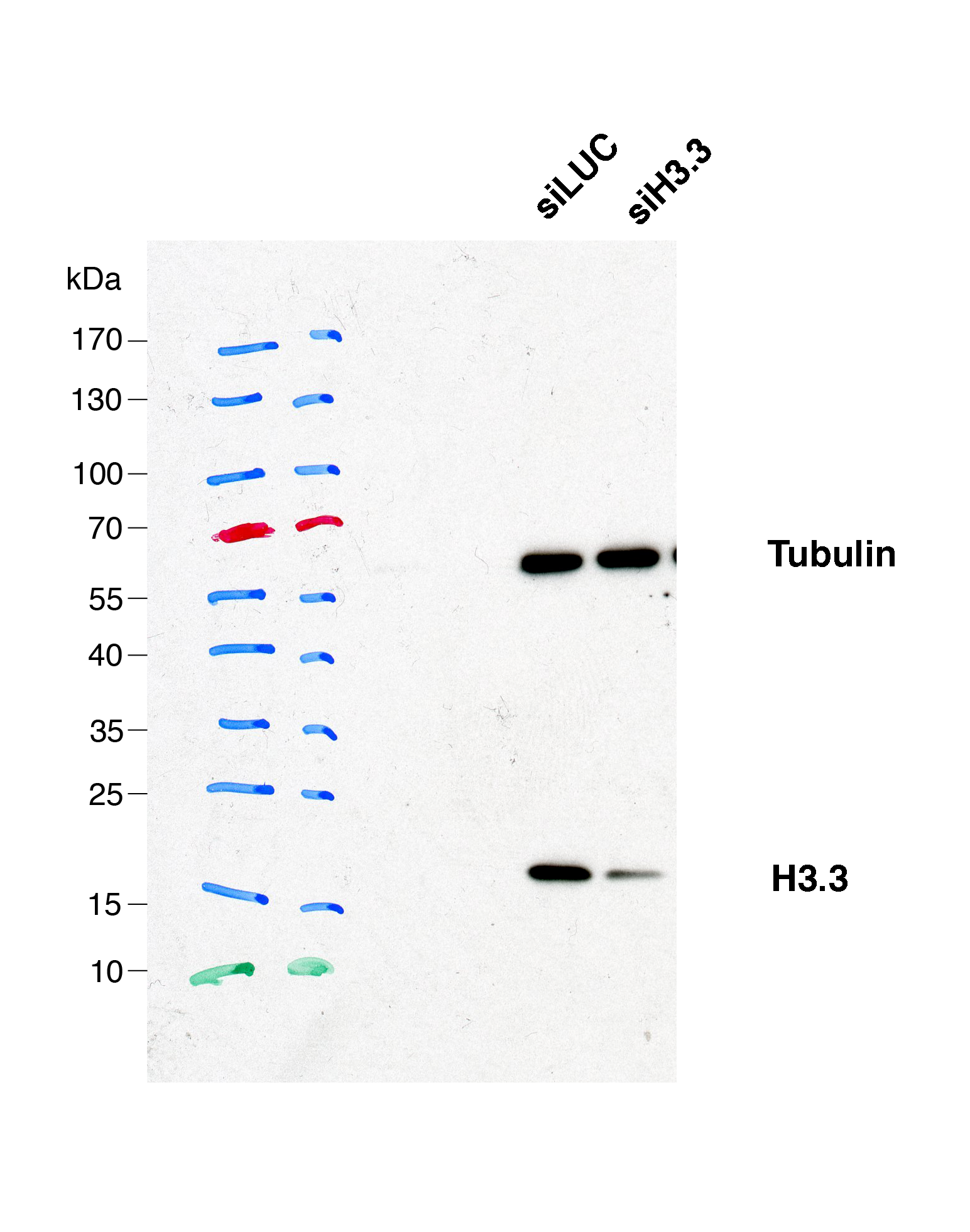

Supplement: Supplementary file 8 — Source Data [file 41467_2021_24153_MOESM8_ESM.zip › RawData/Main Figures/Fig1/d/WB.tif]

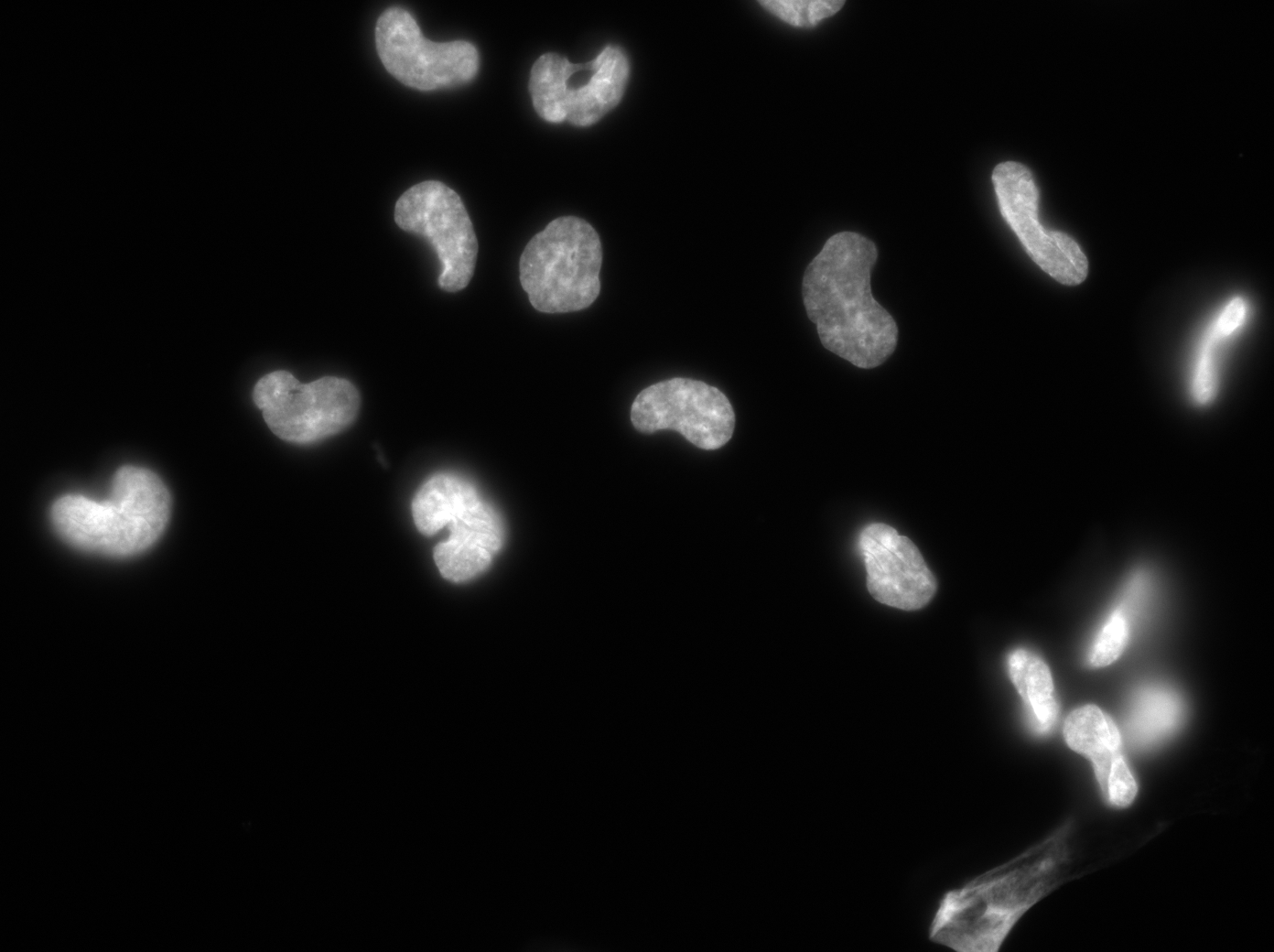

Supplement: Supplementary file 8 — Source Data [file 41467_2021_24153_MOESM8_ESM.zip › RawData/Main Figures/Fig2/b/XP4PA_DMSO_13_w1DAPI.TIF]

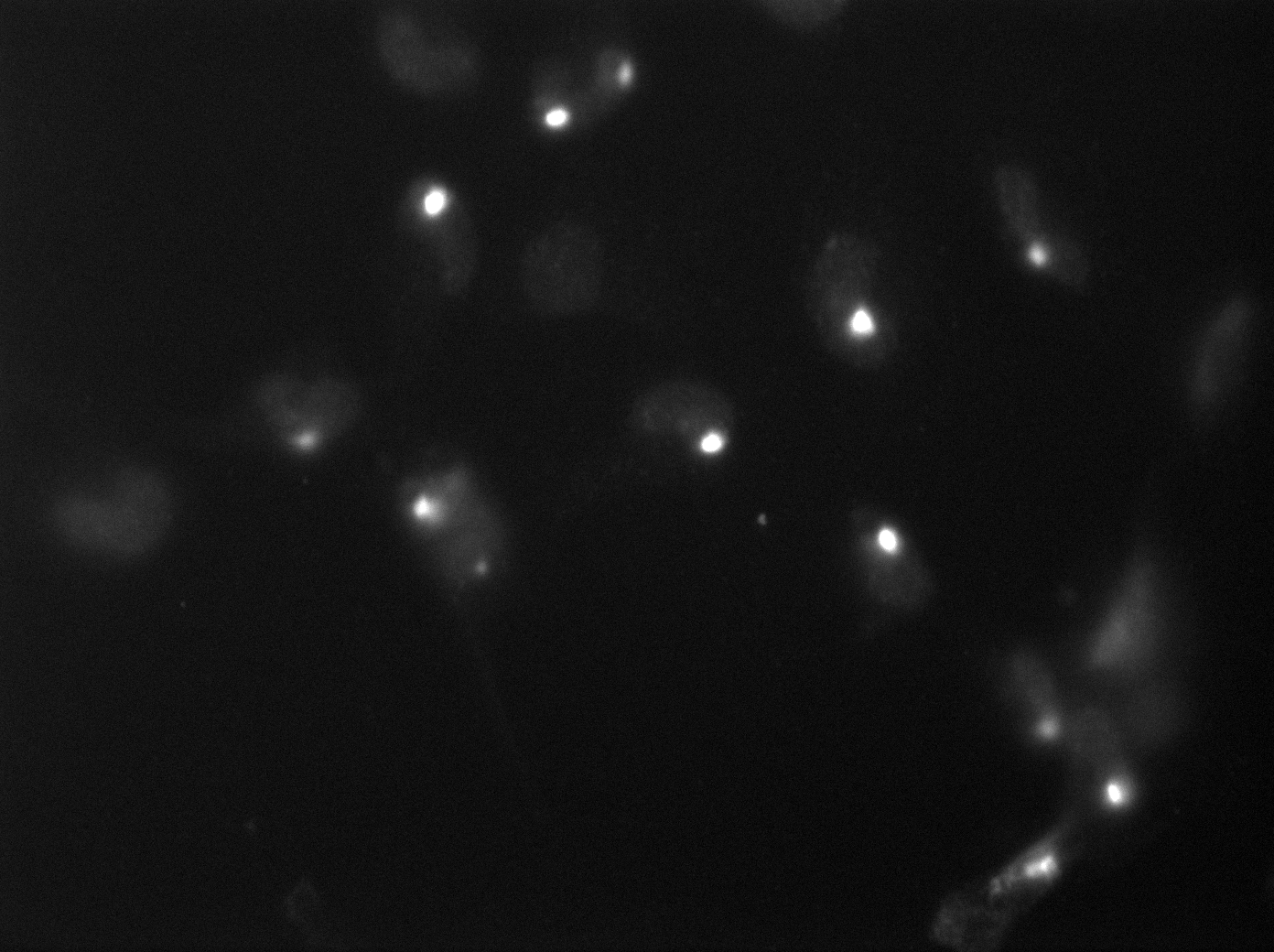

Supplement: Supplementary file 8 — Source Data [file 41467_2021_24153_MOESM8_ESM.zip › RawData/Main Figures/Fig2/b/XP4PA_DMSO_13_w2GFP.TIF]

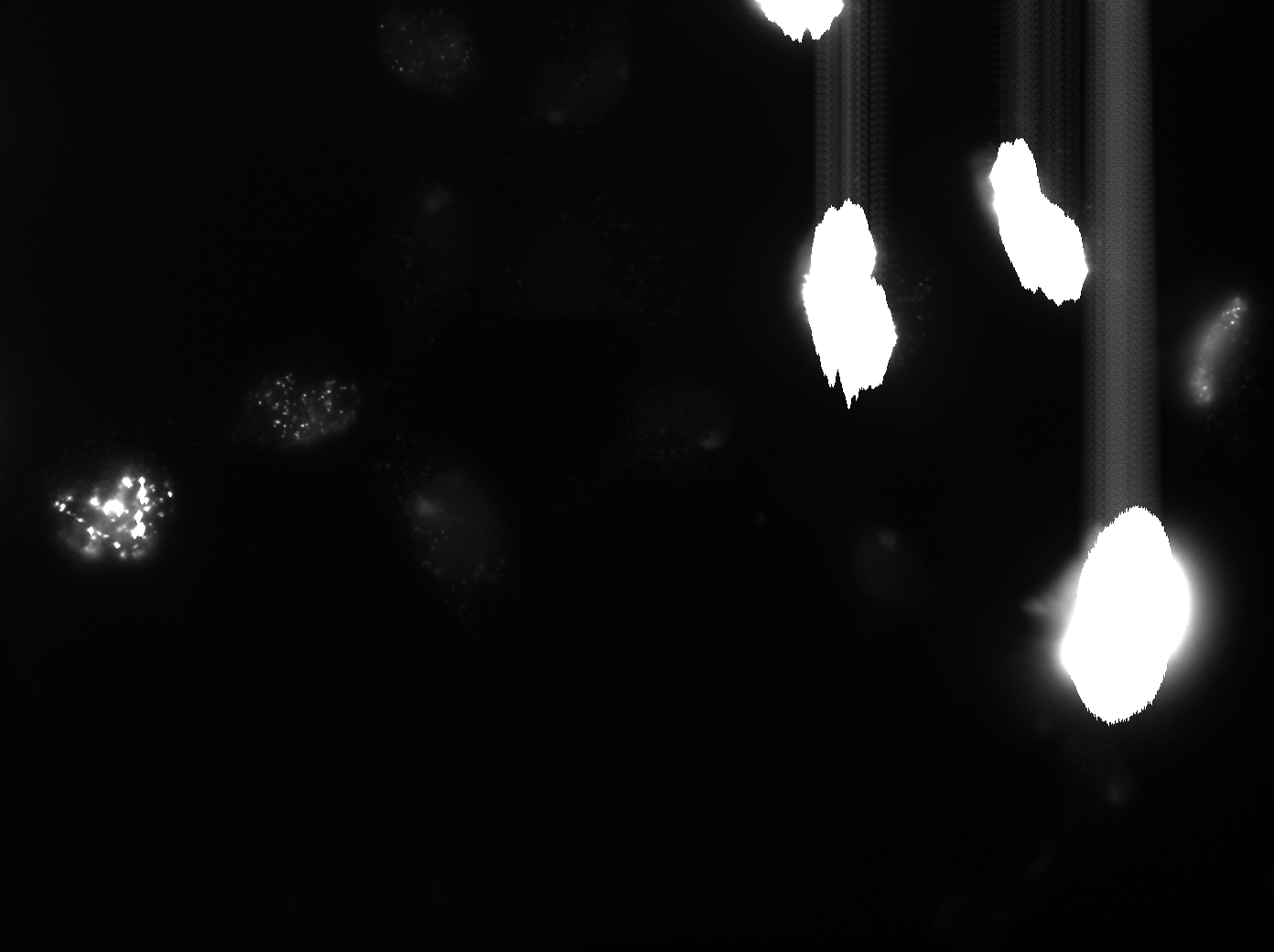

Supplement: Supplementary file 8 — Source Data [file 41467_2021_24153_MOESM8_ESM.zip › RawData/Main Figures/Fig2/b/XP4PA_DMSO_13_w3TX.TIF]

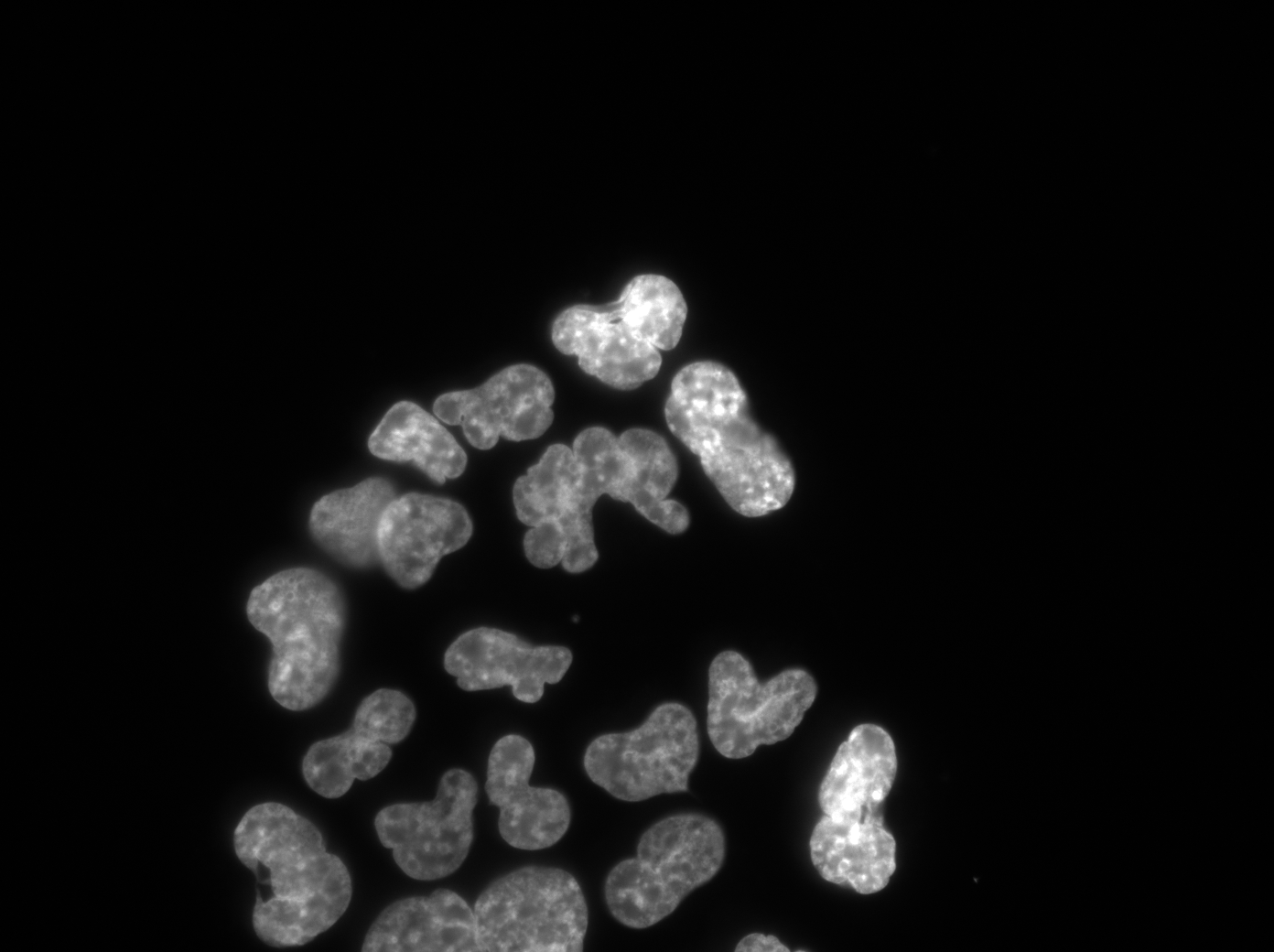

Supplement: Supplementary file 8 — Source Data [file 41467_2021_24153_MOESM8_ESM.zip › RawData/Main Figures/Fig2/b/XP4PA_FLV_05_w1DAPI.TIF]

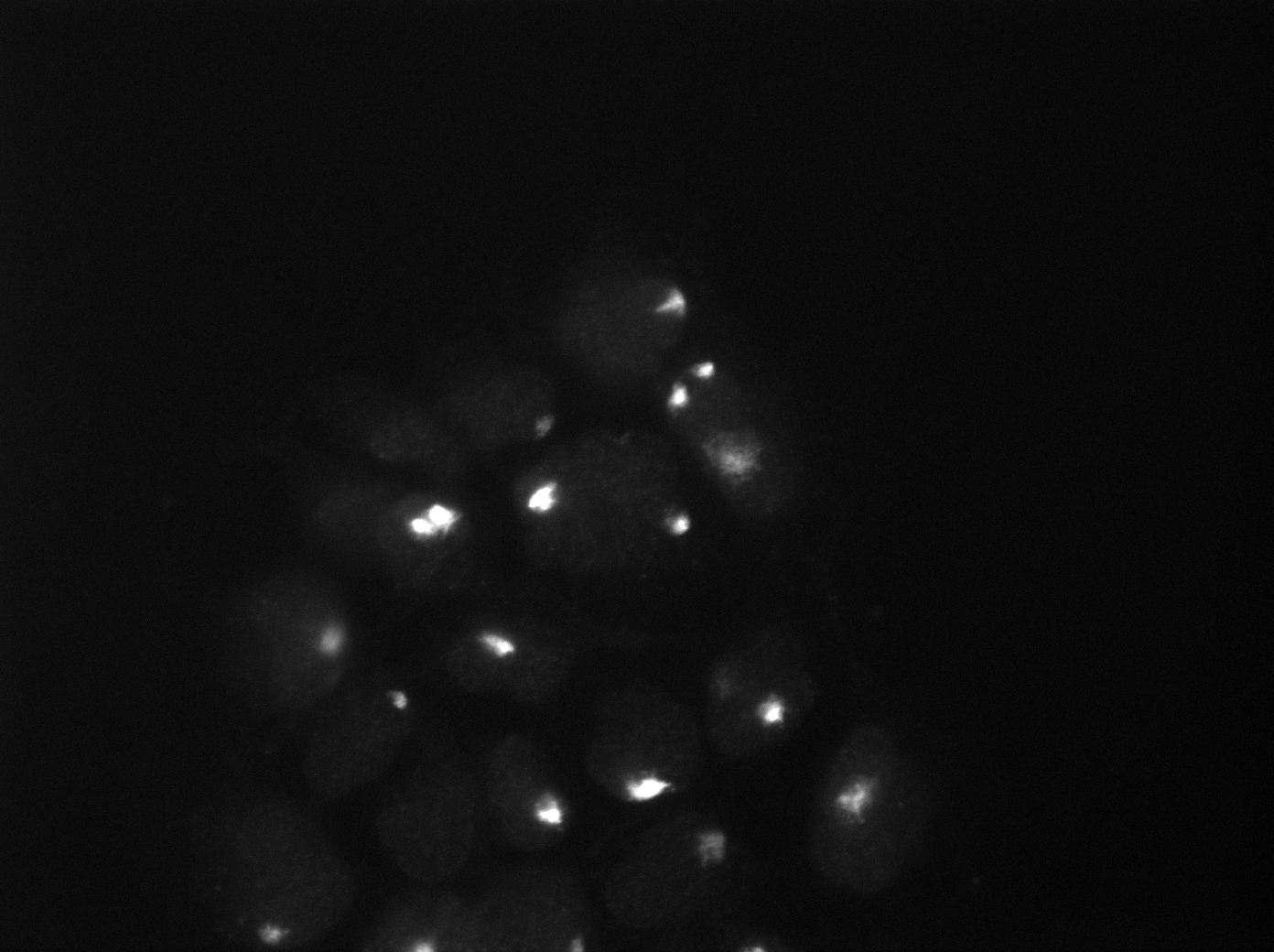

Supplement: Supplementary file 8 — Source Data [file 41467_2021_24153_MOESM8_ESM.zip › RawData/Main Figures/Fig2/b/XP4PA_FLV_05_w2GFP.TIF]

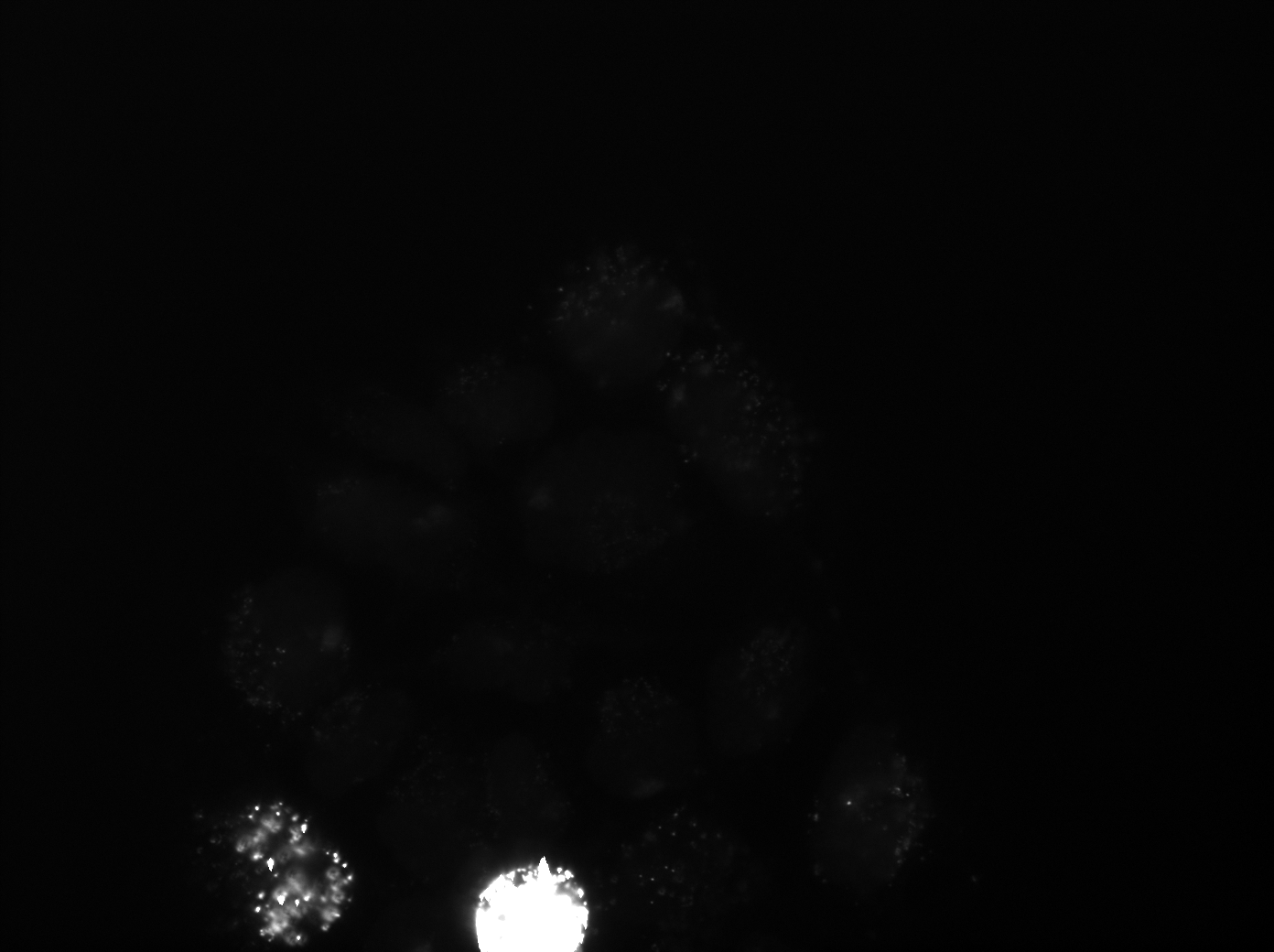

Supplement: Supplementary file 8 — Source Data [file 41467_2021_24153_MOESM8_ESM.zip › RawData/Main Figures/Fig2/b/XP4PA_FLV_05_w3TX.TIF]

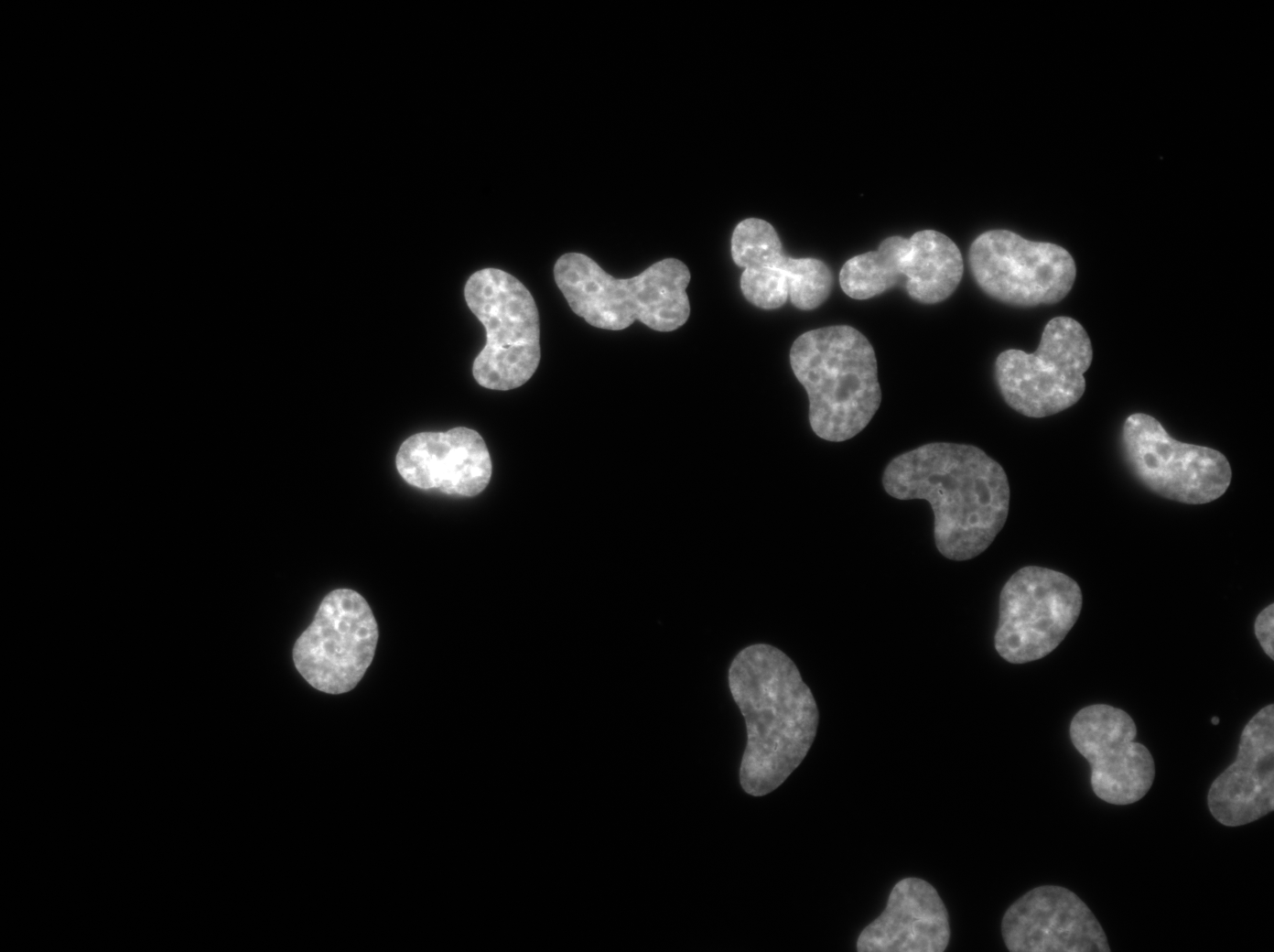

Supplement: Supplementary file 8 — Source Data [file 41467_2021_24153_MOESM8_ESM.zip › RawData/Main Figures/Fig2/c/TCR-XP4_siERCC6_06_w1DAPI.TIF]

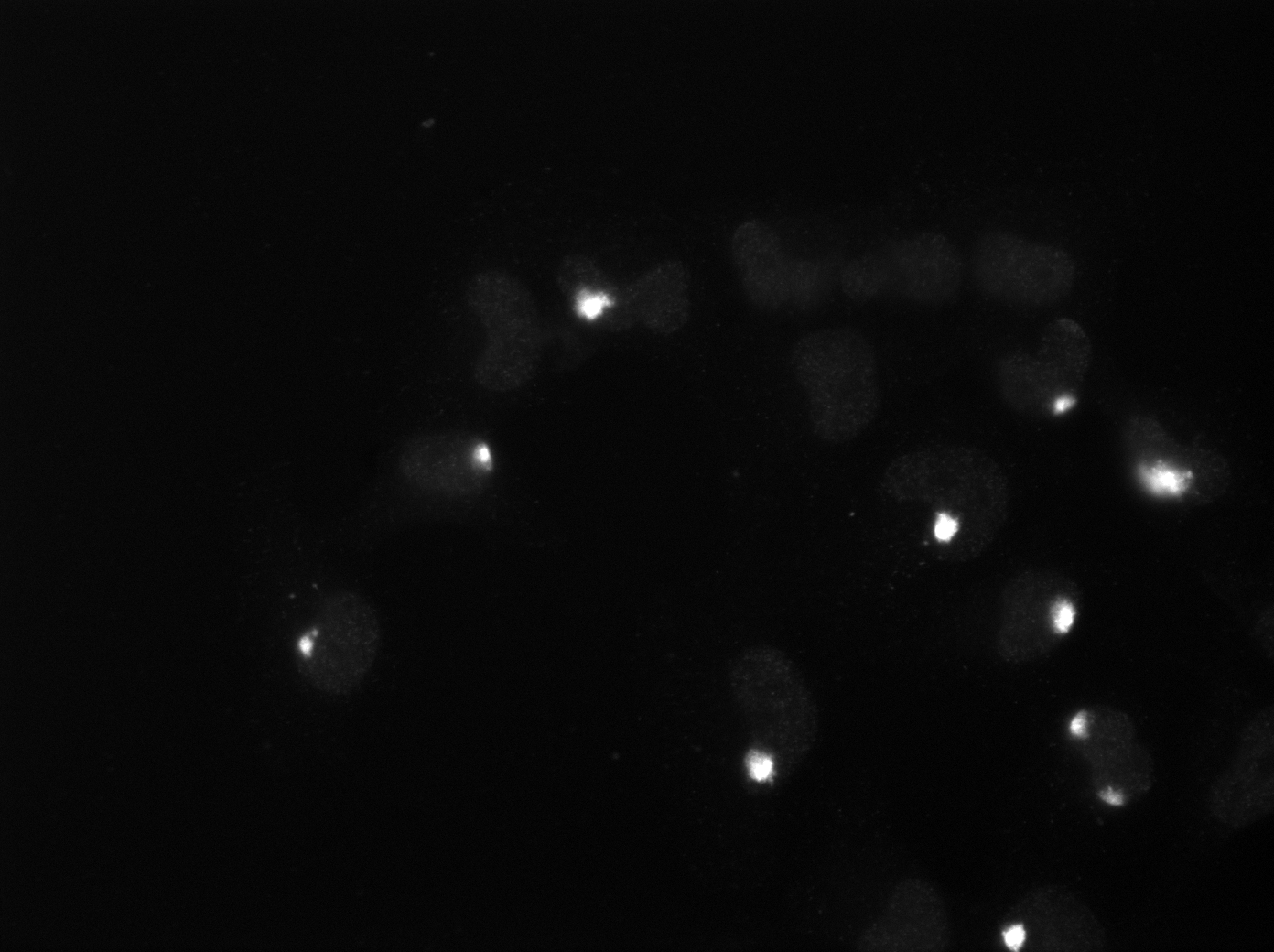

Supplement: Supplementary file 8 — Source Data [file 41467_2021_24153_MOESM8_ESM.zip › RawData/Main Figures/Fig2/c/TCR-XP4_siERCC6_06_w2GFP.TIF]

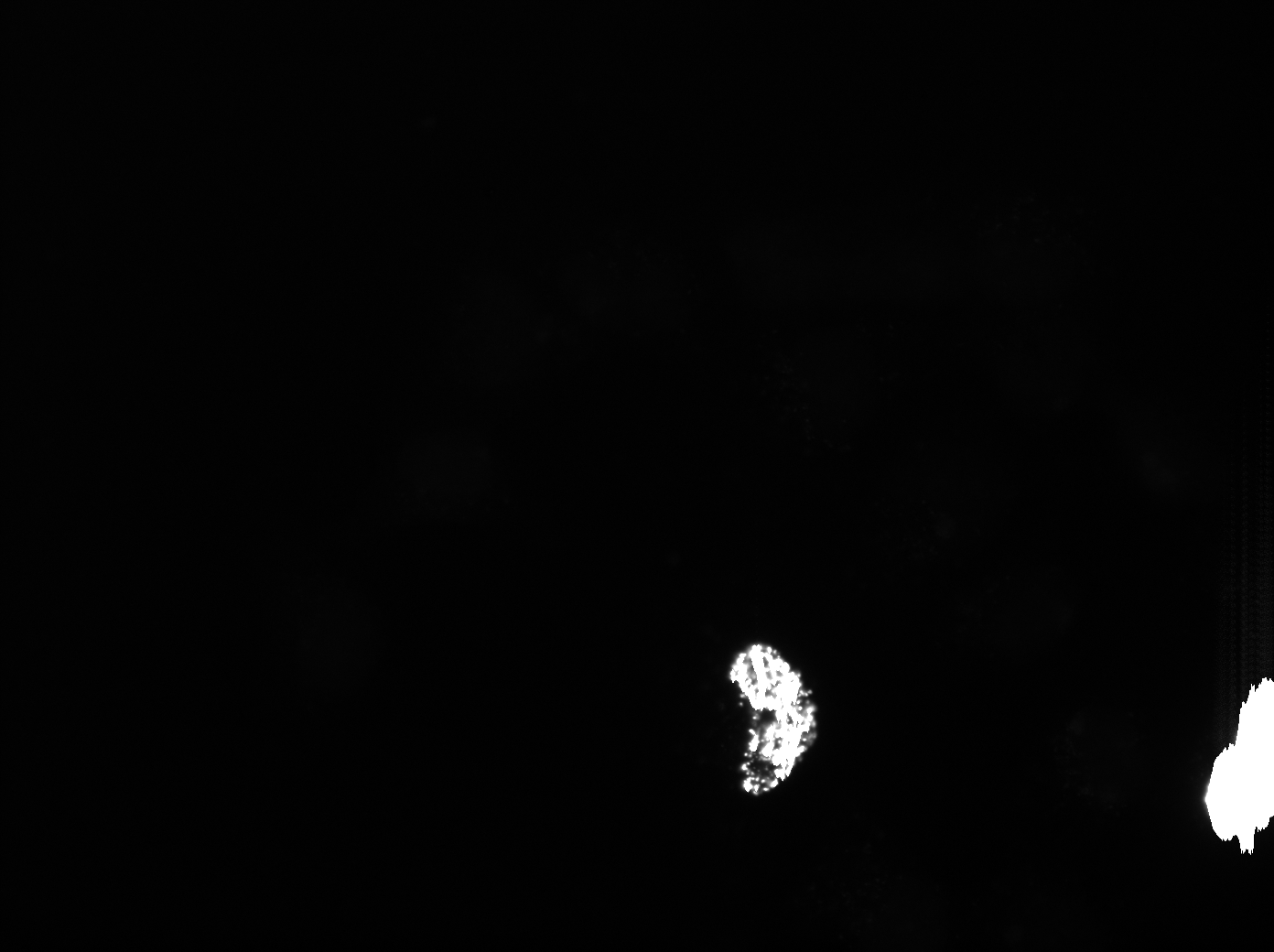

Supplement: Supplementary file 8 — Source Data [file 41467_2021_24153_MOESM8_ESM.zip › RawData/Main Figures/Fig2/c/TCR-XP4_siERCC6_06_w3TX.TIF]

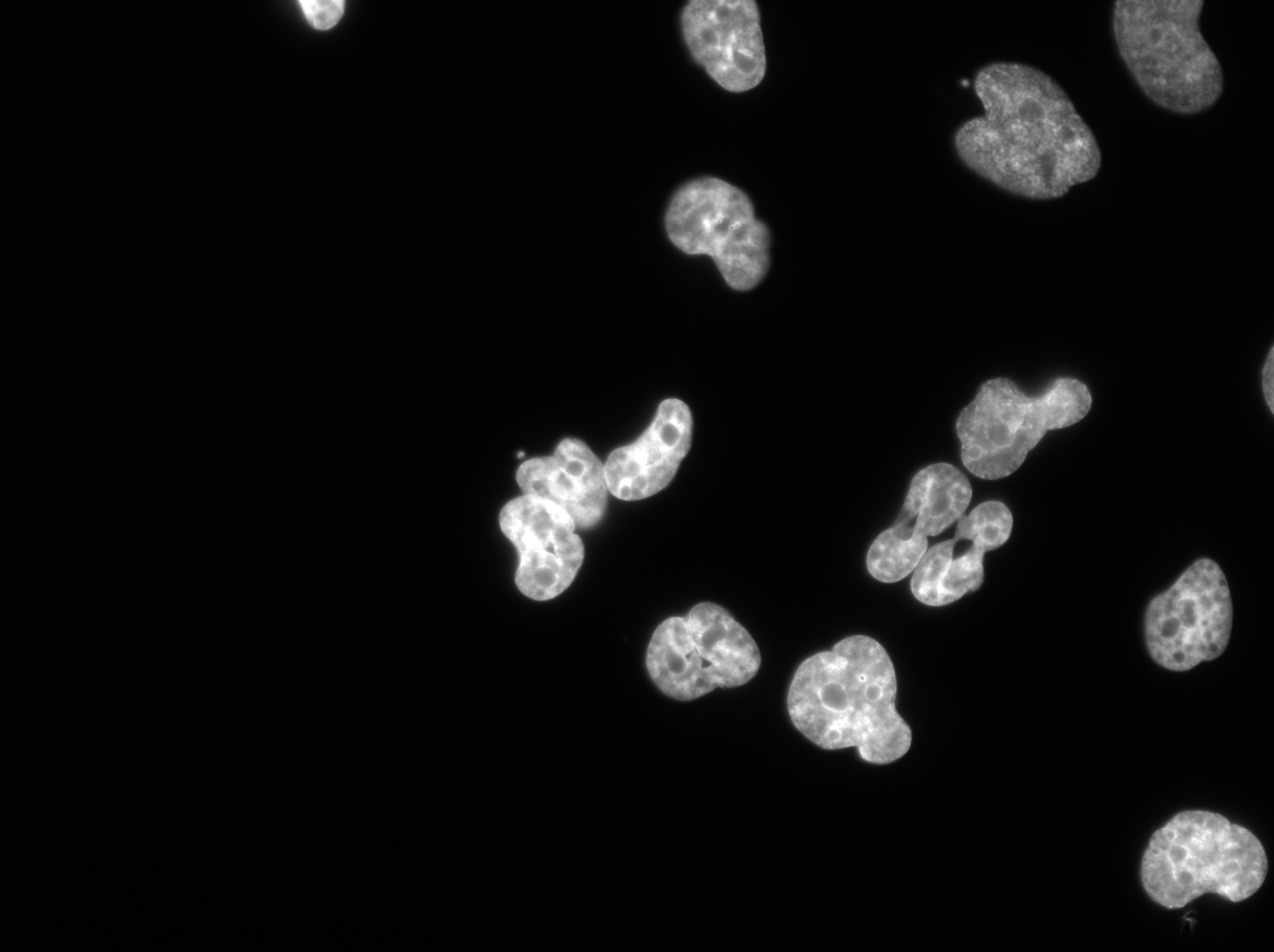

Supplement: Supplementary file 8 — Source Data [file 41467_2021_24153_MOESM8_ESM.zip › RawData/Main Figures/Fig2/c/TCR-XP4_siHIRA_07_w1DAPI.TIF]

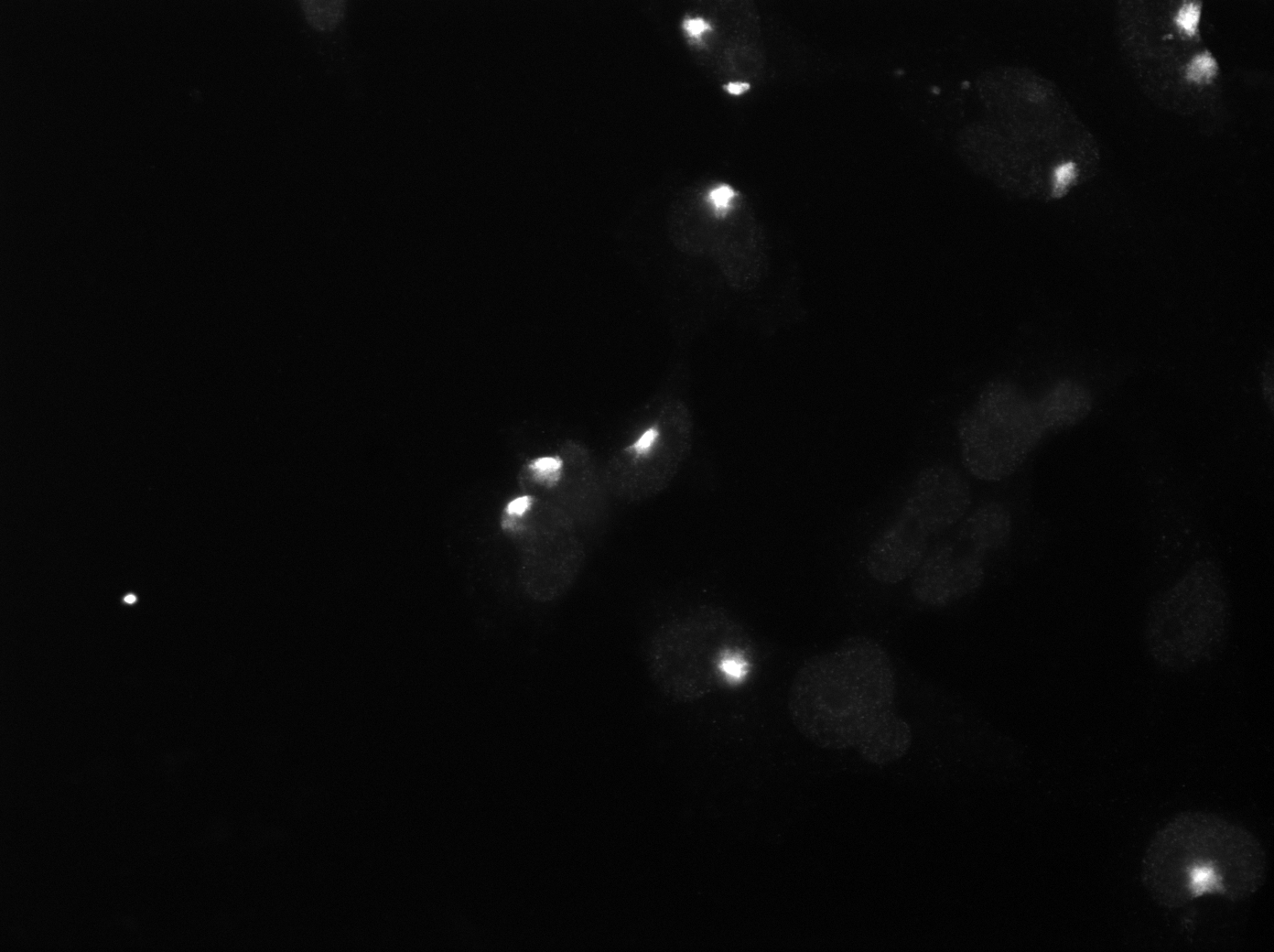

Supplement: Supplementary file 8 — Source Data [file 41467_2021_24153_MOESM8_ESM.zip › RawData/Main Figures/Fig2/c/TCR-XP4_siHIRA_07_w2GFP.TIF]

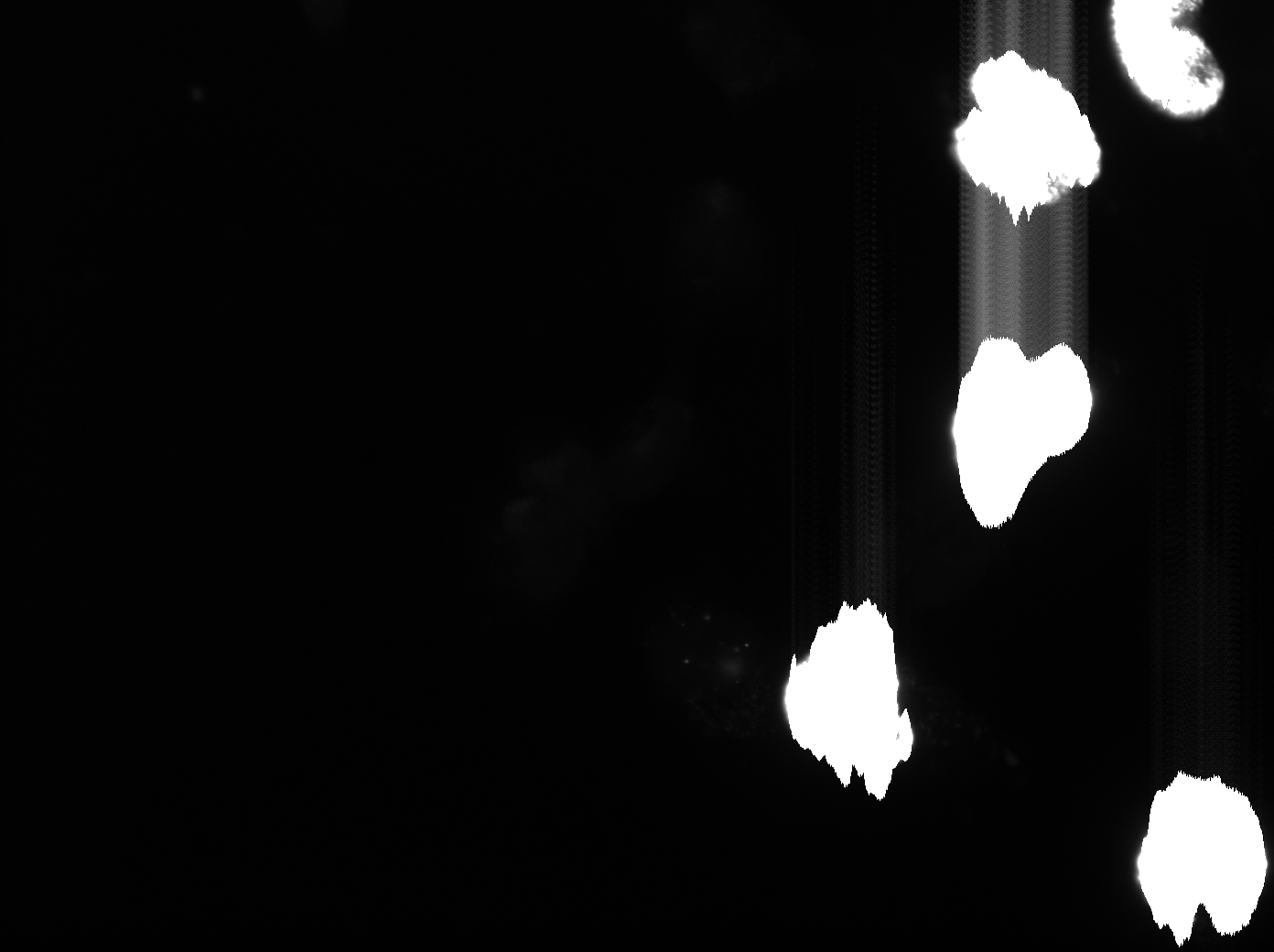

Supplement: Supplementary file 8 — Source Data [file 41467_2021_24153_MOESM8_ESM.zip › RawData/Main Figures/Fig2/c/TCR-XP4_siHIRA_07_w3TX.TIF]

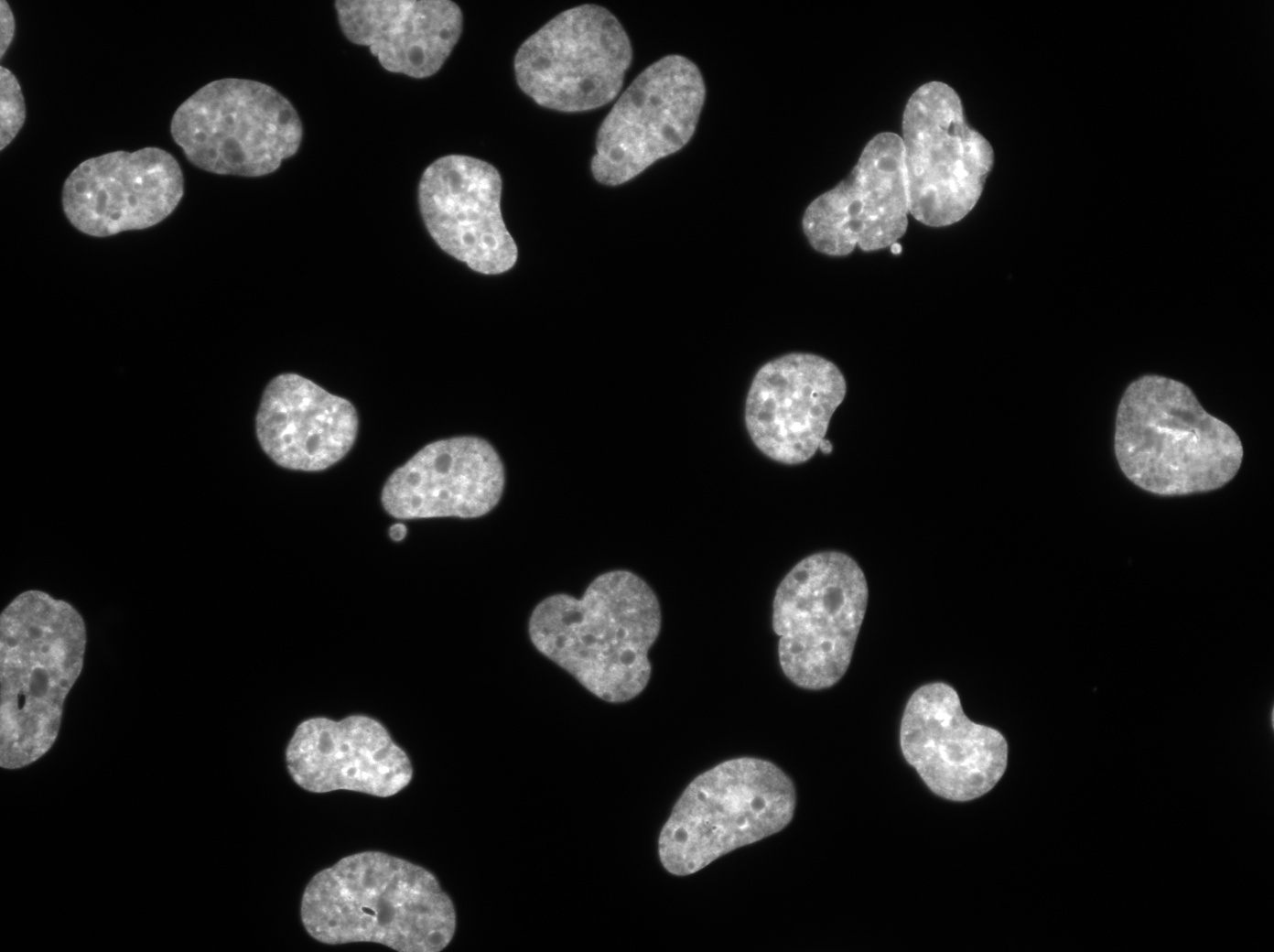

Supplement: Supplementary file 8 — Source Data [file 41467_2021_24153_MOESM8_ESM.zip › RawData/Main Figures/Fig2/c/TCR-XP4_siLUC_01_w1DAPI.TIF]

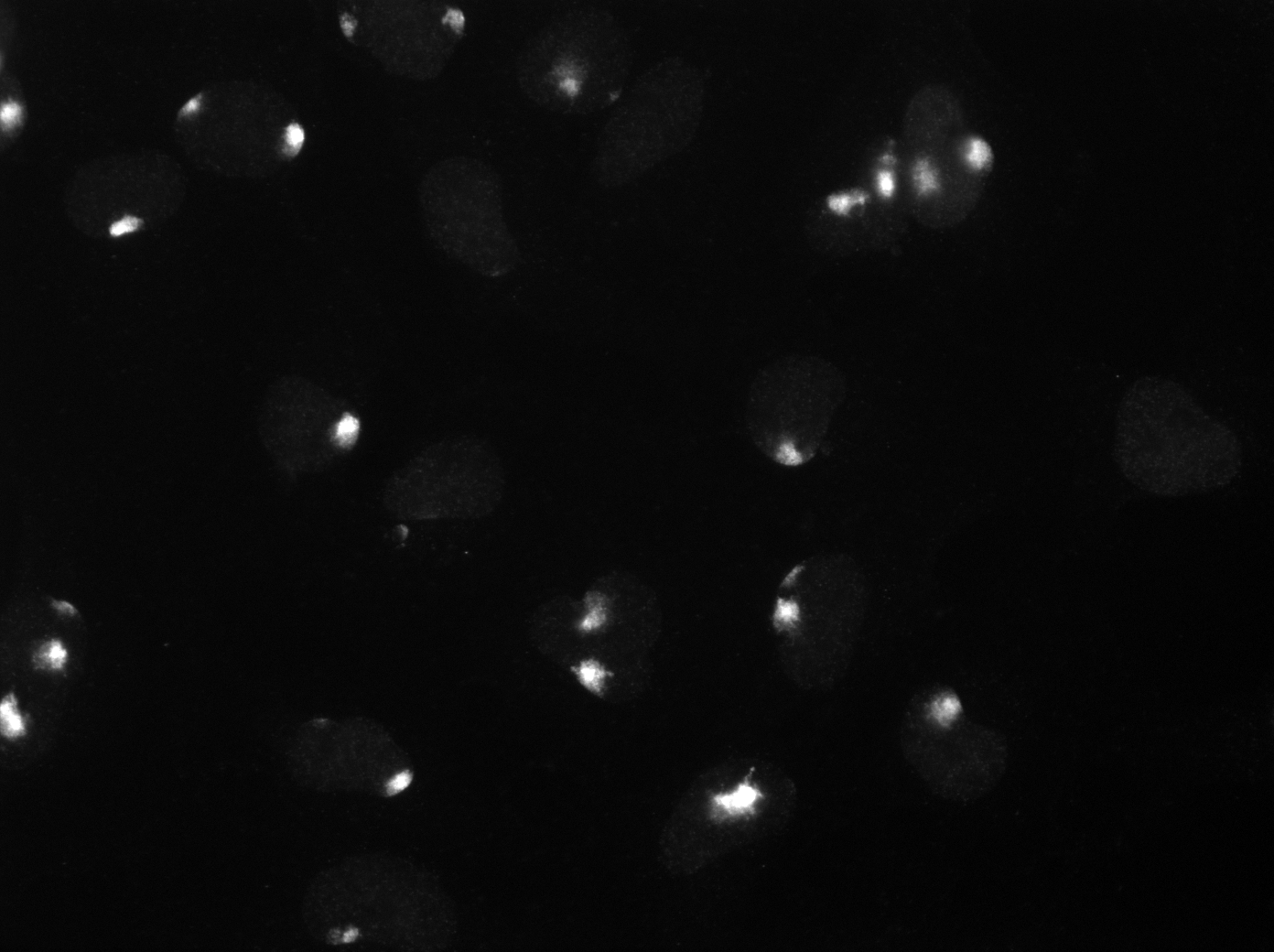

Supplement: Supplementary file 8 — Source Data [file 41467_2021_24153_MOESM8_ESM.zip › RawData/Main Figures/Fig2/c/TCR-XP4_siLUC_01_w2GFP.TIF]

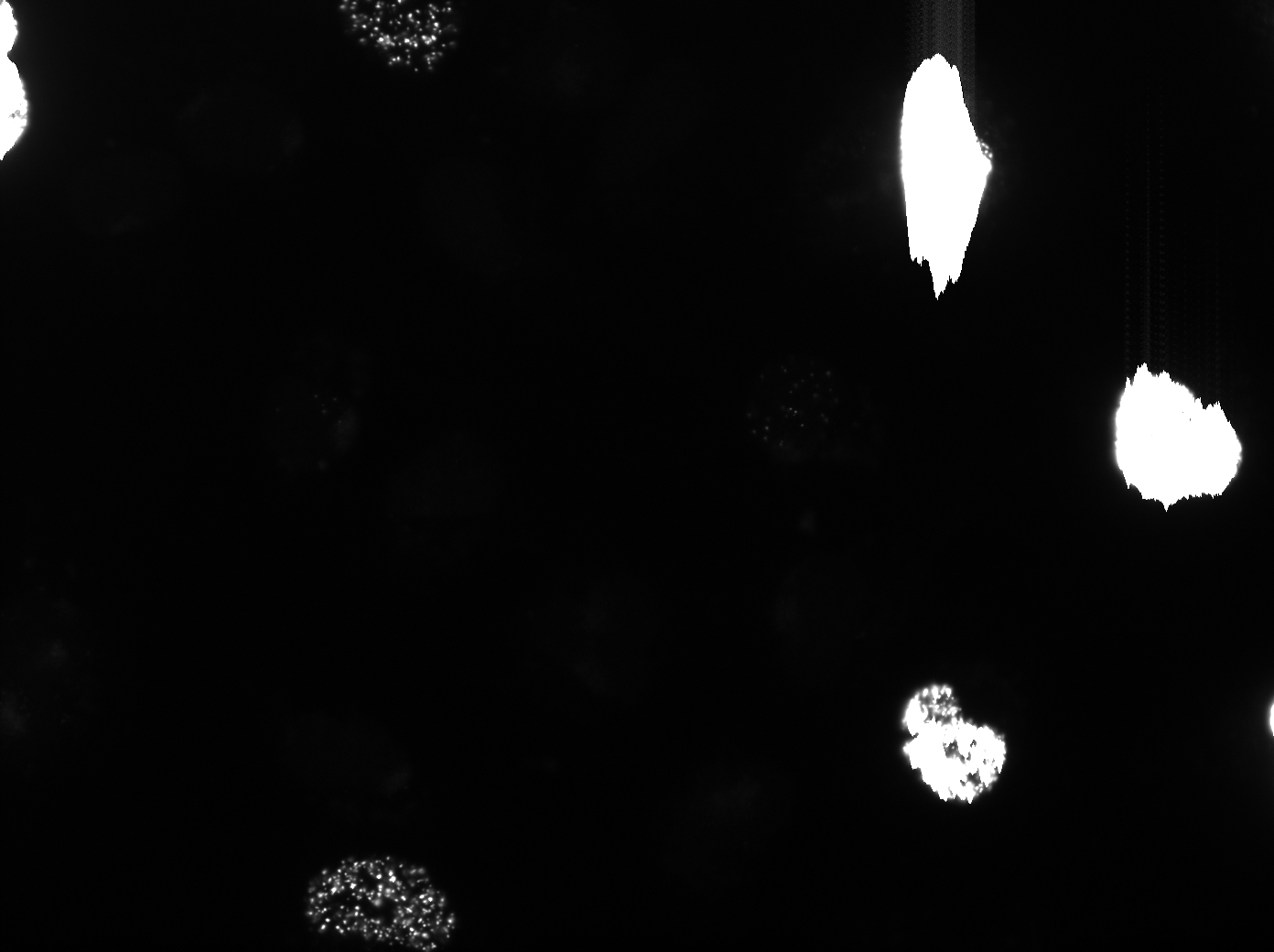

Supplement: Supplementary file 8 — Source Data [file 41467_2021_24153_MOESM8_ESM.zip › RawData/Main Figures/Fig2/c/TCR-XP4_siLUC_01_w3TX.TIF]

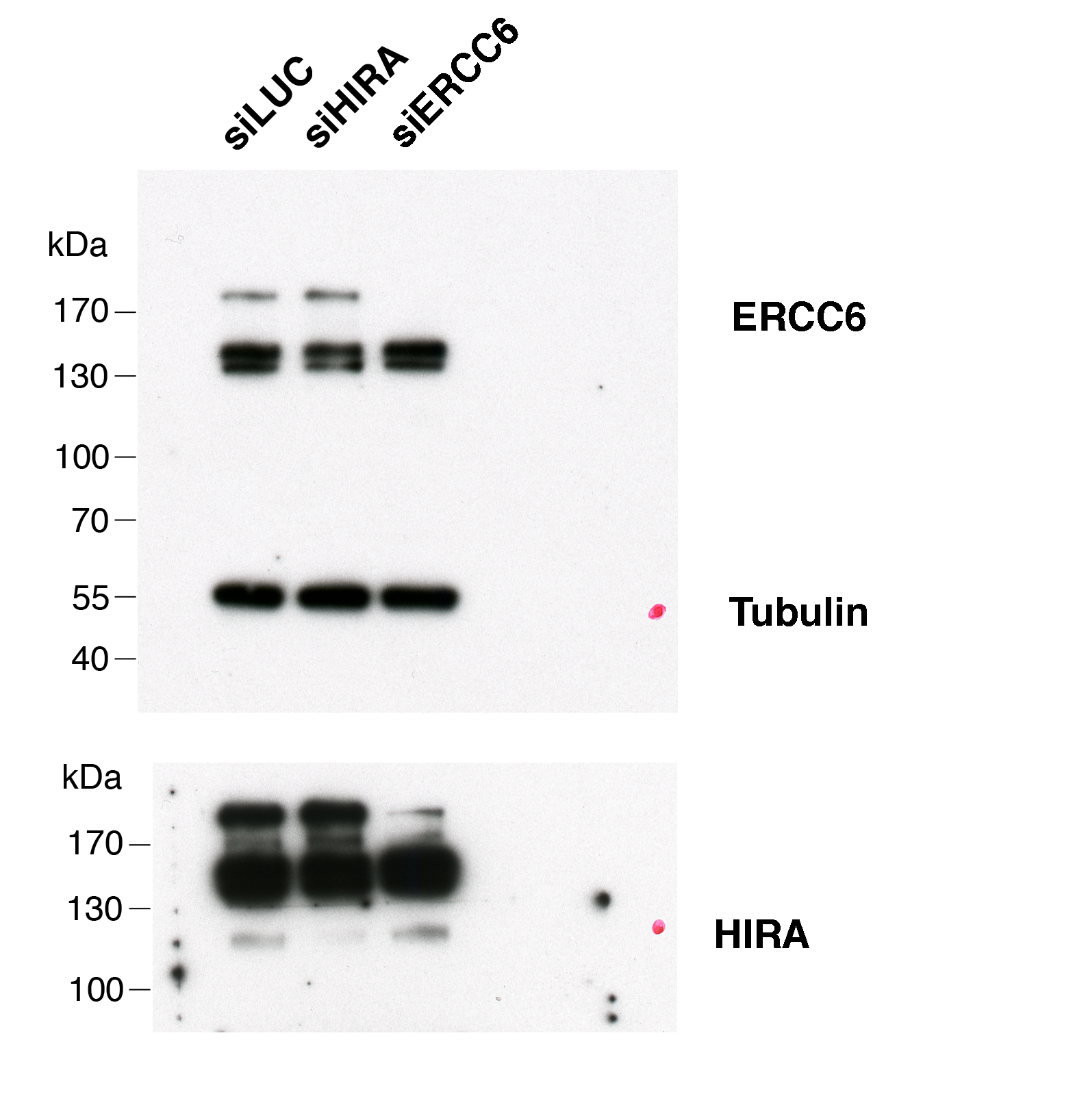

Supplement: Supplementary file 8 — Source Data [file 41467_2021_24153_MOESM8_ESM.zip › RawData/Main Figures/Fig2/c/WB.tif]

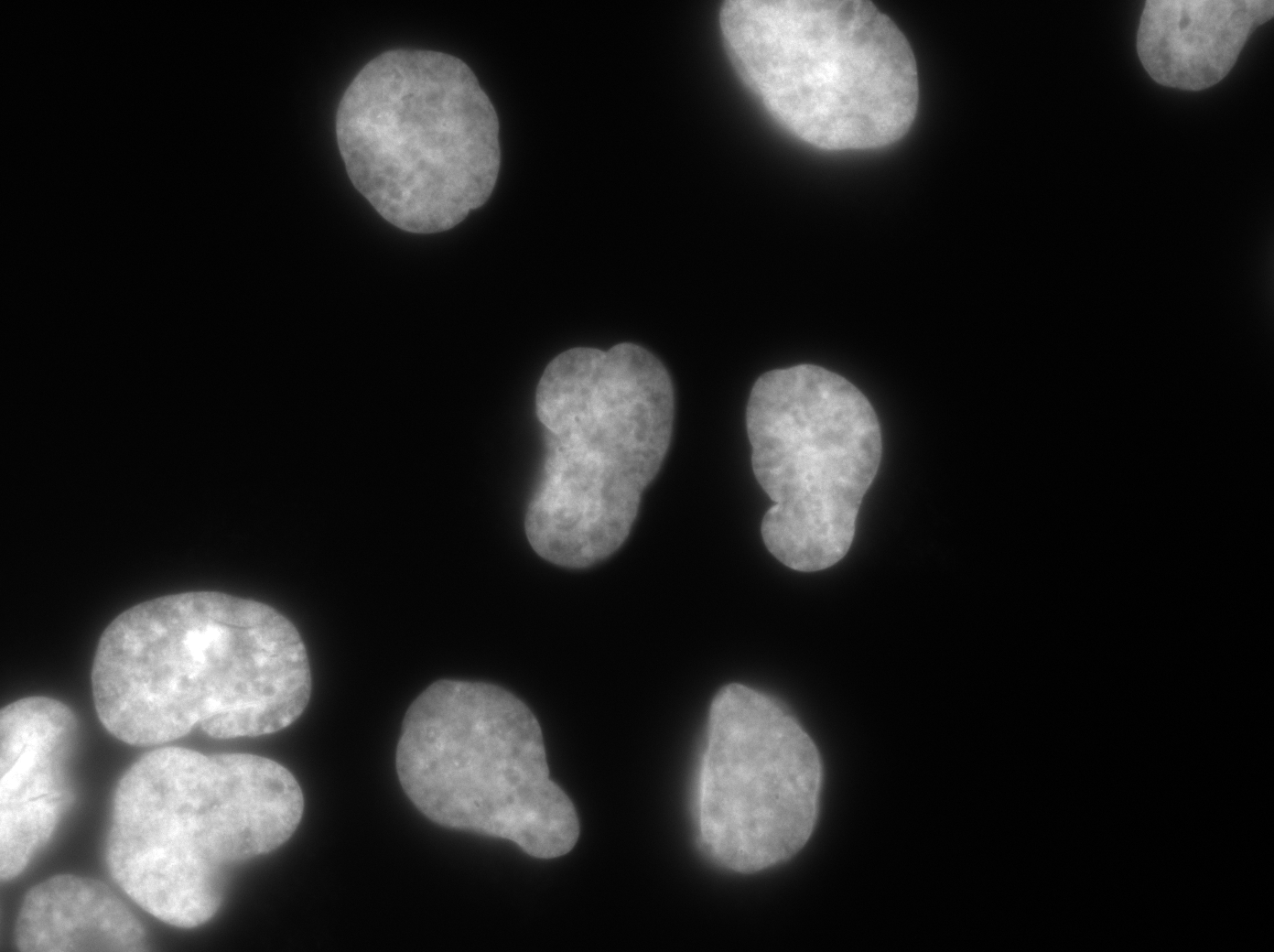

Supplement: Supplementary file 8 — Source Data [file 41467_2021_24153_MOESM8_ESM.zip › RawData/Main Figures/Fig3/a/hira_xpb_siLUC_2_w1DAPI.TIF]

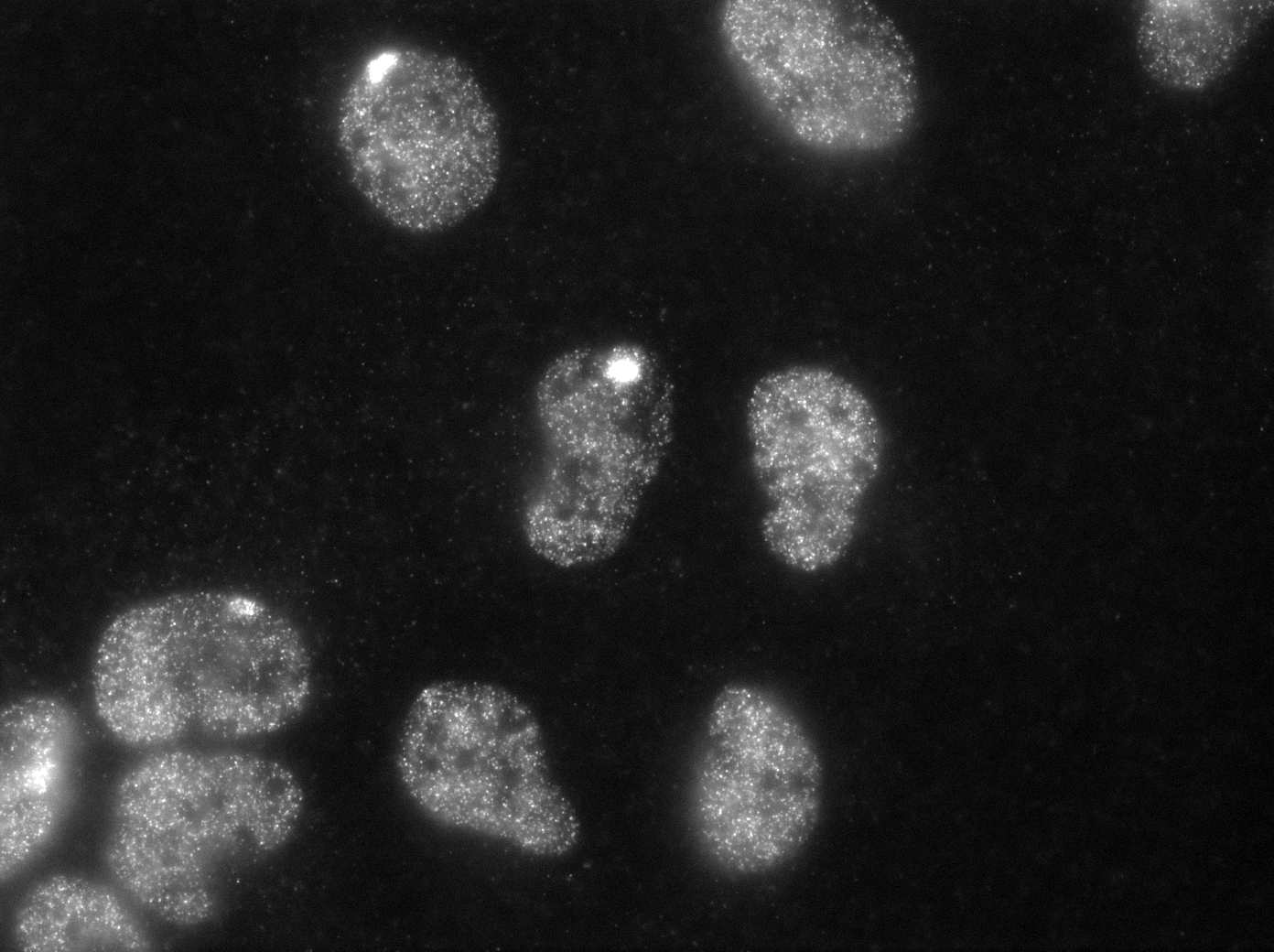

Supplement: Supplementary file 8 — Source Data [file 41467_2021_24153_MOESM8_ESM.zip › RawData/Main Figures/Fig3/a/hira_xpb_siLUC_2_w2GFP.TIF]

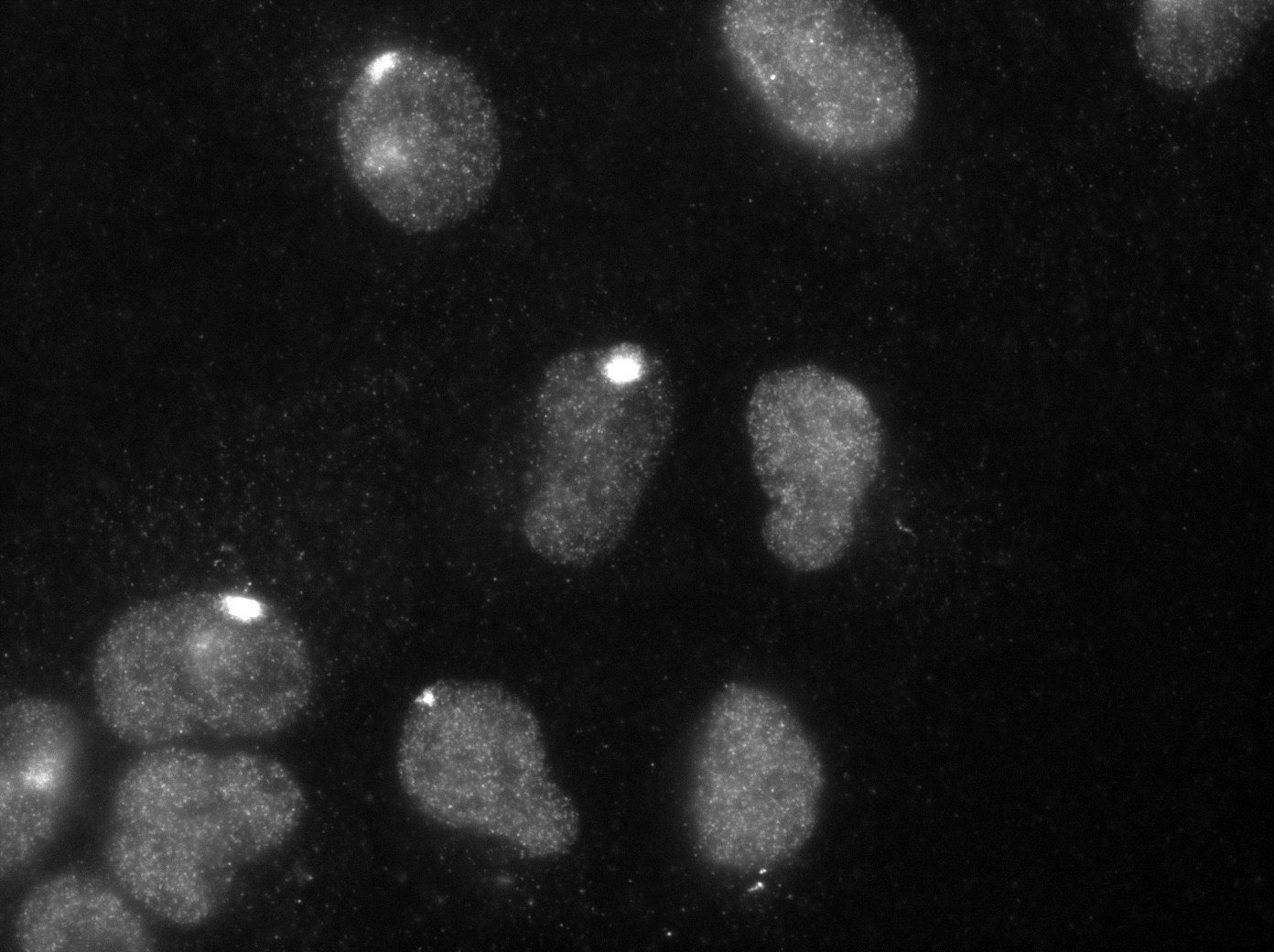

Supplement: Supplementary file 8 — Source Data [file 41467_2021_24153_MOESM8_ESM.zip › RawData/Main Figures/Fig3/a/hira_xpb_siLUC_2_w3TX.TIF]

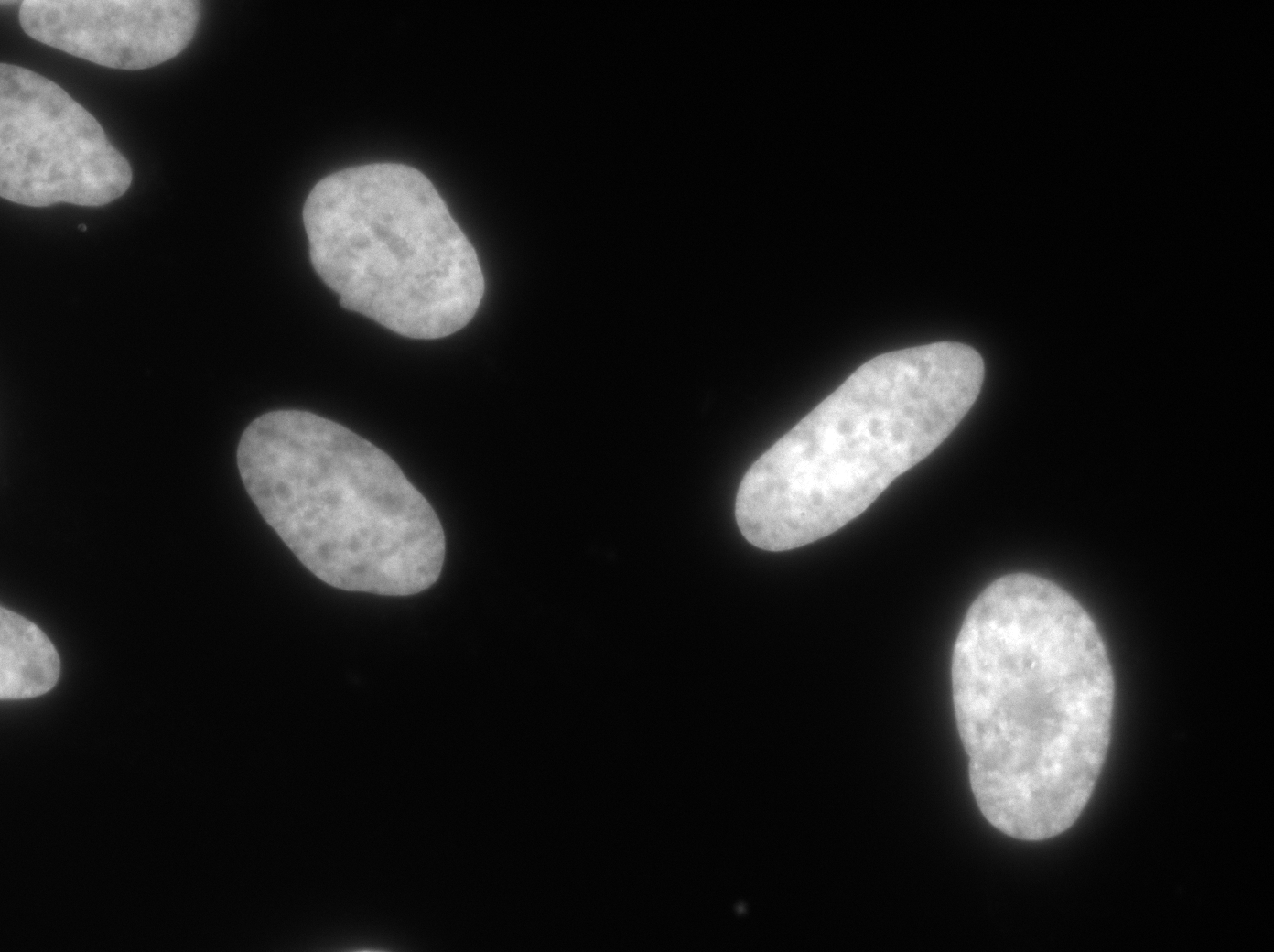

Supplement: Supplementary file 8 — Source Data [file 41467_2021_24153_MOESM8_ESM.zip › RawData/Main Figures/Fig3/a/hira_xpb_siVCP1_3_w1DAPI.TIF]

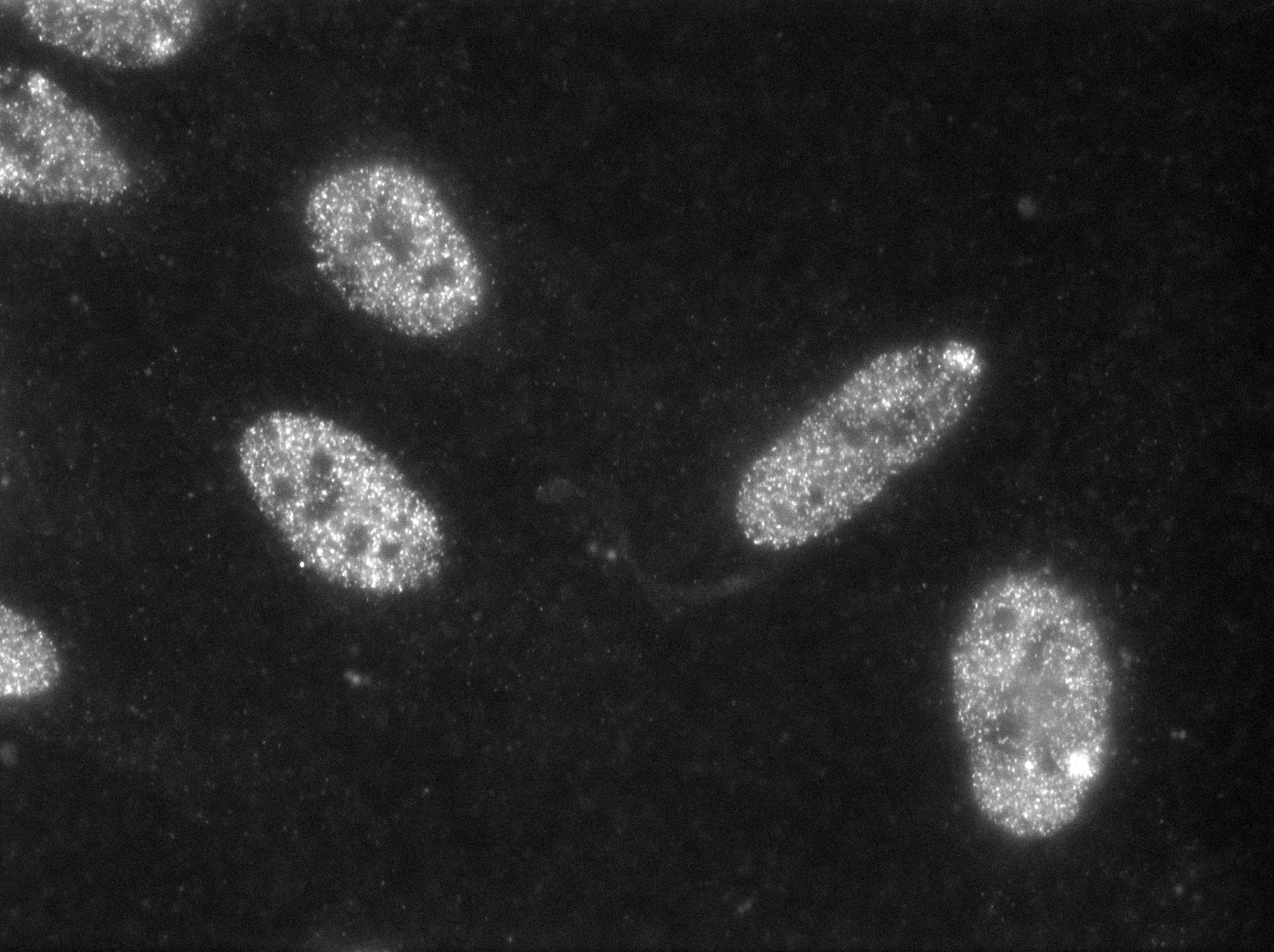

Supplement: Supplementary file 8 — Source Data [file 41467_2021_24153_MOESM8_ESM.zip › RawData/Main Figures/Fig3/a/hira_xpb_siVCP1_3_w2GFP.TIF]

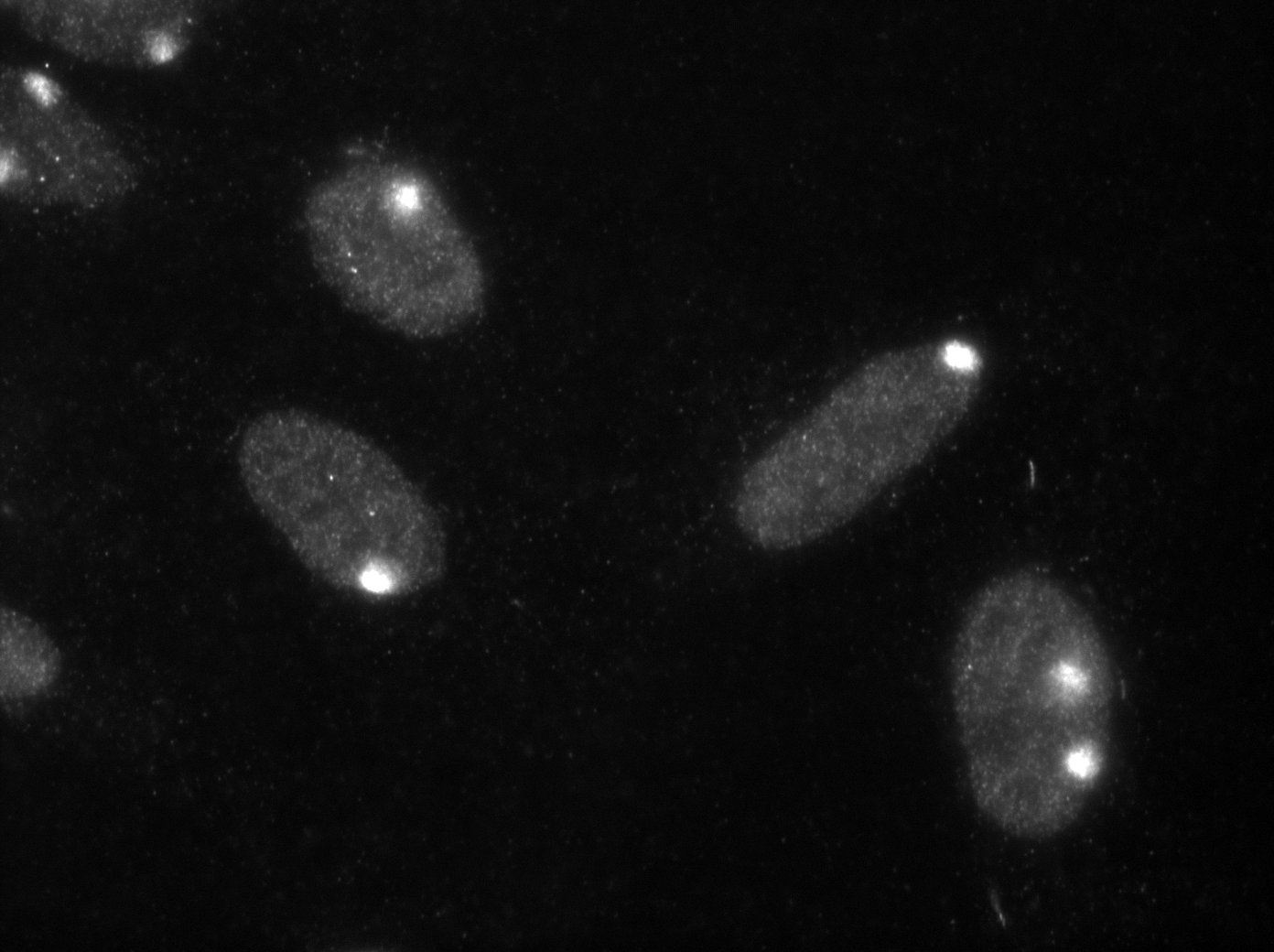

Supplement: Supplementary file 8 — Source Data [file 41467_2021_24153_MOESM8_ESM.zip › RawData/Main Figures/Fig3/a/hira_xpb_siVCP1_3_w3TX.TIF]

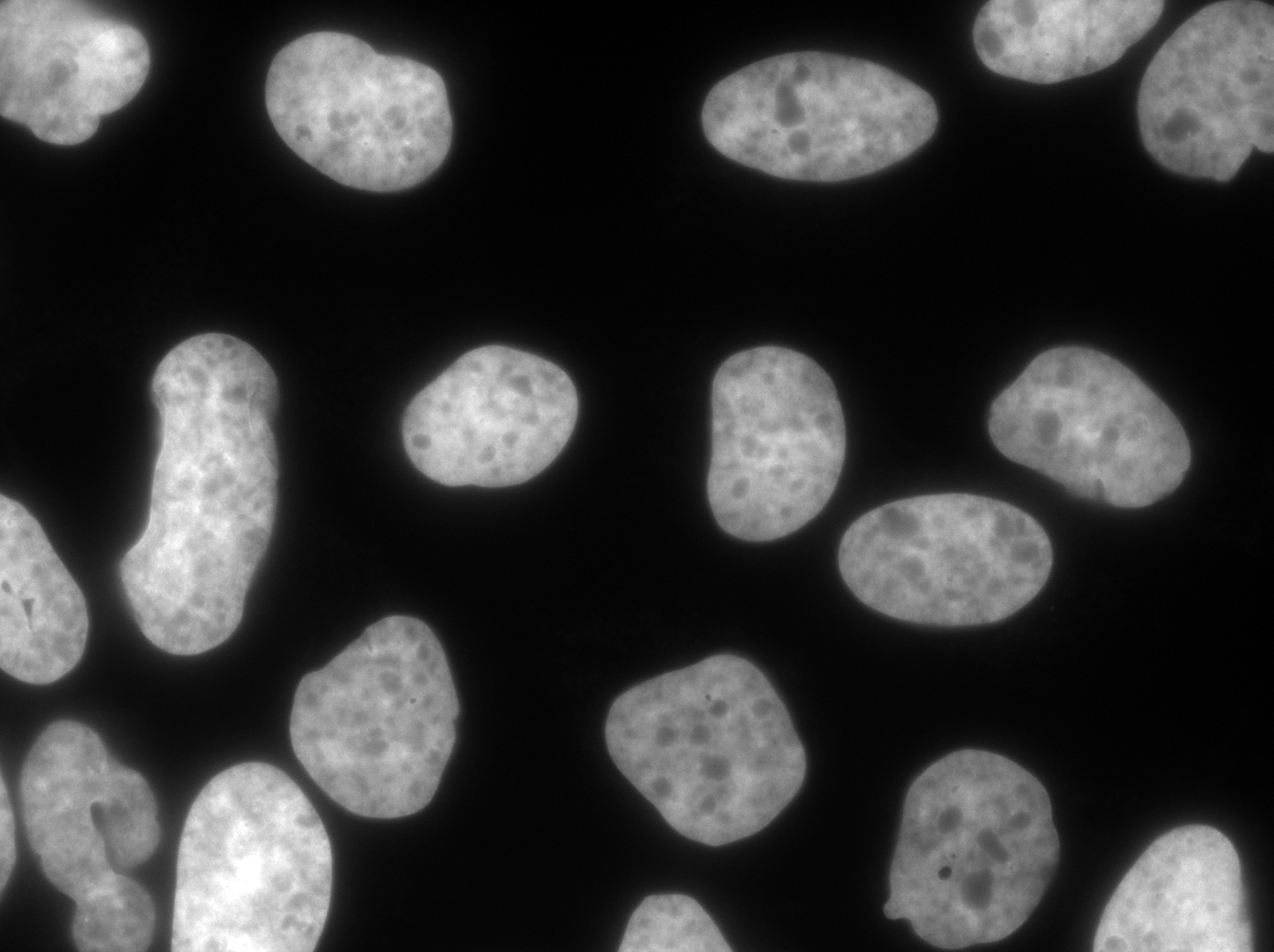

Supplement: Supplementary file 8 — Source Data [file 41467_2021_24153_MOESM8_ESM.zip › RawData/Main Figures/Fig3/a/hira_xpb_siVCP2_1_w1DAPI.TIF]

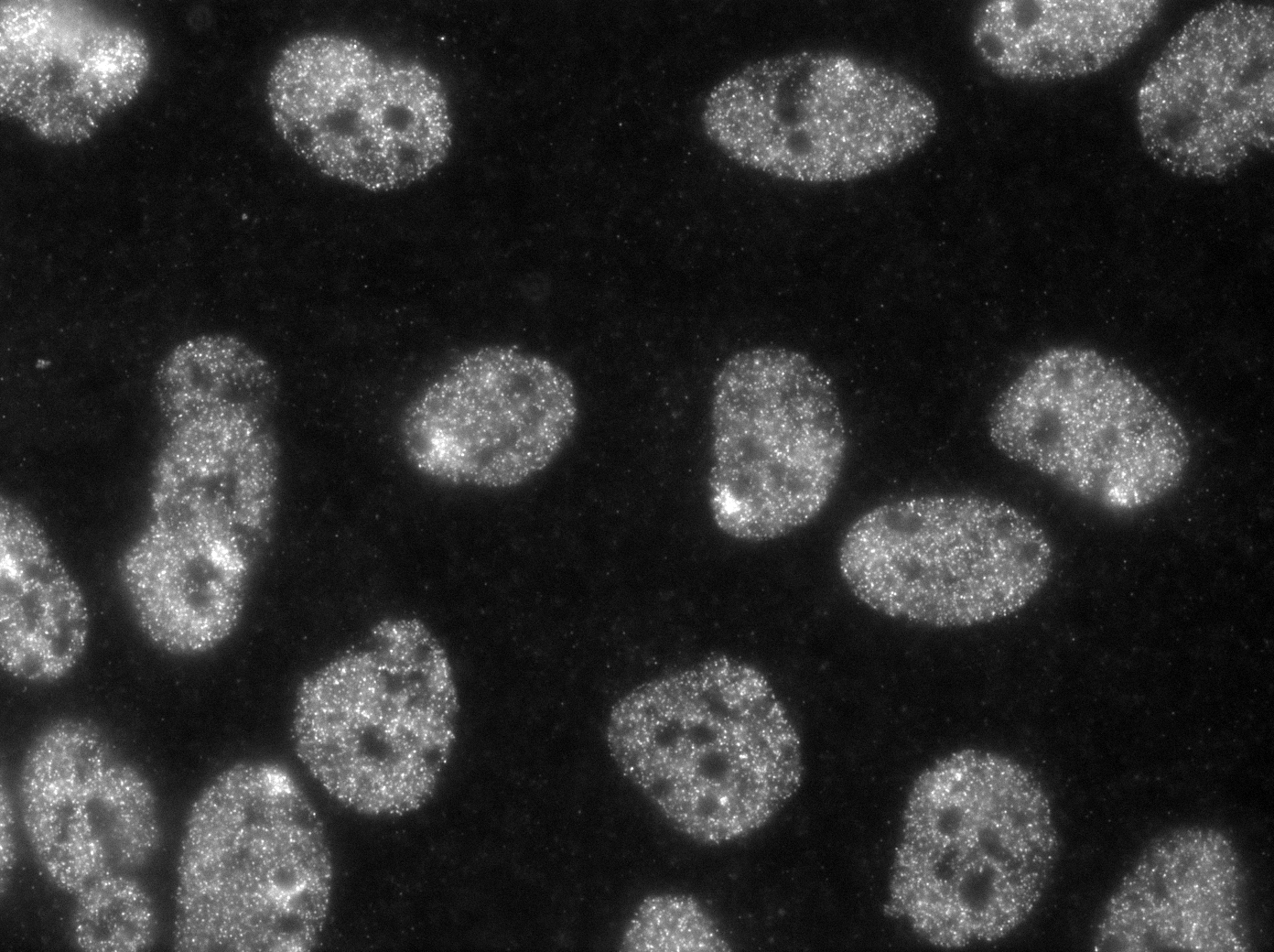

Supplement: Supplementary file 8 — Source Data [file 41467_2021_24153_MOESM8_ESM.zip › RawData/Main Figures/Fig3/a/hira_xpb_siVCP2_1_w2GFP.TIF]

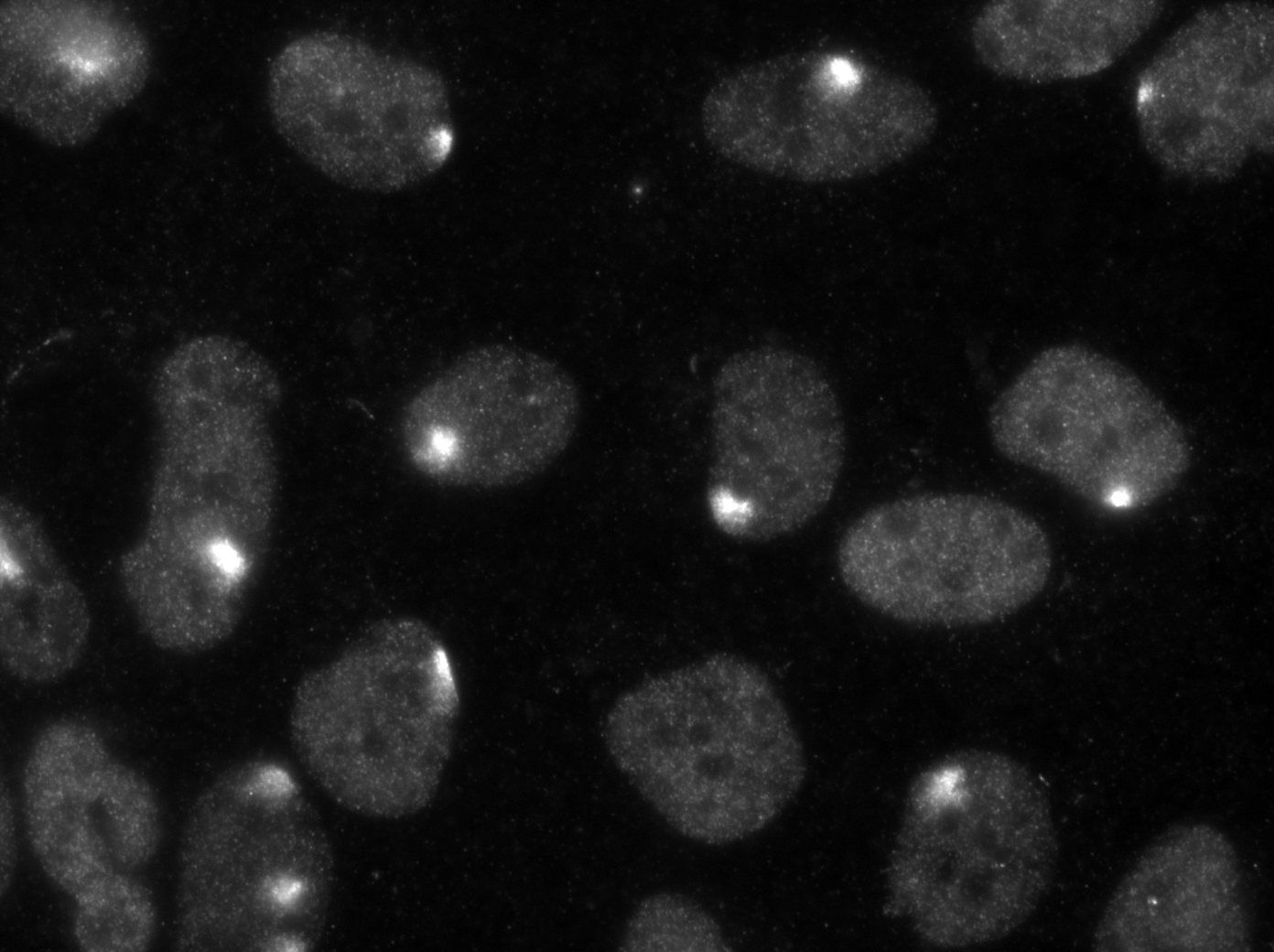

Supplement: Supplementary file 8 — Source Data [file 41467_2021_24153_MOESM8_ESM.zip › RawData/Main Figures/Fig3/a/hira_xpb_siVCP2_1_w3TX.TIF]

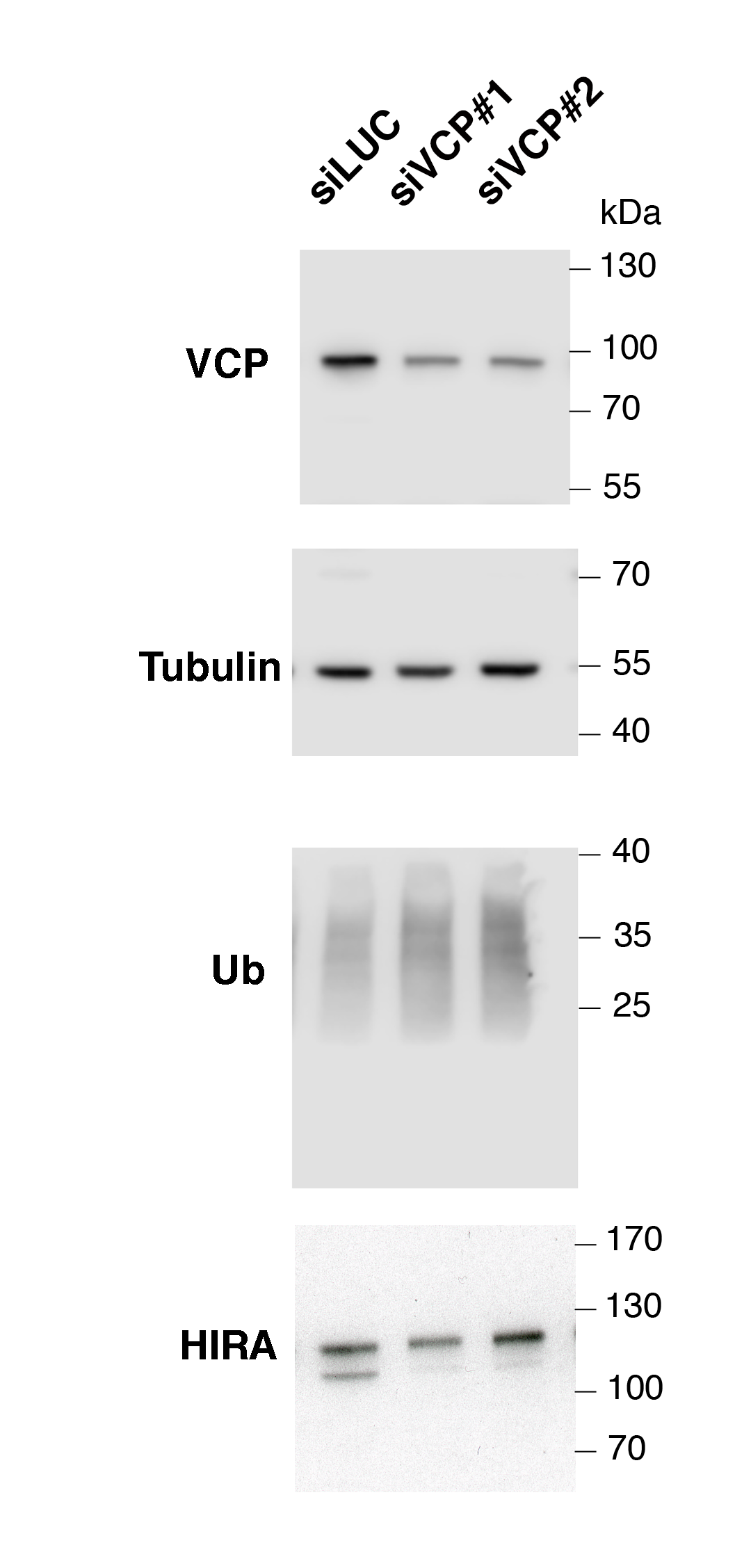

Supplement: Supplementary file 8 — Source Data [file 41467_2021_24153_MOESM8_ESM.zip › RawData/Main Figures/Fig3/a/WB.tif]

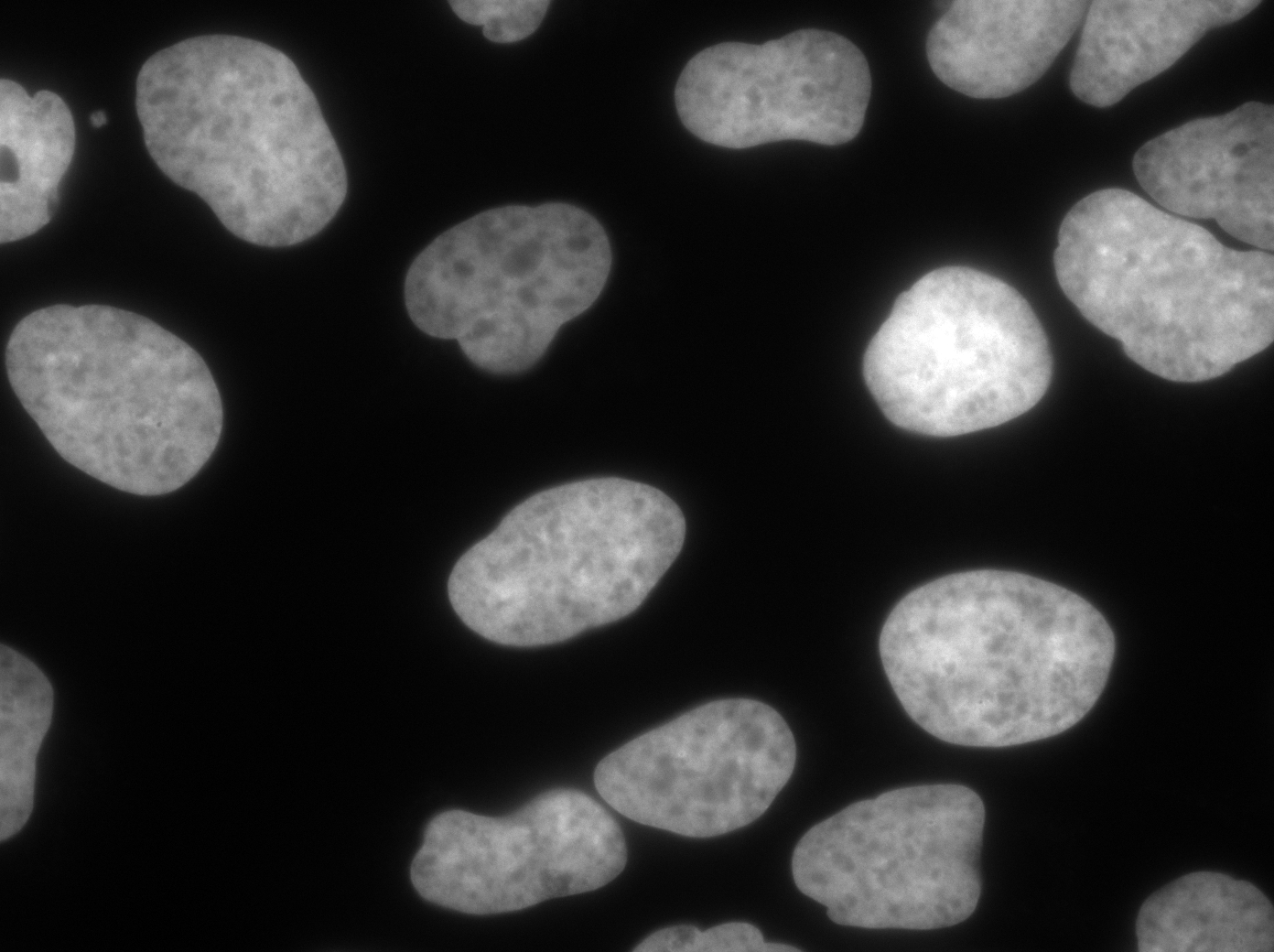

Supplement: Supplementary file 8 — Source Data [file 41467_2021_24153_MOESM8_ESM.zip › RawData/Main Figures/Fig3/b/hira_xpb_DMSO_3_w1DAPI.TIF]

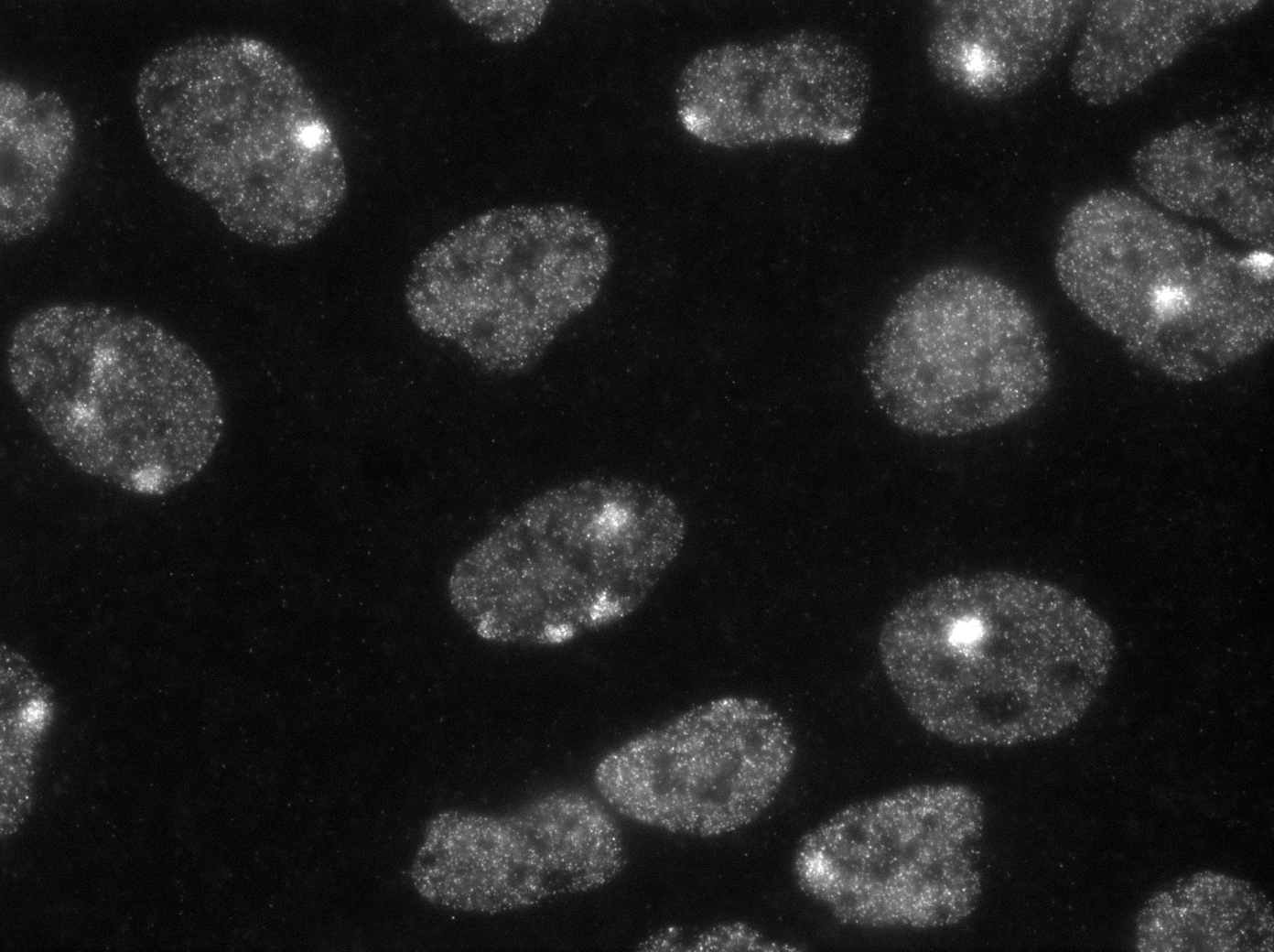

Supplement: Supplementary file 8 — Source Data [file 41467_2021_24153_MOESM8_ESM.zip › RawData/Main Figures/Fig3/b/hira_xpb_DMSO_3_w2GFP.TIF]

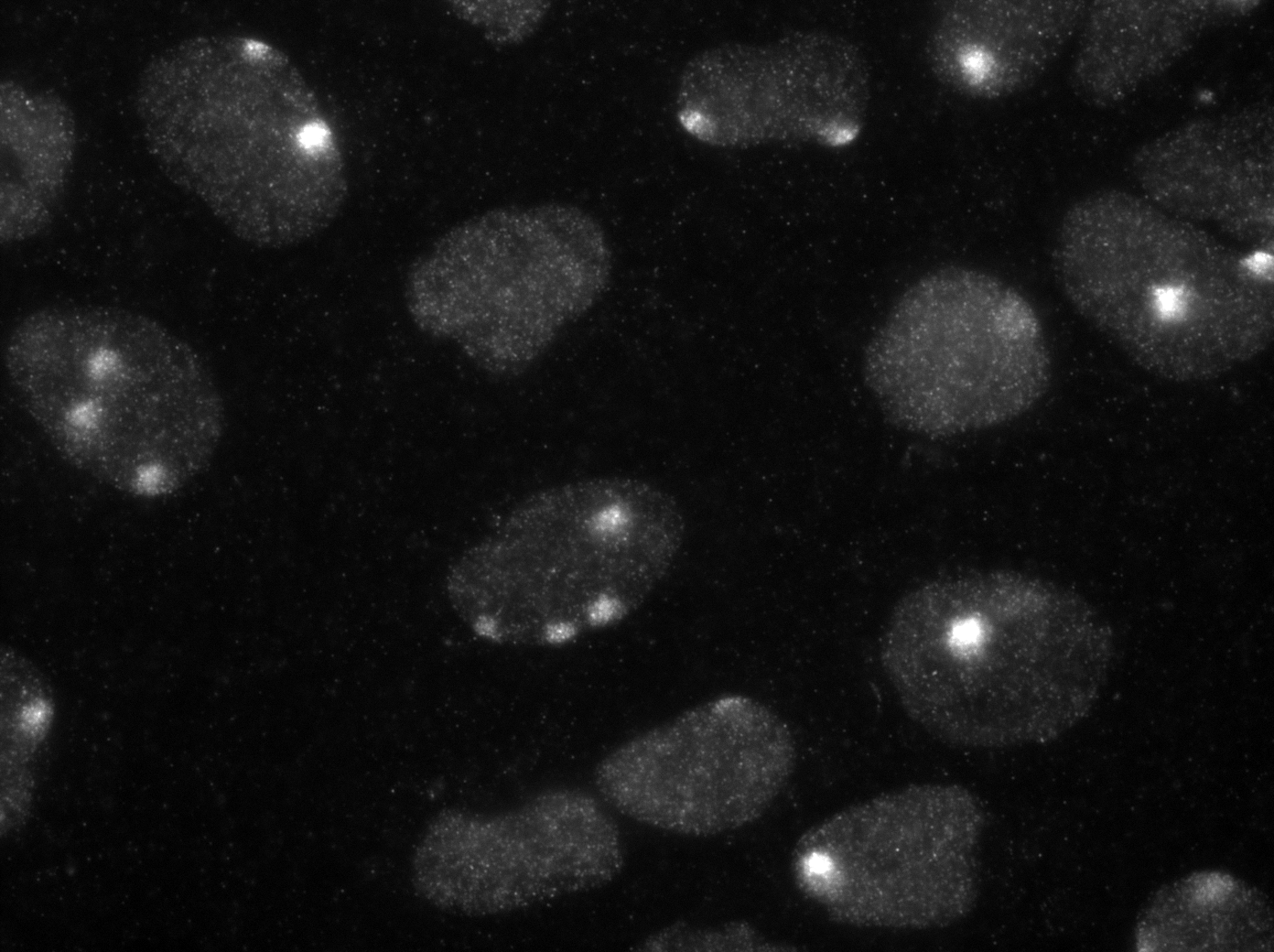

Supplement: Supplementary file 8 — Source Data [file 41467_2021_24153_MOESM8_ESM.zip › RawData/Main Figures/Fig3/b/hira_xpb_DMSO_3_w3TX.TIF]

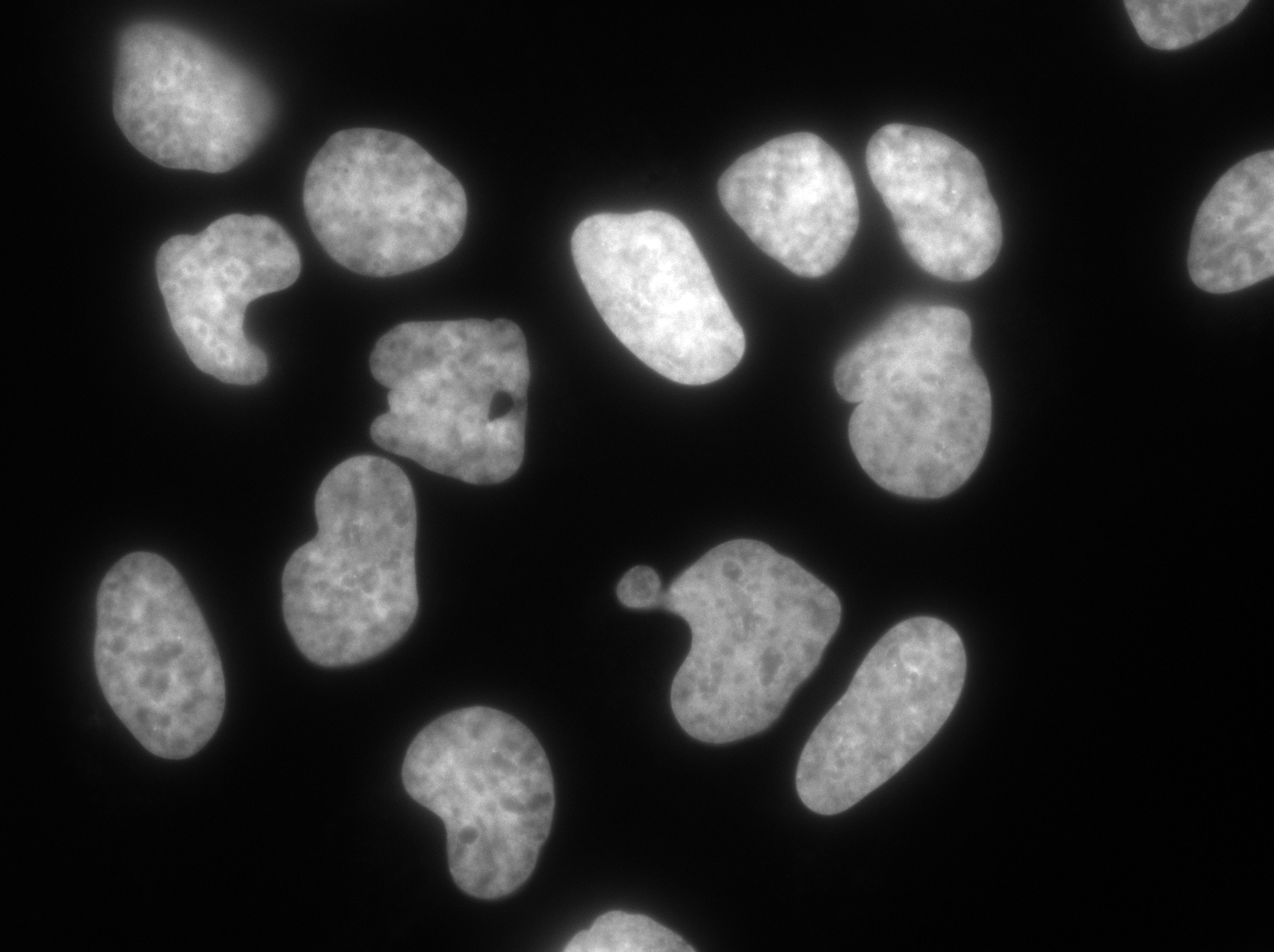

Supplement: Supplementary file 8 — Source Data [file 41467_2021_24153_MOESM8_ESM.zip › RawData/Main Figures/Fig3/b/hira_xpb_NMS_3_w1DAPI.TIF]

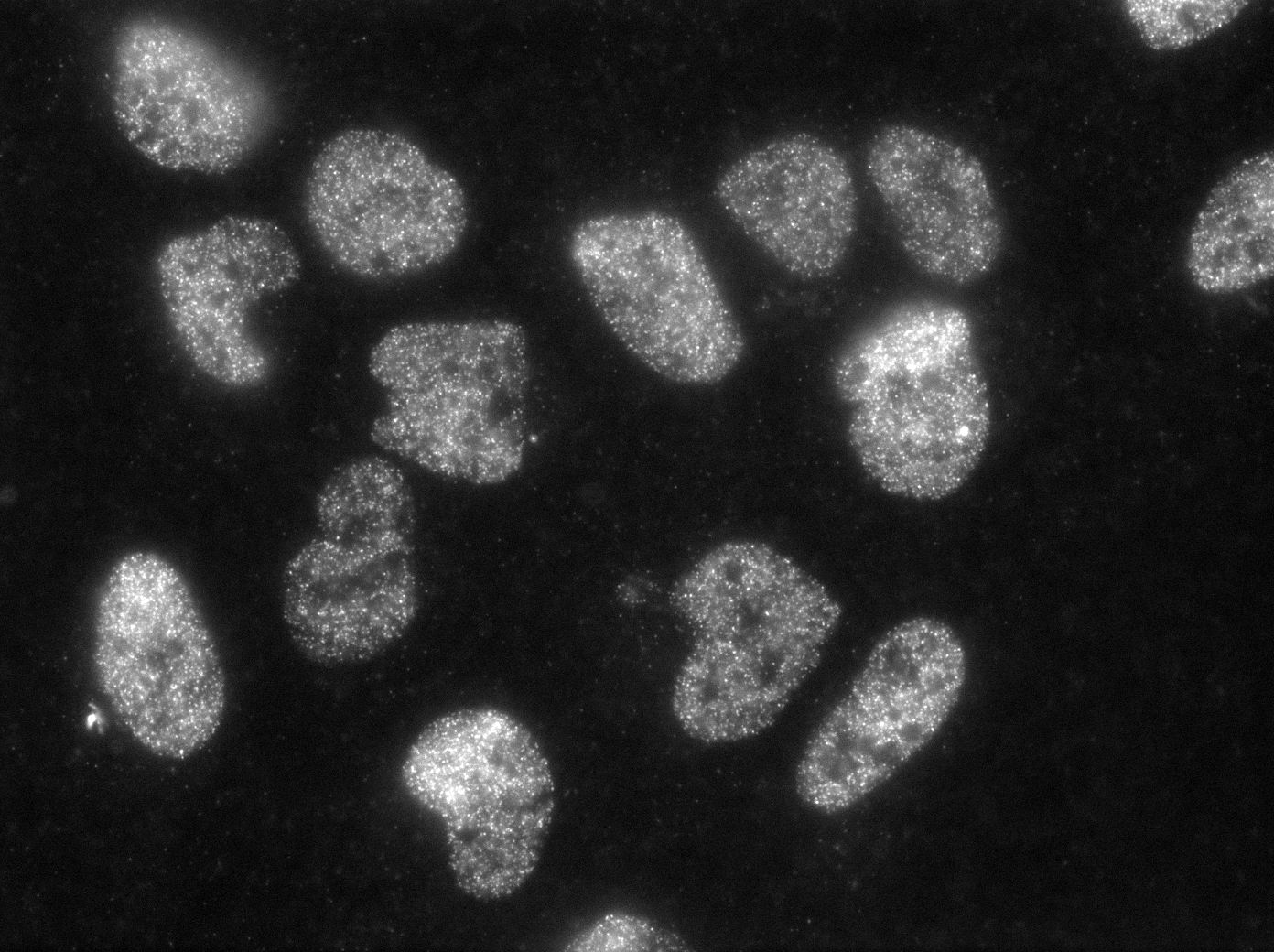

Supplement: Supplementary file 8 — Source Data [file 41467_2021_24153_MOESM8_ESM.zip › RawData/Main Figures/Fig3/b/hira_xpb_NMS_3_w2GFP.TIF]

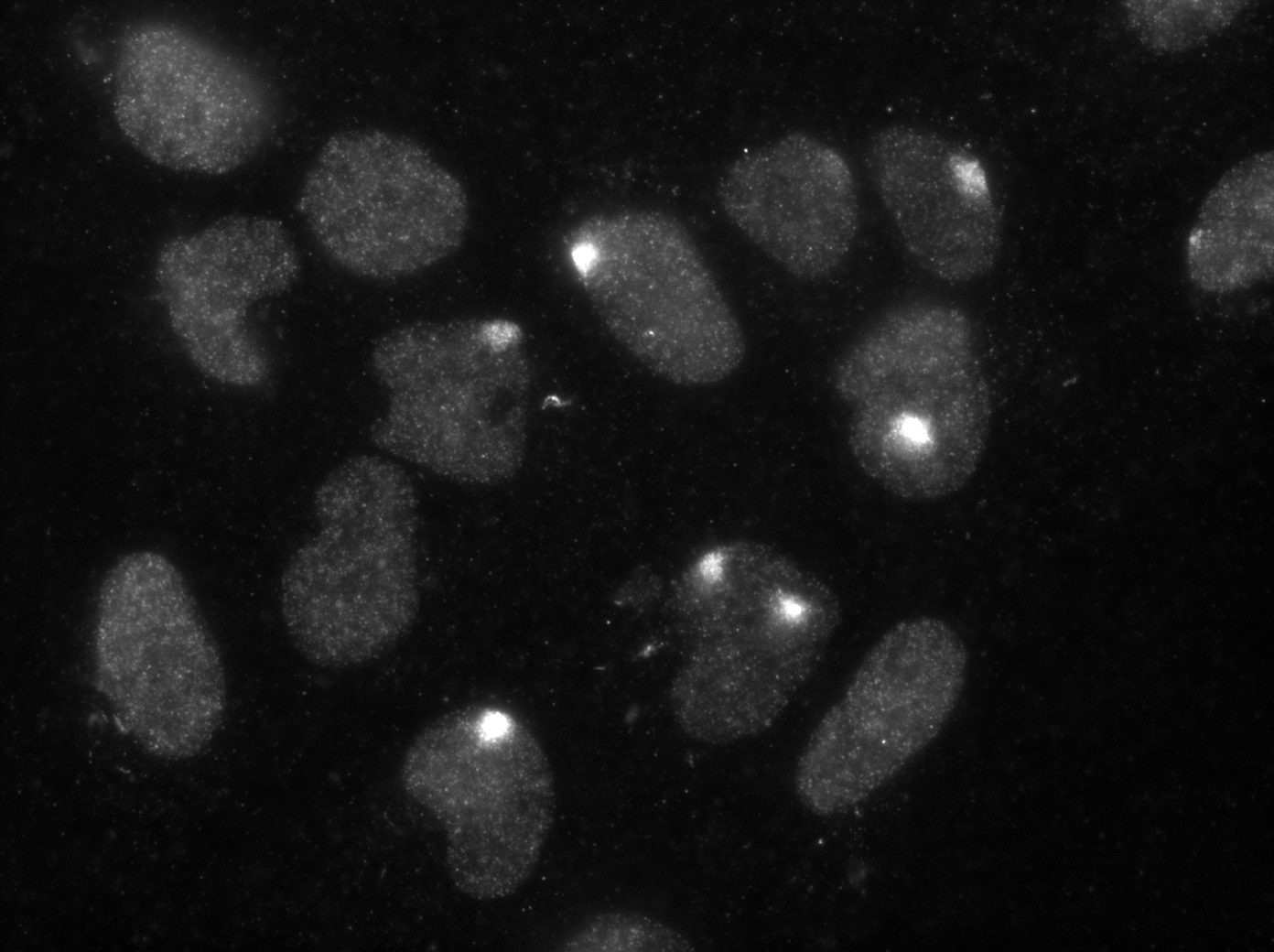

Supplement: Supplementary file 8 — Source Data [file 41467_2021_24153_MOESM8_ESM.zip › RawData/Main Figures/Fig3/b/hira_xpb_NMS_3_w3TX.TIF]

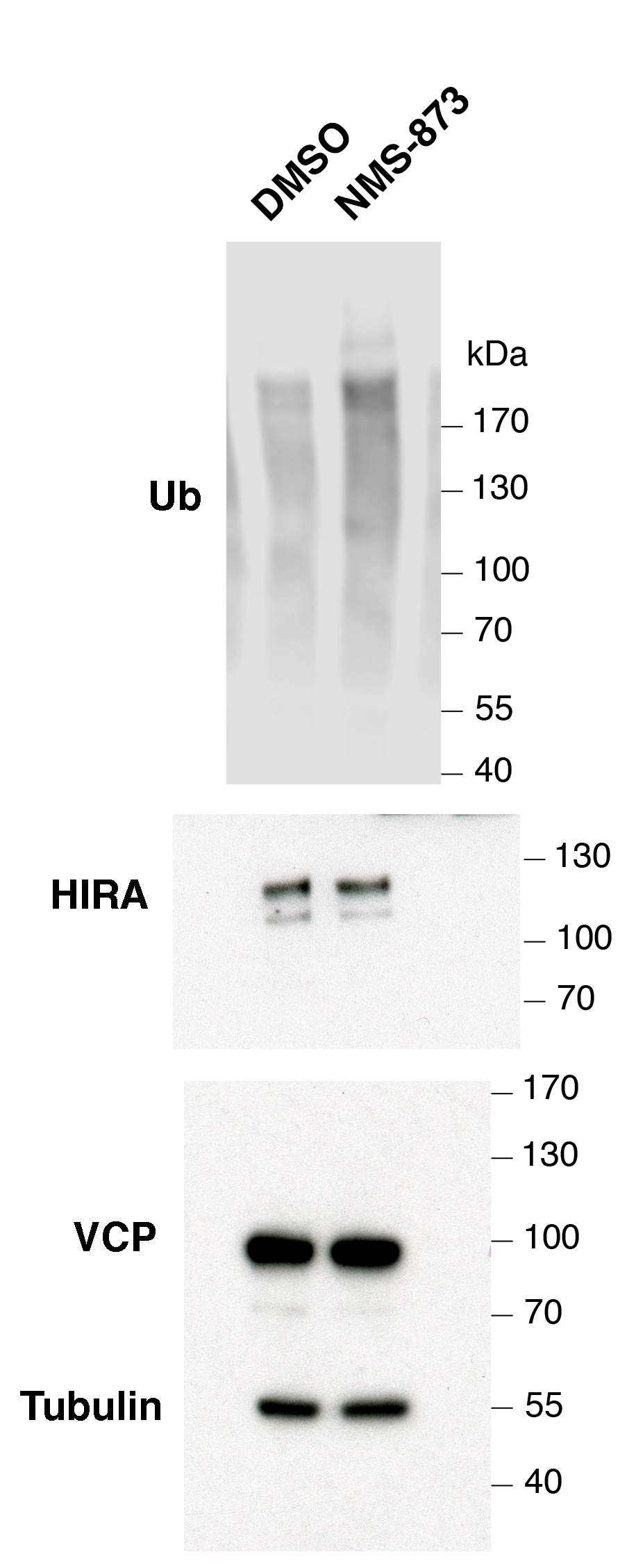

Supplement: Supplementary file 8 — Source Data [file 41467_2021_24153_MOESM8_ESM.zip › RawData/Main Figures/Fig3/b/WB.tif]

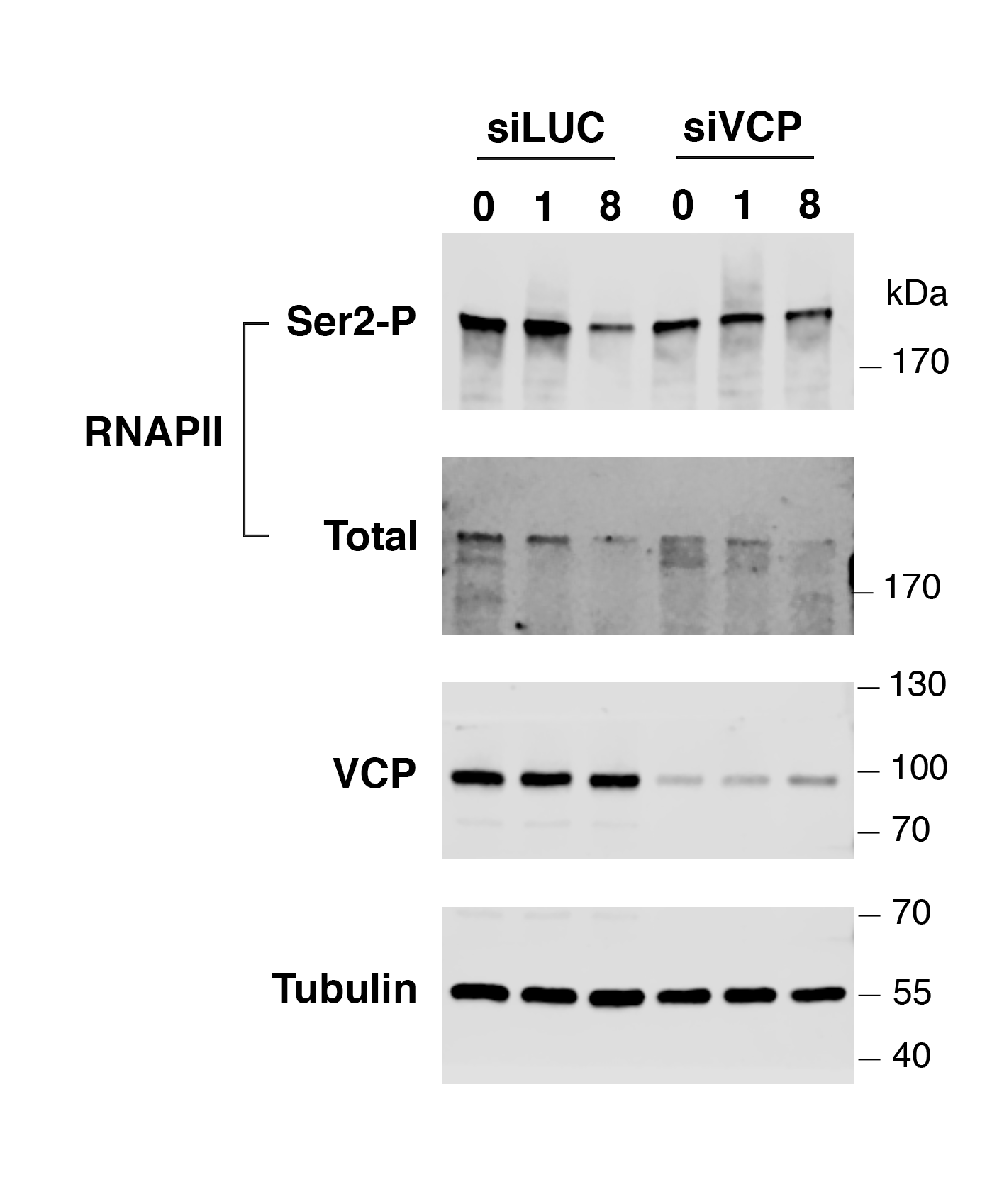

Supplement: Supplementary file 8 — Source Data [file 41467_2021_24153_MOESM8_ESM.zip › RawData/Main Figures/Fig3/c/WB.tif]

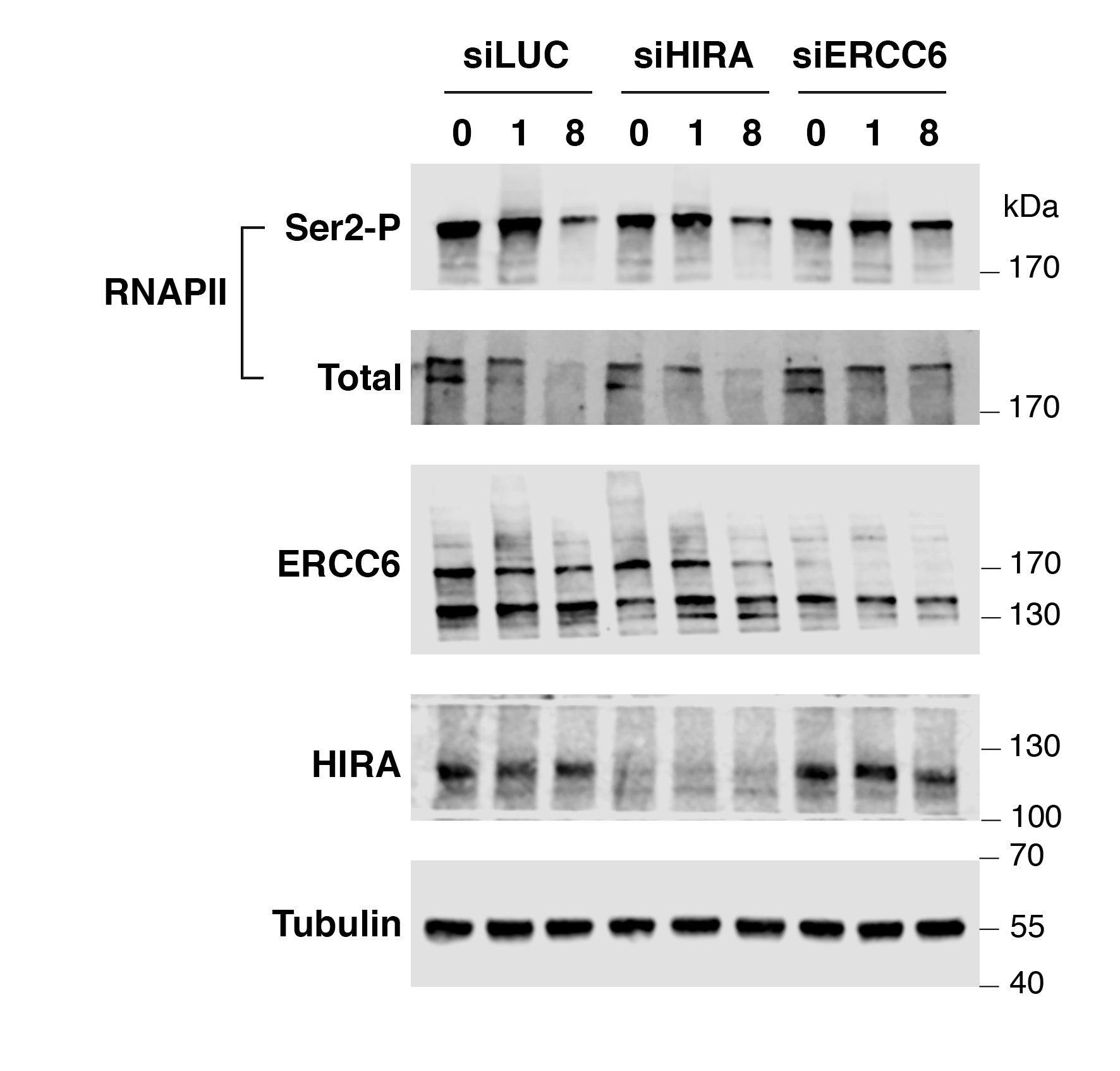

Supplement: Supplementary file 8 — Source Data [file 41467_2021_24153_MOESM8_ESM.zip › RawData/Main Figures/Fig3/d/WB.tif]

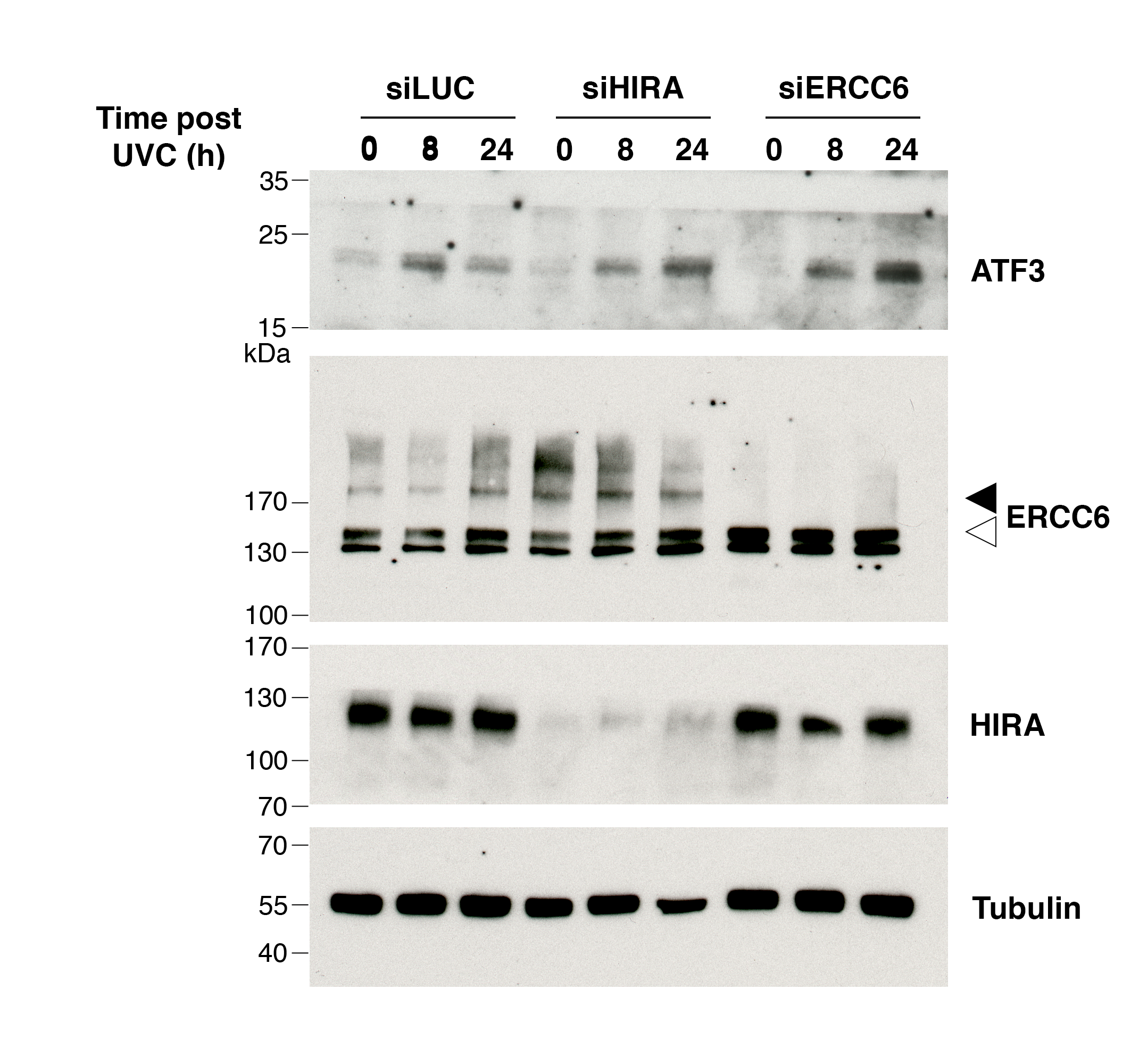

Supplement: Supplementary file 8 — Source Data [file 41467_2021_24153_MOESM8_ESM.zip › RawData/Main Figures/Fig6/a/WB.tif]

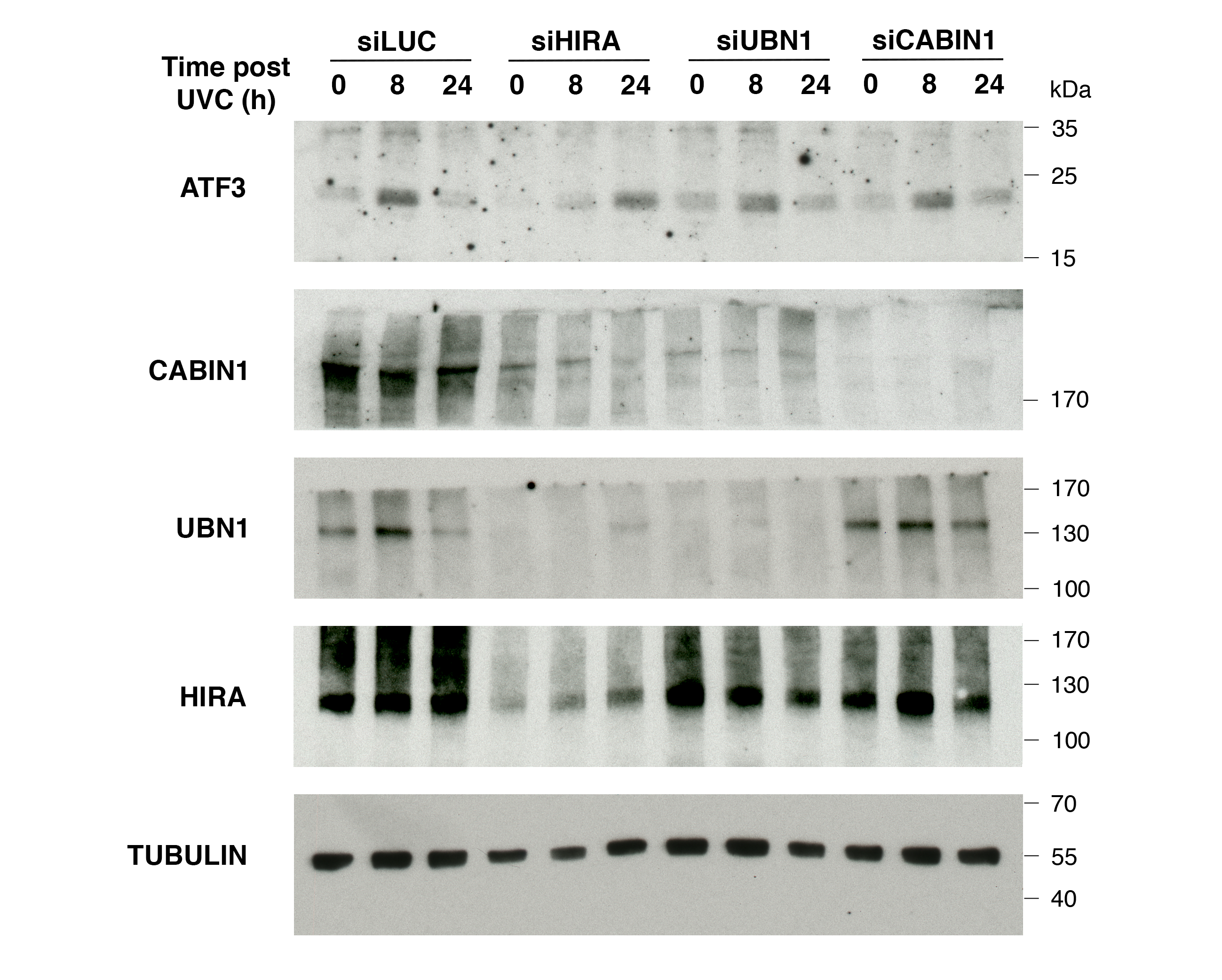

Supplement: Supplementary file 8 — Source Data [file 41467_2021_24153_MOESM8_ESM.zip › RawData/Main Figures/Fig6/d/WB.tif]

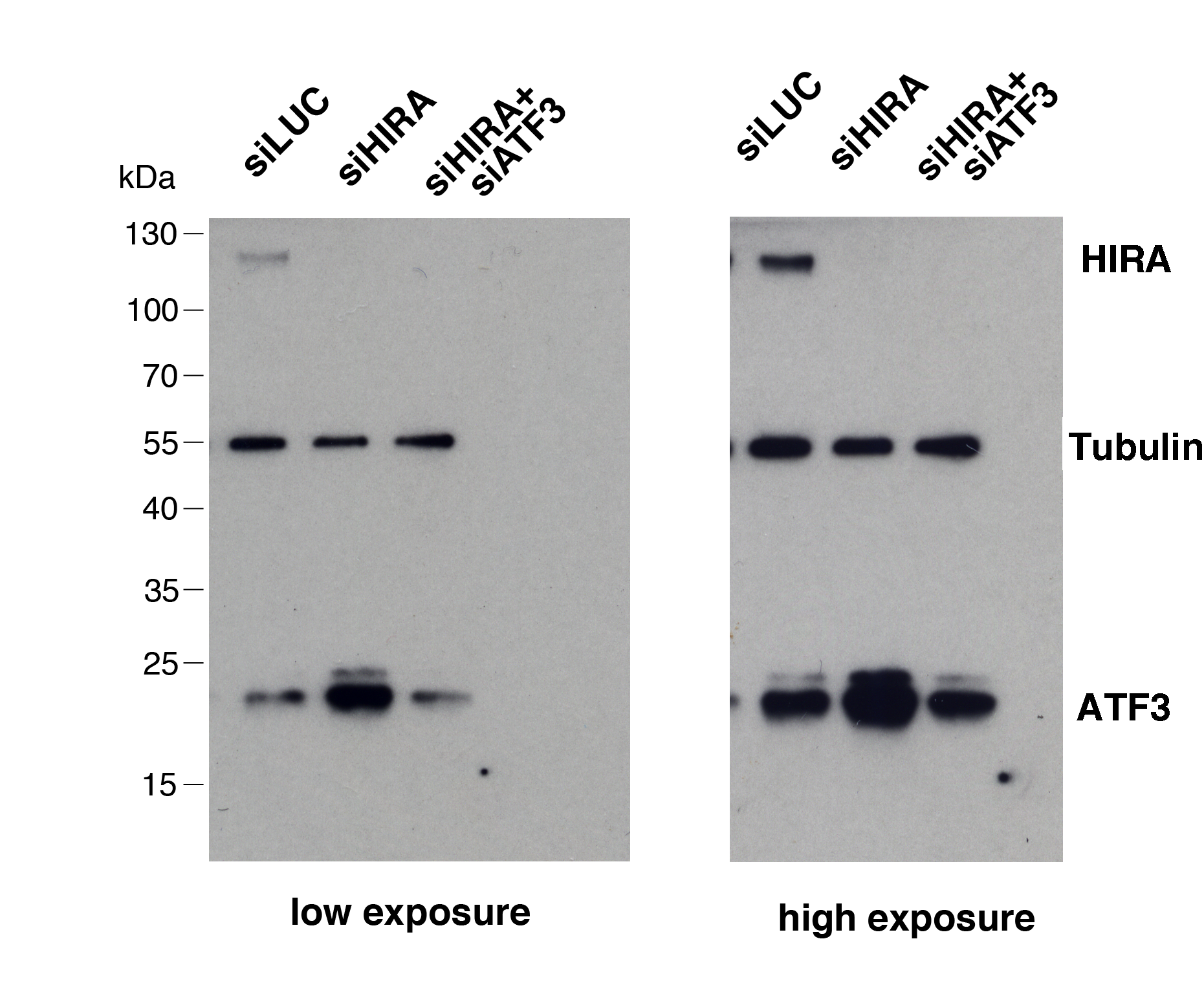

Supplement: Supplementary file 8 — Source Data [file 41467_2021_24153_MOESM8_ESM.zip › RawData/Main Figures/Fig6/h/WB.tif]

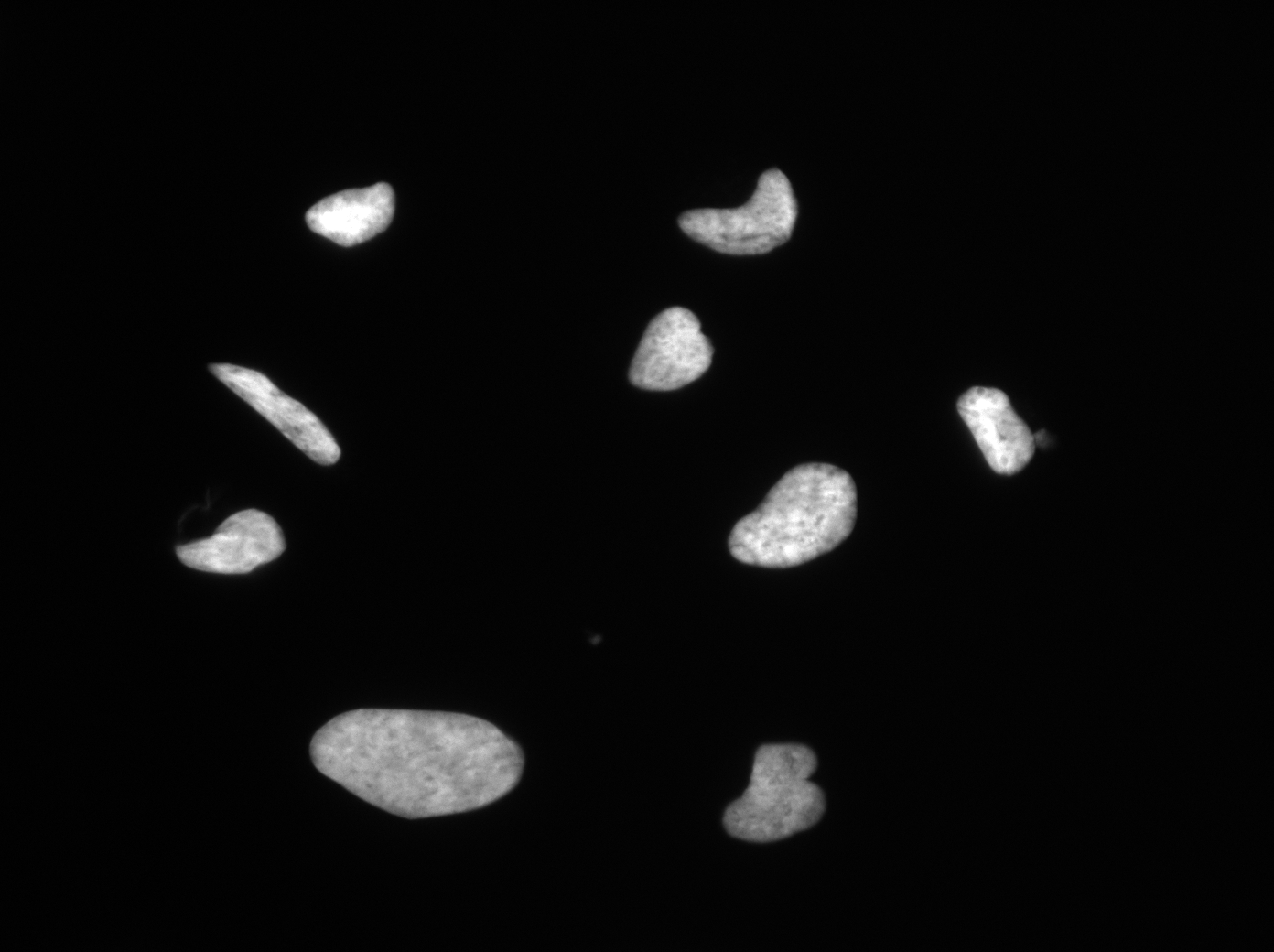

Supplement: Supplementary file 8 — Source Data [file 41467_2021_24153_MOESM8_ESM.zip › RawData/Main Figures/Fig6/j/U3S_150J_siHIRA_03_w1DAPI.TIF]

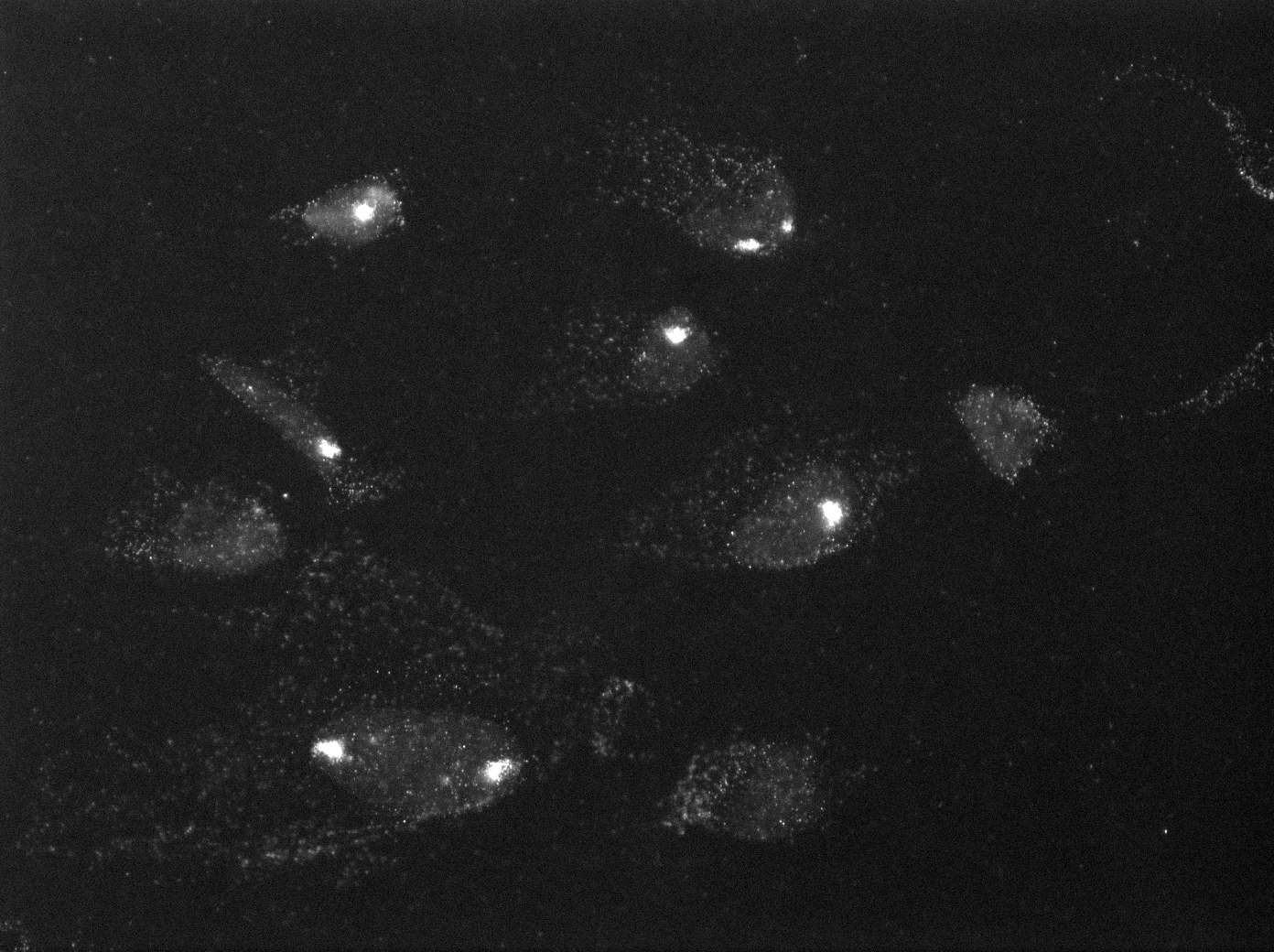

Supplement: Supplementary file 8 — Source Data [file 41467_2021_24153_MOESM8_ESM.zip › RawData/Main Figures/Fig6/j/U3S_150J_siHIRA_03_w2GFP.TIF]

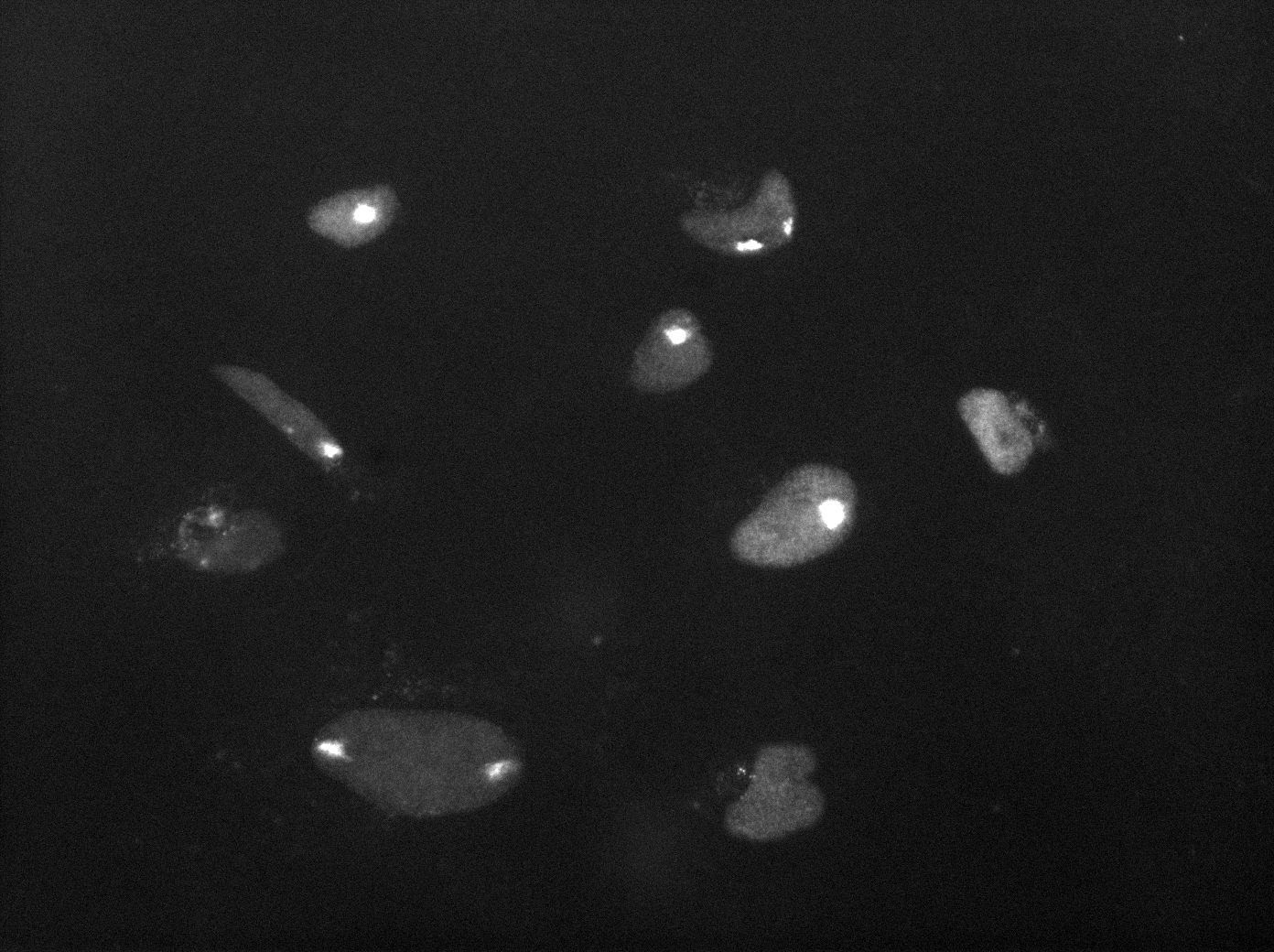

Supplement: Supplementary file 8 — Source Data [file 41467_2021_24153_MOESM8_ESM.zip › RawData/Main Figures/Fig6/j/U3S_150J_siHIRA_03_w3CY3.TIF]

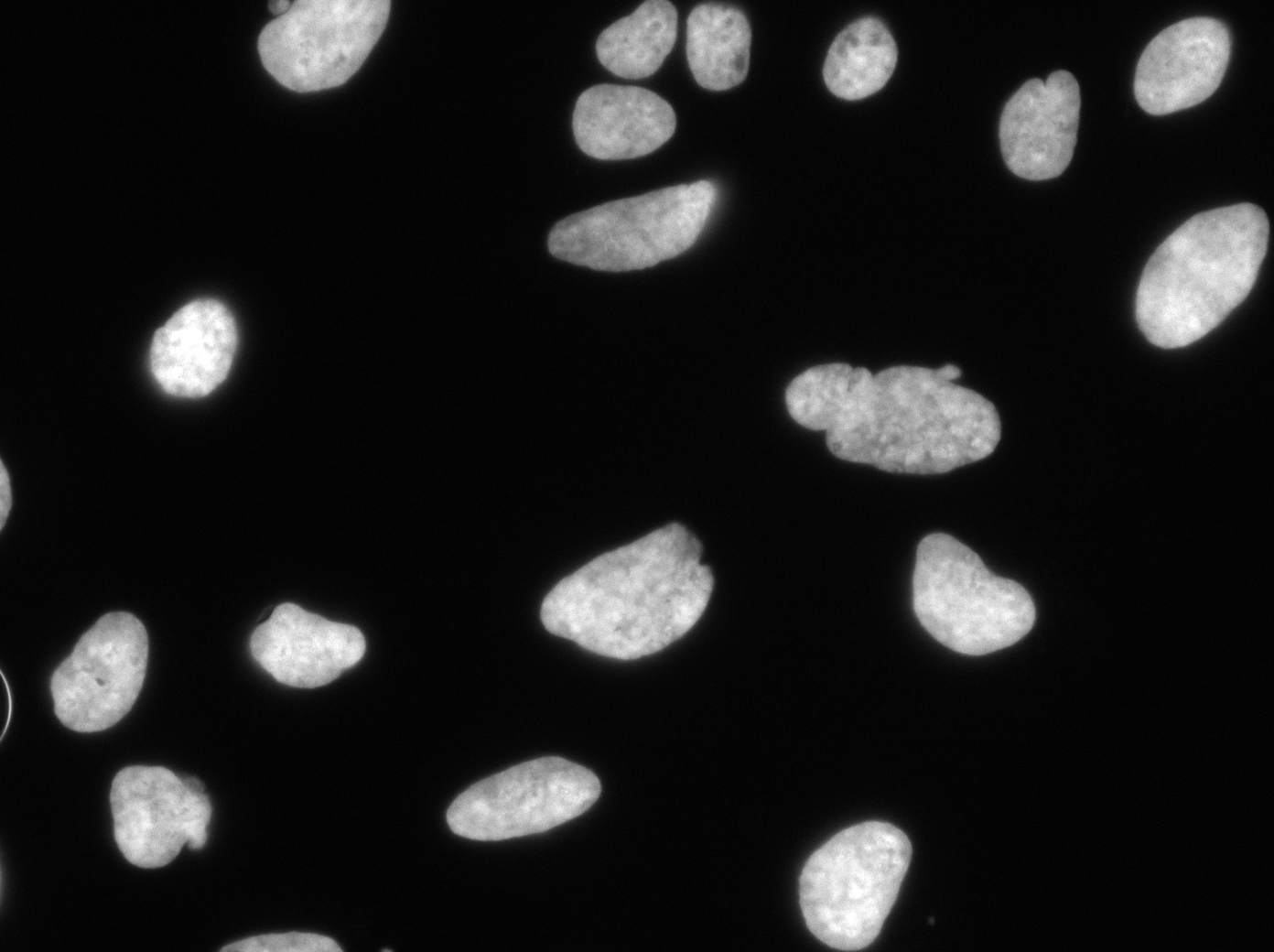

Supplement: Supplementary file 8 — Source Data [file 41467_2021_24153_MOESM8_ESM.zip › RawData/Main Figures/Fig6/j/U3S_150J_siHIRAplusATF3_09_w1DAPI.TIF]

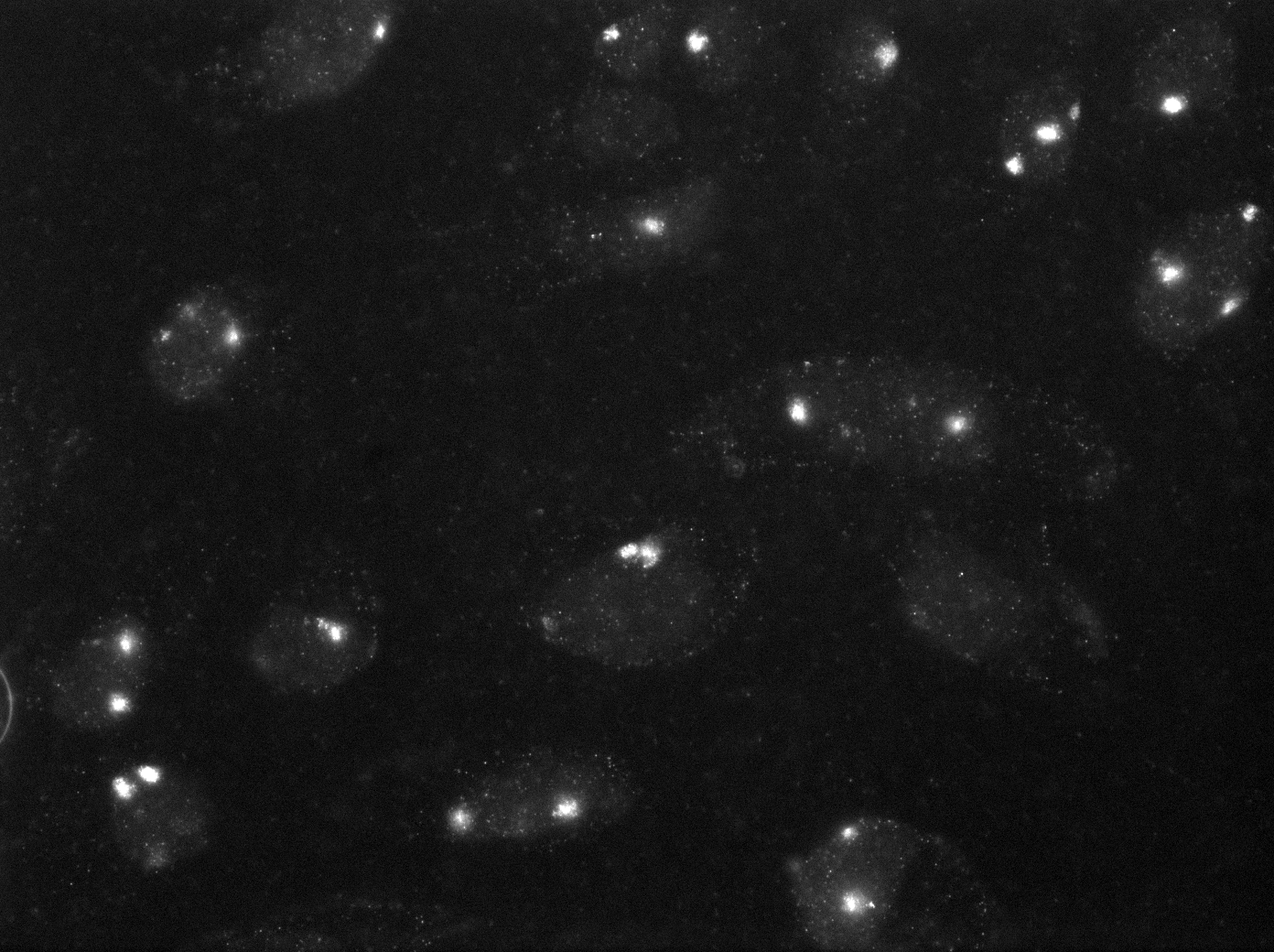

Supplement: Supplementary file 8 — Source Data [file 41467_2021_24153_MOESM8_ESM.zip › RawData/Main Figures/Fig6/j/U3S_150J_siHIRAplusATF3_09_w2GFP.TIF]
