# Supplementary material for: Dissecting regulatory pathways for transcription recovery following DNA damage reveals a non-canonical function of the histone chaperone HIRA
Source: Nat Commun. 2021 Jun 22;12:3835. doi: 10.1038/s41467-021-24153-1 (PMC8219801; doi:10.1038/s41467-021-24153-1)
Supplement: Supplementary file 8 — Source Data [file 41467_2021_24153_MOESM8_ESM.zip › RawData/Supplementary Figures/FigS4/d/Gorilla_analysis_results.docx]

94 common HIRA-inhibited genes exp1 & 2 vs common repressed genes exp1 & 2

GOrilla biological process

| **GO term** | **Description** | [**P-value**](http://cbl-gorilla.cs.technion.ac.il/GOrilla/ztt1be0u/GOResults.html#p_value_info) | [**FDR q-value**](http://cbl-gorilla.cs.technion.ac.il/GOrilla/ztt1be0u/GOResults.html#fdr_info) | [**Enrichment (N, B, n, b)**](http://cbl-gorilla.cs.technion.ac.il/GOrilla/ztt1be0u/GOResults.html#enrich_info) | [**Genes**](http://cbl-gorilla.cs.technion.ac.il/GOrilla/ztt1be0u/GOResults.html#genes_info) |
| --- | --- | --- | --- | --- | --- |
| [GO:0006333](http://www.godatabase.org/cgi-bin/amigo/go.cgi?query=GO:0006333&view=details) | chromatin assembly or disassembly | 5.5E-5 | 5.91E-1 | 11.60 (4259,27,68,5) | [[+] Show genes](javascript:toggle('elements_GO:0006333')) |
| [GO:0006334](http://www.godatabase.org/cgi-bin/amigo/go.cgi?query=GO:0006334&view=details) | nucleosome assembly | 7.89E-5 | 4.24E-1 | 10.80 (4259,29,68,5) | [[+] Show genes](javascript:toggle('elements_GO:0006334')) |
| [GO:0034723](http://www.godatabase.org/cgi-bin/amigo/go.cgi?query=GO:0034723&view=details) | DNA replication-dependent nucleosome organization | 1.3E-4 | 4.67E-1 | 26.84 (4259,7,68,3) | [[+] Show genes](javascript:toggle('elements_GO:0034723')) |
| [GO:0006335](http://www.godatabase.org/cgi-bin/amigo/go.cgi?query=GO:0006335&view=details) | DNA replication-dependent nucleosome assembly | 1.3E-4 | 3.5E-1 | 26.84 (4259,7,68,3) | [[+] Show genes](javascript:toggle('elements_GO:0006335')) |
| [GO:0000183](http://www.godatabase.org/cgi-bin/amigo/go.cgi?query=GO:0000183&view=details) | chromatin silencing at rDNA | 1.3E-4 | 2.8E-1 | 26.84 (4259,7,68,3) | [[+] Show genes](javascript:toggle('elements_GO:0000183')) |
| [GO:0034728](http://www.godatabase.org/cgi-bin/amigo/go.cgi?query=GO:0034728&view=details) | nucleosome organization | 4.29E-4 | 7.69E-1 | 7.64 (4259,41,68,5) | [[+] Show genes](javascript:toggle('elements_GO:0034728')) |
| [GO:0038111](http://www.godatabase.org/cgi-bin/amigo/go.cgi?query=GO:0038111&view=details) | interleukin-7-mediated signaling pathway | 4.31E-4 | 6.62E-1 | 18.79 (4259,10,68,3) | [[+] Show genes](javascript:toggle('elements_GO:0038111')) |
| [GO:0065004](http://www.godatabase.org/cgi-bin/amigo/go.cgi?query=GO:0065004&view=details) | protein-DNA complex assembly | 5.38E-4 | 7.23E-1 | 7.28 (4259,43,68,5) | [[+] Show genes](javascript:toggle('elements_GO:0065004')) |
| [GO:0051290](http://www.godatabase.org/cgi-bin/amigo/go.cgi?query=GO:0051290&view=details) | protein heterotetramerization | 5.86E-4 | 7E-1 | 17.08 (4259,11,68,3) | [[+] Show genes](javascript:toggle('elements_GO:0051290')) |
| [GO:0070593](http://www.godatabase.org/cgi-bin/amigo/go.cgi?query=GO:0070593&view=details) | dendrite self-avoidance | 7.46E-4 | 8.02E-1 | 41.75 (4259,3,68,2) | [[+] Show genes](javascript:toggle('elements_GO:0070593')) |
| [GO:0060968](http://www.godatabase.org/cgi-bin/amigo/go.cgi?query=GO:0060968&view=details) | regulation of gene silencing | 9.12E-4 | 8.91E-1 | 8.95 (4259,28,68,4) | [[+] Show genes](javascript:toggle('elements_GO:0060968')) |

GOrilla molecular function

| **GO term** | **Description** | [**P-value**](http://cbl-gorilla.cs.technion.ac.il/GOrilla/di8a21kj/GOResults.html#p_value_info) | [**FDR q-value**](http://cbl-gorilla.cs.technion.ac.il/GOrilla/di8a21kj/GOResults.html#fdr_info) | [**Enrichment (N, B, n, b)**](http://cbl-gorilla.cs.technion.ac.il/GOrilla/di8a21kj/GOResults.html#enrich_info) | [**Genes**](http://cbl-gorilla.cs.technion.ac.il/GOrilla/di8a21kj/GOResults.html#genes_info) |
| --- | --- | --- | --- | --- | --- |
| [GO:0031492](http://www.godatabase.org/cgi-bin/amigo/go.cgi?query=GO:0031492&view=details) | nucleosomal DNA binding | 1.54E-4 | 4.28E-1 | 13.92 (4259,18,68,4) | [[+] Show genes](javascript:toggle('elements_GO:0031492')) |
| [GO:0031491](http://www.godatabase.org/cgi-bin/amigo/go.cgi?query=GO:0031491&view=details) | nucleosome binding | 7.91E-4 | 1E0 | 9.28 (4259,27,68,4) | [[+] Show genes](javascript:toggle('elements_GO:0031491')) |
